# Supplementary material for: Metal-free electrochemical dihydroxylation of unactivated alkenes
Source: Nat Commun. 2023 Oct 13;14:6467. doi: 10.1038/s41467-023-42106-8 (PMC10575955; doi:10.1038/s41467-023-42106-8)
Supplement: Supplementary file 1 — Supplementary Information [file 41467_2023_42106_MOESM1_ESM.pdf]

# Supplementary Information

## Metal-Free Electrochemical Dihydroxylation of Unactivated Alkenes

**Min Liu[1], Tian Feng[1], Yanwei Wang[1], Guangsheng Kou[1], Qiuyan Wang[1]*, Qian Wang[1], and Youai Qiu[1]***

**[1]State Key Laboratory and Institute of Elemento-Organic Chemistry, Frontiers Science Center for New Organic Matter, College of Chemistry, Nankai University, 94 Weijin Road, Tianjin, 300071, China**

***qiuyouai@nankai.edu.cn**

## Table of Contents

**Supplementary Methods** .......... S2

**General Remarks** .......... S2

**Optimization of the Reaction Conditions** .......... S3

**Preparation of Starting Materials** .......... S7

**General Procedure for Metal-Free Electrochemical Dihydroxylation of Unactivated Alkenes** .......... S10

**General Procedure for Electrochemical Epoxidation of Unactivated Alkenes.** .......... S11

**Gram-Scale Reaction** .......... S12

**Application of Vicinal Diols** .......... S13

**Mechanistic Experiments** .......... S14

**Synthesis of 4** .......... S14

**Isotope labeling experiments** .......... S15

**Control experiment of tertiary ammonium** .......... S17

**Cyclic Voltammetry** .......... S17

**Characterization Data of Products** .......... S21

**X-ray Crystallography of 2y** .......... S47

**NMR Spectra** .......... S49

**Supplementary References** .......... S117

# Supplementary Methods

## General Remarks

Catalytic reactions were carried out in undivided electrochemical cells (15 mL) using pre-dried glassware, if not noted otherwise. Solvents were obtained from commercial sources. All the starting materials were obtained from commercial sources or synthesized according to literature methods (>95% purity). Commercially available chemicals were obtained from *Bide Pharmatech Ltd, Tianjin Heowns OPDE Technologies and Shanghai Macklin Biochemical Co* used as received unless otherwise stated. Iron plate electrodes (0.2 mm × 10.0 mm × 20.0 mm, 99.9%; obtained from Dingsheng scientific research metal materials, Hebei, China), nickel foam electrodes (0.3 mm × 10.0 mm × 20.0 mm, 99.9%; obtained from Guangjiayuan electronic materials Jiangsu, China), were connected using stainless steel adapters. Electrocatalysis was conducted using an HSPY-36-03 potentiostat in constant current mode. Cyclic Voltammetry studies and square wave voltammetry were performed using a Shanghai Chenhua CHI760E workstation. Yields refer to isolated compounds, estimated to be >95% purity as determined by $^{1}$H-NMR. Flash chromatography was performed using Silica gel (200-300 mesh) purchased from Qingdao Haiyang Chemical Co., China. NMR spectra were recorded on Bruker AVANCE AV 400 or 600 in the solvent indicated; chemical shifts are given in ppm relative to the residual solvent peak. The HRMS data were collected on a MicrOTOF mass spectrometer with ESI mass analyzer.

## Optimization of the Reaction Conditions

Supplementary Table 1: Solvent screening[a]

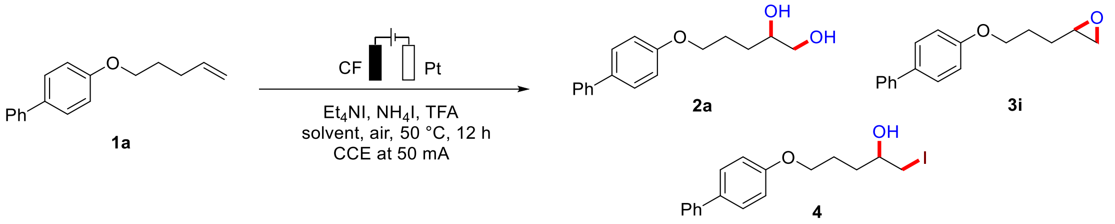

| Entry | Variation | Yield %[b] | | |
|---|---|---|---|---|
| | | **2a** | **3i** | **4** |
| **1** | **$^t$BuOH: $H_2O$ (3:1)** | **80** | **12** | **5** |
| 2 | DMF:$H_2O$ (3:1) | n.d. | 69 | Trace |
| 3 | DMA:$H_2O$ (3:1) | n.d. | 45 | Trace |
| 4 | DMSO:$H_2O$ (3:1) | n.d. | n.d. | n.d. |
| **5** | **DMI:$H_2O$ (3:1)** | **n.d.** | **86** | **Trace** |
| 6 | MeCN:$H_2O$ (3:1) | n.d. | n.d. | n.d. |
| 7 | $^t$AmOH: $H_2O$ (3:1) | n.d. | n.d. | n.d. |

[a] Reaction conditions: undivided cell, **1a** (0.3 mmol), $Et_4NI$ (0.6 mmol, 2.0 equiv.), $NH_4I$ 0.6 mmol, 2.0 equiv.), TFA (0.9 mmol, 3.0 equiv.), solvent (4 mL) under 50 mA constant current in air at 50 °C for 12 h with an CF as the anode and Pt as the cathode. [b] Isolated yield of product. DMI = 1,3-dimethyl-2-imidazolidinone, CF = carbon felt, TFA = Trifluoroacetic acid.

Supplementary Table 2: Electrolyte screening[a]

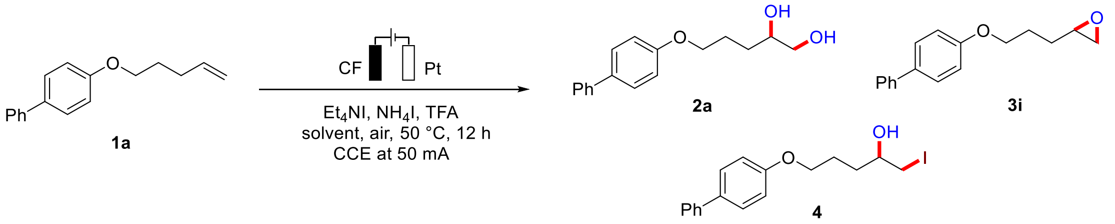

| Entry | Variation | | Yield %[b] | | |
|---|---|---|---|---|---|
| | | | **3** | **3i** | **4** |
| **1** | **$Et_4NI$** | **$NH_4I$** | **80** | **12** | **5** |
| 2[c] | $Et_4NI$ | $NH_4I$ | 78 | 17 | Trace |
| 3 | $Et_4NI$ | LiI | 78 | 21 | Trace |
| 4 | $Et_4NI$ | NaI | n.d. | n.d. | n.d. |

| | | | | | |
|---|---|---|---|---|---|
| 5 | $Et_4NI$ | KI | n.d. | n.d. | n.d. |
| 6 | $Et_4NBr$ | $NH_4Br$ | 32 | 21 | 17 |
| 7 | $Et_4NCl$ | $NH_4Cl$ | n.d. | n.d. | n.d. |

[a] Reaction conditions: undivided cell, **1a** (0.3 mmol), electrolyte 1 (0.6 mmol, 2.0 equiv.), electrolyte 2 (0.6 mmol, 2.0 equiv.), TFA (0.9 mmol, 3.0 equiv.), $^{t}$BuOH:$H_2O$ (3;1) (4 mL) under 50 mA constant current in air at 50 °C for 12 h with an CF as the anode and Pt as the cathode. [b] Isolated yield of product. [c] In an argon atmosphere. CF = carbon felt, TFA = Trifluoroacetic acid.

Supplementary Table 3: Additives screening[a]

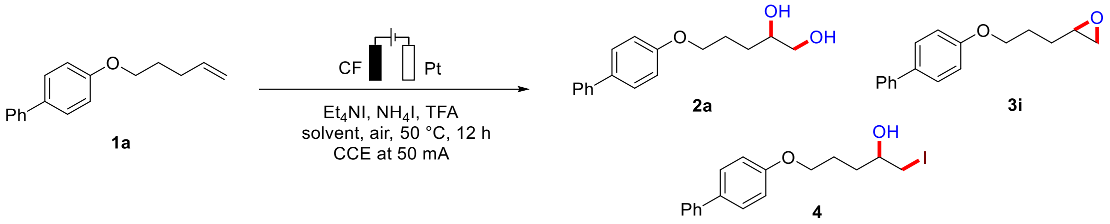


| Entry | Variation | Yield % [b] | | |
|---|---|---|---|---|
| | | **3** | **3i** | **4** |
| **1** | **TFA** | **80** | **12** | **5** |
| 2 | HOAc | 71 | 17 | 13 |
| 3 | PivOH | 63 | 27 | Trace |
| 4 | $AlCl_3$ | 44 | 37 | 13 |
| 5 | $K_2CO_3$ | n.d. | n.d. | n.d. |
| 6 | $Cs_2CO_3$ | n.d. | n.d. | n.d. |

[a] Reaction conditions: undivided cell, **1a** (0.3 mmol), $Et_4NI$ (0.6 mmol, 2.0 equiv.), $NH_4I$ 0.6 mmol, 2.0 equiv.), additive (0.9 mmol, 3.0 equiv.), $^{t}$BuOH:$H_2O$ (3:1) (4 mL) under 50 mA constant current in air at 50 °C for 12 h with an CF as the anode and Pt as the cathode. [b] Isolated yield of product. CF = carbon felt, TFA = Trifluoroacetic acid.

Supplementary Table 4: Current screening[a]

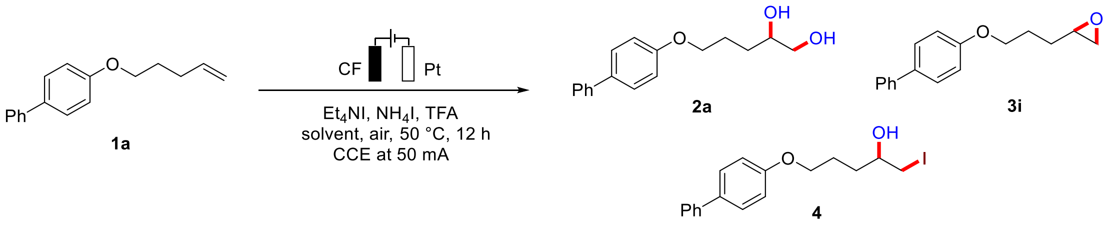


| | | Yield %[a] |
|---|---|---|

| Entry | Variation | 3 | 3i | 4 |
|---|---|---|---|---|
| 1 | 10 mA | n.d. | n.d. | n.d. |
| 2 | 20 mA | n.d. | n.d. | n.d. |
| 3 | 30 mA | 13 | 27 | 15 |
| 4 | 40 mA | 37 | 23 | 6 |
| **5** | **50 mA** | **80** | **12** | **4** |
| 6 | 80 mA | 55 | Trace | Trace |
| 7 | No current | n.d. | n.d. | n.d. |

[a] Reaction conditions: undivided cell, **1** (0.3 mmol), $Et_4NI$ (0.6 mmol, 2.0 equiv.), $NH_4I$ 0.6 mmol, 2.0 equiv.), TFA (0.9 mmol, 3.0 equiv.), $^t$BuOH:$H_2O$ (3:1) (4 mL) under constant current in air at 50 °C for 12 h with an CF as the anode and Pt as the cathode. [b] Isolated yield of product. CF = carbon felt, TFA = Trifluoroacetic acid.

Supplementary Table 5: Temperature screening[a]

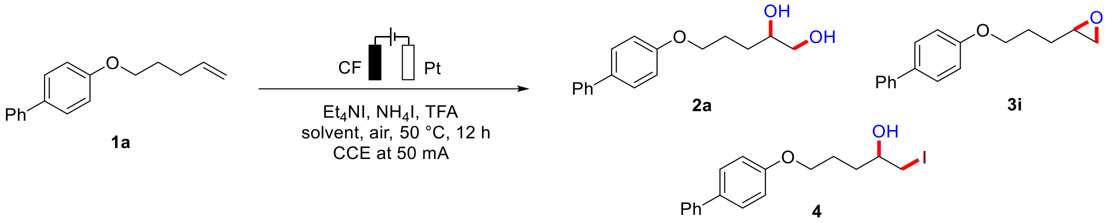


| Entry | Variation | Yield % [b] | | |
|---|---|---|---|---|
| | | 3 | 3i | 4 |
| 1 | r.t. | 47 | n.d. | n.d. |
| **2** | **50 °C** | **80** | **12** | **5** |
| 3 | 80 °C | 62 | Trace | Trace |
| 4 | 100 °C | 47 | Trace | Trace |

[a] Reaction conditions: undivided cell, **1** (0.3 mmol), $Et_4NI$ (0.6 mmol, 2.0 equiv.), $NH_4I$ 0.6 mmol, 2.0 equiv.), TFA (0.9 mmol, 3.0 equiv.), $^t$BuOH:$H_2O$ (3:1) (4 mL) under constant current in air for 12 h with an CF as the anode and Pt as the cathode. [b] Isolated yield of product. r.t. = room temperature, CF = carbon felt, TFA = Trifluoroacetic acid.

Supplementary Table 6: The ratio of $H_2O$ and $^tBuOH$ screening[a]

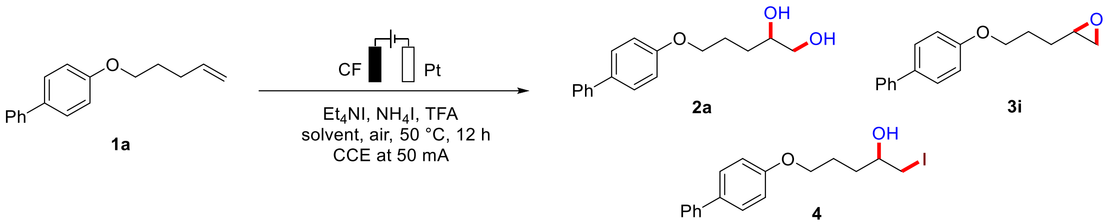


| Entry | Variation | Yield %[b] | | |
|---|---|---|---|---|
| | | **3** | **3i** | **4** |
| 1 | $^tBuOH$: $H_2O$ (4:0.1) | n.d. | n.d. | n.d. |
| 2 | $^tBuOH$: $H_2O$ (4:0.5) | n.d. | n.d. | n.d. |
| 3 | $^tBuOH$: $H_2O$ (4:1) | 41 | Trace | Trace |
| **4** | **$^tBuOH$: $H_2O$ (3:1)** | **80** | **12** | **5** |
| 5 | **$^tBuOH$: $H_2O$ (1:1)** | 56 | Trace | Trace |
| 6 | No $H_2O$ | n.d. | n.d. | n.d. |

[a] Reaction conditions: undivided cell, **1** (0.3 mmol), $Et_4NI$ (0.6 mmol, 2.0 equiv.), $NH_4I$ 0.6 mmol, 2.0 equiv.), TFA (0.9 mmol, 3.0 equiv.), $^tBuOH$:$H_2O$ (4 mL) under constant current in air at 50 °C for 12 h with an CF as the anode and Pt as the cathode. [b] Isolated yield of product. CF = carbon felt, TFA = Trifluoroacetic acid.

## Preparation of Starting Materials

### General procedure for the preparation of 1a-1q, 1w, 1x, 1ao (1i as an example):

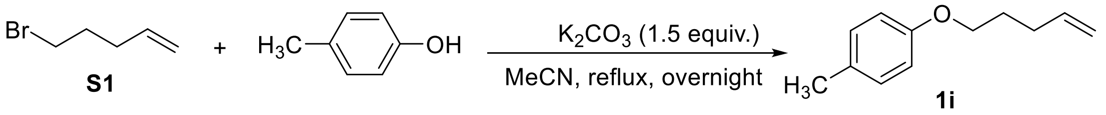


Method **A**.[1] A mixture of **S1** (581 mg, 3.9 mmol, 1.3 equiv.), 4-methylphenol (324 mg, 3 mmol, 1.0 equiv.), and $K_2CO_3$ (621 mg, 4.5 mmol, 1.5 equiv.) in anhydrous acetonitrile (30 mL) was heated to reflux in an oil bath. After refluxing overnight, the reaction mixture was cooled to room temperature, concentrated under reduced pressure, diluted with saturated $NH_4Cl$ (30 mL) and extracted with ethyl acetate (30 mL × 3). The combined organic layers were dried with $Na_2SO_4$, filtered, and concentrated under reduced pressure. The residue was purified by silica gel column chromatography (petroleum ether) to afford the corresponding pure product.

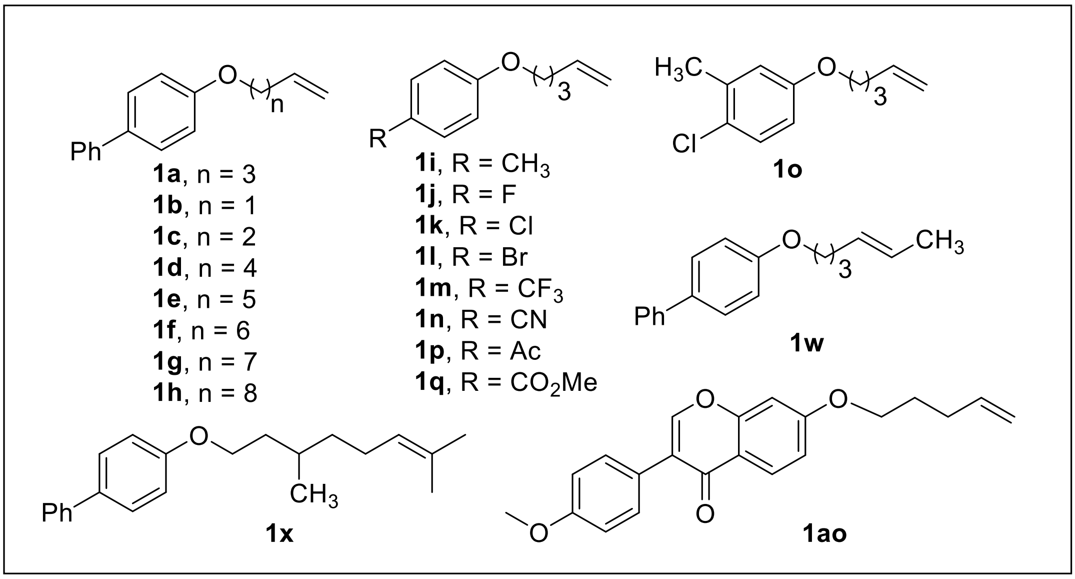


**Supplementary Figure 1.** Substrates synthesized according to general procedure **A**

### General procedure for the preparation of 1s, 1ah, 1aj, 1ak, 1al, 1am,1an 1ap, 1aq, 1ar.

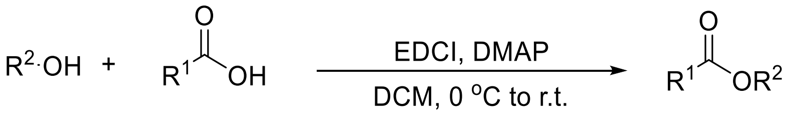

Method **B**.[2] The corresponding acid (10 mmol) was added to a solution of 1-(3-dimethylaminopropyl)-3-ethylcarbodiimide (EDCI, 1.3 equiv.) and DMAP (0.1 equiv.) in $CH_2Cl_2$ (25 mL) at 0 °C. Alcohol (1.2 equiv.) was then added. The reaction mixture was allowed to warm to room temperature overnight. The solution was diluted with $CH_2Cl_2$ (40 mL) and washed with 1N HCl (3×20 mL), saturated $NaHCO_3$ (40 mL), brine (40 mL) sequentially. The organic layer was dried over anhydrous $Na_2SO_4$. After removal of solvent under reduced pressure, the crude product was purified by column chromatography on silica gel.

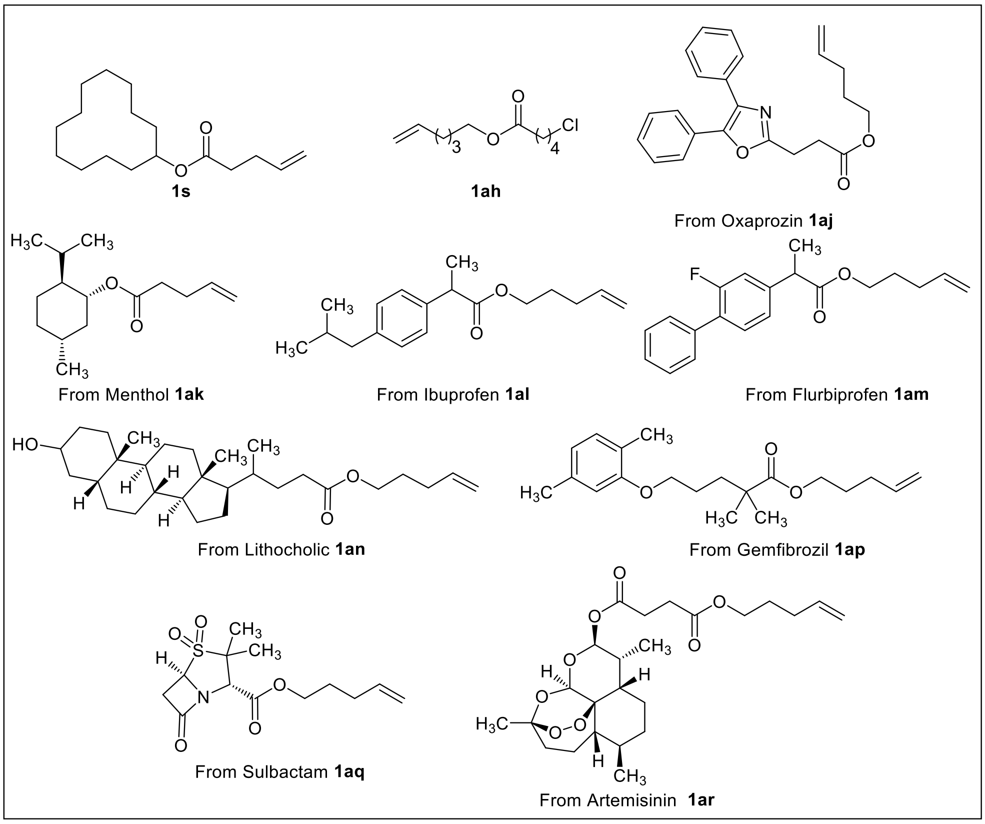


**Supplementary Figure 2.** Substrates synthesized according to general procedure **C**

**General procedure for the preparation of 1v.**

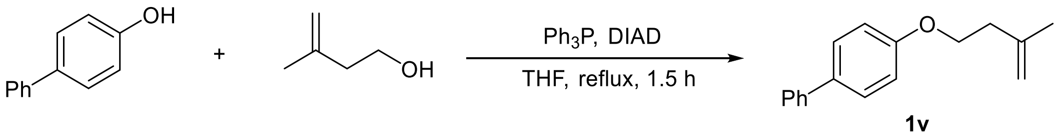


**1v**

Method **C**.[3] A solution of phenol (769 mg, 8.18 mmol), 3-methyl-3-buten-1-ol (0.28 mL, 2.73 mmol), triphenylphosphine (927 mg, 3.54 mmol), diisopropyl

azodicarboxylate (40% in toluene, 1.9 mL, 3.54 mmol) in THF (27 mL) for 1.5 h at reflux. After concentrated in vacuo, the residue was purified by flash column chromatography to give **1v**.

**General procedure for the preparation of 1r.**

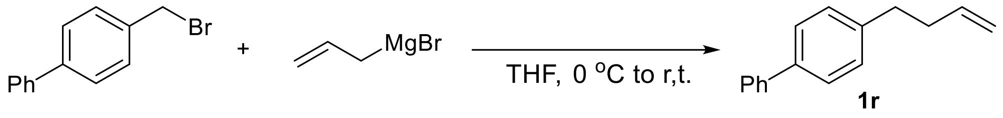


Method **D**.[4] Benzyl bromide (1.0 equiv.) and 20 mL anhydrous THF were added to a flame dried 100 mL round-bottom flash assembled with a constant pressure funnel which had been previously flame dried. Allyl magnesium bromide (2.0 equiv.) in the constant pressure funnel were added dropwise. The reaction was stirred for 4 h at room temperature and then quenched with saturated aqueous $NH_4Cl$. The aqueous layer was extracted with $CH_2Cl_2$ and the combined organics were dried over $MgSO_4$, filtered through celite and concentrated under reduced pressure. The crude product was purified by flash chromatography (eluent: hexane), affording the desired product as a clear colorless oil.

**General Procedure for Metal-Free Electrochemical Dihydroxylation of Unactivated Alkenes**

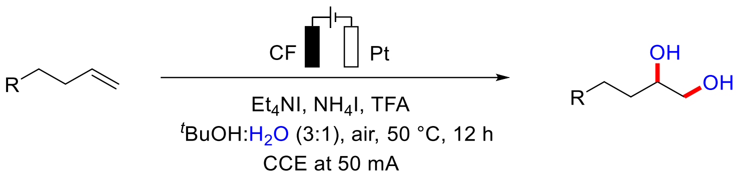


The electrocatalysis was carried out in an undivided cell with a carbon felt (CF) anode (5.0 mm × 10.0 mm × 20.0 mm) and a Pt plate cathode (0.1 mm × 10.0 mm × 20.0 mm). To a 15 mL oven-dried undivided electrochemical cell equipped with a magnetic bar were added primary unactivated alkenes (0.3 mmol, 1.0 equiv.), $Et_4NI$ (154.2 mg, 0.6 mmol, 2.0 equiv.), $NH_4I$ (86.9 mg, 0.6 mmol, 2.0 equiv.) and TFA (67.0 µL, 0.9 mmol, 3.0 equiv.). Then *ᵗ*BuOH (3 mL) and $H_2O$ (1 mL) were added under air. The electrocatalysis was performed at 50 °C with a constant current of 50 mA maintained for 12 h. The electrodes were washed with EtOAc (3 × 5 mL) in an ultrasonic bath. Then the solution of $Na_2S_2O_3$ (10 mL) was added to the system, and the resulting mixture was extracted with EtOAc (2 × 20 mL). The combined organic phase was dried with anhydrous $Na_2SO_4$, filtered, and concentrated in vacuo. The crude product was purified by column chromatography to furnish the desired product.

## General Procedure for Electrochemical Epoxidation of Unactivated Alkenes.

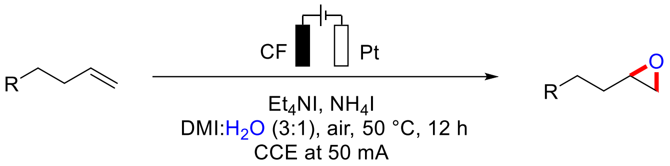


The electrocatalysis was carried out in an undivided cell with a carbon felt (CF) anode (5.0 mm × 10.0 mm × 20.0 mm) and a Pt plate cathode (0.1 mm × 10.0 mm × 20.0 mm). To a 15 mL oven-dried undivided electrochemical cell equipped with a magnetic bar were added primary unactivated alkenes (0.3 mmol, 1.0 equiv.), $Et_4NI$ (154.2 mg, 0.6 mmol, 2.0 equiv.), and $NH_4I$ (86.9 mg, 0.6 mmol, 2.0 equiv.). Then DMI (3 mL) and $H_2O$ (1 mL) were added under air. The electrocatalysis was performed at 50 °C with a constant current of 50 mA maintained for 12 h. The electrodes were washed with EtOAc (3 × 5 mL) in an ultrasonic bath. $H_2O$ (20 mL) was added to the system, and the resulting mixture was extracted with EtOAc (2 × 50 mL). The combined organic phase was dried with anhydrous $Na_2SO_4$, filtered, and concentrated in vacuo. The crude product was purified by column chromatography to furnish the desired product.

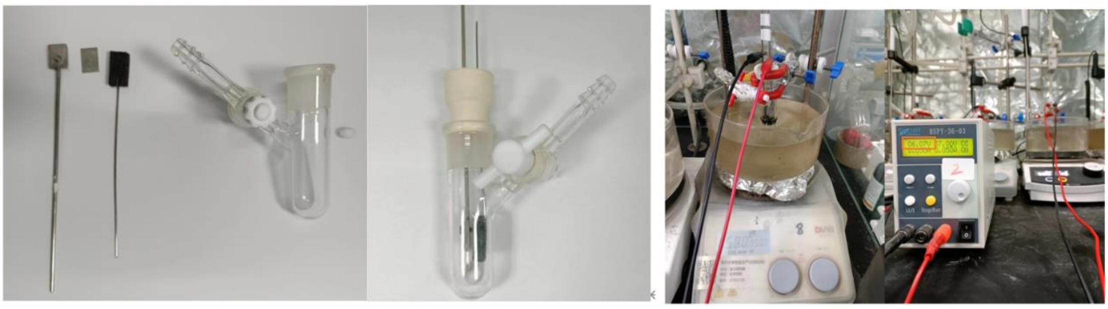

**Supplementary Figure 3**. Electrolysis set-up

## Gram-Scale Reaction

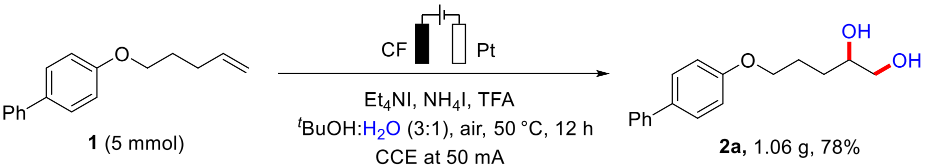


**1** (5 mmol) **2a**, 1.06 g, 78%

The electrocatalysis was carried out in an undivided cell with a carbon felt (CF) anode (5.0 mm × 25.0 mm × 50.0 mm) and a Pt plate cathode (0.25 mm × 25.0 mm × 50.0 mm). To an oven-dried undivided electrochemical cell (diameter: 40 mm; length: 130 mm; volume: 200 mL) equipped with a magnetic bar were added primary unactivated alkenes. (5.0 mmol, 1.0 equiv.), $Et_4NI$ (2.57 g, 10.0 mmol, 2.0 equiv.), $NH_4I$ (1.45 g, 10.0 mmol, 2.0 equiv.) and TFA (1.12 mL, 15.0 mmol, 3.0 equiv.). Then $^t$BuOH (60 mL) and $H_2O$ (20 mL) were added under air. The electrocatalysis was performed at 50 °C with a constant current of 50 mA maintained for 12 h. The electrodes were washed with EtOAc (3 × 20 mL) in an ultrasonic bath. Then the solution of $Na_2S_2O_3$ (50 mL) was added to the system, and the resulting mixture was extracted with EtOAc (2 × 100 mL).The combined organic phase was dried with anhydrous $Na_2SO_4$, filtered, and concentrated in vacuo. The product was purified by column chromatography to provide 1.06 g (78%) of compound **2a**.

## Application of Vicinal Diols

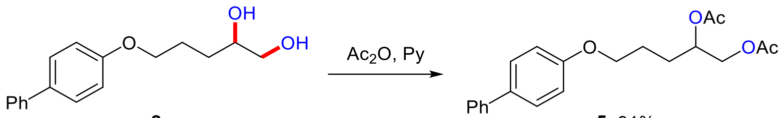


**2a** **5**, 91%

A mixture of the **2a** (1.0 mmol), acetic anhydride (1.0 M), and pyridine (10 mmol%) was stirred at room temperature. After completion of the reaction (TLC), the reaction mixture was triturated with EtOAc (15 mL) and the reagent was filtered. The organic layer was washed with saturated $NaHCO_3$ and water (3×15 mL), dried over anhydrous $MgSO_4$ and filtered. Evaporation of the solvent under reduced pressure and purified by column chromatography gave the requested product **5** in 91% isolated yield.

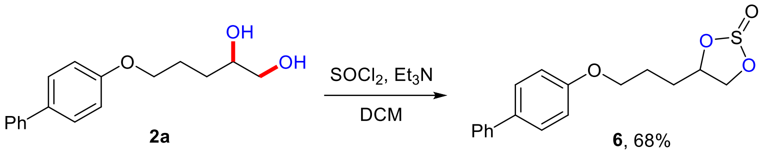


**2a** **6**, 68%

To a solution of diol **2a** (136 mg, 0.5 mmol) and $NEt_3$ (1.7 mL, 2.4 equiv.) in DCM (15 mL) cooled in an ice-bath was added dropwise $SOCl_2$ (0.45 mL, 1.2 equiv.). The resulting solution was stirred for 30 minutes over the ice bath. The reaction mixtur e was diluted with water (20 mL) and organic layers were separated. Aqueous layer was further extracted with DCM (2 × 15 mL). Combined organics were sequentially washed with IN HCl (10 mL), saturated $NaHCO_3$ (10 mL), brine (10 mL), dried over $NaSO_4$, filtered and concentrated in vacuo to give the crude intermediate sulfite. The crude product was purified by column chromatography to furnish the desired product **6** in 68%.

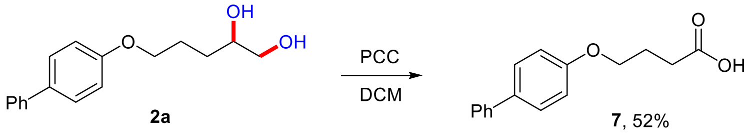


**2a** **7**, 52%

To a 50 mL flask was added **2a** (136 mg, 0.5 mmol), PCC (215.6 mg, 1.0 mmol) and DCM (0.2 M). The mixture was stirred overnight at room temperature. The reaction mixture was diluted with $Et_2O$, filtered through a short pad of silica gel, concentrated under reduced pressure and purified through column chromatography using PE/EA (20:1) to afford **7** (66.4 mg, 52%) as a white solid.

## Mechanistic Experiments

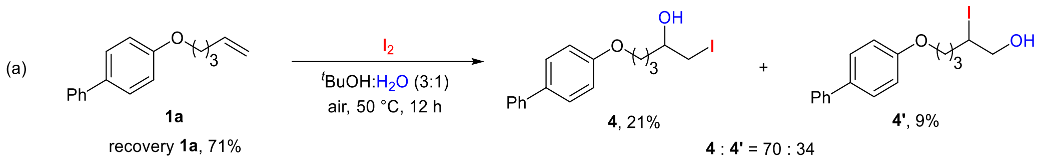


Add **1a** (0.2 mmol, 1.0 equiv.) and $I_2$ (101.5 mg, 0.4 mmol, 4.0 equiv.) to a 15 mL reaction tube. Then ${}^{t}$BuOH (3 mL) and $H_2O$ (1 mL) were added under air. The system was performed at 50 °C for 12 h. Then the solution of $Na_2S_2O_3$ (10 mL) was added to the system, and the resulting mixture was extracted with EtOAc (2 × 20 mL). The combined organic phase was dried with anhydrous $Na_2SO_4$, filtered, and concentrated in vacuo. The crude product was purified by column chromatography to furnish the desired product **4** and **4'** (70:34).

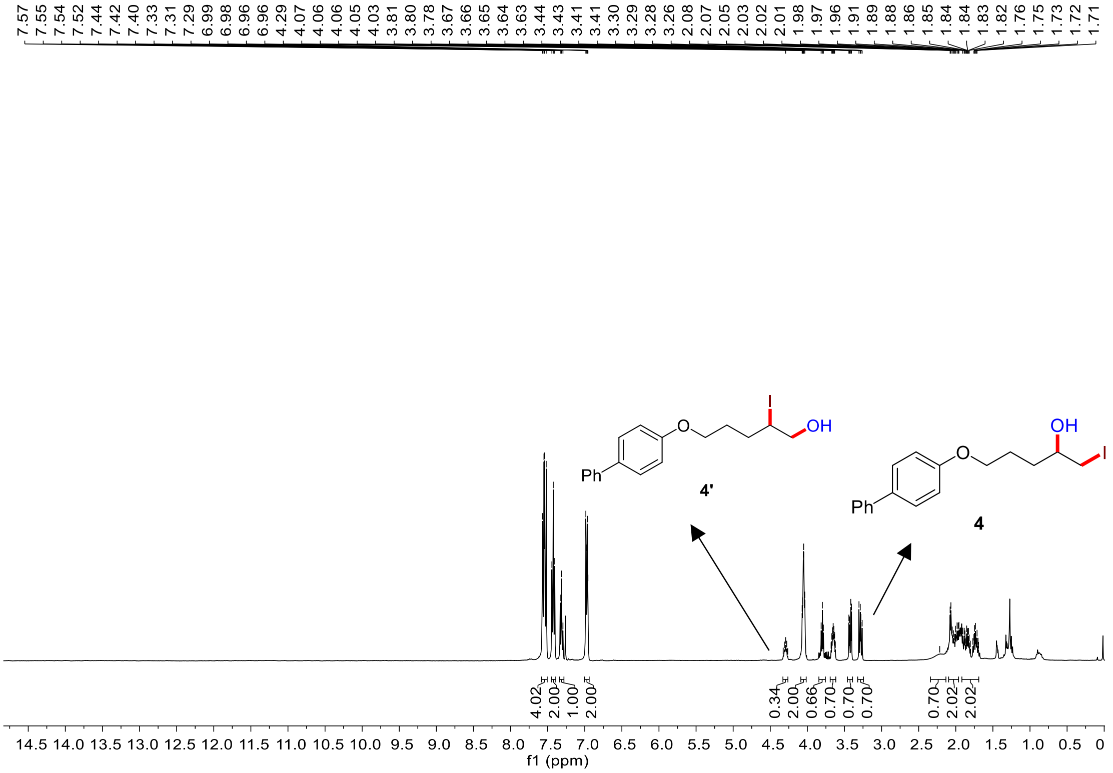


**Supplementary Figure 4**. $^{1}$H NMR (400MHz, Chloroform-*d*)

## Synthesis of 4

(b) 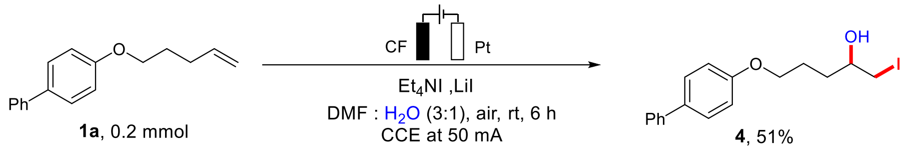

The electrocatalysis was carried out in an undivided cell with a carbon felt (CF) anode (5.0 mm × 10.0 mm × 20.0 mm) and a Pt plate cathode (0.1 mm × 10.0 mm × 20.0 mm). To a 15 mL oven-dried undivided electrochemical cell equipped with a magnetic bar were added primary unactivated alkenes. (0.2 mmol, 1.0 equiv.), $Et_4NI$ (25.7 mg, 0.1 mmol, 0.5 equiv.), and LiI (13.3 mg, 0.1 mmol, 0.5 equiv.). Then DMF (3 mL) and $H_2O$ (1 mL) were added under air. The electrocatalysis was performed at room temperature with a constant current of 50 mA maintained for 6 h. The electrodes were washed with EtOAc (3 × 5 mL) in an ultrasonic bath. $H_2O$ (20 mL) was added to the system, and the resulting mixture was extracted with EtOAc (2 × 50 mL). The combined organic phase was dried with anhydrous $Na_2SO_4$, filtered, and concentrated in vacuo. The crude product was purified by column chromatography to furnish the desired product **4**.

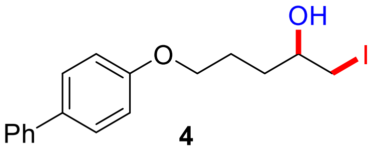
**4**

**5-([1,1'-Biphenyl]-4-yloxy)-1-iodopentan-2-ol**

Compound **4** was prepared following the general procedure, purification by column chromatography on silica gel (petroleum ether/ethyl acetate = 10:1) yielded **4** (39.0 mg, 51%) as a white solid. M.p.: 62–63 °C. $^1$H NMR (400 MHz, Chloroform-*d*) δ 7.58 – 7.49 (m, 4H), 7.41 (t, J = 7.7 Hz, 2H), 7.30 (t, J = 7.3 Hz, 1H), 6.99 – 6.94 (m, 2H), 4.07 – 4,04 (m, 2H), 3.68 – 3.62 (m, 1H), 3.43 (dd, J = 10.2, 3.7 Hz, 1H), 3.28 (dd, J = 10.2, 6.8 Hz, 1H), 2.21 (d, J = 13.2 Hz, 1H), 2.03 – 1.92 (m, 2H), 1.88 – 1.82 (m, 1H), 1.78 – 1.71 (m, 1H). $^{13}$C NMR (100 MHz, Chloroform-*d*) δ 158.3, 140.7, 133.8, 128.7, 128.1, 126.7, 126.6, 114.7, 70.7, 67.6, 33.3, 25.6, 16.1.

**Isotope labeling experiments**

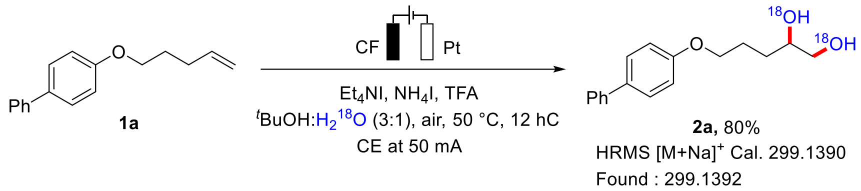


The electrocatalysis was carried out in an undivided cell with a carbon felt (CF) anode (5.0 mm × 10.0 mm × 20.0 mm) and a Pt plate cathode (0.1 mm × 10.0 mm × 20.0 mm). To a 15 mL oven-dried undivided electrochemical cell equipped with a magnetic bar were added primary unactivated alkenes. (0.3 mmol, 1.0 equiv.), $Et_4NI$ (154.2 mg, 0.6 mmol, 2.0 equiv.), $NH_4I$ (86.9 mg, 0.6 mmol, 2.0 equiv.) and TFA (67.0 µL, 0.9 mmol, 3.0 equiv.). Then $^t$BuOH (3 mL) and $H_2{}^{18}O$ (1 mL) were added under air. The electrocatalysis was performed at 50 °C with a constant current of 50 mA maintained for 12 h. The electrodes were washed with EtOAc (3 × 5 mL) in an ultrasonic bath. Then the solution of $Na_2S_2O_3$ (10 mL) was added to the system, and the resulting mixture was extracted with EtOAc (2 × 20 mL). The combined organic phase was dried with anhydrous $Na_2SO_4$, filtered, and concentrated in vacuo. The crude product was purified by column chromatography to furnish the desired product.

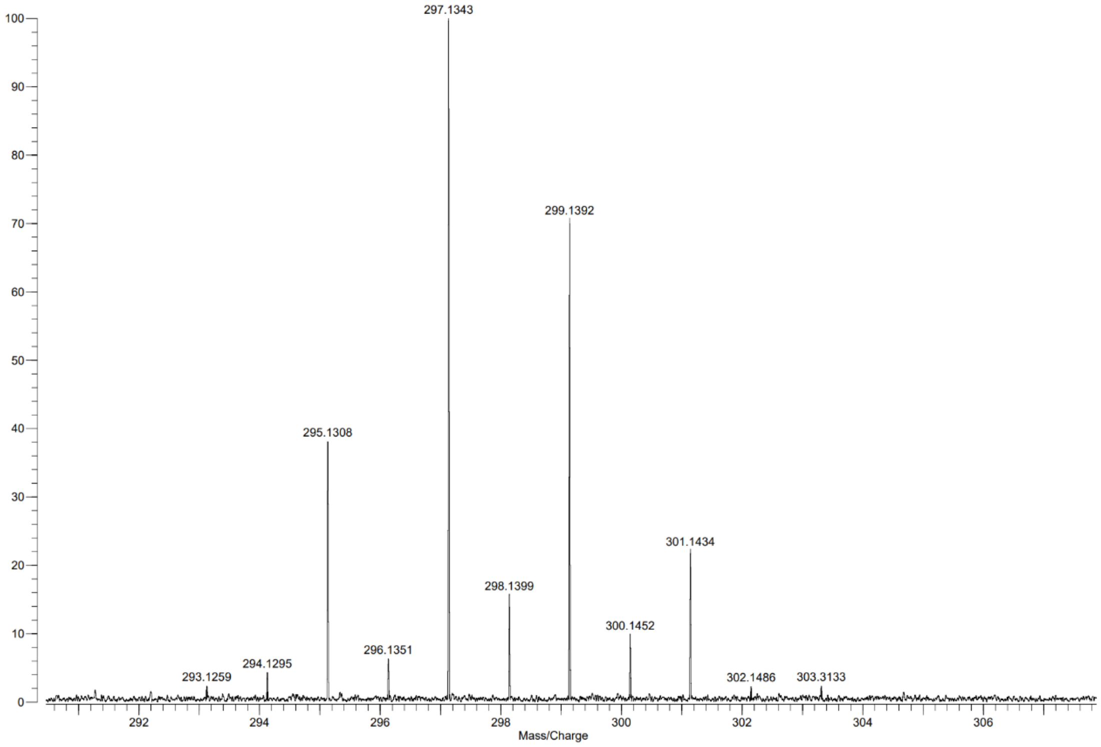


**Supplementary Figure 5**. The HRMS of **2a** ($^{18}$O)

## Control experiment of tertiary ammonium

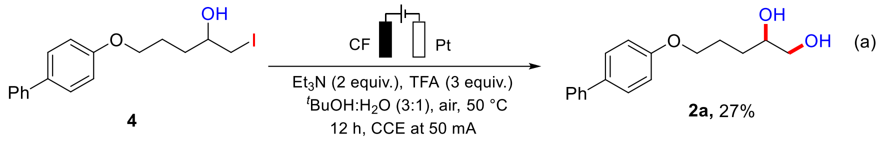


The electrocatalysis was carried out in an undivided cell with a carbon felt (CF) anode (5.0 mm × 10.0 mm × 20.0 mm) and a Pt plate cathode (0.1 mm × 10.0 mm × 20.0 mm). To a 15 mL oven-dried undivided electrochemical cell equipped with a magnetic bar were added primary **4** (0.3 mmol, 1.0 equiv.), $Et_3N$ (55.6 µL, 0.4 mmol, 2 equiv.) and TFA (67.0 µL, 0.9 mmol, 3.0 equiv.). Then $^t$BuOH (3 mL) and $H_2O$ (1 mL) were added under air. The electrocatalysis was performed at 50 °C with a constant current of 50 mA maintained for 12 h. The electrodes were washed with EtOAc (3 × 5 mL) in an ultrasonic bath. Then the solution of $Na_2S_2O_3$ (10 mL) was added to the system, and the resulting mixture was extracted with EtOAc (2 × 20 mL). The combined organic phase was dried with anhydrous $Na_2SO_4$, filtered, and concentrated in vacuo. The crude product was purified by column chromatography to furnish the desired product.

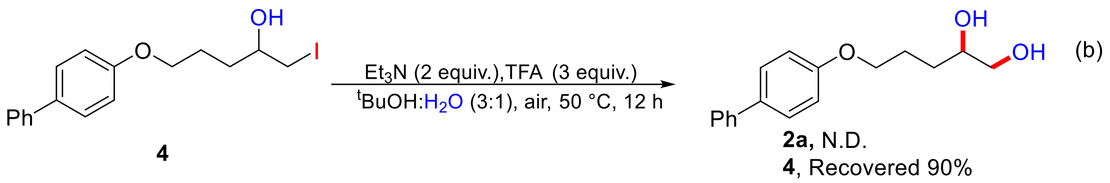


To a 15 mL oven-dried undivided electrochemical cell equipped with a magnetic bar were added primary **4** (0.3 mmol, 1.0 equiv.), $Et_3N$ (55.6 µL, 0.4 mmol, 2 equiv.) and TFA (67.0 µL, 0.9 mmol, 3.0 equiv.). Then $^t$BuOH (3 mL) and $H_2O$ (1 mL) were added under air. The electrocatalysis was performed at 50 °C maintained for 12 h.

## Cyclic Voltammetry

The cyclic voltammetry was carried out with a Shanghai Chenhua CHI760E workstation. A glassy-carbon (GC) electrode (5 mm-diameter, disk-electrode) was used as the working electrode, Pt wire was used as the counter electrode and an Ag wire electrode was used as the reference electrode. The measurements were carried out at a scan rate of 50 mV $s^{-1}$ in MeCN/$Et_4NBF_4$ (0.1 M) under oxidation conditions. The

operation temperature was 298 K.

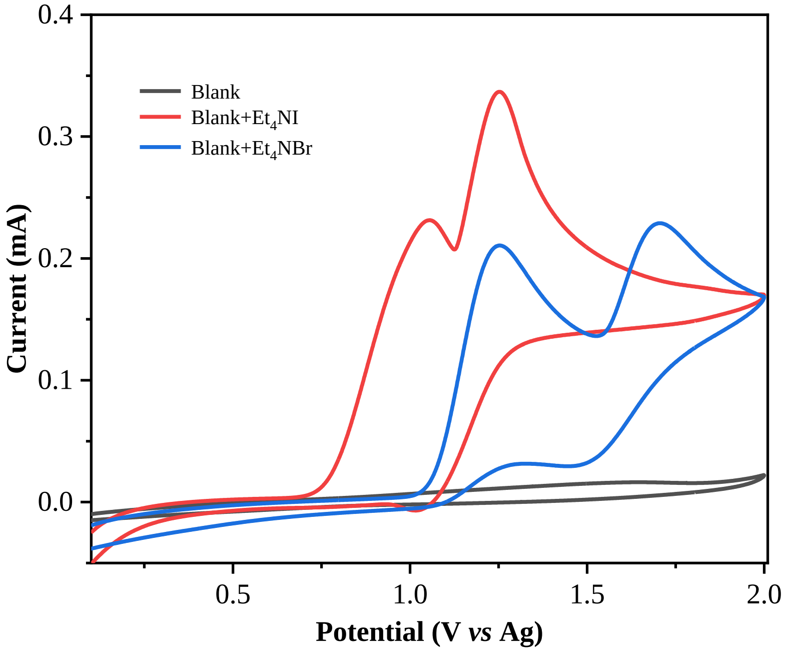


**Supplementary Figure 6**. Cyclic voltammograms of Et4NI and Et4NBr (10 mM) at 50.0 mVs$^{-1}$ in MeCN (10.0 mL). Et4NBF4 (0.1 M in MeCN).

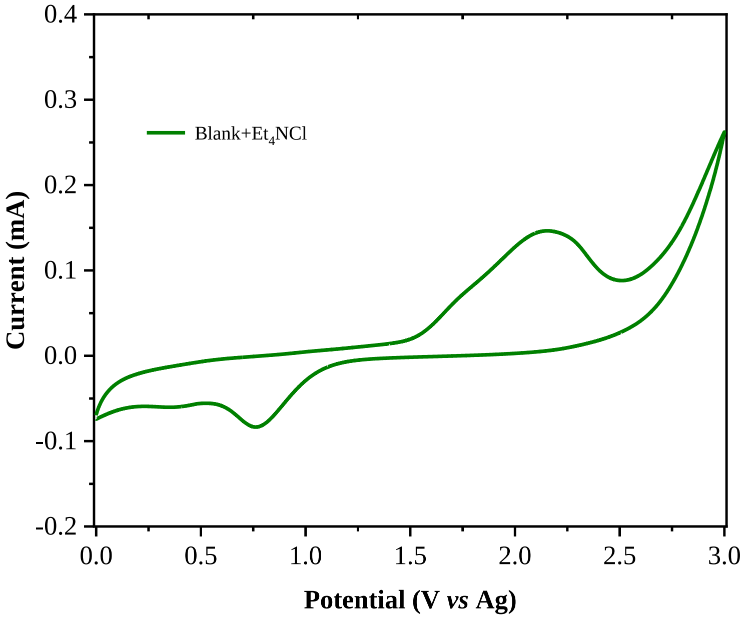


**Supplementary Figure 7**. Cyclic voltammetry of Et4NCl (green line, 10.0 mM), at 50.0 mVs$^{-1}$ in MeCN (10 mL). Et4NBF4 (0.1 M in MeCN).

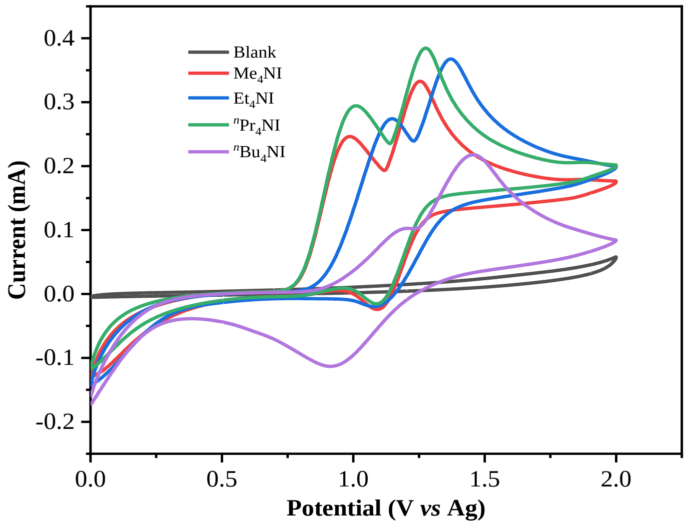


**Supplementary Figure 8**. Cyclic voltammetry of $Me_4NI$ (red line, 10.0 mM), $Et_4NI$ (blue line, 10.0 mM), ${}^nPr_4NI$ (green line, 10.0 mM), ${}^nBu_4NI$ (purple line, 10.0 mM), at 50.0 $mVs^{-1}$ in MeCN (10 mL). $Et_4NBF_4$ (0.1 M in MeCN).

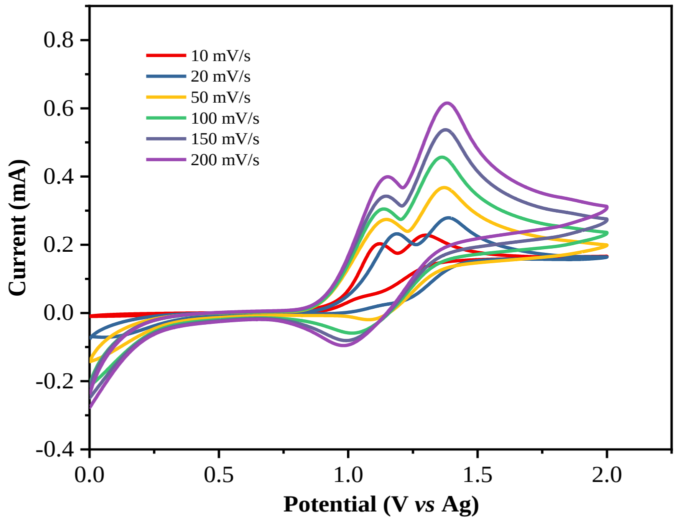


**Supplementary Figure 9**. Cyclic voltammetry of $Et_4NI$ (10.0 mM), at different scanning speeds in MeCN (10 mL). $Et_4NBF_4$ (0.1 M in MeCN).

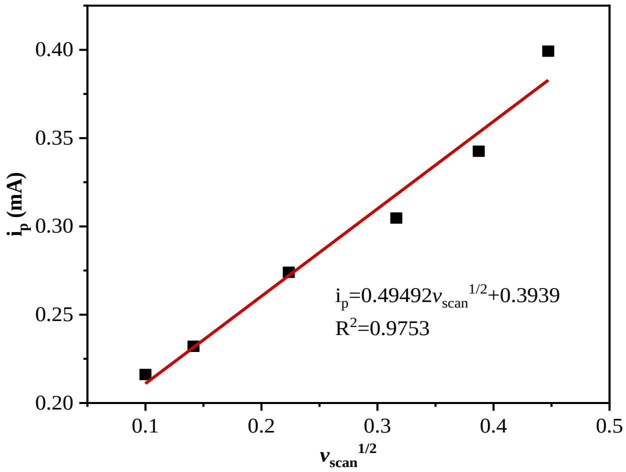

**Supplementary Figure 10**. Linear fit analysis of $v_{scan}^{1/2}$ and $i_p$ for the first oxidation peak of $Et_4NI$.

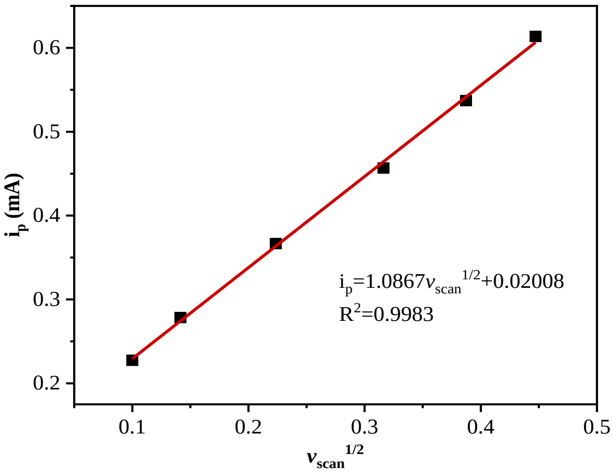


**Supplementary Figure 11**. Linear fit analysis of $v_{scan}^{1/2}$ and $i_p$ for the second oxidation peak of $Et_4NI$.

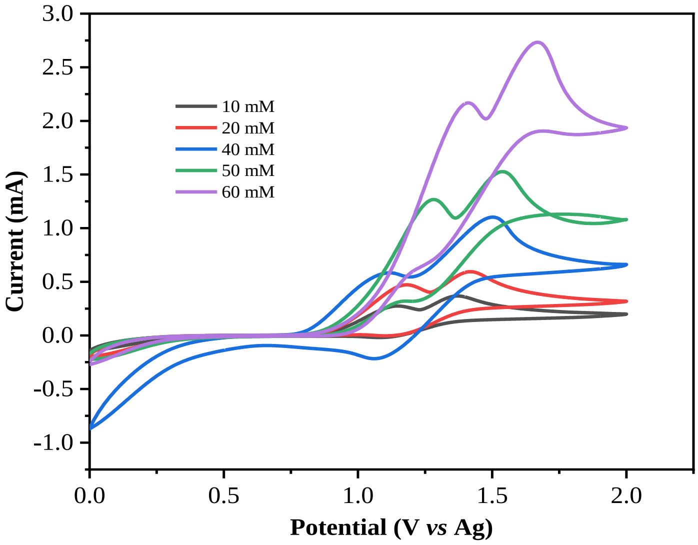


**Supplementary Figure 12**. Cyclic voltammetry of $Et_4NI$ performed in the presence of increasing 60.0 mM, at 50.0 $mVs^{-1}$ in MeCN (10 mL). $Et_4NBF_4$ (0.1 M in MeCN).

## Characterization Data of Products

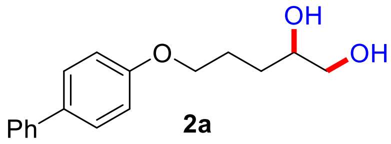

**2a**

**5-([1,1'-Biphenyl]-4-yloxy)pentane-1,2-diol**

Compound **2a** was prepared following the general procedure, purification by column chromatography on silica gel (DCM/MeOH = 50:1) yielded **2a** (65.3 mg, 80%) as a white solid. M.p.: 67–68 °C. $^{1}$H NMR (400 MHz, DMSO-$d_6$) δ 7.61 – 7.57 (m, 4H), 7.44 – 7.40 (m, 2H), 7.30 (t, J = 7.6 Hz, 1H), 7.02 – 6.99 (m, 2H), 4.50 (t, J = 4.4 Hz, 2H), 4.01 (t, J = 6.4 Hz, 2H), 3.49 – 3.45 (m, 1H), 3.32 – 3.25 (m, 2H), 1.90 – 1.84 (m, 1H), 1.78 – 1.70 (m, 1H), 1.66 – 1.58 (m, 1H), 1.41 – 1.31 (m, 1H). $^{13}$C NMR (100 MHz, DMSO-$d_6$) δ 158.4, 140.0, 132.4, 128.9, 127.8, 126.7, 126.2, 115.0, 70.9, 67.9, 66.1, 30.0, 25.2. HR-MS (ESI) m/z calc. for $C_{17}H_{20}O_3$ [M+H]$^+$: 273.1485, found: 273.1488.

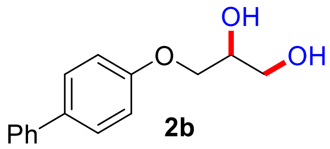

**2b**

**3-([1,1'-Biphenyl]-4-yloxy)propane-1,2-diol**

Compound **2b** was prepared following the general procedure, purification by column chromatography on silica gel (DCM/MeOH = 50:1) yielded **2b** (38.1 mg, 52%) as a white solid. M.p.: 62–63 °C. $^{1}$H NMR (400 MHz, DMSO-$d_6$) δ 7.62 – 7.58 (m, 4H), 7.45 – 7.41 (m, 2H), 7.30 (t, J = 7.2 Hz, 1H), 7.05 – 7.01 (m, 2H), 4.96 (d, J = 5.2 Hz, 1H), 4.68 (t, J = 5.6 Hz, 1H), 4.05 (dd, J = 9.6, 4.0 Hz, 1H), 3.91 (dd, J = 10.0, 6.4 Hz, 1H), 3.84 – 3.80 (m, 1H), 3.47 (t, J = 5.6 Hz, 2H). $^{13}$C NMR (100 MHz, DMSO-$d_6$) δ 158.4, 139.8, 132.4, 128.8, 127.7, 126.6, 126.1, 114.9, 69.9, 69.7, 62.7. HR-MS (ESI) m/z calc. for $C_{15}H_{17}O_3$ [M+H]$^+$: 245.1172, found: 245.1171.

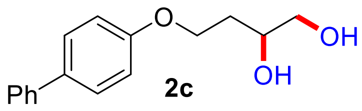

**2c**

**4-([1,1'-Biphenyl]-4-yloxy)butane-1,2-diol**

Compound **2c** was prepared following the general procedure, purification by column chromatography on silica gel (DCM/MeOH = 50:1) yielded **2c** (58.3 mg, 75%) as a white solid. M.p.: 65–66 °C. $^{1}$H NMR (400 MHz, DMSO-*d*$_6$) δ 7.61 – 7.58 (m, 4H), 7.44 – 7.40 (m, 2H), 7.30 (t, J = 7.6 Hz, 1H), 7.02 – 7.00 (m, 2H), 4.62 (d, J = 5.2 Hz, 1H), 4.56 (t, J = 5.6 Hz, 1H), 4.11 (t, J = 6.8 Hz, 2H), 3.68 – 3.63 (m, 1H), 3.40 – 3.33 (m, 2H), 1.98 – 1.92 (m, 1H), 1.70 – 1.61 (m, 1H). $^{13}$C NMR (100 MHz, DMSO-*d*$_6$) δ 158.3, 139.8, 132.3, 128.8, 127.7, 126.6, 126.1, 114.8, 68.0, 66.0, 64.6, 33.1. HR-MS (ESI) m/z calc. for $C_{16}H_{19}O_3$ $[M+H]^+$: 259.1329, found: 259.1333.

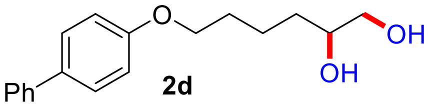


**2d**

**6-([1,1'-Biphenyl]-4-yloxy)hexane-1,2-diol**

Compound **2d** was prepared following the general procedure, purification by column chromatography on silica gel (DCM/MeOH = 50:1) yielded **2d** (59.3 mg, 69%) as a white solid. M.p.: 68–69 °C. $^{1}$H NMR (400 MHz, DMSO-*d*$_6$) δ 7.61 – 7.57 (m, 4H), 7.44 – 7.41 (m, 2H), 7.32 – 7.28 (m, 1H), 7.01 (d, J = 6.8 Hz, 2H), 4.43 (s br, 2H), 4.00 (t, J = 6.4 Hz, 2H), 3.44 – 3.41 (m, 1H), 3.32 – 3.24 (m, 2H), 1.77 – 1.69 (m, 2H), 1.58 – 1.42 (m, 3H), 1.32 – 1.26 (m, 1H). $^{13}$C NMR (100 MHz, DMSO-*d*$_6$) δ 158.3, 139.8, 132.3, 128.8, 127.7, 126.6, 126.1, 114.8, 71.0, 67.5, 66.0, 33.1, 28.9, 21.8. HR-MS (ESI) m/z calc. for $C_{18}H_{23}O_3$ $[M+H]^+$: 287.1642, found: 287.1639.

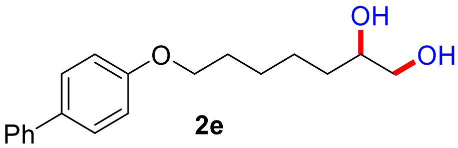


**2e**

**7-([1,1'-Biphenyl]-4-yloxy)heptane-1,2-diol**

Compound **2e** was prepared following the general procedure, purification by column chromatography on silica gel (DCM/MeOH = 50:1) yielded **2e** (68.6 mg, 76%) as a white solid. M.p.: 67–68 °C. $^{1}$H NMR (400 MHz, DMSO-*d*$_6$) δ 7.61 – 7.56 (m, 4H), 7.44 – 7.40 (m, 2H), 7.30 (t, J = 7.2 Hz, 1H), 7.01 – 6.99 (m, 2H), 4.44 (t, J = 5.6 Hz, 1H), 4.37 (d, J = 4.8 Hz, 1H), 3.98 (t, J = 6.4 Hz, 2H), 3.45 – 3.39 (m, 1H), 3.30 – 3.23 (m, 2H), 1.74 – 1.71 (m, 2H), 1.47 – 1.39 (m, 4H), 1.34 – 1.22 (m, 2H). $^{13}$C NMR (100 MHz, DMSO-*d*$_6$) δ 158.4, 139.9, 132.4, 128.9, 127.8, 126.7, 126.2, 114.9, 71.1, 67.6,

66.1, 33.5, 28.9, 25.8, 25.0. HR-MS (ESI) m/z calc. for $C_{19}H_{25}O_3$ $[M+H]^+$: 301.1798, found: 301.1801.

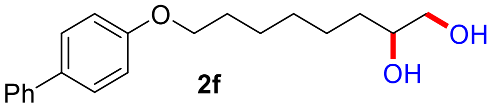
**2f**

**8-([1,1'-Biphenyl]-4-yloxy)octane-1,2-diol**

Compound **2f** was prepared following the general procedure, purification by column chromatography on silica gel (DCM/MeOH = 50:1) yielded **2f** (68.2 mg, 72%) as a white solid. M.p.: 69–70 °C. $^1$H NMR (400 MHz, DMSO-$d_6$) δ 7.61 – 7.56 (m, 4H), 7.44 – 7.40 (m, 2H), 7.30 (t, J = 7.2 Hz, 1H), 7.01 – 6.99 (m, 2H), 4.44 (t, J = 5.6 Hz, 1H), 4.36 (d, J = 4.8 Hz, 1H), 3.98 (t, J = 6.4 Hz, 2H), 3.42 – 3.39 (m, 1H), 3.31 – 3.22 (m, 2H), 1.76 – 1.69 (m, 2H), 1.45 – 1.41 (m, 5H), 1.34 – 1.29 (m, 3H). $^{13}$C NMR (100 MHz, DMSO-$d_6$) δ 158.3, 139.8, 132.3, 128.8, 127.7, 126.6, 126.1, 114.8, 71.1, 67.5, 66.0, 33.4, 29.0, 28.7, 25.6, 25.1. HR-MS (ESI) m/z calc. for $C_{20}H_{27}O_3$ $[M+H]^+$: 315.1955, found: 315.1956.

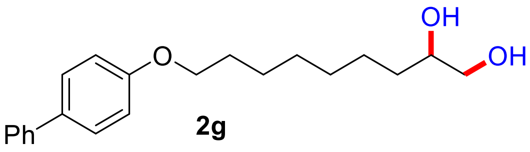
**2g**

**9-([1,1'-Biphenyl]-4-yloxy)nonane-1,2-diol**

Compound **2g** was prepared following the general procedure, purification by column chromatography on silica gel (DCM/MeOH = 50:1) yielded **2g** (69.8 mg, 71%) as a white solid. M.p.: 69–70 °C. $^1$H NMR (400 MHz, DMSO-$d_6$) δ 7.61 – 7.56 (m, 4H), 7.44 – 7.40 (m, 2H) 7.30 (t, J = 7.4 Hz, 1H), 7.01 – 7.00 (m, 2H), 4.41 (t, J = 5.6 Hz, 1H), 4.32 (d, J = 4.8 Hz, 1H), 3.99 (t, J = 6.4 Hz, 2H), 3.41 – 3.38 (m, 1H), 3.30 – 3.20 (m, 2H), 1.75 – 1.69 (m, 2H), 1.42 – 1.22 (m, 10H). $^{13}$C NMR (100 MHz, DMSO-$d_6$) δ 158.3, 139.8, 132.3, 128.8, 127.7, 126.6, 126.1, 114.8, 71.1, 67.5, 66.0, 33.4, 29.2, 28.8, 28.7, 25.5, 25.1. HR-MS (ESI) m/z calc. for $C_{21}H_{29}O_3$ $[M+H]^+$: 329.2111, found: 329.2106.

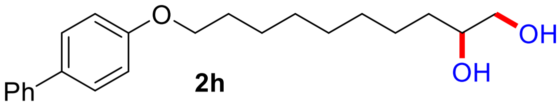
**2h**

**10-([1,1'-Biphenyl]-4-yloxy)decane-1,2-diol**

Compound **2h** was prepared following the general procedure, purification by column chromatography on silica gel (DCM/MeOH = 50:1) yielded **2h** (66.9 mg, 65%) as a white solid. M.p.: 73–75 °C. $^{1}$H NMR (400 MHz, DMSO-$d_6$) δ 7.61 – 7.56 (m, 4H), 7.44 – 7.40 (m, 2H), 7.31 – 7.28 (m, 1H), 7.01 – 6.99 (m, 2H), 4.41 (t, J = 4.8 Hz, 1H), 4.33 (d, J = 4.0 Hz, 1H), 3.98 (t, J = 6.0 Hz, 2H), 3.37 (s, 1H), 3.26 – 3.22 (m, 2H), 1.73 – 1.70 (m, 2H), 1.40 – 1.26 (m, 12H). $^{13}$C NMR (100 MHz, DMSO-$d_6$) δ 158.3, 139.8, 132.3, 128.8, 127.7, 126.6, 126.1, 114.8, 71.1, 67.5, 66.0, 33.4, 29.2, 29.1, 28.8, 28.7, 25.5, 25.2. HR-MS (ESI) m/z calc. for $C_{22}H_{31}O_3$ $[M+H]^+$: 343.2268, found: 343.2269.

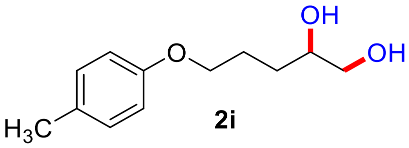


**2i**

**5-(*p*-Tolyloxy)pentane-1,2-diol**

Compound **2i** was prepared following the general procedure, purification by column chromatography on silica gel (DCM/MeOH = 50:1) yielded **2i** (49.3 mg, 78%) as a colorless oil $^{1}$H NMR (400 MHz, Chloroform-*d*) δ 7.08 – 7.06 (m, 2H), 6.80 – 6.78 (m, 2H), 4.00 – 3.96 (m, 2H), 3.83 – 3.77 (m, 1H), 3.68 (dd, J = 10.8, 3.2 Hz, 1H), 3.48 (dd, J = 11.2, 7.6 Hz, 1H), 2.28 (s, 3H), 2.15 – 2.12 (m, 2H), 1.99 – 1.86 (m, 2H), 1.68 – 1.58 (m, 2H). $^{13}$C NMR (100 MHz, Chloroform-*d*) δ 156.6, 130.1, 129. 9, 114.3, 71.9, 67.9, 66.8, 30.1, 25.5, 20.4. HR-MS (ESI) m/z calc. for $C_{12}H_{19}O_3$ $[M+H]^+$: 211.1329, found: 211.1334.

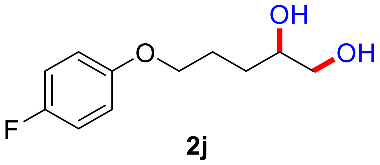


**2j**

**5-(4-Fluorophenoxy)pentane-1,2-diol**

Compound **2j** was prepared following the general procedure, purification by column chromatography on silica gel (DCM/MeOH = 50:1) yielded **2j** (51.4 mg, 80%) as a colorless oil. $^{1}$H NMR (400 MHz, Chloroform-*d*) δ 6.98 – 6.94 (m, 2H), 6.84 – 6.81 (m, 2H), 4.00 – 3.92 (m, 2H), 3.82 – 3.77 (m, 1H), 3.68 (dd, J = 11.2, 3.2 Hz, 1H), 3.48 (dd, J = 11.2, 7.6 Hz, 1H), 2.23 (s br, 2H), 1.99 – 1.83 (m, 2H), 1.71 – 1.57 (m, 2H). $^{13}$C NMR (100 MHz, Chloroform-*d*) δ 157.2 (d, $J_{C–F}$ = 237.0 Hz), 154.9 (d, $J_{C–F}$ = 2.0 Hz),

115.8 (d, $J_{C-F}$ = 23.0 Hz), 115.4 (d, $J_{C-F}$ = 8.0 Hz), 71.9, 68.4, 66.7, 29.8, 25.5. $^{19}$F NMR (375 MHz, Chloroform-*d*) δ -123.96. HR-MS (ESI) m/z calc. for $C_{11}H_{15}FO_3Na$ $[M+Na]^+$: 237.0897, found: 237.0899.

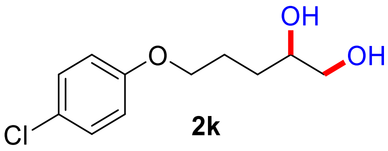

**2k**

**5-(4-Chlorophenoxy)pentane-1,2-diol**

Compound **2k** was prepared following the general procedure, purification by column chromatography on silica gel (DCM/MeOH = 50:1) yielded **2k** (55.1 mg, 80%) as a colorless oil.$^1$H NMR (400 MHz, Chloroform-*d*) δ 7.22 – 7.20 (m, 2H), 6.81 – 6.78 (m, 2H), 3.93 (t, J = 6.0 Hz, 2H), 3.85 (s, 1H), 3.73 (d, J = 10.0 Hz, 1H), 3.53 – 3.49 (m, 1H), 3.33 (s br, 2H), 1.96 – 1.82 (m, 2H), 1.64 – 1.60 (m, 2H). $^{13}$C NMR (100 MHz, Chloroform-*d*) δ 157.3, 129.3, 125.7, 115.7, 71.9, 68.1, 66.4, 29.8, 25.3. HR-MS (ESI) m/z calc. for $C_{11}H_{15}ClO_3Na$ $[M+Na]^+$: 253.0602, found: 253.0603.

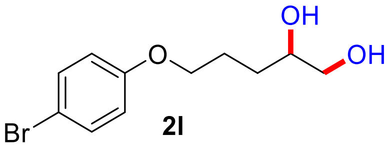

**2l**

**5-(4-Bromophenoxy)pentane-1,2-diol**

Compound **2l** was prepared following the general procedure, purification by column chromatography on silica gel (DCM/MeOH = 50:1) yielded **2l** (33.8 mg, 41%) as a white solid. M.p.: 75–76 °C. $^1$H NMR (400 MHz, Chloroform-*d*) δ 7.37 – 7.34 (m, 2H), 6.77 – 6.75 (m, 2H), 3.97 – 3.94 (m, 2H), 3.78 (s, 1H), 3.68 (d, J = 11.2 Hz, 1H), 3.50 – 3.45 (m, 1H), 2.51 (s br, 2H), 1.97 – 1.84 (m, 2H), 1.68 – 1.56 (m, 2H). $^{13}$C NMR (100 MHz, Chloroform-*d*) δ 157.9, 132.2, 116.3, 112.9, 71.9, 68.0, 66.7, 29.7, 25.4. HR-MS (ESI) m/z calc. for $C_{11}H_{16}BrO_3$ $[M+H]^+$: 275.0277, found: 275.0279.

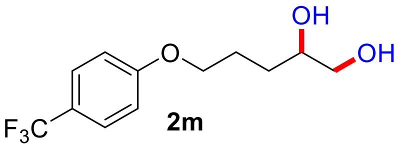

**2m**

**5-(4-(Trifluoromethyl)phenoxy)pentane-1,2-diol**

Compound **2m** was prepared following the general procedure, purification by column chromatography on silica gel (DCM/MeOH = 50:1) yielded **2m** (42.8 mg, 54%) as a

white soild. M.p.: 73–74 °C. $^{1}$H NMR (400 MHz, Chloroform-*d*) δ 7.52 – 7.50 (m, 2H), 6.93 – 6.91 (m, 2H), 4.03 – 3.99 (m, 2H), 3.80 – 3.75 (m, 1H), 3.68 (dd, J = 11.2, 2.8 Hz, 1H), 3.49 – 3.31 (m, 3H), 2.02 – 1.93 (m, 1H), 1.90 – 1.83 (m, 1H), 1.67 – 1.55 (m, 2H). $^{13}$C NMR (100 MHz, Chloroform-*d*) 161.3, 126.9 (q, $J_{C-F}$ = 3.8 Hz), 124.4 (q, $J_{C-F}$ = 270.5 Hz), 122.9 (q, $J_{C-F}$ = 33.5 Hz), 114.3, 71.9, 68.0, 66.7, 29.6, 25.3. $^{19}$F NMR (375 MHz, Chloroform-*d*) δ -61.47. HR-MS (ESI) m/z calc. for $C_{12}H_{16}F_3O_3$ $[M+H]^+$: 265.1046, found: 265.1043.

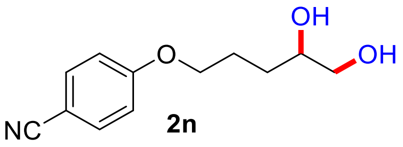

**2n**

**4-((4,5-Dihydroxypentyl)oxy)benzonitrile**

Compound **2n** was prepared following the general procedure, purification by column chromatography on silica gel (DCM/MeOH = 50:1) yielded **2n** (41.9 mg, 63%) as a white solid. M.p.: 76–77 °C. $^{1}$H NMR (400 MHz, DMSO-$d_6$) δ 7.76 – 7.73 (m, 2H), 7.10 – 7.07 (m, 2H), 4.48 (s br, 2H), 4.06 (t, J = 6.8 Hz, 2H), 3.47 – 3.42 (m, 1H), 3.34 – 3.29 (m, 1H), 3.26 – 3.22 (m, 1H), 1.89 – 1.83 (m, 1H), 1.77 – 1.70 (m, 1H), 1.64 – 1.56 (m, 1H), 1.38 – 1.30 (m, 1H). $^{13}$C NMR (100 MHz, DMSO-$d_6$) δ 162.2, 134.2, 134.1, 119.2, 115.5, 70.7, 68.3, 68.3, 65.9, 29.7, 24.8. HR-MS (ESI) m/z calc. for $C_{12}H_{15}NO_3Na$ $[M+Na]^+$: 244.0994, found: 244.0947.

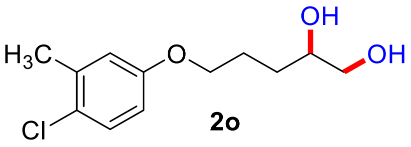

**2o**

**5-(4-Chloro-3-methylphenoxy)pentane-1,2-diol**

Compound **2o** was prepared following the general procedure, purification by column chromatography on silica gel (DCM/MeOH = 50:1) yielded **2o** (30.2 mg, 41%) as a colorless oil. $^{1}$H NMR (400 MHz, Chloroform-*d*) δ 7.21 (d, J = 8.8 Hz, 1H), 6.76 (d, J = 2.8 Hz, 1H), 6.66 (dd, J = 8.8, 2.8 Hz, 1H), 3.99 – 3.94 (m, 2H), 3.82 – 3.76 (m, 1H), 3.69 (dd, J = 11.2, 3.2 Hz, 1H), 3.48 (dd, J = 11.2, 7.2 Hz, 1H), 2.33 (s, 3H), 1.95 – 1.87 (m, 2H), 1.67 – 1.59 (m, 2H). $^{13}$C NMR (100 MHz, Chloroform-*d*) δ 157.3, 137.0, 129.6,

125.9, 117.1, 113.0, 71.8, 68.0, 66.8, 29.9, 25.4, 20.3. HR-MS (ESI) m/z calc. for $C_{12}H_{18}ClO_3$ $[M+H]^+$: 245.0939, found: 245.0938.

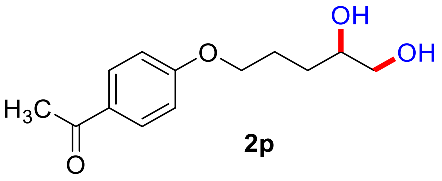


**2p**

**1-(4-((4,5-Dihydroxypentyl)oxy)phenyl)ethan-1-one**

Compound **2p** was prepared following the general procedure, purification by column chromatography on silica gel (DCM/MeOH = 50:1) yielded **2p** (40.8 mg, 57%) as a white solid. M.p.: 40–41 °C. $^1$H NMR (400 MHz, Chloroform-*d*) δ 7.91 – 7.89 (m, 2H), 6.91 – 6.89 (m, 2H), 4.07 – 4.03 (m, 2H), 3.82 – 3.76 (m, 1H), 3.71 – 3.68 (m, 1H), 3.51 – 3.47 (m, 1H), 2.73 (s br, 2H), 2.54 (s, 3H), 2.03 – 1.85 (m, 2H), 1.69 – 1.57 (m, 2H). $^{13}$C NMR (100 MHz, Chloroform-*d*) δ 197.0, 162.8, 130.6, 130.2, 114.1, 71.8, 68.0, 66.7, 29.6, 26.3, 25.3. HR-MS (ESI) m/z calc. for $C_{13}H_{19}O_4$ $[M+H]^+$: 239.1278, found: 239.1283.

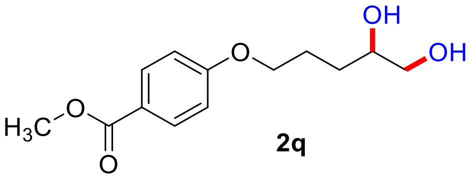


**2q**

**Methyl-4-((4,5-dihydroxypentyl)oxy)benzoate**

Compound **2q** was prepared following the general procedure, purification by column chromatography on silica gel (DCM/MeOH = 50:1) yielded **2q** (45.0 mg, 59%) as a white solid. M.p.: 45–46 °C. $^1$H NMR (400 MHz, Chloroform-*d*) δ 7.98 – 7.96 (m, 2H), 6.90 – 6.88 (m, 2H), 4.06 – 4.02 (m, 2H), 3.87 (s, 3H), 3.81 – 3.77 (m, 1H), 3.69 (dd, J = 11.2, 2.8 Hz, 1H), 3.48 (dd, J = 11.2, 7.2 Hz, 1H), 2.32 (s br, 2H), 2.01 – 1.89 (m, 2H), 1.67 – 1.58 (m, 2H). $^{13}$C NMR (100 MHz, Chloroform-*d*) δ 166.9, 162.6, 131.6, 122.5, 114.0, 71.8, 67.9, 66.8, 51.7, 29.7, 25.3. HR-MS (ESI) m/z calc. for $C_{13}H_{19}O_5$ $[M+H]^+$: 255.1227, found: 255.1228.

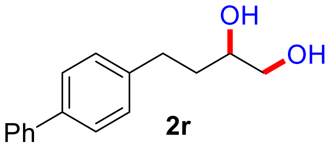


**2r**

**4-([1,1'-Biphenyl]-4-yl)butane-1,2-diol**

Compound **2r** was prepared following the general procedure, purification by column chromatography on silica gel (DCM/MeOH = 50:1) yielded **2r** (48.1 mg, 66%) as a white solid. $^1$H NMR (400 MHz, DMSO-$d_6$) δ 7.64 – 7.62 (m, 2H), 7.57 – 7.55 (m, 2H), 7.46 – 7.42 (m, 2H), 7.35 – 7.28 (m, 3H), 4.54 (s br, 2H), 3.45 – 3.43 (m, 1H), 3.37 – 3.29 (m, 2H), 2.78 – 2.74 (m, 1H), 2.67 – 2.61 (m, 1H), 1.79 – 1.75 (m, 1H), 1.58 – 1.53 (m, 1H). $^{13}$C NMR (100 MHz, DMSO-$d_6$) δ 141.9, 140.2, 137.5, 128.2, 128.9, 127.1, 126.5, 126.5, 70.4, 65.9, 35.3, 30.9. Data in concordance with literature.[5]

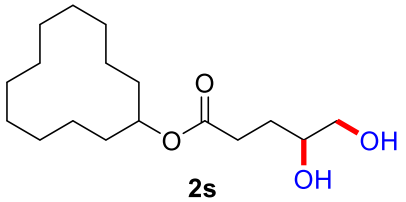


**2s**

**Cyclododecyl-4,5-dihydroxypentanoate**

Compound **2s** was prepared following the general procedure, purification by column chromatography on silica gel (DCM/MeOH = 50:1) yielded **2s** (56.9 mg, 63%) as a colorless oil. $^1$H NMR (400 MHz, Chloroform-$d$) δ 5.04 – 4.98 (m, 1H), 3.74 – 3.68 m, 1H), 3.62 (dd, J = 11.2, 2.8 Hz, 1H), 3.45 (dd, J = 11.2, 7.8 Hz, 1H), 2.94 (s br, 2H), 2.46 – 2.42 (m, 2H), 1.78 – 1.66 (m, 4H), 1.52 – 1.46 (m, 2H), 1.37 – 1.33 (m, 18H). $^{13}$C NMR (100 MHz, Chloroform-$d$) δ 173.9, 72.7, 72.6, 71.5, 66.5, 47.0, 30.9, 30.9, 30.8, 29.0, 28.0, 24.02, 23.8, 23.3, 23.2, 23.1, 20.8. HR-MS (ESI) m/z calc. for $C_{17}H_{33}O_3$ $[M+H]^+$: 301.2373, found: 301.2369.

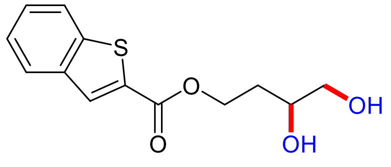


**2t**

**3,4-Dihydroxybutyl benzo[*b*]thiophene-2-carboxylate**

Compound **2t** was prepared following the general procedure, purification by column chromatography on silica gel (DCM/MeOH = 50:1) yielded **2t** (43.0 mg, 54%) as a white solid. M.p.: 48–49 °C. $^1$H NMR (400 MHz, DMSO-$d_6$) δ 8.20 (s, 1H), 8.05 (dd, J = 11.4, 8.0 Hz, 2H), 7.55 – 7.46 (m, 2H), 4.56 – 4.49 (m, 2H), 4.32 (t, J = 6.4 Hz, 2H),

3.49 – 3.44 (m, 1H), 3.33 – 3.24 (m, 2H), 1.89 – 1.83 (m, 1H), 1.78 – 1.69 (m, 1H), 1.64 – 1.56 (m, 1H), 1.38 – 1.34 (m, 1H). $^{13}$C NMR (100 MHz, DMSO-$d_6$) δ 162 5, 141.7, 138.9, 133.4, 131.1, 127.7, 126.3, 125.6, 123.4, 71.1, 66.3, 66.1, 30.1, 25.1. HR-MS (ESI) m/z calc. for $C_{13}H_{15}O_4S$ [M+H]$^+$: 267.0686, found: 267.0689.

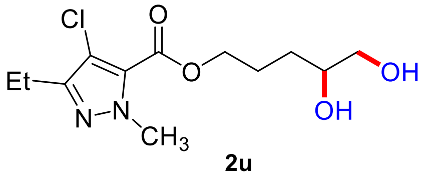


**2u**

**4,5-Dihydroxypentyl4-chloro-3-ethyl-1-methyl-1H-pyrazole-5carboxylate**

Compound **2u** was prepared following the general procedure, purification by column chromatography on silica gel (DCM/MeOH = 50:1) yielded **2u** (54.8 mg, 63%) as a colorless oil.$^1$H NMR (400 MHz, Chloroform-*d*) δ 4.35 (t, J = 6.3 Hz, 2H), 4.07 (s, 3H), 3.77 – 3.74(m, 1H), 3.65 (dd, J = 11.1, 2.8 Hz, 1H), 3.45 (dd, J = 11.0, 7.6 Hz, 1H), 2.73 (s br, 2H), 2.61 (q, J = 7.6 Hz, 2H), 2.00 – 1.91 (m, 1H), 1.89 – 1.79 (m, 1H), 1.64 – 1.55 (m, 2H), 1.21 (t, J = 7.6 Hz, 3H). $^{13}$C NMR (100 MHz, Chloroform-*d*) δ 159.2, 150.4, 128.8, 113.0, 71.6, 66.7, 65.1, 40.5, 29.4, 24.7, 19.2, 12.7. HR-MS (ESI) m/z calc. for $C_{12}H_{19}ClN_2O_4$ [M+H]$^+$: 291.1106, found: 291.1107.

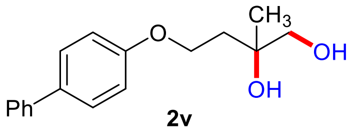


**2v**

**4-([1,1'-Biphenyl]-4-yloxy)-2-methylbutane-1,2-diol**

Compound **2v** was prepared following the general procedure, purification by column chromatography on silica gel (DCM/MeOH = 50:1) yielded **2v** (41.8 mg, 51%) as a white solid. M.p.: 80–81 °C. $^1$H NMR (400 MHz, Chloroform-*d*) δ 7.56 – 7.52 (m, 4H), 7.44 – 7.40 (m, 2H), 7.33 – 7.30 (m, 1H), 7.00 – 6.98 (m, 2H), 4.29 – 4.17 (m, 2H), 3.54 (dd, J = 24.4, 11.2 Hz, 2H), 2.59 (s br, 2H), 2.18 – 2.11 (m, 1H), 2.01 – 1.94 (m, 1H), 1.29 (s, 3H). $^{13}$C NMR (100 MHz, Chloroform-*d*) δ 157.7, 140.6, 134.4, 128.7, 128.2, 126.8, 126.7, 114.8, 72.3, 70.0, 64.7, 37.4, 24.1. HR-MS (ESI) m/z calc. for $C_{17}H_{21}O_3$ [M+H]$^+$: 273.1485, found: 273.1490.

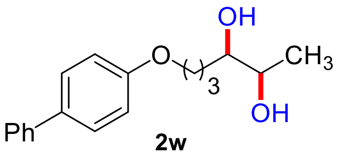


**2w**

**6-([1,1'-Biphenyl]-4-yloxy)hexane-2,3-diol**

Compound **2w** was prepared following the general procedure, purification by column chromatography on silica gel (DCM/MeOH = 50:1) yielded **2w** (54.9 mg, 64%) as a white solid. M.p.: 85–86 °C. $^{1}$H NMR (400 MHz, Chloroform-*d*) 7.56 – 7.50 (m, 4H), 7.43 – 7.39 (m, 2H), 7.32 – 7.28 (m, 1H), 6.99 – 6.95 (m, 2H), 6.86 (d, J = 8.8 Hz, 0.1H), 4.10 – 4.01 (m, 2H), 3.86 – 3.83 (m, 1H), 3.69 (dd, J = 12.4, 3.2 Hz, 1H), 2.09 – 1.99 (m, 3H), 1.96 – 1.85 (m, 1H), 1.74 – 1.66 (m, 1H), 1.62 – 1.53 (m, 1H), 1.18 (d, J = 6.4 Hz, 3H). $^{13}$C NMR (100 MHz, Chloroform-*d*) δ 158.3, 140.8, 133.8, 128.7, 128.1, 126.7, 126.6, 114.7, 74.6, 70.5, 68.0, 28.4, 26.0, 16.9. HR-MS (ESI) m/z calc. for $C_{18}H_{23}O_3$ [M+H]$^+$: 287.1642, found: 287.1639.

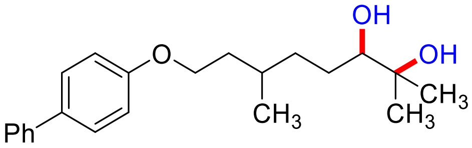


**2x**

**8-([1,1'-Biphenyl]-4-yloxy)-2,6-dimethyloctane-2,3-diol**

Compound **2x** was prepared following the general procedure, purification by column chromatography on silica gel (DCM/MeOH = 50:1) yielded **2x** (68.8 mg, 67%) as a white solid. M.p.: 66–67 °C. $^{1}$H NMR (400 MHz, Chloroform-*d*) δ 7.57 – 7.50 (m, 8H), 7.44 – 7.40 (m, 4H), 7.33 – 7.29 (m, 2H), 6.99 – 6.96 (m, 4H), 4.08 – 4.03 (m, 4H), 3.38 – 3.34 (m, 2H), 2.66 – 2.03 (m, 4H), 1.92 – 1.85 (m, 2H), 1.78 – 1.57 (m, 6H), 1.53 – 1.28 (m, 6H), 1.23 (s, 6H), 1.17 (s, 6H), 1.00 (d, J = 4.0 Hz, 3H), 0.99 (d, J = 4.0 Hz, 3H). $^{13}$C NMR (100 MHz, Chloroform-*d*) δ 158.6, 140.8, 140.8, 133.6, 133.6, 128.7, 128.1, 126.7, 126.6, 114.7, 79.0, 78.7, 73.2, 73.2, 66.2, 36.3, 35.9, 34.2, 33.9, 30.0, 29.9, 29.0, 28.9, 26.6, 26.5, 23.2, 19.8, 19.5. HR-MS (ESI) m/z calc. for $C_{22}H_{31}O_3$ [M+H]$^+$: 343.2268, found: 343.2269.

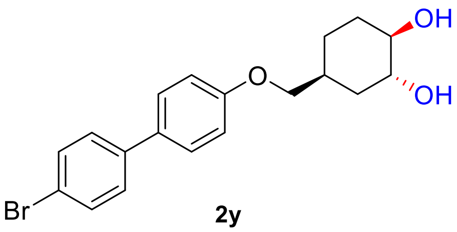

2y

**4-(((4'-Bromo-[1,1'-biphenyl]-4-yl)oxy)methyl)cyclohexane-1,2-diol**

Compound **2y** was prepared following the general procedure, purification by column chromatography on silica gel (DCM/MeOH = 50:1) yielded **2y** (93.9 mg, 83%) as a white solid. 66–67 °C. $^1$H NMR (400 MHz, DMSO-$d_6$) δ 7.59 – 7.53 (m, 6H), 6.99 (d, J = 8.8 Hz, 2H), 4.57 (d, J = 21.9 Hz, 2H), 3.80 (d, J = 6.6 Hz, 2H), 3.54 (d, J = 37.7 Hz, 2H), 2.17 – 2.04 (m, 1H), 1.75 – 1.67 (m, 1H), 1.63 – 1.51 (m, 2H), 1.46 – 1.45 (m, 3H). $^{13}$C NMR (100 MHz, DMSO- $d_6$) δ 158.7, 139.0, 131.7, 130.9, 128.2, 127.7, 119.9, 115.0, 72.2, 69.2, 68.6, 31.3, 31.1, 27.1, 23.1. HR-MS (ESI) m/z calc. for $C_{19}H_{22}BrO_3$ $[M+H]^+$: 377.0747, found: 377.0746.

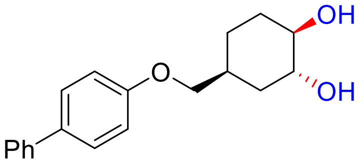

2y

**4-(([1,1'-Biphenyl]-4-yloxy)methyl)cyclohexane-1,2-diol**

Compound **2z** was prepared following the general procedure, purification by column chromatography on silica gel (DCM/MeOH = 50:1) yielded **2z** (77.0 mg, 86%) as a white solid. M.p.: 65–66 °C. $^1$H NMR (400 MHz, DMSO-$d_6$) δ 7.61 – 7.56 (m, 4H), 7.44 – 7.40 (m, 2H), 7.30 (t, J = 7.2 Hz, 1H), 7.02 – 7.00 (m, 2H), 4.54 (dd, J = 22.8, 3.6 Hz, 2H), 3.82 (d, J = 6.8 Hz, 2H), 3.60 – 3.56 (m, 1H), 3.50 – 3.47 (m, 1H), 2.11 (s br, 1H), 1.77 – 1.68 (m, 1H), 1.60 – 1.55 (m, 2H), 1.48 – 1.42 (m, 3H). $^{13}$C NMR (100 MHz, DMSO-$d_6$) δ 158.5, 139.9, 132.3, 128.8, 127.7, 126.6, 126.1, 114.9, 72.2, 69.2, 68.6, 31.4, 31.1, 27.1, 23.1. HR-MS (ESI) m/z calc. for $C_{19}H_{22}O_3Na$ $[M+Na]^+$: 321.1461, found: 321.1466.

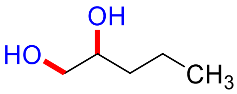

2aa

**Pentane-1,2-diol**

Compound **2aa** was prepared following the general procedure, purification by column chromatography on silica gel (DCM/MeOH = 50:1) yielded **2aa** (12.9 mg, 41%) as a colorless oli. $^1$H NMR (400 MHz, Chloroform-*d*) δ 3.67 – 3.45 (m, 4H), 3.39 (t, J = 8.8 Hz, 1H), 1.46 – 1.33 (m, 4H), 0.91 (t, J = 6.4 Hz, 3H). $^{13}$C NMR (100 MHz, Chloroform-*d*) δ 72.0, 66.7, 35.1, 18.7, 14.0. Data in concordance with literature.[6]

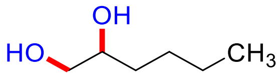


**2ab**

**Hexane-1,2-diol**

Compound **2ab** was prepared following the general procedure, purification by column chromatography on silica gel (DCM/MeOH = 50:1) yielded **2ab** (18.5 mg, 52%) as a colorless oli. $^1$H NMR (400 MHz, Chloroform-*d*) δ 3.80 (s, 2H), 3.67 – 3.61 (m, 1H), 3.58 (dd, J = 11.2, 2.8 Hz, 1H), 3.37 (dd, J = 11.2, 8.0 Hz, 1H), 1.40 – 1.34 (m, 3H), 1.33 – 1.26 (m, 3H), 0.88 (t, J = 7.2 Hz, 3H). $^{13}$C NMR (100 MHz, Chloroform-*d*) δ 72.3, 66.7, 32.7, 27.7, 22.7, 13.9. Data in concordance with literature.[6]

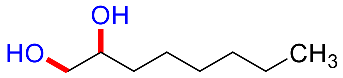


**2ac**

**Octane-1,2-diol**

Compound **2ac** was prepared following the general procedure, purification by column chromatography on silica gel (DCM/MeOH = 50:1) yielded **2ac** (26.7 mg, 61%) as a white solid. $^1$H NMR (400 MHz, Chloroform-*d*) δ 3.73 – 3.67 (m, 1H), 3.64 (dd, J = 11.2, 2.8 Hz, 1H), 3.42 (dd, J = 11.2, 7.6 Hz, 1H), 2.33 (s br, 2H), 1.43 – 1.25 (m, 10H), 0.88 (t, J = 6.8 Hz, 3H). $^{13}$C NMR (100 MHz, Chloroform-*d*) δ 72.3, 66.8, 33.2, 31.7, 29.3, 25.5, 22.6, 14.0. Data in concordance with literature.[6]

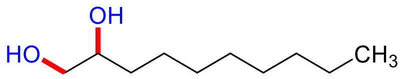


**2ad**

**Decane-1,2-diol**

Compound **2ad** was prepared following the general procedure, purification by column chromatography on silica gel (DCM/MeOH = 50:1) yielded **2ad** (37.1mg, 71%) as a white solid. $^1$H NMR (400 MHz, Chloroform-*d*) δ 3.70 – 3.61 (m, 2H), 3.41 (dd, J = 11.2, 7.6 Hz, 1H), 2.95 (s br, 2H), 1.41 (t, J = 5.2 Hz, 3H), 1.31 – 1.22 (m, 11H), 0.88 – 0.85 (m, 3H). $^{13}$C NMR (100 MHz, Chloroform-*d*) δ 72.4, 66.8, 33.1, 31.8, 29.7, 29.5, 29.3, 25.6, 22.6, 14.1. Data in concordance with literature.[6]

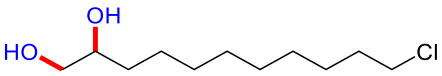


**2ae**

**11-Chloroundecane-1,2-diol**

Compound **2ae** was prepared following the general procedure, purification by column chromatography on silica gel (DCM/MeOH = 50:1) yielded **2ae** (44.7 mg, 67%) as a white solid. $^1$H NMR (400 MHz, Chloroform-*d*) δ 3.68 – 3.66 (m, 1H), 3.62 (dd, J = 11.2, 2.8 Hz, 1H), 3.52 (t, J = 6.8 Hz, 2H), 3.40 (dd, J = 11.2, 7.6 Hz, 1H), 3.06 (s br, 2H), 1.78 – 1.71 (m, 2H), 1.42–1.24 (m, 15H). $^{13}$C NMR (100 MHz, Chloroform-*d*) δ 72.3, 66.7, 45.1, 33.1, 32.6, 29.7, 29.4, 29.3, 28.8, 26.8, 25.5. Data in concordance with literature.[7]

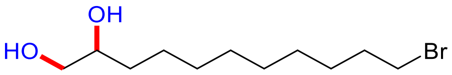


**2af**

**11-Bromoundecane-1,2-diol**

Compound **2af** was prepared following the general procedure, purification by column chromatography on silica gel (DCM/MeOH = 50:1) yielded **2af** (48.9 mg, 61%) as a white solid. $^1$H NMR (400 MHz, Chloroform-*d*) δ 3.69 – 3.61 (m, 2H), 3.43 – 3.37 (m, 3H), 2.79 (s br, 2H), 1.85 – 1.80 (m, 2H), 1.42 – 1.39 (m, 5H), 1.28 (s, 9H). $^{13}$C NMR (100 MHz, Chloroform-*d*) δ 72.3, 66.8, 34.0, 33.1, 32.8, 29.5, 29.4, 29.3, 28.7, 28.1, 25.5. Data in concordance with literature.[7]

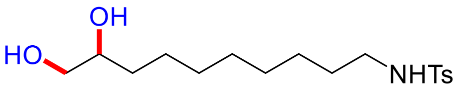


**2ag**

**N-(9,10-Dihydroxydecyl)-4-methylbenzenesulfonamide**

Compound **2ag** was prepared following the general procedure, purification by column chromatography on silica gel (DCM/MeOH = 50:1) yielded **2ag** (73.1 mg, 71%) as a colorless oil.$^{1}$H NMR (400 MHz, Chloroform-*d*) δ 7.73 (d, J = 8.2 Hz, 2H), 7.29 (d, J = 8.0 Hz, 2H), 5.13 – 4.92 (m, 1H), 3.71 – 3.58 (m, 2H), 3.44 – 3.37 (m, 1H), 2.88 (q, J = 6.4 Hz, 2H), 2.72 (s br, 2H), 2.41 (s, 3H), 1.44 – 1.22 (m 14H). $^{13}$C NMR (100 MHz, Chloroform-*d*) δ 143.2, 136.9, 129.6, 127.0, 72.1, 66.7, 43.0, 32.9, 29.6, 29.3, 29.2, 28.7, 26.2, 25.2, 21.4. HR-MS (ESI) m/z calc. for $C_{17}H_{30}NO_4S$[M+H]$^+$: 344.1890, found: 344.1891.

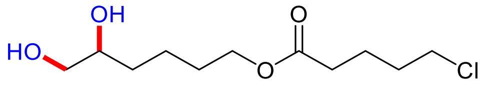


**2ah**

**5,6-Dihydroxyhexyl 5-chloropentanoate**

Compound **2ah** was prepared following the general procedure, purification by column chromatography on silica gel (DCM/MeOH = 50:1) yielded **2ah** (59.1 mg, 78%) as a colorless oil. $^{1}$H NMR (400 MHz, Chloroform-*d*) δ 4.06 (t, J = 6.4 Hz, 2H), 3.67 – 3.64 (m, 1H), 3.60 (dd, J = 11.2, 2.8 Hz, 1H), 3.54 – 3.51 (m, 2H), 3.40 (dd, J = 11.2, 7.6 Hz, 1H), 2.95 (s br, 2H), 2.32 (t, J = 7.2 Hz, 2H), 1.80 – 1.72 (m, 4H), 1.66 – 1.59 (m, 2H), 1.50 – 1.34 (m, 4H). $^{13}$C NMR (100 MHz, Chloroform-*d*) δ 173.4, 72.0, 66.6, 64.3, 44.4, 33.4, 32.5, 31.8, 28.6, 22.2, 22.0. HR-MS (ESI) m/z calc. for $C_{11}H_{22}ClO_4$ [M+H]$^+$: 253.1201, found: 253.1199.

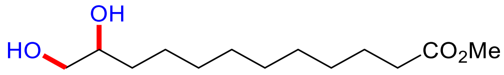


**2ai**

**Methyl 11,12-dihydroxydodecanoate**

Compound **2ai** was prepared following the general procedure, purification by column chromatography on silica gel (DCM/MeOH = 50:1) yielded **2ai** (45.9 mg, 62%) as a colorless oil. $^{1}$H NMR (400 MHz, Chloroform-*d*) δ 3.69 –3.67 (m, 1H), 3.65 (s, 3H), 3.62 – 3.61 (m, 1H), 3.41 (dd, J = 10.8, 7.6 Hz, 1H), 2.72 (s br, 2H), 2.29 (t, J = 7.6 Hz, 2H), 1.62 – 1.58 (m, 2H), 1.43 – 1.25 (m, 14H). $^{13}$C NMR (100 MHz, Chloroform-*d*) δ

174.4, 72.3, 66.8, 51.4, 34.1, 33.1, 29.5, 29.2, 29.1, 29.1, 27.5, 25.5, 24.9. Data in concordance with literature.[7]

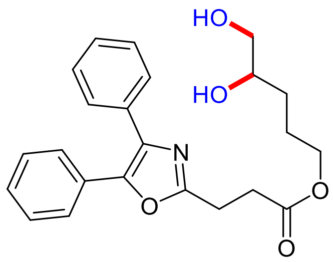


**2aj**

**4,5-Dihydroxypentyl 3-(4,5-diphenyloxazol-2-yl)propanoate**

Compound **2aj** was prepared following the general procedure, purification by column chromatography on silica gel (DCM/MeOH = 50:1) yielded **2aj** (78.4 mg, 66%) as a colorless oil. $^{1}$H NMR (400 MHz, Chloroform-*d*) δ 7.63 – 7.60 (m, 2H), 7.57 – 7.55 (m, 2H), 7.38 – 7.31 (m, 6H), 4.17 – 4.13 (m, 2H), 3.68 – 3.62 (m, 1H), 3.54 (dd, J = 11.2, 3.6 Hz, 1H), 3.35 (dd, J = 11.2, 3.6 Hz, 1H), 3.18 (t, J = 7.6 Hz, 2H), 2.91 (t, J = 7.6 Hz, 2H), 2.55 (s br, 2H), 1.80 – 1.67 (m, 2H), 1.48 – 1.42 (m, 2H). $^{13}$C NMR (100 MHz, Chloroform-*d*) δ 172.0, 161.8, 145.4, 135.0, 132.3, 128.8, 128.6, 128.5, 128.5, 128.1, 127.9, 126.4, 71.6, 66.6, 64.7, 31.1, 29.4, 24.8, 23.5. HR-MS (ESI) m/z calc. for $C_{23}H_{26}NO_5$ $[M+H]^+$: 396.1805, found: 396.1802.

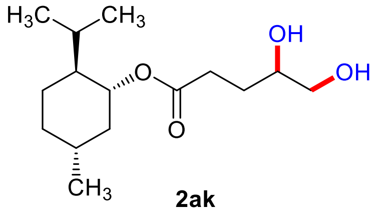


**2ak**

**(1*R*,2*S*,5*R*)-2-Isopropyl-5-methylcyclohexyl-4,5-dihydroxypentanoate**

Compound **2ak** was prepared following the general procedure, purification by column chromatography on silica gel (DCM/MeOH = 50:1) yielded **2ak** (46.6 mg, 57%) as a colorless oil. $^{1}$H NMR (400 MHz, Chloroform-*d*) δ 4.70 – 4.64 (m, 1H), 3.72 – 3.61 (m, 2H), 3.47 – 3.43 (m, 1H), 3.04 (s br, 2H), 2.50 –2.40 (m, 2H), 1.95 (d, J = 11.6 Hz, 1H), 1.84 – 1.75 (m, 3H), 1.68 – 1.63 (m, 2H), 1.51 – 1.42 (m, 1H), 1.38 – 1.33 (m, 1H), 1.05 – 0.94 (m, 2H), 0.89– 0.87 (m, 7H), 0.74 (d, J = 6.8 Hz, 3H). $^{13}$C NMR (100 MHz, Chloroform-*d*) δ 173.8, 74.5, 71.5, 66.4, 46.9, 40.8, 34.2, 31.3, 30.9, 28.0, 26.3, 23.4,

22.0, 20.7, 16.3. HR-MS (ESI) m/z calc. for $C_{15}H_{29}O_4Na$ [M+Na]$^+$: 295.1880, found: 295.1881.

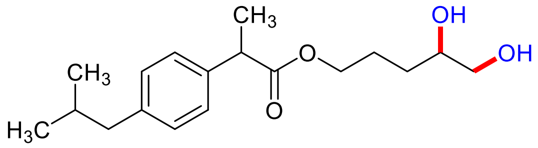


**2al**

**4,5-Dihydroxypentyl 2-(4-isobutylphenyl)propanoate**

Compound **2al** was prepared following the general procedure, purification by column chromatography on silica gel (DCM/MeOH = 50:1) yielded **2al** (49.1 mg, 53%) as a colorless oil. $^1$H NMR (400 MHz, Chloroform-*d*) δ 7.18 (d, J = 8.0 Hz, 2H), 7.08 (d, J = 8.0 Hz, 2H), 4.11 – 4.06 (m, 2H), 3.69 – 3.60 (m, 2H), 3.55 – 3.51 (m, 1H), 3.36 – 3.33 (m, 1H), 2.97 (s br, 2H), 2.44 (d, J = 7.2 Hz, 2H), 1.85 – 1.80 (m, 1H), 1.76 – 1.71 (m, 1H), 1.67 – 1.62 (m, 1H), 1.47 (d, J = 6.8 Hz, 3H), 1.34 (d, J = 7.6 Hz, 2H), 0.90 (s, 3H), 0.88 (s, 3H). $^{13}$C NMR (100 MHz, Chloroform-*d*) δ 174.9, 140.5, 137.6, 129.2, 127.1, 71.5, 66.6, 64.4, 58.2, 45.1, 44.9, 30.1, 29.6, 29.1, 24.7, 22.3, 18.3, 18.2. HR-MS (ESI) m/z calc. for $C_{18}H_{29}O_4$ [M+H]$^+$: 309.2060, found: 309.2061.

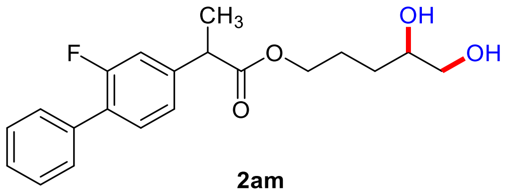


**2am**

**4,5-Dihydroxypentyl 2-(2-fluoro-[1,1'-biphenyl]-4-yl)propanoate**

Compound **2am** was prepared following the general procedure, purification by column chromatography on silica gel (DCM/MeOH = 50:1) yielded **2am** (64.5 mg, 62%) as a colorless oil. $^1$H NMR (400 MHz, Chloroform-*d*) δ 7.54 – 7.52 (m, 2H), 7.45 – 7.34 (m, 4H), 7.15 – 7.10 (m, 2H), 4.14 – 4.11 (m, 2H), 3.77 – 3.72 (m, 1H), 3.66 – 3.64 (m, 1H), 3.59 – 3.54 (m, 1H), 3.39 – 3.36 (m, 1H), 2.68 (s br, 2H), 1.82 – 1.75 (m, 1H), 1.71 – 1.64 (m, 1H), 1.53 (d, J = 7.2 Hz, 3H), 1.42 – 1.37 (m, 2H). $^{13}$C NMR (100 MHz, Chloroform-*d*) δ 174.1, 159.5 (d, $J_{C-F}$ = 247.0 Hz), 141.8 (d, $J_{C-F}$ = 2.0 Hz), 141.7 (d, $J_{C-F}$ = 1.0 Hz), 135.3, 130.7 (d, $J_{C-F}$ = 4.0 Hz), 128.9 (d, $J_{C-F}$ = 3.0 Hz), 128.4, 127.6, 123.5 (d, $J_{C-F}$ = 3.0 Hz), 115.2 (d, $J_{C-F}$ = 23.0 Hz). HR-MS (ESI) m/z calc. for $C_{20}H_{24}FO_4$ [M+H]$^+$: 347.1653, found: 347.1646.

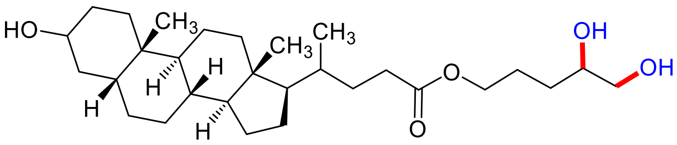


**2an**

**4,5-Dihydroxypentyl-4-((5*R*,8*R*,9*S*,10*S*,13*R*,14*S*,17*R*)-3-hydroxy-10,13-dimethyl-hex-adecahydro-1H-cyclopenta[a]phenanthren-17-yl)pentanoate**

Compound **2an** was prepared following the general procedure, purification by column chromatography on silica gel (DCM/MeOH = 50:1) yielded **2an** (73.5 mg, 51%) as a colorless oil. $^{1}$H NMR (400 MHz, Chloroform-*d*) δ 4.09 – 4.06 (m, 2H), 3.71 (s, 1H), 3.65 – 3.58 (m, 2H), 3.46 – 3.41 (m, 1H), 2.91 (s br, 3H), 2.37 – 2.29 (m, 1H), 2.23 – 2.17 (m, 1H), 1.86 – 1.63 (m, 10H), 1.55 – 1.47 (m, 4H), 1.41 – 1.32 (m, 7H), 1.26 – 1.23 (m, 5H), 1.12 – 1.04 (m, 4H), 0.90 – 0.89 (m, 6H), 0.63 (s, 3H). $^{13}$C NMR (100 MHz, Chloroform-*d*) δ 174.5, 71.7, 66.6, 6.13, 56.5, 55.8, 42.7, 42.0, 40.4, 40.1, 36.3, 35.8, 35.3, 34.5, 31.1, 30.9, 30.4, 29.4, 28.2, 27.1, 26.4, 24.7, 24.2, 23.3, 23.3, 20.8, 18.2, 12.0. HR-MS (ESI) m/z calc. for $C_{29}H_{51}O_5$ $[M+H]^+$: 479.3731, found: 479.3728.

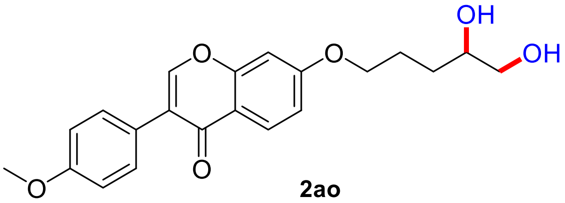


**2ao**

**7-((4,5-Dihydroxypentyl)oxy)-3-(4-methoxyphenyl)-4H-chromen-4-one**

Compound **2ao** was prepared following the general procedure, purification by column chromatography on silica gel (DCM/MeOH = 50:1) yielded **2ao** (52.3 mg, 47%) as a white solid. M.p.: 52–53 °C. $^{1}$H NMR (400 MHz, DMSO-*d*$_6$) δ 8.41 (s, 1H), 8.02 (d, J = 8.9 Hz, 1H), 7.52 (d, J = 8.7 Hz, 2H), 7.15 (d, J = 2.1 Hz, 1H), 7.07 (dd, J = 8.9, 2.2 Hz, 1H), 7.05 – 6.93 (m, 2H), 4.52 (s, 2H), 4.14 (t, J = 6.5 Hz, 2H), 3.79 (s, 3H), 3.49 – 3.44 (m, 1H), 3.32 – 3.23 (m, 2H), 1.93 – 1.87 (m, 1H), 1.82 – 1.73 (m, 1H), 1.67 – 1.58 (m, 1H), 1.40 – 1.36 (m, 1H). $^{13}$C NMR (100 MHz, DMSO-*d*$_6$) δ 175.0, 163.5, 159.4, 157.8, 153.8, 130.6, 127.3, 124.5, 123.8, 117.9, 115.5, 114.0, 101.6, 101.4, 71.2, 69.2, 66.3, 55.6, 30.1, 25.2. HR-MS (ESI) m/z calc. for $C_{21}H_{23}O_6$ $[M+H]^+$: 371.1489, found: 371.1485.

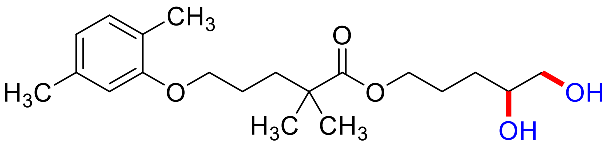

**2ap**

Compound **2ap** was prepared following the general procedure, purification by column chromatography on silica gel (DCM/MeOH = 50:1) yielded **2ap** (59.5 mg, 61%) as a colorless oli. $^{1}$H NMR (400 MHz, Chloroform-*d*) δ 7.00 (d, J = 7.5 Hz, 1H), 6.66 (d, J = 7.5 Hz, 1H), 6.61 (s, 1H), 4.10 (t, J = 6.6 Hz, 2H), 3.91 (t, J = 5.1 Hz, 2H), 3.72 – 3.68 (m, 1H), 3.63 (dd, J = 11.1, 3.0 Hz, 1H), 3.42 (dd, J = 11.0, 7.6 Hz, 1H), 2.48 (s br, 2H), 2.30 (s, 3H), 2.17 (s, 3H), 1.85 – 1.77 (m, 1H), 1.74 – 1.66 (m, 5H), 1.51 – 1.45 (m, 2H), 1.21 (s, 6H). $^{13}$C NMR (100 MHz, Chloroform-*d*) δ 177.9, 156.9, 136.4, 130.3, 123.5, 120.7, 112.0, 71.6, 67.9, 66.7, 64.2, 42.1, 37.0, 29.4, 25.1, 24.8, 21.4, 15.7. HR-MS (ESI) m/z calc. for $C_{20}H_{33}O_5$ $[M+H]^+$: 353.2323, found: 353.2321.

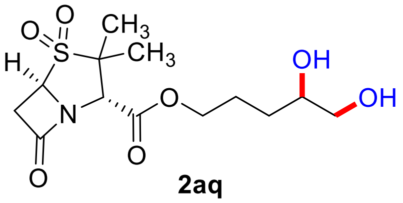

**2aq**

Compound **2aq** was prepared following the general procedure, purification by column chromatography on silica gel (DCM/MeOH = 50:1) yielded **2aq** (52.3 mg, 52%) as a colorless oli. $^{1}$H NMR (400 MHz, Chloroform-*d*) δ 4.66 – 4.59 (m, 1H), 4.36 (s, 1H), 4.25 – 4.17 (m, 2H), 3.67 (d, J = 7.0 Hz, 1H), 3.59 (d, J = 10.8 Hz, 1H), 3.51 – 3.46 (m, 1H), 3.43 – 3.35 (m, 2H), 3.14 (s br, 2H), 1.87 – 1.70 (m, 2H), 1.57 (s, 3H), 1.50 – 1.42 (m, 2H), 1.38 (s, 3H). $^{13}$C NMR (100 MHz, Chloroform-*d*) δ 171.2, 166.9, 71.4, 66.5, 66.4, 63.1, 62.7, 61.0, 38.2, 29.1, 24.6, 20.2, 18.5. HR-MS (ESI) m/z calc. for $C_{13}H_{22}NO_7S$ $[M+H]^+$: 336.1111, found: 336.1115.

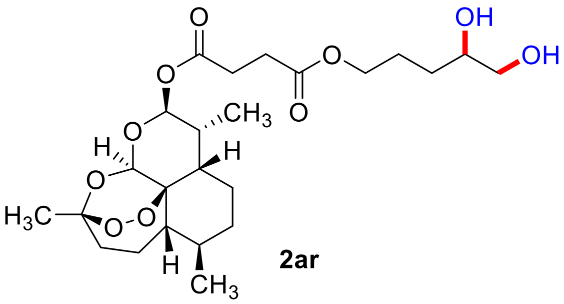

**2ar**

Compound **2ar** was prepared following the general procedure, purification by column chromatography on silica gel (DCM/MeOH = 50:1) yielded **2ar** (62.7 mg, 43%) as a colorless oli. $^{1}$H NMR (400 MHz, Chloroform-*d*) δ 5.77 (dd, J = 9.9, 2.0 Hz, 1H), 5.44 (d, J = 2.6 Hz, 1H), 4.23 – 4.05 (m, 2H), 3.73 – 3.71 (m, 1H), 3.66 – 3.63 (m, 1H), 3.49 – 3.41 (m, 1H), 2.74 – 2.71 (m, 2H), 2.67 – 2.60 (m, 2H), 2.57 – 2.53 (m, 1H), 2.41 – 2.32 (m, 1H), 2.19 (s br, 2H), 2.06 – 2.00 (m, 1H), 1.91 – 1.86 (m, 1H), 1.83 – 1.68 (m, 4H), 1.65 – 1.59 (m, 1H), 1.51 – 1.48 (m, 2H), 1.43 (s, 3H), 1.39 – 1.23 (m, 4H), 1.06 – 0.97 (m, 1H), 0.96 (d, J = 5.9 Hz, 3H), 0.85 (d, J = 7.1 Hz, 3H). $^{13}$C NMR (100 MHz, Chloroform-*d*) δ 172.0, 171.4, 104.5, 92.2, 91.5, 80.1, 71.6, 66.7, 66.5, 64.6, 64.6, 51.5, 45.1, 37.2, 36.1, 34.0, 31.7, 29.4, 29.2, 28.9, 25.8, 24.7, 24.5, 21.9, 20.1, 12.0. HR-MS (ESI) m/z calc. for $C_{24}H_{39}O_{10}$ $[M+H]^{+}$: 487.2538, found: 487.2531.

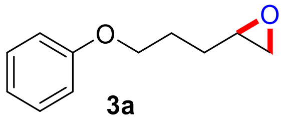

**3a**

**2-(3-Phenoxypropyl)oxirane**

Compound **3a** was prepared following the general procedure, purification by column chromatography on silica gel (petroleum ether/EtOAc = 50:1) yielded **3a** (44.4 mg, 83%) as a colorless oil. $^{1}$H NMR (400 MHz, Chloroform-*d*) δ 7.30 – 7.28 (m, 1H), 6.96 – 6.89 (m, 3H), 4.07 – 3.97 (m, 2H), 3.02 – 2.98 (m, 1H), 2.78 (t, J = 4.8 Hz, 1H), 2.52 (dd, J = 4.8, 2.8 Hz, 1H), 2.00 – 1.92 (m, 2H), 1.86 – 1.77 (m, 1H), 1.72 – 1.63 (m, 1H). $^{13}$C NMR (100 MHz, Chloroform-*d*) δ 158.9, 140.9, 129.4, 120.7, 114.4, 110.3, 67.2, 52.0, 47.1, 29.2, 25.8. Data in concordance with literature.[8]

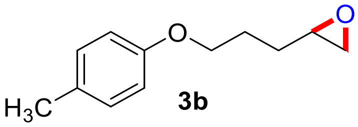

**3b**

**2-(3-(*p*-Tolyloxy)propyl)oxirane**

Compound **3b** was prepared following the general procedure, purification by column chromatography on silica gel (petroleum ether/EtOAc = 50:1) yielded **3b** (45.0 mg, 78%) as a colorless oil. $^{1}$H NMR (400 MHz, Chloroform-*d*) δ 7.09 – 7.07 (m, 2H), 6.82 – 6.78 (m, 2H), 4.04 – 3.94 (m, 2H), 3.02 – 2.98 (m, 1H), 2.80 (t, J = 4.8 Hz, 1H), 2.51

(dd, J = 4.8, 2.8 Hz, 1H), 2.29 (s, 3H), 1.99 – 1.90 (m, 2H), 1.85 – 1.76 (m, 1H), 1.72 – 1.65 (m, 1H). $^{13}$C NMR (100 MHz, Chloroform-*d*) δ 156.7, 129.8, 114.3, 67.3, 51.9, 47.0, 29.2, 25.8, 20.4. Data in concordance with literature.[8]

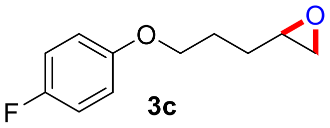

**3c**

**2-(3-(4-Fluorophenoxy)propyl)oxirane**

Compound **3c** was prepared following the general procedure, purification by column chromatography on silica gel (petroleum ether/EtOAc = 50:1) yielded **3c** (27.2 mg, 46%) as a colorless oil. $^{1}$H NMR (400 MHz, Chloroform-*d*) δ 6.99 – 6.93 (m, 2H), 6.85 – 6.79 (m, 2H), 4.02 – 3.92 (m, 2H), 3.01 – 2.97 (m, 1H), 2.78 (dd, J = 4.8, 4.0 Hz, 1H), 2.51 (dd, J = 5.2, 2.8 Hz, 1H), 1.98 – 1.90 (m, 2H), 1.85 – 1.77 (m, 1H), 1.69 – 1.60 (m, 1H). $^{13}$C NMR (100 MHz, Chloroform-*d*) 157.2 (d, $J_{C-F}$ = 237.0 Hz), 155.0 (d, $J_{C-F}$ = 1.0 Hz), 115.8 (d, $J_{C-F}$ = 23.0 Hz), 115.4 (d, $J_{C-F}$ = 8.0 Hz), 68.0, 52.0, 47.0, 29.1, 25.8. $^{19}$F NMR (375 MHz, Chloroform-*d*) δ -124.16. Data in concordance with literature.[8]

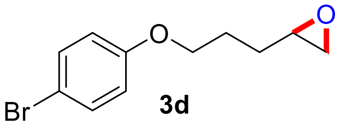

**3d**

**2-(3-(4-Bromophenoxy)propyl)oxirane**

Compound **3d** was prepared following the general procedure, purification by column chromatography on silica gel (petroleum ether/EtOAc = 50:1) yielded **3d** (32.8 mg, 42%) as a colorless oil. $^{1}$H NMR (400 MHz, Chloroform-*d*) δ 7.38 – 7.34 (m, 2H), 6.79 – 6.75 (m, 2H), 4.02 – 3.92 (m, 2H), 3.00 – 2.96 (m, 1H), 2.77 (t, J = 4.8 Hz, 1H), 2.50 (dd, J = 5.2, 2.8 Hz, 1H), 1.98– 1.90 (m, 2H), 1.85 – 1.76 (m, 1H), 1.68 – 1.59 (m, 1H). $^{13}$C NMR (100 MHz, Chloroform-*d*) δ 158.0, 132.2, 116.2, 112.7, 67.5, 51.9, 47.0, 29.1, 25.7. Data in concordance with literature.[8]

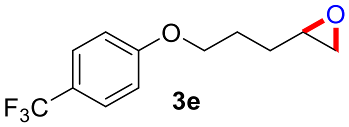

**3e**

**2-(3-(4-(Trifluoromethyl)phenoxy)propyl)oxirane**

Compound **3e** was prepared following the general procedure, purification by column

chromatography on silica gel (petroleum ether/EtOAc = 50:1) yielded **3e** (46.7 mg, 63%) as a colorless oil. $^1$H NMR (400 MHz, Chloroform-*d*) δ 7.53 (d, J = 8.4 Hz, 2H), 6.95 (d, J = 8.8 Hz, 2H), 4.10 – 4.01 (m, 2H), 3.02 – 2.97 (m, 1H), 2.79 (dd, J = 5.2, 4.0 Hz, 1H), 2.52 (dd, J = 5.2, 2.8 Hz, 1H), 2.04 – 1.92 (m, 2H), 1.88 – 1.80 (m, 1H), 1.69 – 1.60 (m, 1H). $^{13}$C NMR (100 MHz, Chloroform-*d*) 161.3, 126.9 (q, $J_{C–F}$ = 3.5 Hz) , 124.4 (q, $J_{C–F}$ = 269.5 Hz) , 122.8 (q, $J_{C–F}$ = 32.5 Hz), 114.4, 67.5, 51.8, 47.0, 29.0, 25.7. $^{19}$F NMR (375 MHz, Chloroform-d) δ -61.46. HR-MS (ESI) m/z calc. for $C_{12}H_{14}F_3O_2$ $[M+H]^+$: 247.0940, found: 247.0941.

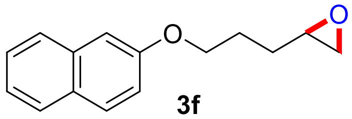


**3f**

**2-(3-(Naphthalen-2-yloxy)propyl)oxirane**

Compound **3f** was prepared following the general procedure, purification by column chromatography on silica gel (petroleum ether/EtOAc = 50:1) yielded **3f** (21.4 mg, 31%) as a white solid. M.p.: 56–57 °C. $^1$H NMR (400 MHz, Chloroform-*d*) δ 7.78 – 7.71 (m, 3H), 7.46 – 7.42 (m, 1H), 7.35 – 7.31 (m, 1H), 7.16 – 7.13 (m, 2H), 4.19 – 4.09 (m, 2H), 3.06 – 3.01 (m, 1H), 2.80 (dd, J = 5.2, 3.6 Hz, 1H), 2.54 (dd, J = 5.2, 2.8 Hz, 1H), 2.07 – 1.99 (m, 2H), 1.91 – 1.83 (m, 1H), 1.76 – 1.67 (m, 1H). $^{13}$C NMR (100 MHz, Chloroform-*d*) δ 156.9, 134.6, 129.4, 129.0, 127.6, 126.7, 126.3, 123.6, 118.9, 106.6, 67.3, 52.0, 47.1, 29.2, 25.8. HR-MS (ESI) m/z calc. for $C_{15}H_{17}O_2$ $[M+H]^+$: 229.1223, found: 229.1219.

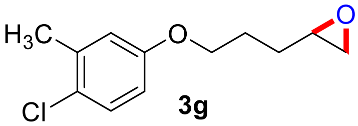


**3g**

**2-(3-(4-Chloro-3-methylphenoxy)propyl)oxirane**

Compound **3g** was prepared following the general procedure, purification by column chromatography on silica gel (petroleum ether/EtOAc = 50:1) yielded **3g** (39.4 mg, 58%) as a colorless oil. $^1$H NMR (400 MHz, Chloroform-*d*) δ 7.20 (d, J = 8.4 Hz, 1H), 6.76 (d, J = 2.8 Hz, 1H), 6.66 (dd, J = 8.8, 2.8 Hz, 1H), 4.02 – 3.92 (m, 2H), 3.01 – 2.96 (m, 1H), 2.79 – 2.77 (m, 1H), 2.51 (dd, J = 5.2, 2.8 Hz, 1H), 2.33 (s, 3H), 1.98 – 1.89

(m, 2H), 1.85 – 1.76 (m, 1H), 1.68 – 1.63 (m, 1H). $^{13}$C NMR (100 MHz, Chloroform-*d*) δ 157.4, 136.9, 129.5, 125.8, 117.0, 113.0, 67.5, 51.9, 47.0, 29.1, 25.8, 20.3. HR-MS (ESI) m/z calc. for $C_{12}H_{16}ClO_2$ [M+H]$^+$: 227.0833, found: 227.0828.

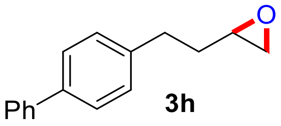

**3h**

**2-(2-([1,1'-Biphenyl]-4-yl)ethyl)oxirane**

Compound **3h** was prepared following the general procedure, purification by column chromatography on silica gel (petroleum ether/EtOAc = 50:1) yielded **3h** (44.5 mg, 66%) as a colorless oil. $^1$H NMR (400 MHz, Chloroform-*d*) δ 7.62 – 7.60 (m, 2H), 7.57 – 7.54 (m, 2H), 7.47 – 7.43 (m, 2H), 7.37 – 7.33 (m, 1H), 7.32 – 7.30 (m, 2H), 3.03 – 2.99 (m, 1H), 2.93 – 2.78 (m, 3H), 2.52 (dd, J = 5.2, 2.8 Hz, 1H), 2.00 – 1.85 (m, 2H). $^{13}$C NMR (100 MHz, Chloroform-*d*) δ 140.9, 140.3, 139.0, 128.8, 128.7, 127.1, 127.0, 126.9, 51.7, 47.2, 34.2, 31.8. HR-MS (ESI) m/z calc. for $C_{16}H_{17}O$ [M+H]$^+$: 225.1274, found: 225.1269.

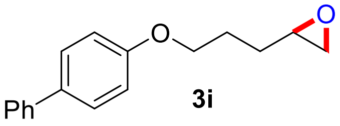

**3i**

**2-(3-([1,1'-Biphenyl]-4-yloxy)propyl)oxirane**

Compound **3i** was prepared following the general procedure, purification by column chromatography on silica gel (petroleum ether/EtOAc = 50:1) yielded **3i** (65.6mg, 86%) as a white solid. M.p.: 58–59 °C. $^1$H NMR (400 MHz, Chloroform-*d*) δ 7.58 – 7.52 (m, 4H), 7.45 – 7.41 (m, 2H), 7.33 – 7.29 (m, 1H), 7.00 – 6.96 (m, 2H), 4.12 – 4.02 (m, 2H), 3.04 – 3.00 (m, 1H), 2.80 (dd, J = 4.8, 4.0 Hz, 1H), 2.54 (dd, J = 4.8, 2.8 Hz, 1H), 2.04 – 1.95 (m, 2H), 1.89 – 1.81 (m, 1H), 1.74 – 1.65 (m, 1H) $^{13}$C NMR (100 MHz, Chloroform-*d*) δ 158.4, 140.8, 133.7, 128.7, 128.1, 126.7, 126.6, 114.7, 67.4, 51.9, 47.0, 29.2, 25.8. HR-MS (ESI) m/z calc. for $C_{17}H_{19}O_2$ [M+H]$^+$: 255.1380, found: 255.1377.

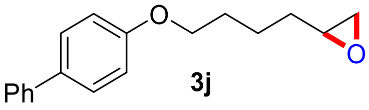

**3j**

**2-(4-([1,1'-Biphenyl]-4-yloxy)butyl)oxirane**

Compound **3j** was prepared following the general procedure, purification by column chromatography on silica gel (petroleum ether/EtOAc = 50:1) yielded **3j** (57.8 mg, 72%) as a white solid. M.p.: 66–67 °C. $^{1}$H NMR (400 MHz, Chloroform-*d*) δ 7.59 – 7.52 (m, 4H), 7.45 – 7.41 (m, 2H), 7.32 (t, J = 7.2 Hz, 1H), 7.00 – 6.97 (m, 2H), 4.03 (t, J = 6.4 Hz, 2H), 2.99 – 2.95 (m, 1H), 2.79 (dd, J = 5.2, 4.0 Hz, 1H), 2.52 (dd, J = 5.2, 2.8 Hz, 1H), 1.93 – 1.86 (m, 2H), 1.72 – 1.60 (m, 4H). $^{13}$C NMR (100 MHz, Chloroform-*d*) δ 158.6, 140.8, 133.6, 128.7, 128.2, 128.0, 126.1, 114.7, 67.6, 52.1, 47.1, 32.2, 29.1, 22.7. HR-MS (ESI) m/z calc. for $C_{18}H_{21}O_2$ [M+H]$^+$: 269.1536, found: 269.1529.

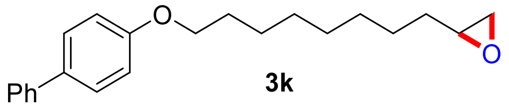


**3k**

**2-(8-([1,1'-Biphenyl]-4-yloxy)octyl)oxirane**

Compound **3k** was prepared following the general procedure, purification by column chromatography on silica gel (petroleum ether/EtOAc = 50:1) yielded **3k** (65.1mg, 67%) as a white solid. M.p.: 67–68 °C. $^{1}$H NMR (400 MHz, Chloroform-*d*) δ 7.57 – 7.51 (m, 4H), 7.44 – 7.40 (m, 2H), 7.32 – 7.29 (m, 1H), 7.00 – 6.96 (m, 2H), 4.00 (t, J = 6.4 Hz, 2H), 2.94 – 2.90 (m, 1H), 2.76 (dd, J = 4.8, 4.0 Hz, 1H), 2.48 (dd, J = 5.2, 2.8 Hz, 1H), 1.85 – 1.78 (m, 2H), 1.56 – 1.47 (m, 6H), 1.39 – 1.37 (m, 6H). $^{13}$C NMR (100 MHz, Chloroform-*d*) δ 158.7, 140.9, 133.5, 128.7, 128.1, 126.7, 126.6, 114.7, 68.0, 52.4, 47.1, 32.5, 29.5, 29.4, 29.3, 26.0, 25.9. HR-MS (ESI) m/z calc. for $C_{22}H_{29}O_2$ [M+H]$^+$: 325.2162, found: 325.2159.

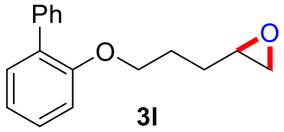


**3l**

**2-(3-([1,1'-Biphenyl]-2-yloxy)propyl)oxirane**

Compound **3l** was prepared following the general procedure, purification by column chromatography on silica gel (petroleum ether/EtOAc = 50:1) yielded **3l** (39.6mg, 52%) as a colorless oil. $^{1}$H NMR (400 MHz, Chloroform-*d*) δ 7.55 – 7.52 (m, 2H), 7.42 – 7.38 (m, 2H), 7.35 – 7.29 (m, 3H), 7.06 – 7.02 (m, 1H), 6.98 (dd, J = 8.0, 1.2 Hz, 1H), 4.07 – 3.97 (m, 2H), 2.91 – 2.86 (m, 1H), 2.68 (dd, J = 4.8, 4.0 Hz, 1H), 2.41 (dd, J = 4.8, 2.4 Hz, 1H), 1.95 – 1.82 (m, 2H), 1.68 – 1.56 (m, 2H). $^{13}$C NMR (100 MHz,

Chloroform-*d*) δ 155.8, 138.5, 131.0, 130.9, 129.6, 128.5, 127.8, 126.8, 121.0, 112.5, 67.9, 51.9, 47.1, 29.2, 25.7. HR-MS (ESI) m/z calc. for $C_{17}H_{19}O_2$ $[M+H]^+$: 255.1380, found: 255.1381.

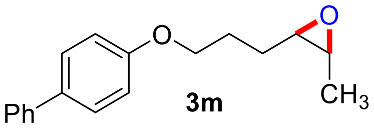

**3m**

**2-Methyl-3-(3-phenoxypropyl)oxirane**

Compound **3m** was prepared following the general procedure, purification by column chromatography on silica gel (petroleum ether/EtOAc = 50:1) yielded **3m** (62.7 mg, 78%) as a white solid. M.p.: 57–58 °C. $^1$H NMR (400 MHz, Chloroform-*d*) δ 7.58 – 7.52 (m, 4H), 7.45 – 7.41 (m, 2H), 7.34 – 7.30 (m, 1H), 7.00 – 6.97 (m, 2H), 4.10 – 4.01 (m, 2H), 2.84 – 2.80 (m, 1H), 2.76 – 2.73 (m, 1H), 2.04 – 1.91 (m, 2H), 1.87 – 1.79 (m, 1H), 1.72 – 1.64 (m, 1H), 1.33 (d, J = 5.2 Hz, 3H). $^{13}$C NMR (100 MHz, Chloroform-*d*) δ 158.5, 140.8, 128.7, 128.1, 126.7, 126.6, 114.7, 67.4, 59.3, 54.5, 28.7, 25.8, 17.6. HR-MS (ESI) m/z calc. for $C_{18}H_{21}O_2$ $[M+H]^+$: 269.1536, found: 269.1533.

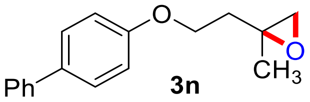

**3n**

**2-(2-([1,1'-Biphenyl]-4-yloxy)ethyl)-2-methyloxirane**

Compound **3n** was prepared following the general procedure, purification by column chromatography on silica gel (petroleum ether/EtOAc = 50:1) yielded **3n** (57.2 mg, 75%) as a white solid. M.p.: 63–64 °C. $^1$H NMR (400 MHz, Chloroform-*d*) δ 7.58 – 7.53 (m, 4H), 7.45 – 7.41 (m, 2H), 7.34 – 7.30 (m, 1H), 7.00 – 6.96 (m, 2H), 4.16 – 4.07 (m, 2H), 2.78 (d, J = 4.8 Hz, 1H), 2.67 (d, J = 4.8 Hz, 1H), 2.19 – 2.03 (m, 2H), 1.44 (s, 3H). $^{13}$C NMR (100 MHz, Chloroform-*d*) δ 158.2, 140.7, 133.9, 128.7, 128.1, 126.7, 126.6, 114.7, 64.3, 55.2, 54.0, 36.0, 21.6. HR-MS (ESI) m/z calc. for $C_{17}H_{19}O_2$ $[M+H]^+$: 255.1380, found: 255.1378.

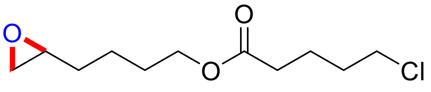

**3o**

**4-(Oxiran-2-yl)butyl 5-chloropentanoate**

Compound **3o** was prepared following the general procedure, purification by column chromatography on silica gel (petroleum ether/EtOAc = 50:1) yielded **3o** (26.4 mg, 45%) as a colorless oil. $^1$H NMR (400 MHz, Chloroform-*d*) δ 4.09 (t, J = 6.4 Hz, 2H), 3.54 (t, J = 6.4 Hz, 2H), 2.93 – 2.89 (m, 1H), 2.75 (dd, J = 5.2, 4.0 Hz, 1H), 2.47 (dd, J = 5.2, 2.4 Hz, 1H), 2.34 (t, J = 7.2 Hz, 2H), 1.85 – 1.75 (m, 4H), 1.73 – 1.66 (m, 2H), 1.63 – 1.55 (m, 2H), 1.54 – 1.50 (m, 2H). $^{13}$C NMR (100 MHz, Chloroform-*d*) δ 173.2, 64.2, 52.1, 47.0, 44.5, 33.4, 32.0, 31.9, 28.4, 22.5, 22.3. HR-MS (ESI) m/z calc. for $C_{11}H_{20}ClO_3$ $[M+H]^+$: 235.1095, found: 235.1096.

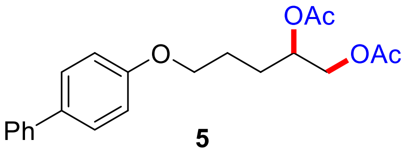


**5**

**5-([1,1'-Biphenyl]-4-yloxy)pentane-1,2-diyl diacetate**

Compound **5** was prepared following the general procedure, purification by column chromatography on silica gel (petroleum ether/EtOAc = 10:1) yielded **5** (97.2 mg, 91%) as a white soild. $^1$H NMR (400 MHz, Chloroform-*d*) δ 7.58 – 7.50 (m, 4H), 7.42 (t, J = 7.7 Hz, 2H), 7.31 (t, J = 7.3 Hz, 1H), 6.99 – 6.93 (m, 2H), 5.19 (td, J = 6.4, 3.4 Hz, 1H), 4.28 (dd, J = 11.9, 3.4 Hz, 1H), 4.09 (dd, J = 11.9, 6.4 Hz, 1H), 4.05 – 3.96 (m, 2H), 2.09 (s, 3H), 2.08 (s, 3H), 1.91 – 1.78 (m, 4H).$^{13}$C NMR (100 MHz, Chloroform-*d*) δ 170.7, 170.5, 158.3, 140.7, 133.7, 128.6, 128.1, 126.7, 126.6, 114.6, 71.1, 67.2, 64.9, 27.4, 25.0, 21.0, 20.7. HR-MS (ESI) m/z calc. for $C_{21}H_{25}O_5$ $[M+H]^+$: 357.1697, found: 357.1707.

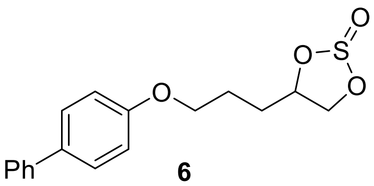


**6**

**4-(3-([1,1'-Biphenyl]-4-yloxy)propyl)-1,3,2-dioxathiolane 2-oxide**

Compound **6** was prepared following the general procedure, purification by column chromatography on silica gel (petroleum ether/EtOAc = 50:1) yielded **6** (65.0 mg, 68%) as a colorless oil. $^1$H NMR (400 MHz, Chloroform-*d*) δ 7.56 – 7.51 (m, 4H), 7.42 (t, J = 7.7 Hz, 2H), 7.31 (t, J = 7.3 Hz, 1H), 7.00 – 6.91 (m, 2H), 4.66 – 4.38 (m, 2H), 4.40 (t, J = 8.8 Hz, 1H), 4.12 – 4.02 (m, 2H), 2.17 – 2.04 (m, 3H), 1.97 (dt, J = 13.9, 6.6 Hz,

1H). $^{13}C$ NMR (100 MHz, Chloroform-*d*) δ 158.2, 140.7, 134.0, 128.7, 128.2, 126.7, 114.7, 83.6, 70.2, 67.0, 30.3, 25.8. HR-MS (ESI) m/z calc. for $C_{17}H_{19}O_4S$ $[M+H]^+$: 319.0999, found: 319.0995.

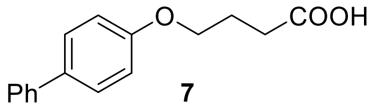


**7**

**4-([1,1'-Biphenyl]-4-yloxy)butanoic acid**

Compound **6** was prepared following the general procedure, purification by column chromatography on silica gel (petroleum ether/EtOAc = 20:1) yielded **7** (66.4 mg, 52%) as a white solid. M.p.: 43–44 °C. $^1H$ NMR (400 MHz, Chloroform-*d*) δ 9.86 (s, 1H), 7.58 – 7.48 (m, 4H), 7.42 (t, J = 7.6 Hz, 2H), 7.30 (t, J = 7.4 Hz, 1H), 6.95 (d, J = 8.7 Hz, 2H), 4.05 (t, J = 6.0 Hz, 2H), 2.72 – 2.68 (m, 2H), 2.15 (p, J = 6.6 Hz, 2H). $^{13}C$ NMR (100 MHz, Chloroform-*d*) δ 201.7, 158.2, 140.7, 134.0, 128.7, 128.2, 126.7, 126.7, 114.7, 66.7, 40.7, 22.0. HR-MS (ESI) m/z calc. for $C_{16}H_{17}O_3$ $[M+H]^+$: 255.1274, found: 255.1277.

**X-ray Crystallography of 2y**

Compound **2y** (25 mg) was dissolved in 6 mL of ether/n-hexane (v1/v2 = 1:2), and it was crystallized to give crystal as yellow prisms after the solvent was slowly volatilized in 7 days at room temperature (~ 28 °C).

All diffraction data were obtained on a Bruker Smart Apex CCD diffractometer equipped with graphite-monochromated Mo Kα radiation. CCDC-2286663 (**2y**), contain the supplementary crystallographic data. These data can be obtained free of charge from the Cambridge Crystallographic Data Centre (http://www.ccdc.cam.ac.uk/). X-ray crystallographic data for **2y** is available as **Table S7**.

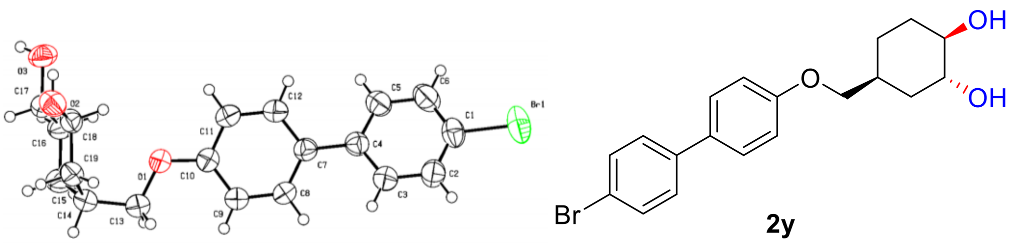


**2y**

**Supplementary Figure 13** The molecular structure of **2y**

**Table S7**. Crystal data and structure refinement for **2y**

| | |
|---|---|
| Empirical formula | $C_{19}H_{21}BrO_3$ |
| Formula weight | 377.27 |
| Temperature/K | 293 |
| Crystal system, Space group | monoclinic, P21/c |
| Unit cell dimensions | a/Å 24.4551(9) α/° 90 |
| | b/Å 8.2654(4) β/° 90.774(3) |
| | c/Å 8.4350(3) γ/° 90 |
| Volume/Å$^3$ | 1704.82(12) |
| Z | 4 |
| $\rho_{calc}$g/cm$^3$ | 1.470 |
| μ/mm$^{-1}$ | 3.387 |
| F(000) | 776.0 |
| Crystal size/mm$^3$ | 0.07 × 0.06 × 0.05 |
| Radiation | CuKα (λ = 1.54184) |
| 2Θ range for data collection/° | 3.614 to 136.82 |
| Index ranges | $-28 \le h \le 20, -9 \le k \le 8, -10 \le l \le 8$ |
| Reflections collected | 8664 |
| Independent reflections | 3055 [$R_{int}$ = 0.0245, $R_{sigma}$ = 0.0254] |
| Data/restraints/parameters | 3055/0/211 |
| Goodness-of-fit on F$^2$ | 1.068 |
| Final R indexes [I>=2σ (I)] | $R_1$ = 0.0321, w$R_2$ = 0.0873 |

| | |
|---|---|
| Final R indexes [all data] | $R_1$ = 0.0372, $wR_2$ = 0.0909 |
| Largest diff. peak/hole / e Å$^{-3}$ | 0.24/-0.39 |

## NMR Spectra

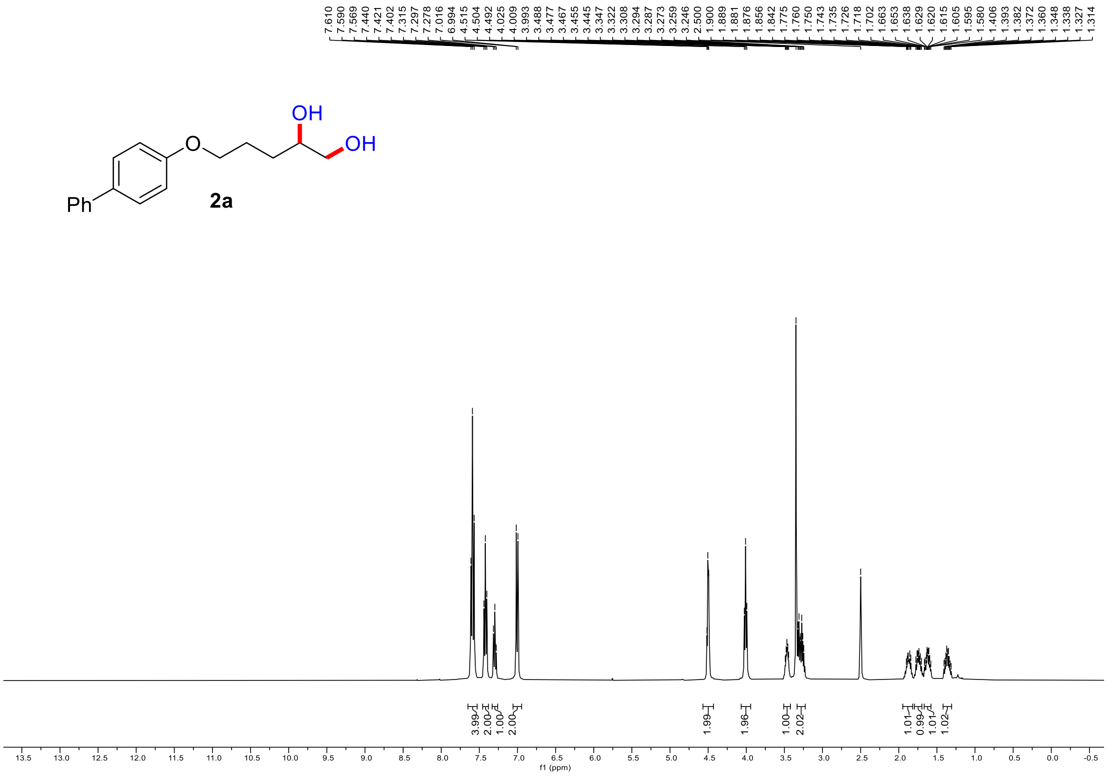

**Supplementary Figure 14**. $^{1}$H NMR of compound **2a** (400 MHz, DMSO-$d_6$)

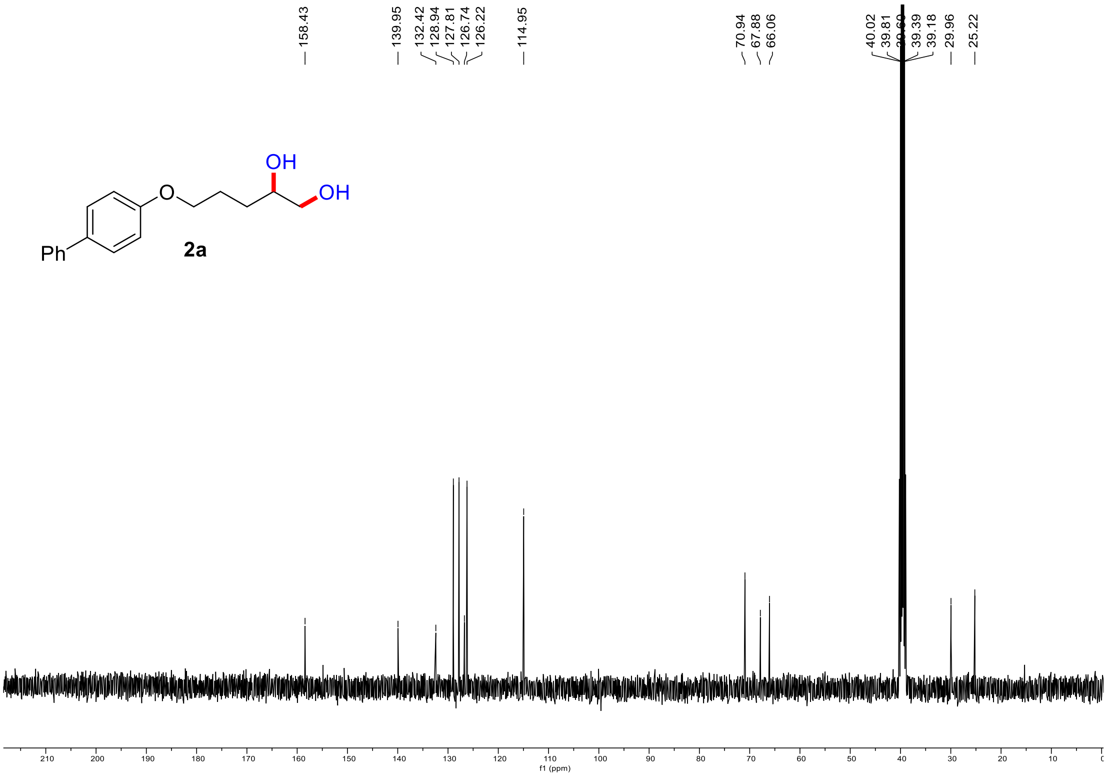

**Supplementary Figure 15**. $^{13}$C NMR of compound **2a** (100 MHz, DMSO-$d_6$)

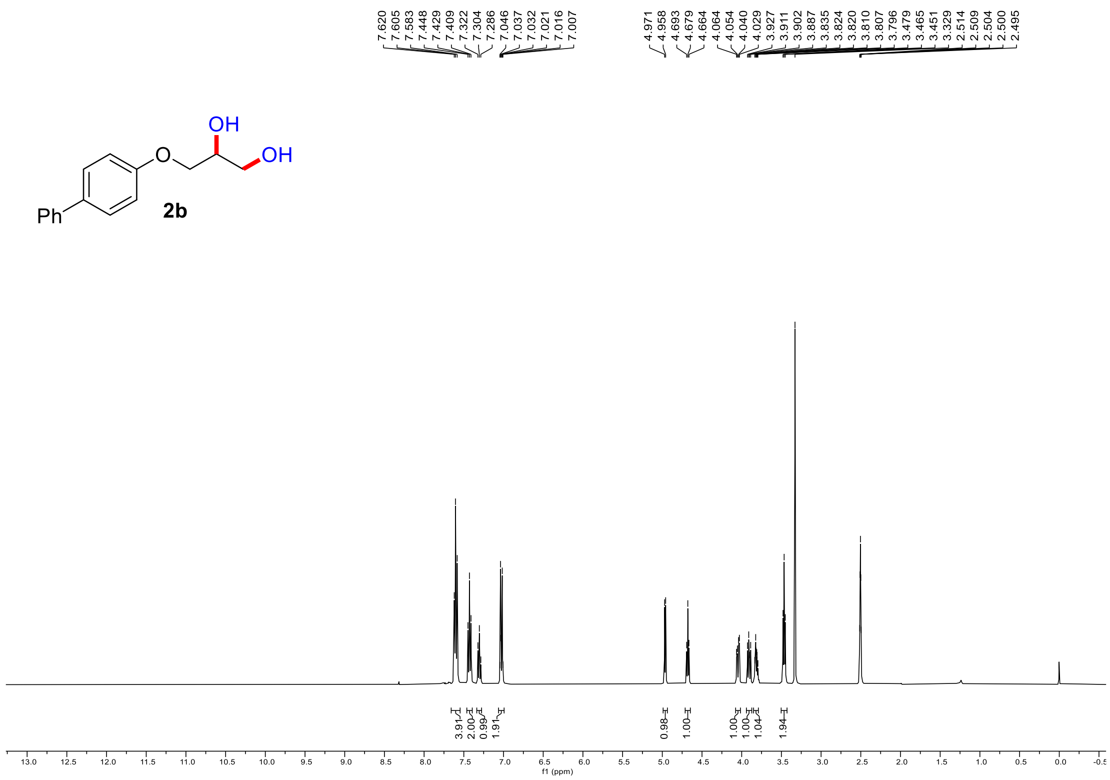

**Supplementary Figure 16**. $^{1}$H NMR of compound **2b** (400 MHz, DMSO-$d_6$)

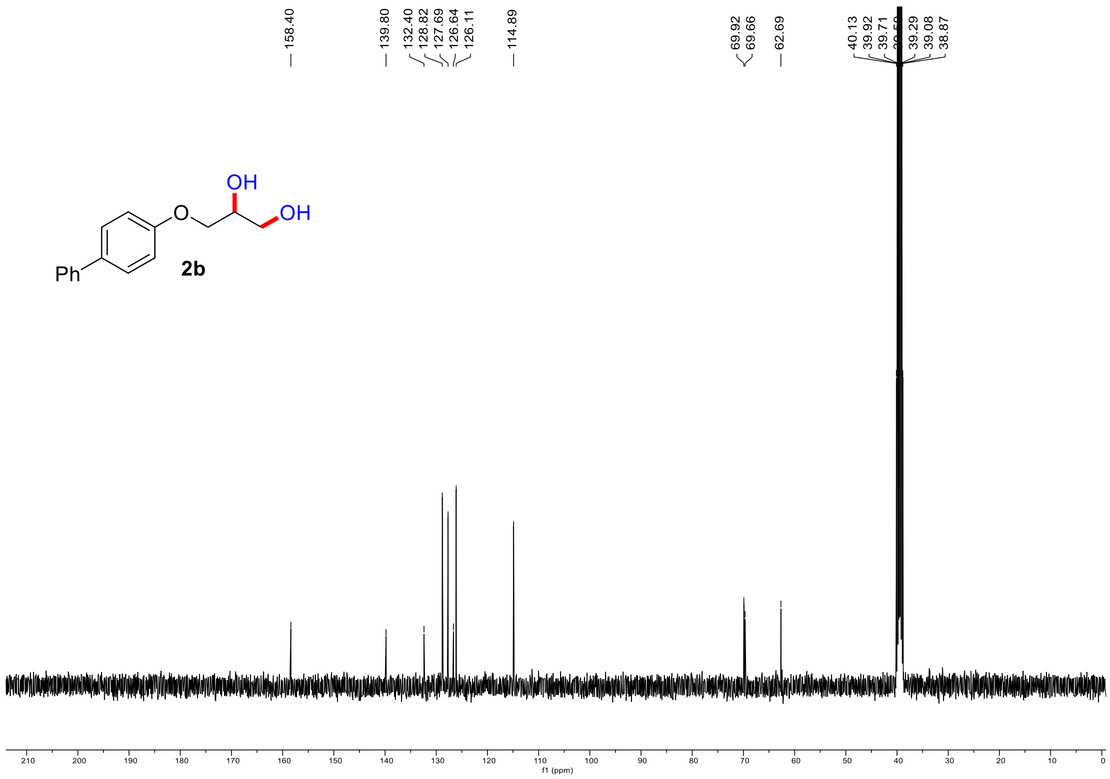

**Supplementary Figure 17**. $^{13}$C NMR of compound **2b** (100 MHz, DMSO-$d_6$)

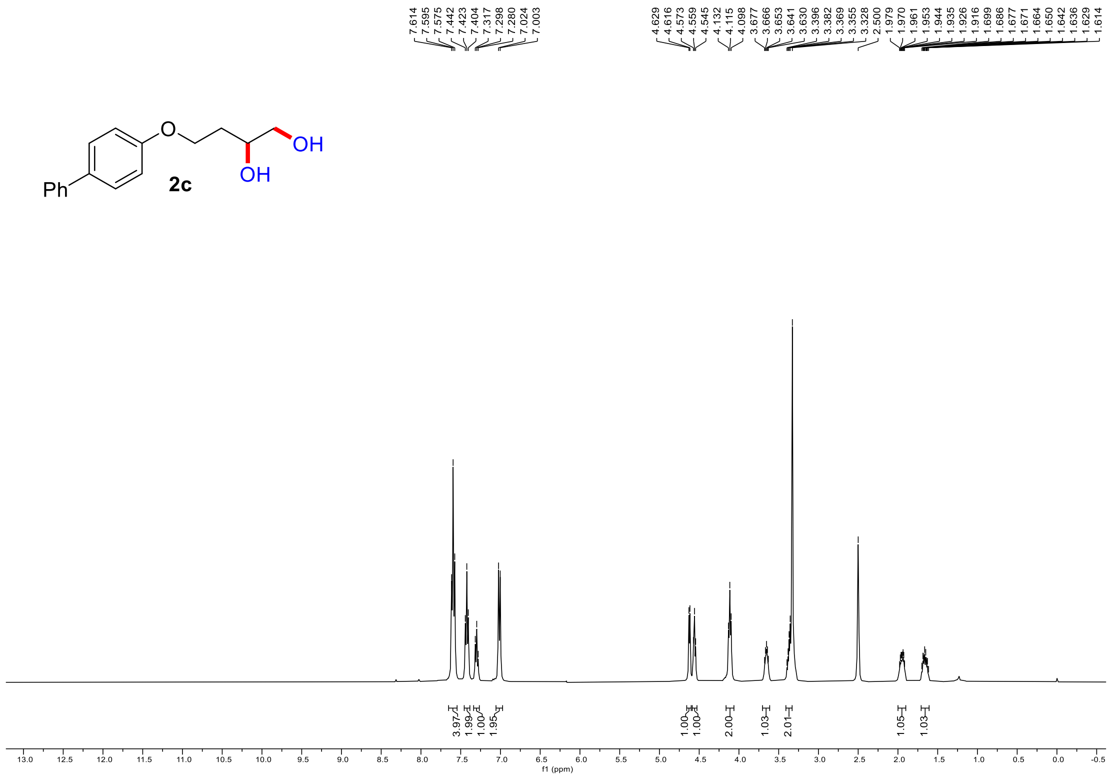

**Supplementary Figure 18**. $^{1}$H NMR of Compound **2c** (400 MHz, DMSO-$d_6$)

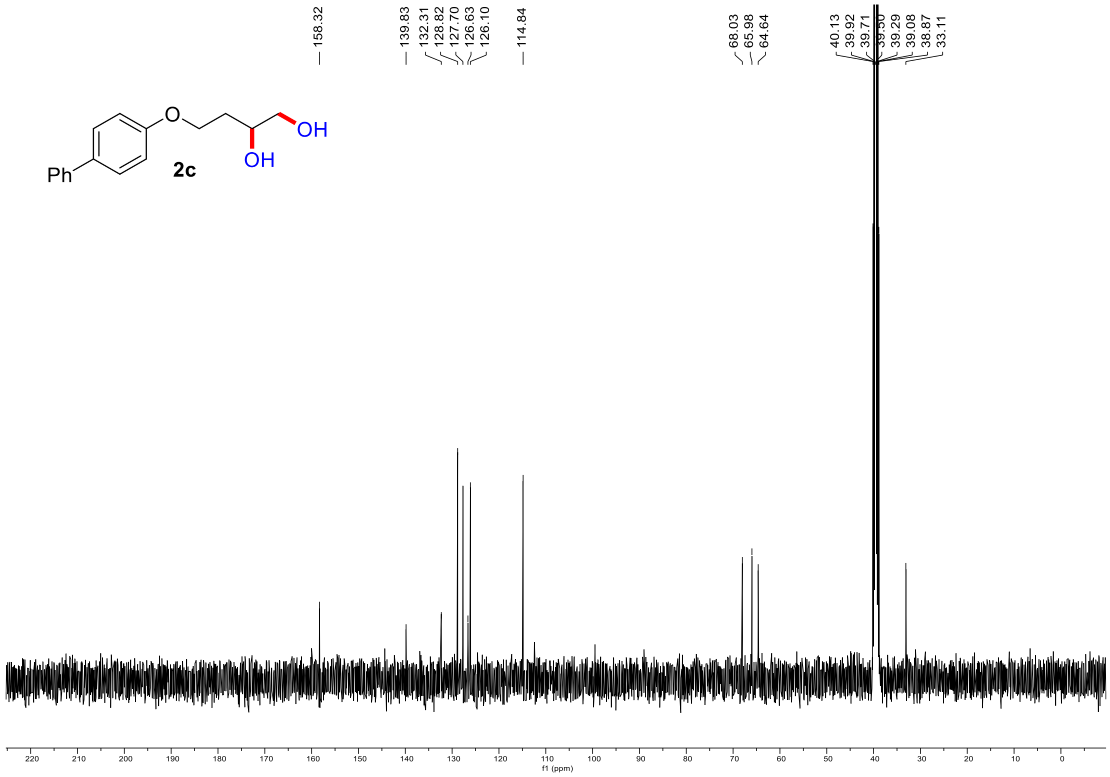

**Supplementary Figure 19**. $^{13}$C NMR of Compound **2c** (100 MHz, DMSO-$d_6$)

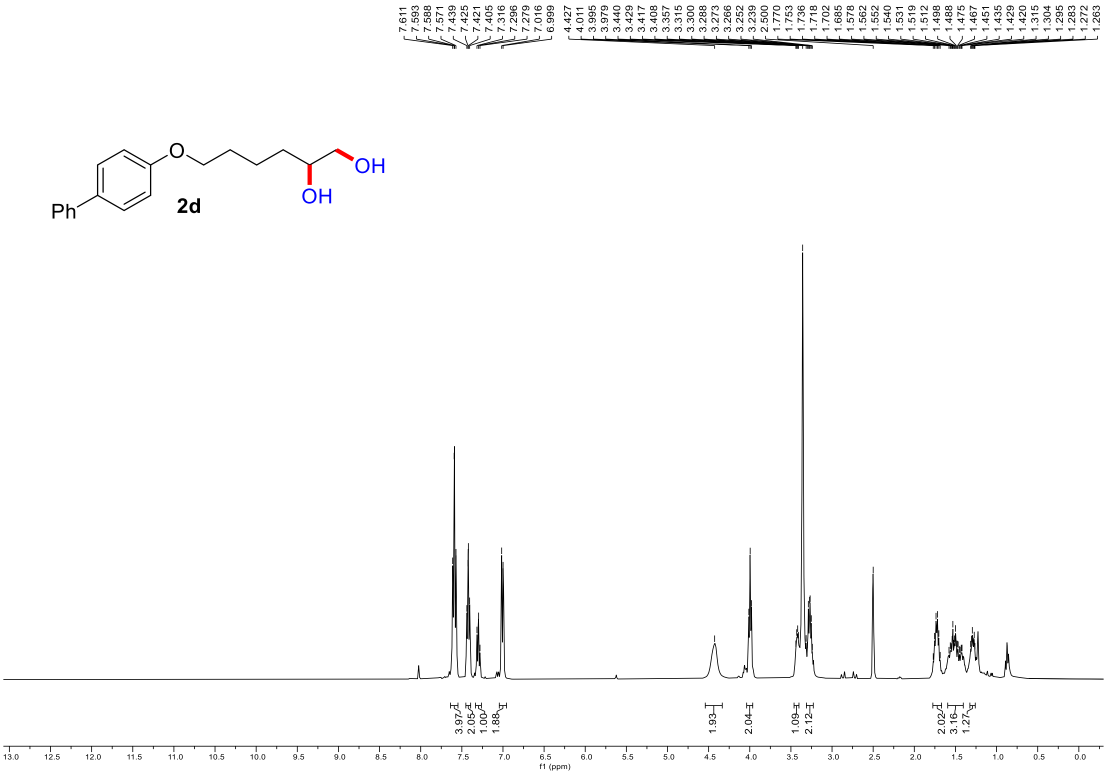

**Supplementary Figure 20**. $^{1}$H NMR of Compound **2d** (400 MHz, DMSO-$d_6$)

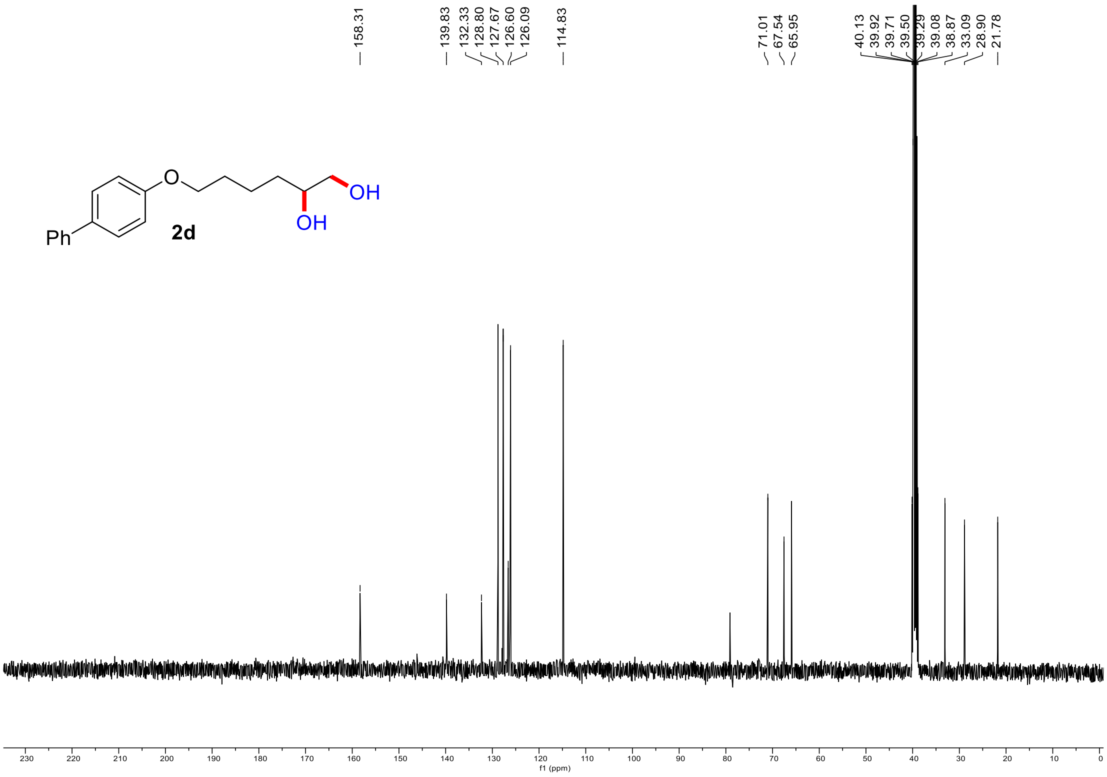

**Supplementary Figure 21**. $^{13}$C NMR of Compound **2d** (100 MHz, DMSO-$d_6$)

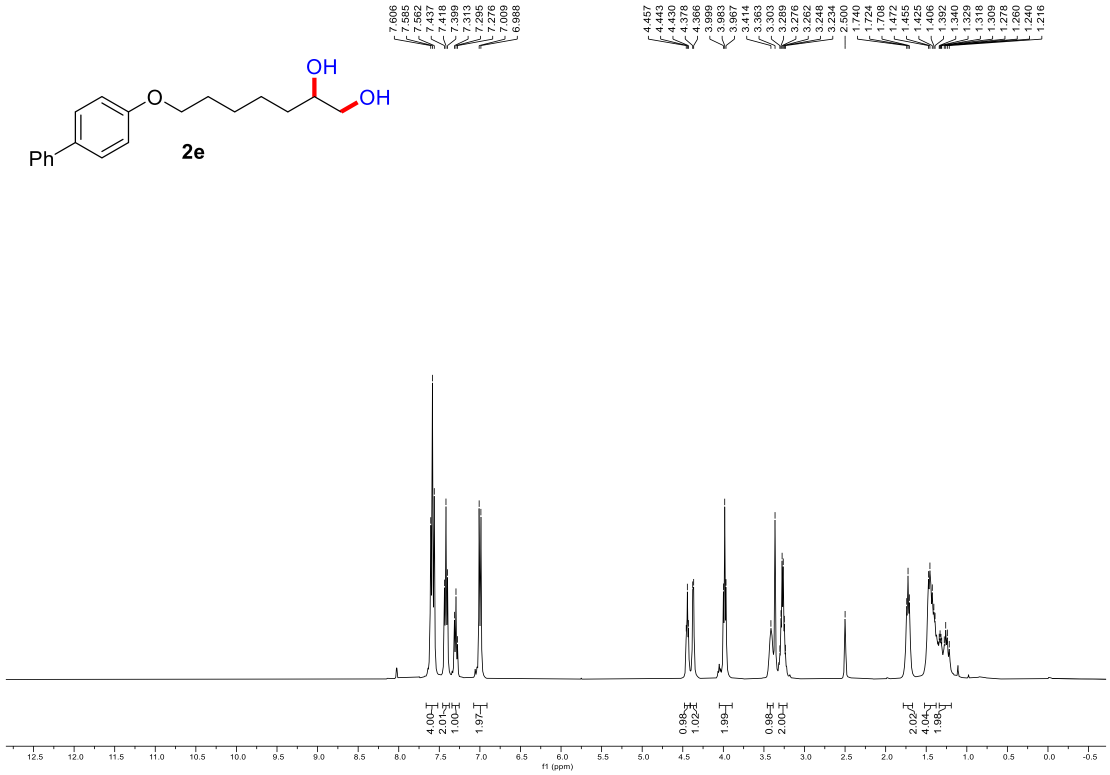


**Supplementary Figure 22**. $^{1}$H NMR of Compound **2e** (400 MHz, DMSO-$d_6$)

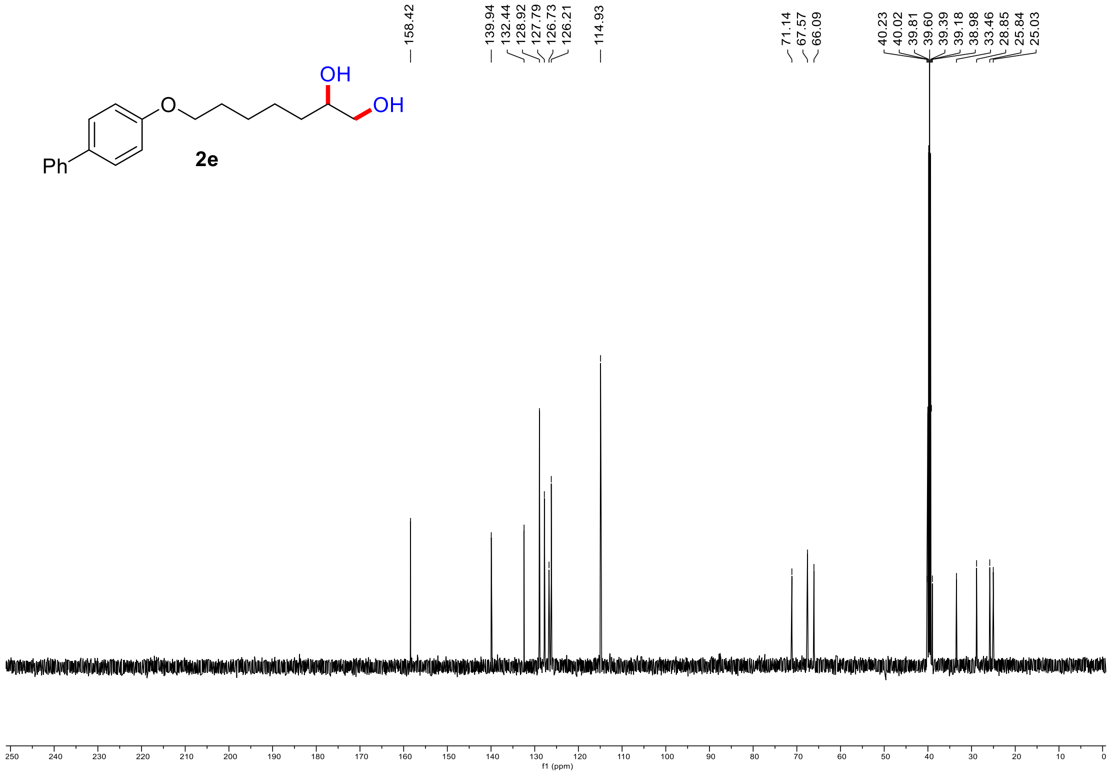


**Supplementary Figure 23**. $^{13}$C NMR of Compound **2e** (100 MHz, DMSO-$d_6$)

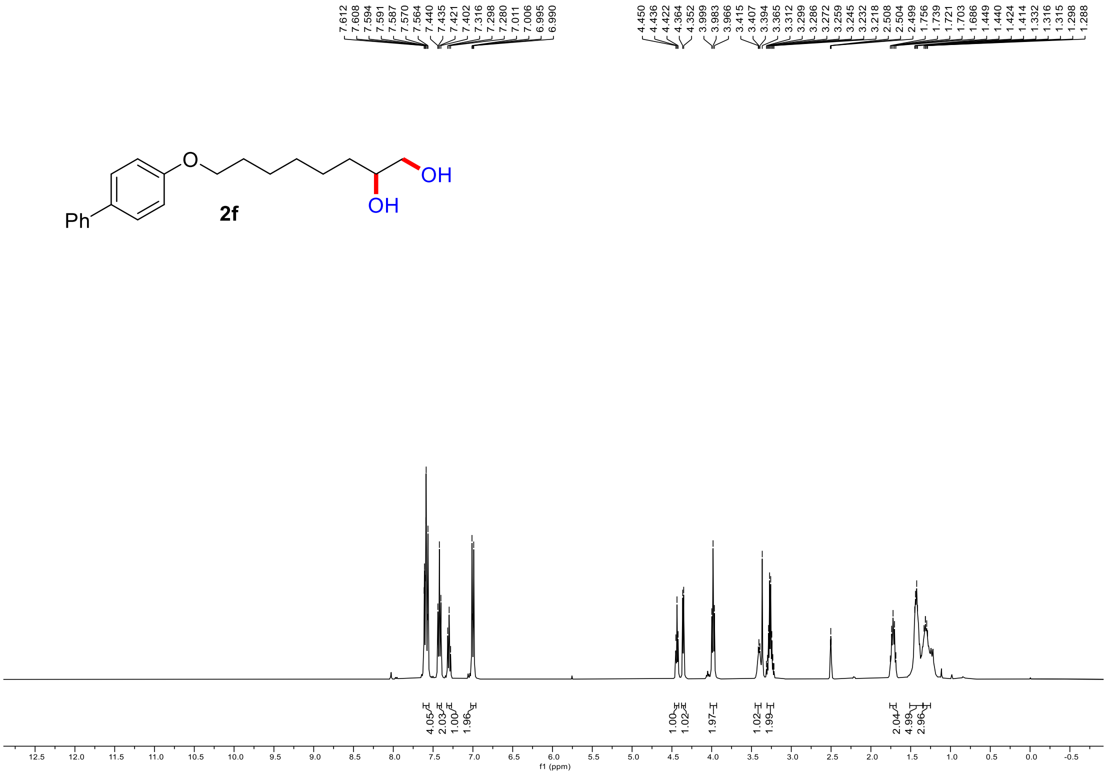

**Supplementary Figure 24**. $^{1}H$ NMR of Compound **2f** (400 MHz, DMSO-$d_6$)

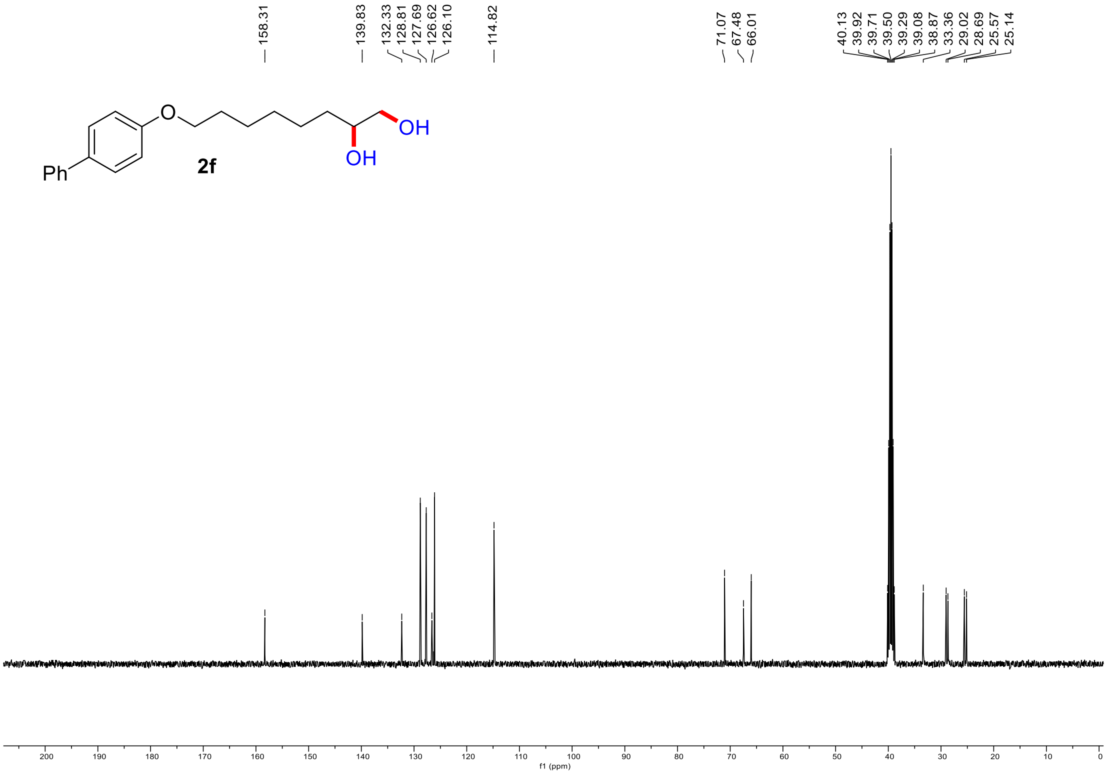

**Supplementary Figure 25**. $^{13}C$ NMR of Compound **2f** (100 MHz, DMSO-$d_6$)

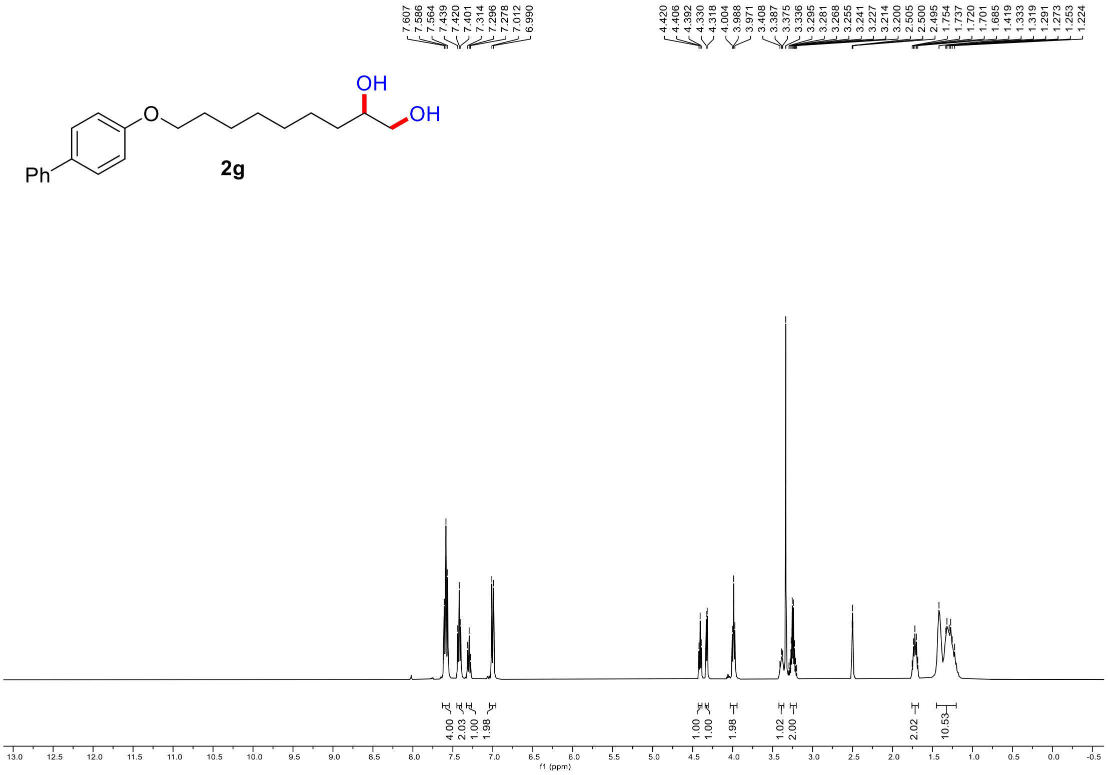

**Supplementary Figure 26**. $^{1}H$ NMR of Compound **2g** (400 MHz, DMSO-$d_6$)

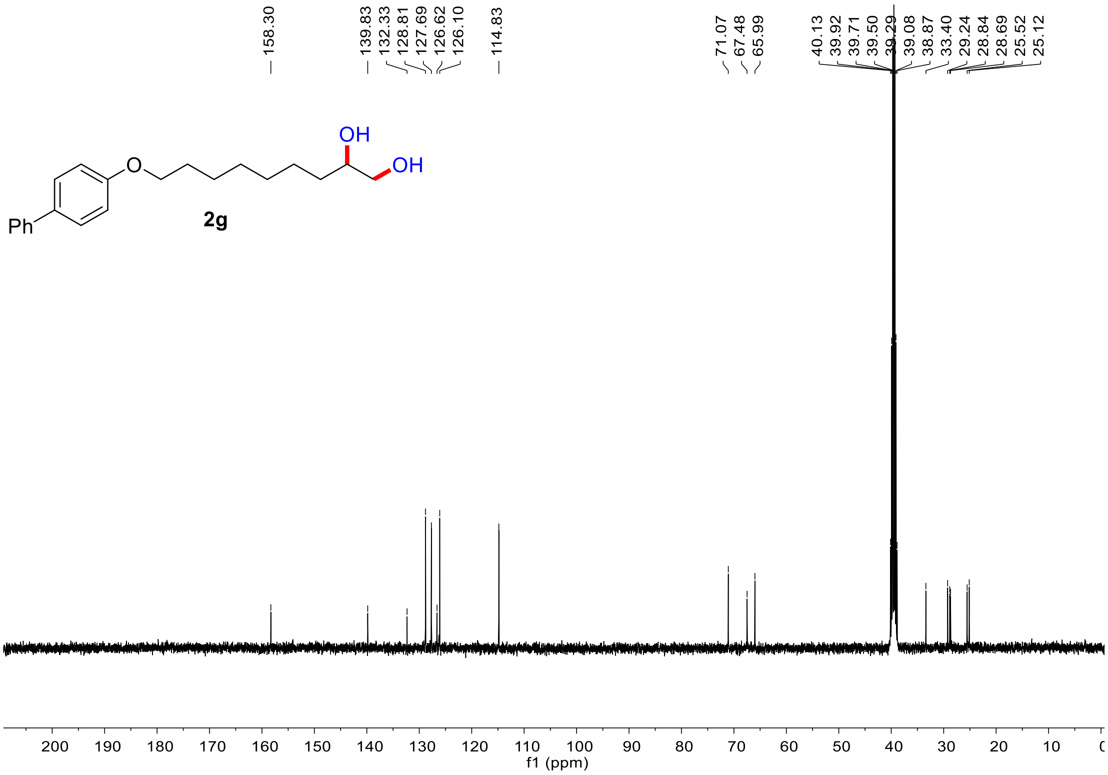

**Supplementary Figure 27**. $^{13}C$ NMR of Compound **2g** (100 MHz, DMSO-$d_6$)

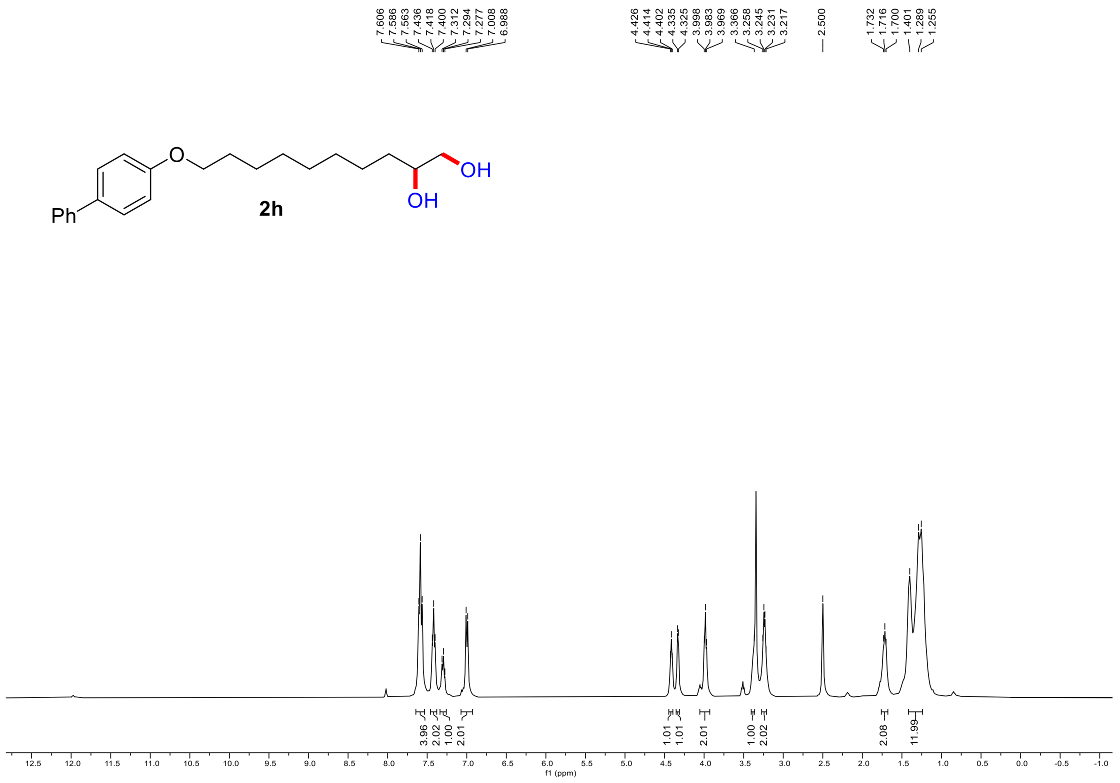


**Supplementary Figure 28**. $^{1}$H NMR of Compound **2h** (400 MHz, DMSO-$d_6$)

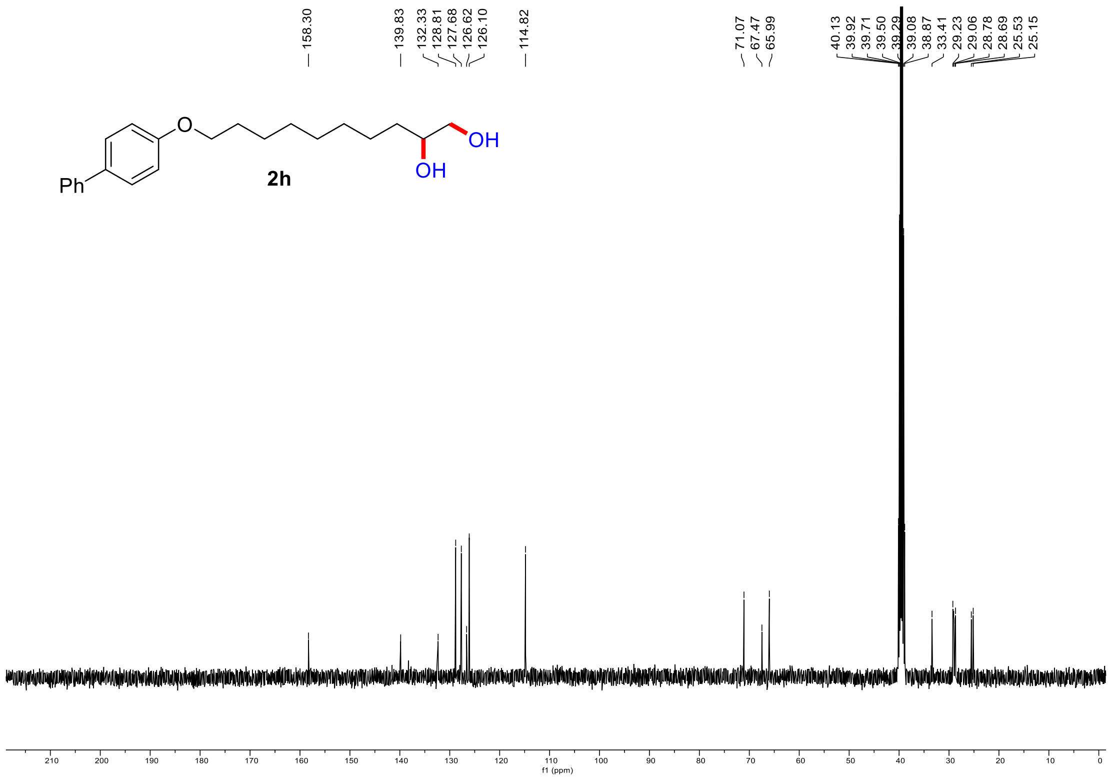


**Supplementary Figure 29**. $^{13}$C NMR of Compound **2h** (100 MHz, DMSO-$d_6$)

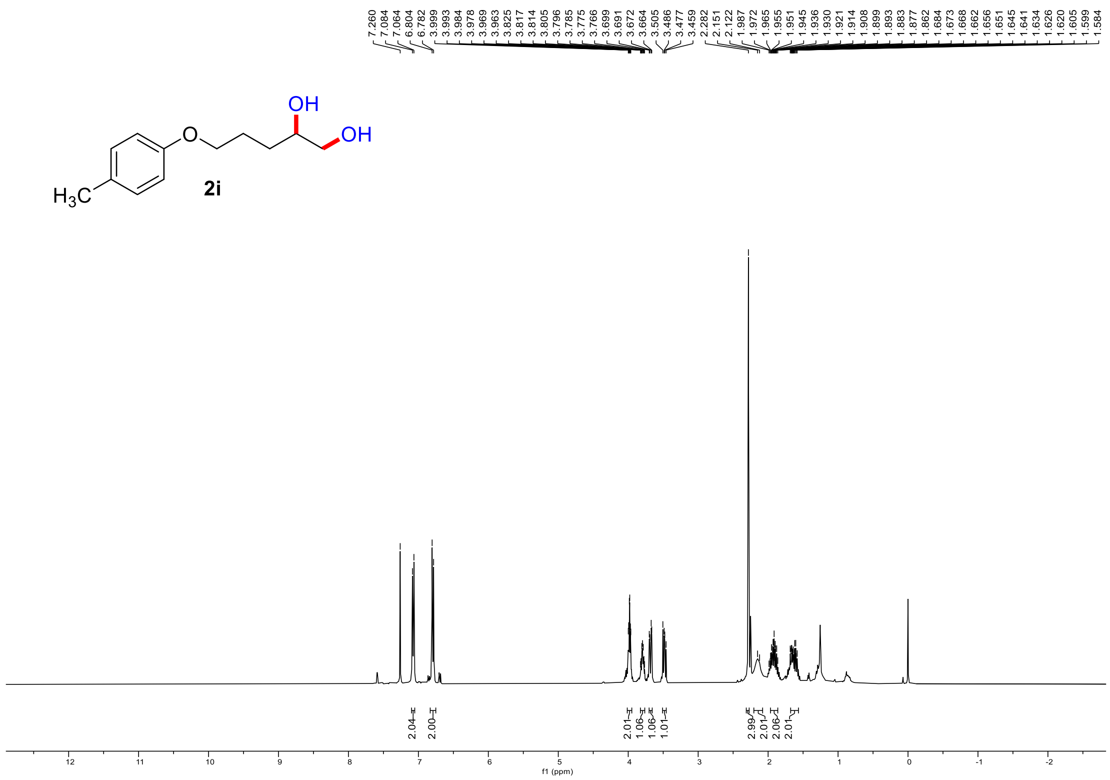

**Supplementary Figure 30**. $^{1}$H NMR of compound **2i** (400 MHz, Chloroform-*d*)

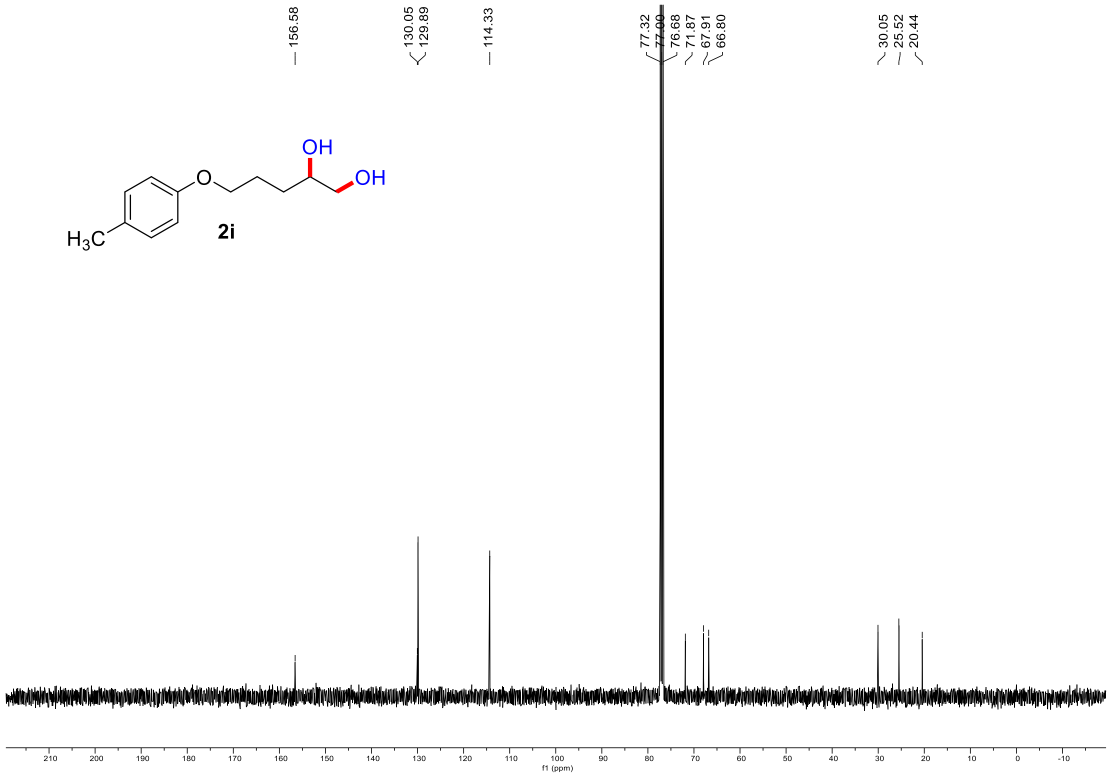

**Supplementary Figure 31**. $^{13}$C NMR of compound **2i** (100 MHz, Chloroform-*d*)

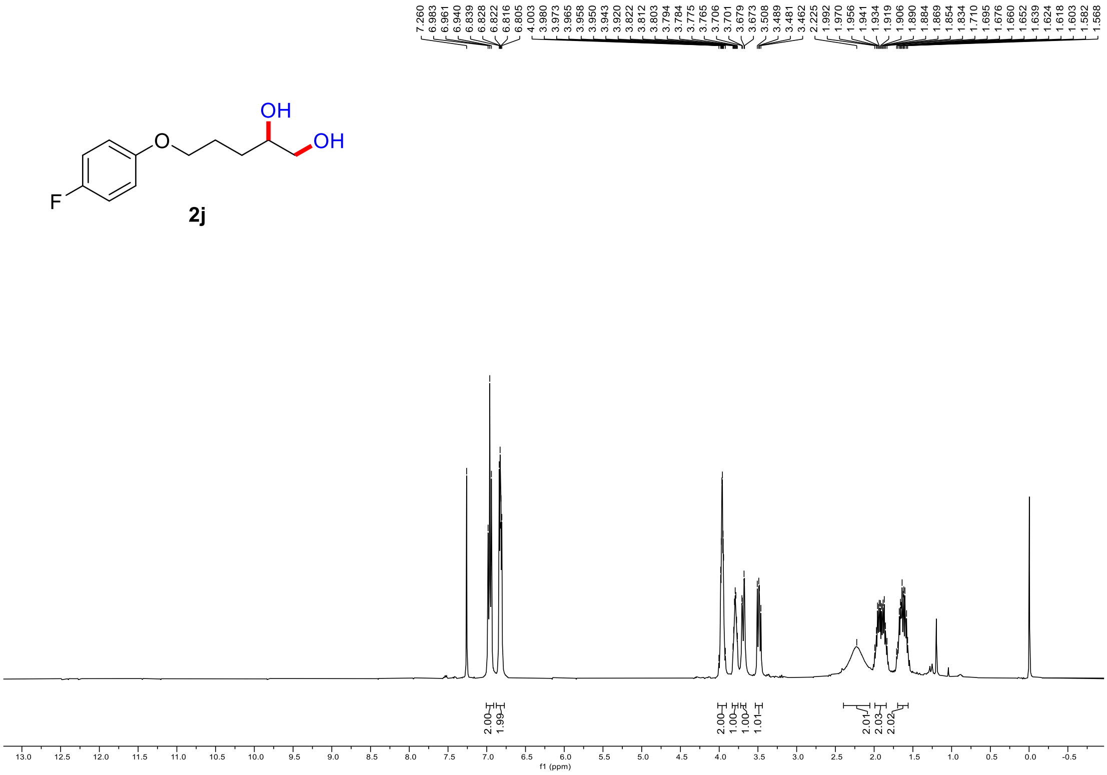


**Supplementary Figure 32**. $^{1}$H NMR of compound **2j** (400 MHz, Chloroform-*d*)

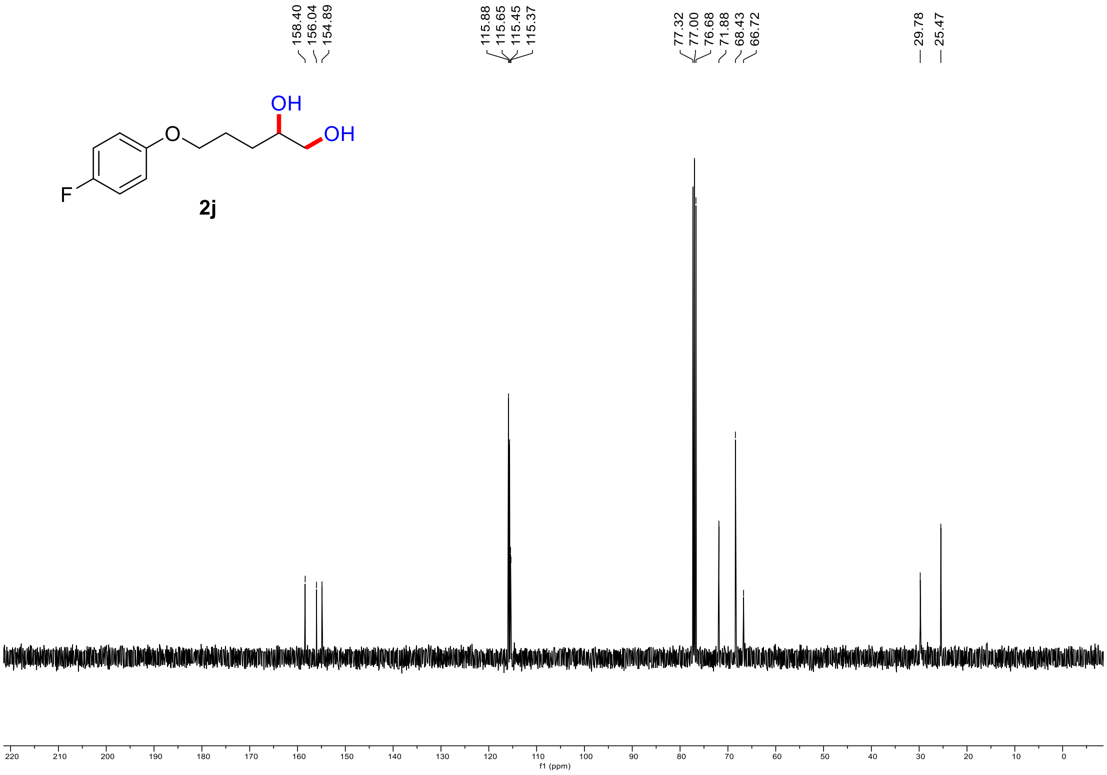


**Supplementary Figure 33**. $^{13}$C NMR of compound **2j** (100 MHz, Chloroform-*d*)

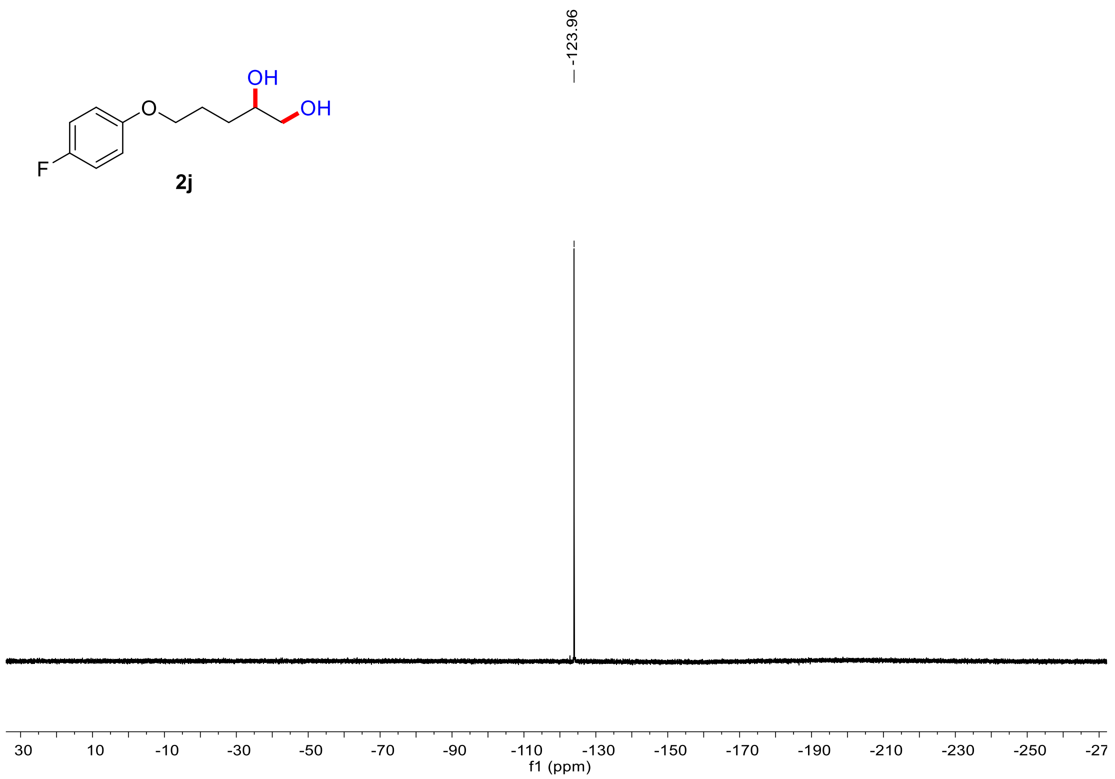


**Supplementary Figure 34**. $^{19}$F NMR of compound **2j** (375 MHz, Chloroform-*d*)

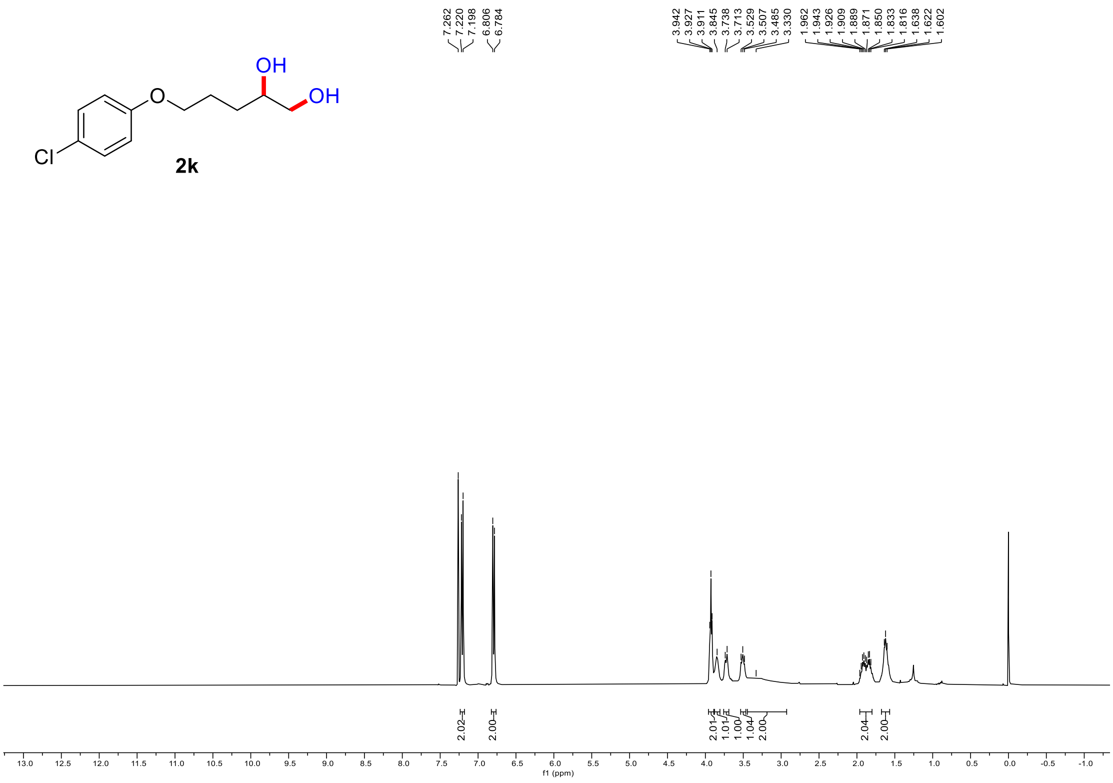


**Supplementary Figure 35**. $^{1}$H NMR of compound **2k** (400 MHz, Chloroform-*d*)

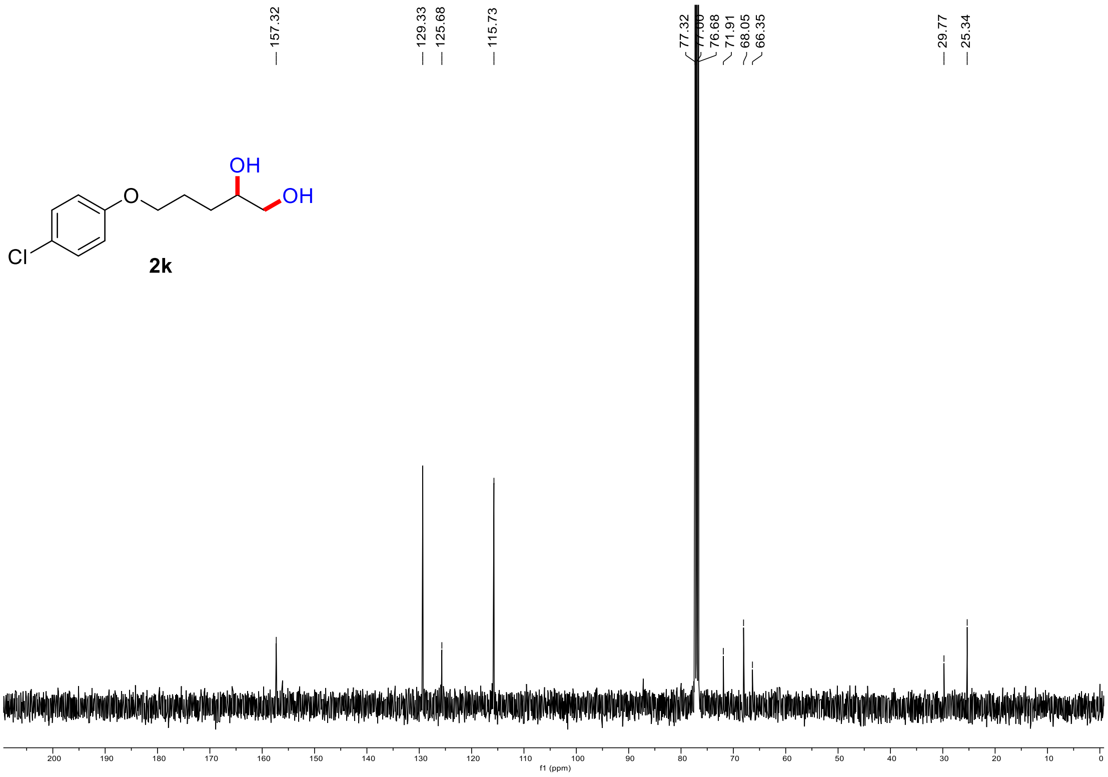


**Supplementary Figure 36**. $^{13}$C NMR of compound **2k** (100 MHz, Chloroform-*d*)

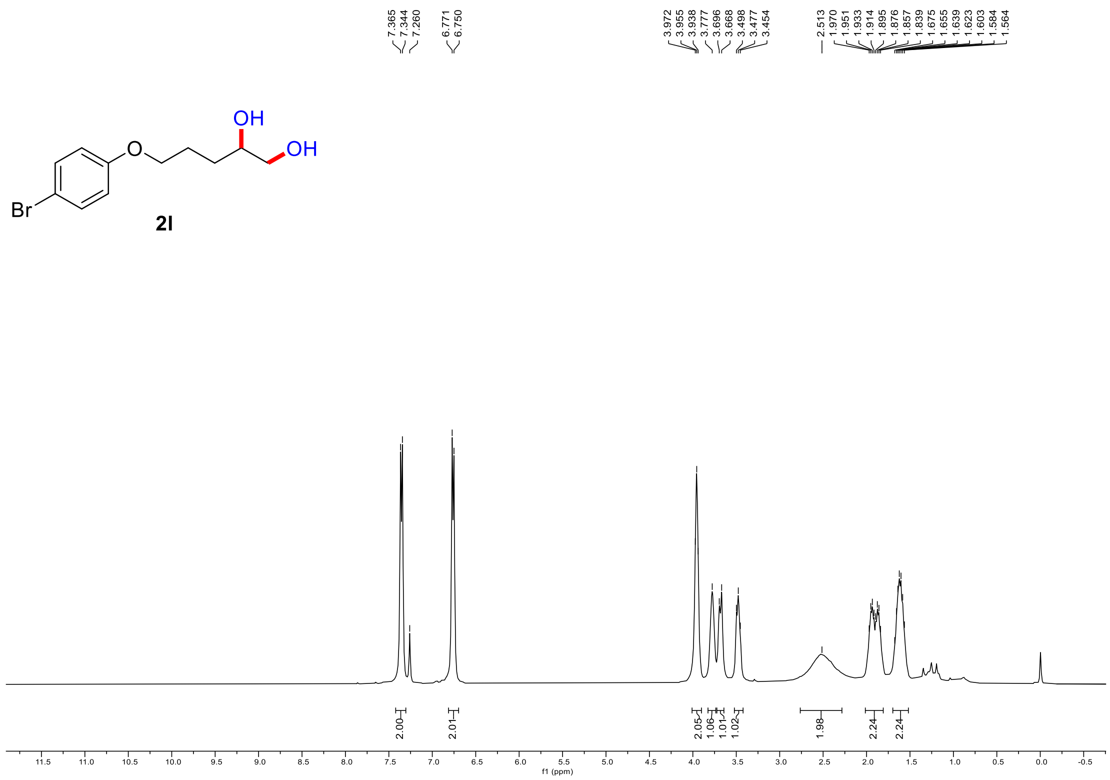

**Supplementary Figure 37**. $^{1}$H NMR of compound **2l** (400 MHz, Chloroform-*d*)

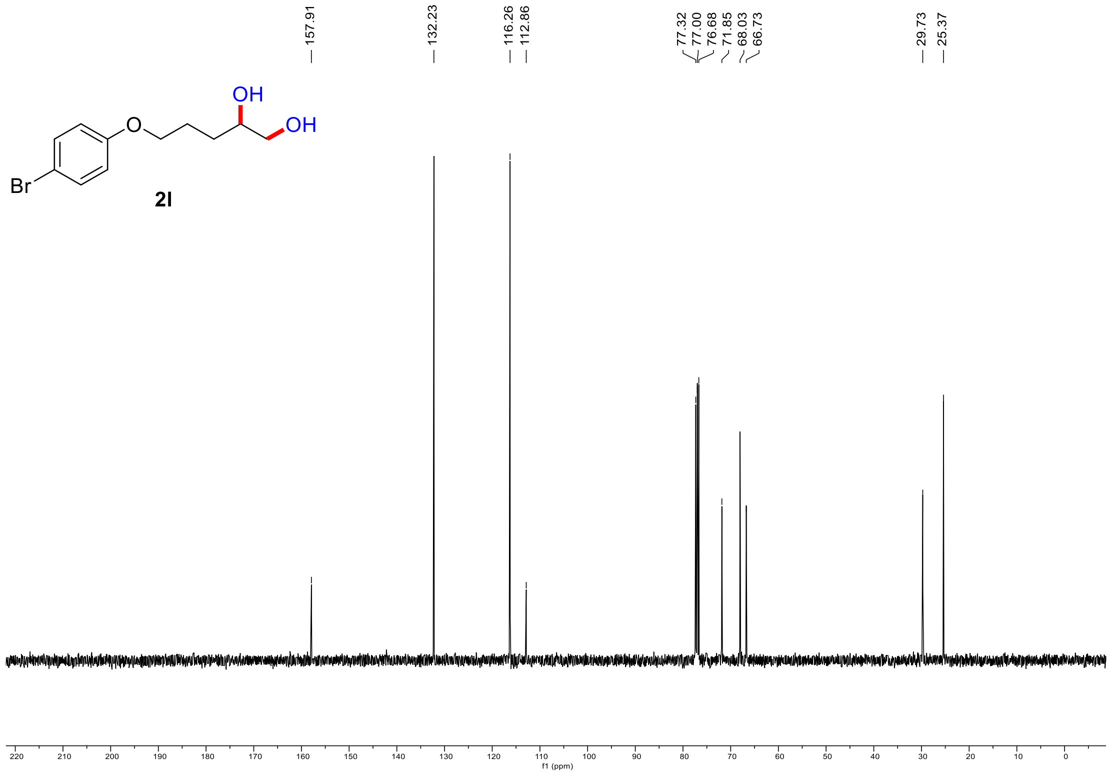

**Supplementary Figure 38**. $^{13}$C NMR of compound **2l** (100 MHz, Chloroform-*d*)

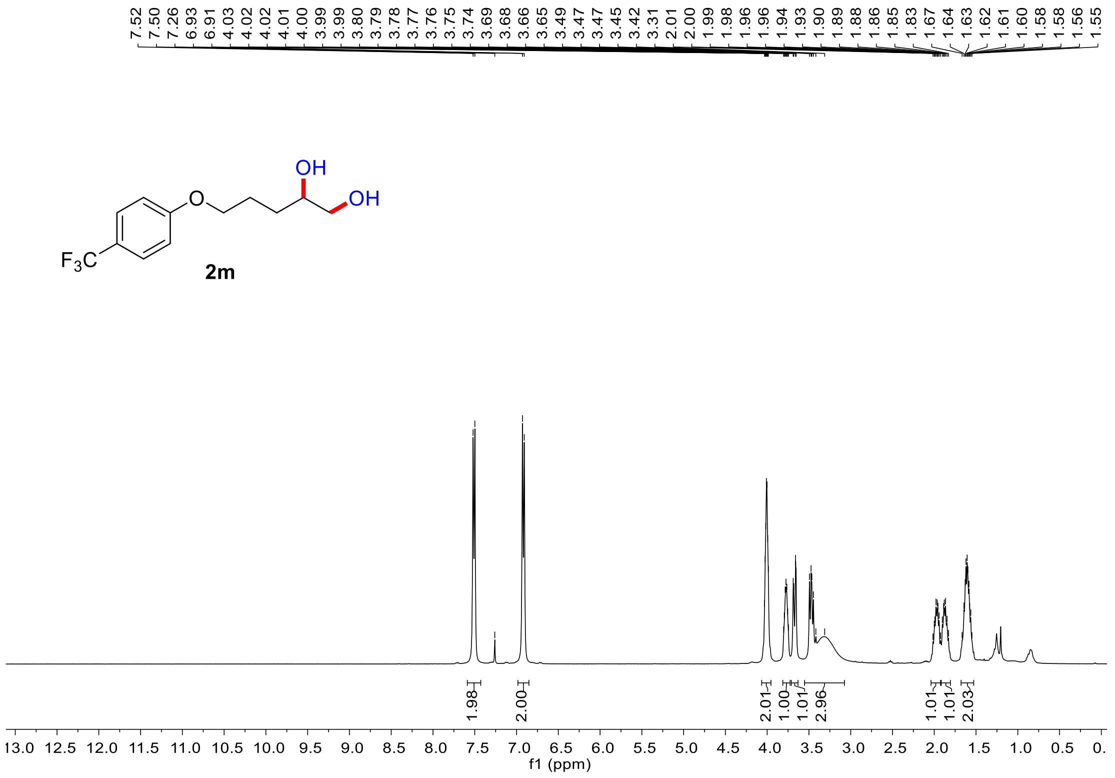

**Supplementary Figure 39**. $^{1}$H NMR of compound **2m** (400 MHz, Chloroform-*d*)

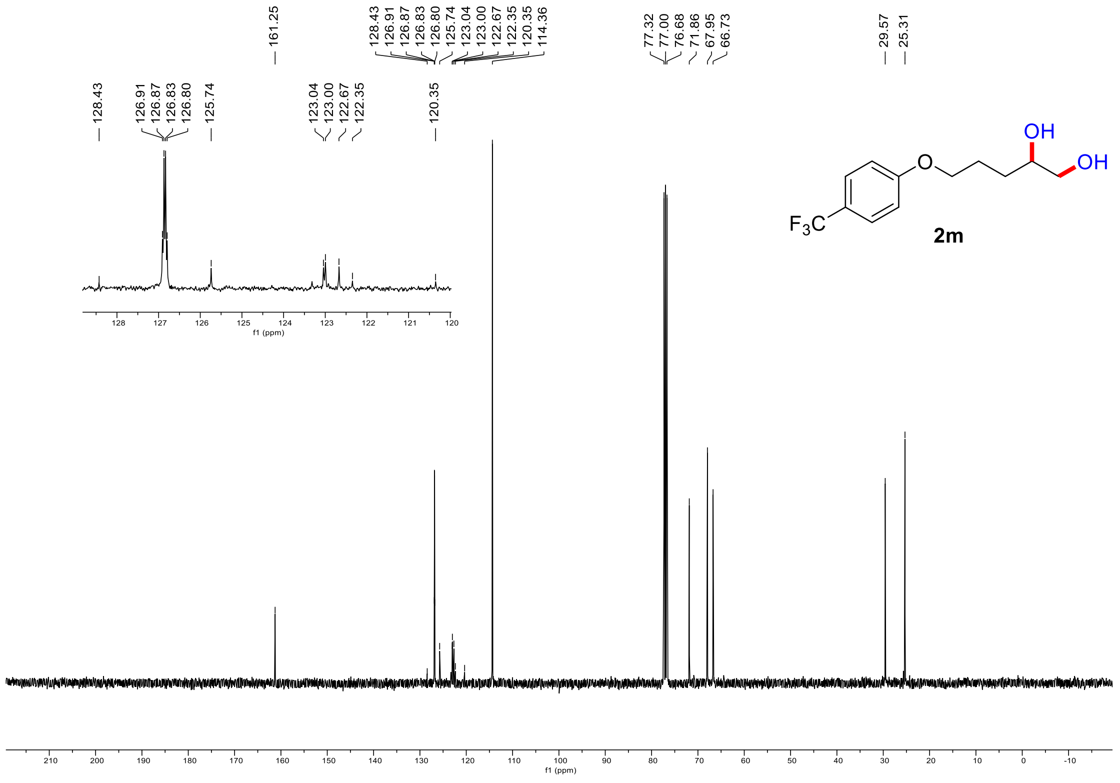

**Supplementary Figure 40**. $^{13}$C NMR of compound **2m** (100 MHz, Chloroform-*d*)

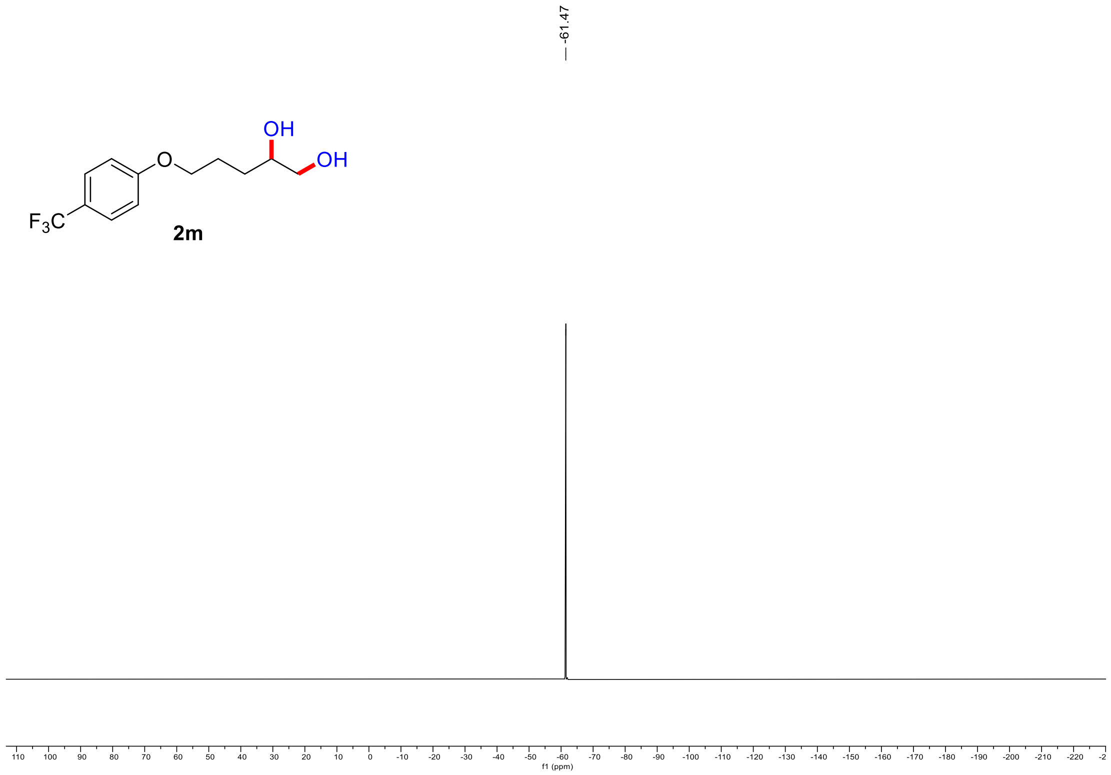


**Supplementary Figure 41**. $^{19}$F NMR of compound **2m** (375 MHz, Chloroform-*d*)

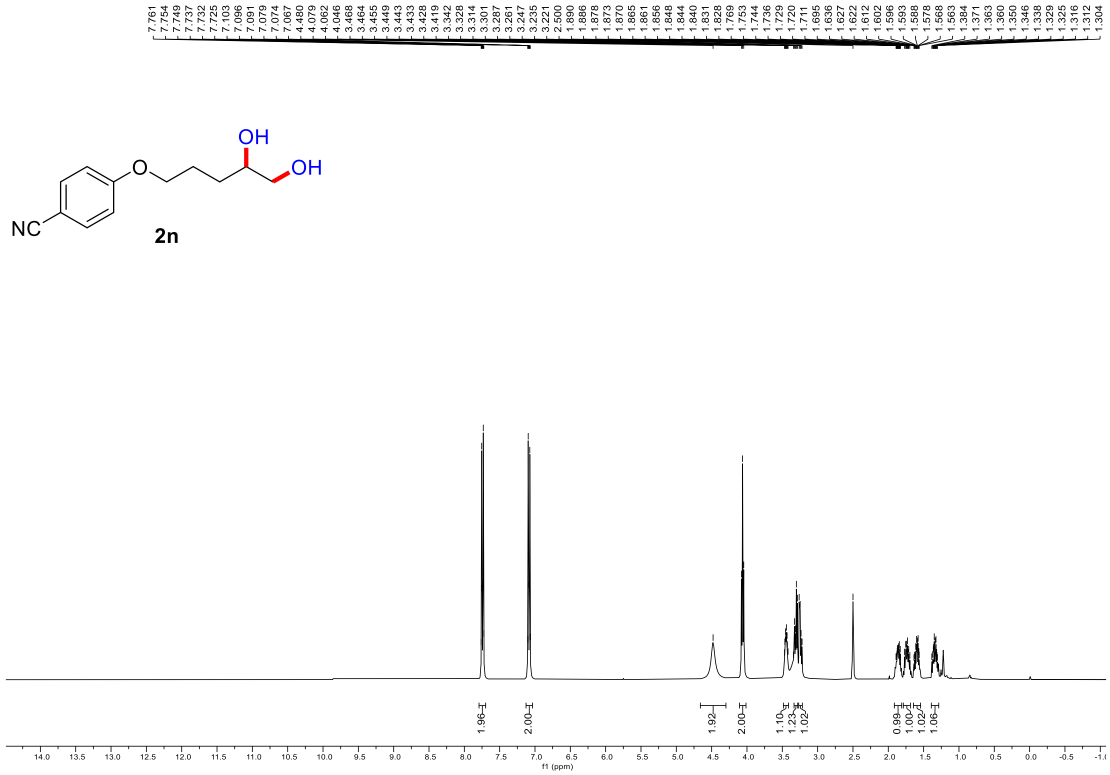


**Supplementary Figure 42**. $^{1}$H NMR of compound **2n** (400 MHz, DMSO-$d_6$)

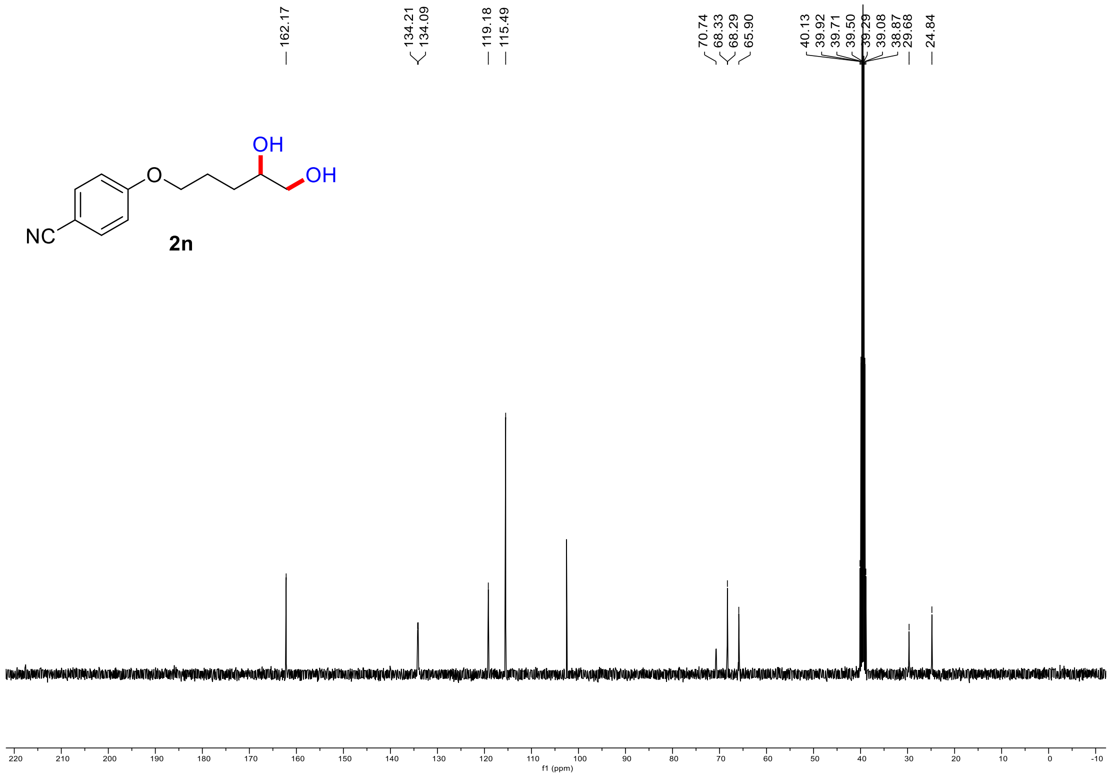


**Supplementary Figure 43**. $^{13}$C NMR of compound **2n** (100 MHz, DMSO-$d_6$)

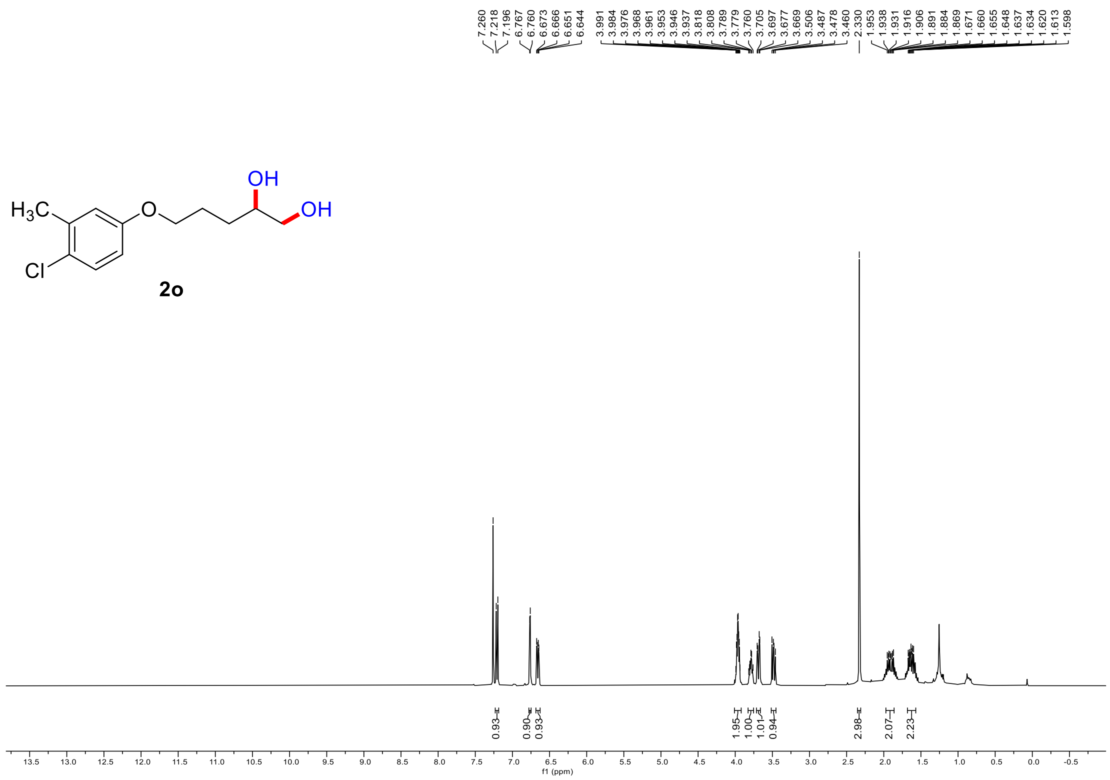

**Supplementary Figure 44**. $^{1}H$ NMR of compound **2o** (400 MHz, Chloroform-*d*)

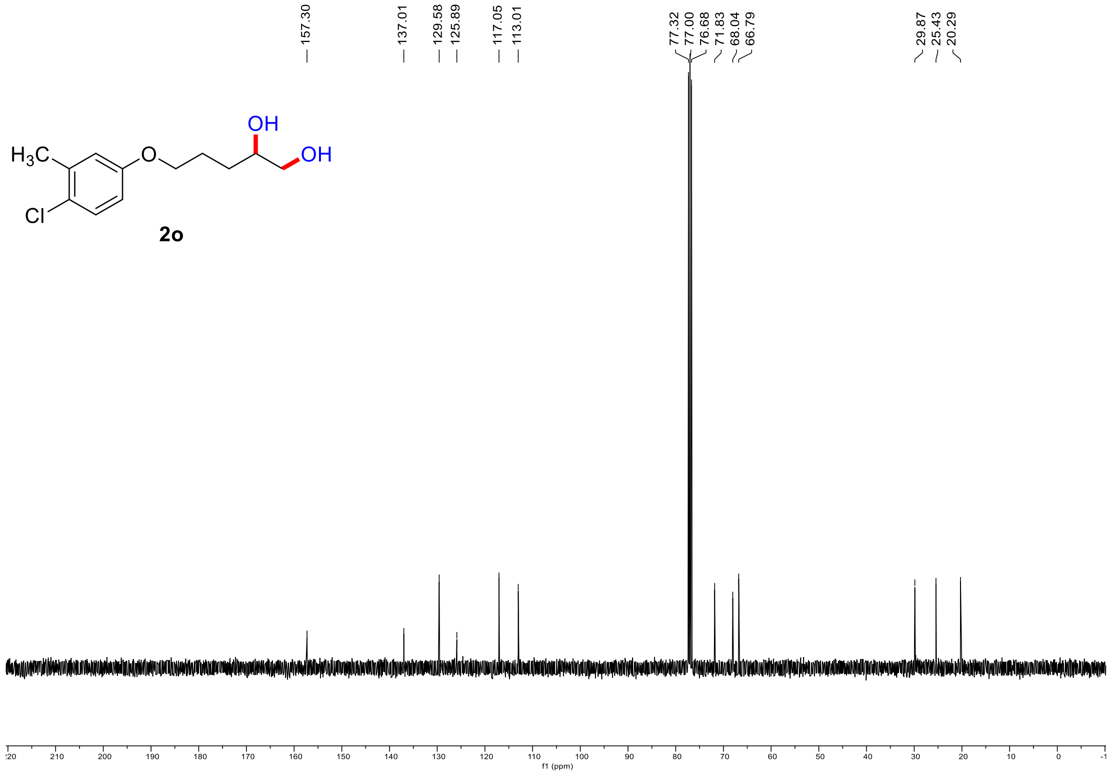

**Supplementary Figure 45**. $^{13}C$ NMR of compound **2o** (100 MHz, Chloroform-*d*)

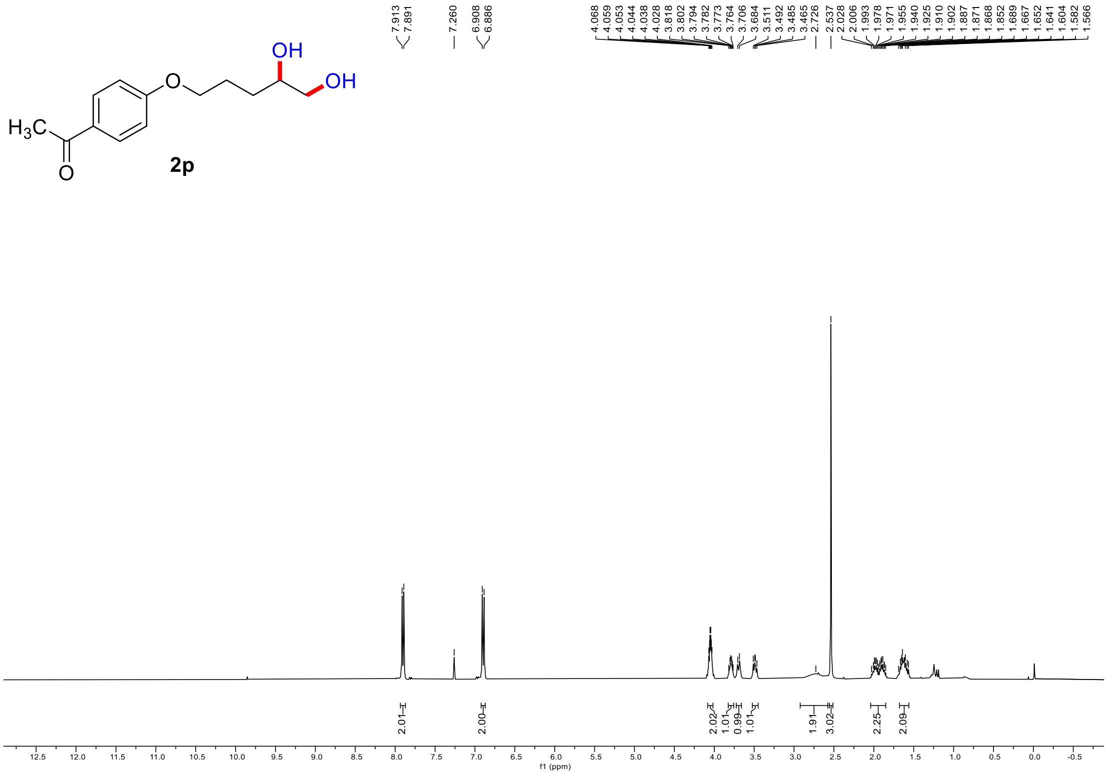


**Supplementary Figure 46**. $^{1}H$ NMR of compound **2p** (400 MHz, Chloroform-*d*)

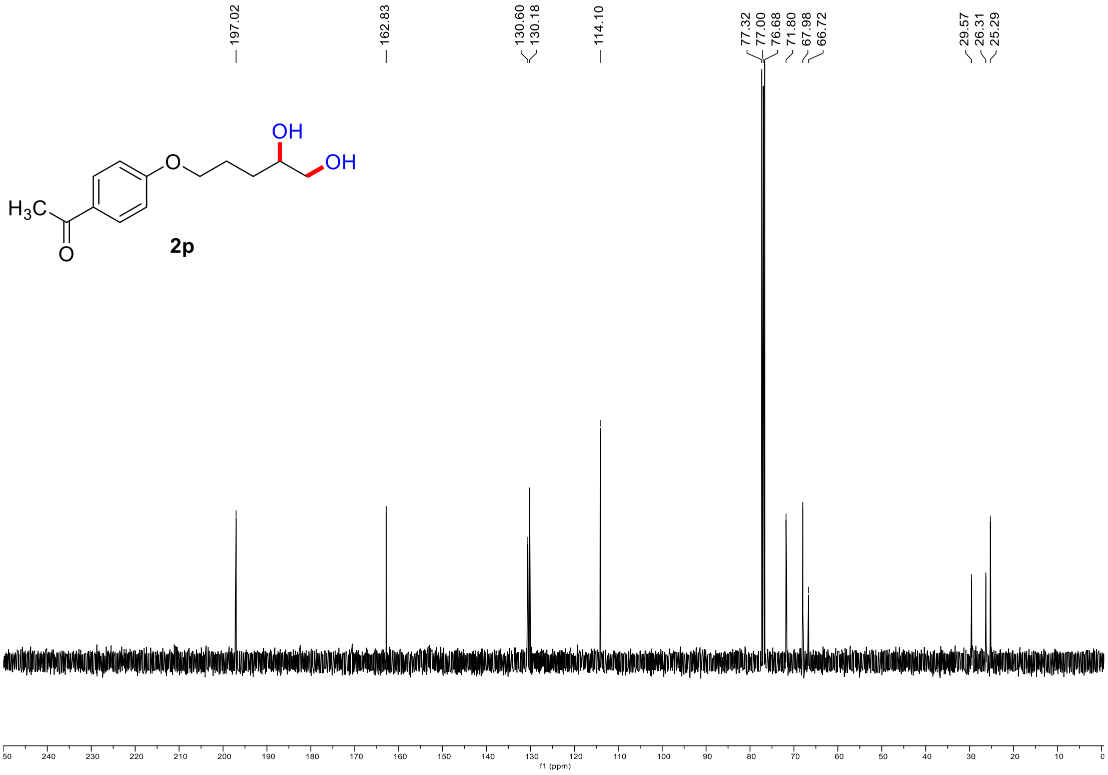


**Supplementary Figure 47**. $^{13}C$ NMR of compound **2p** (100 MHz, Chloroform-*d*)

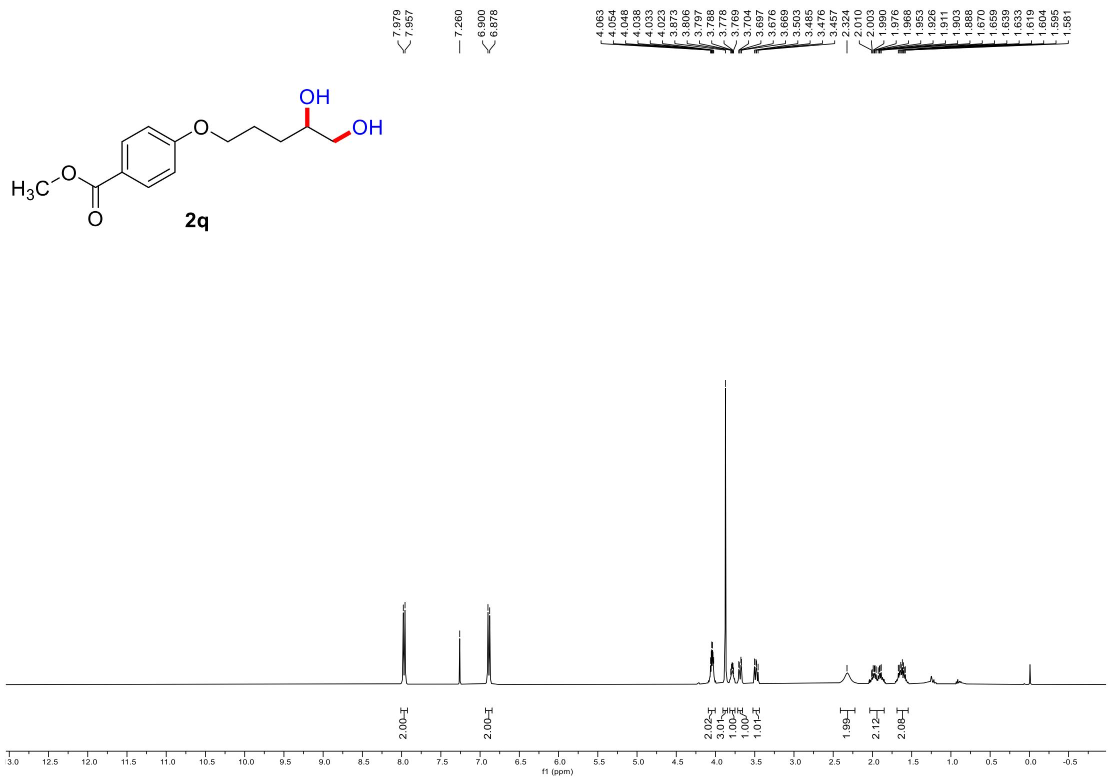

**Supplementary Figure 48**. $^{1}H$ NMR of compound **2q** (400 MHz, Chloroform-*d*)

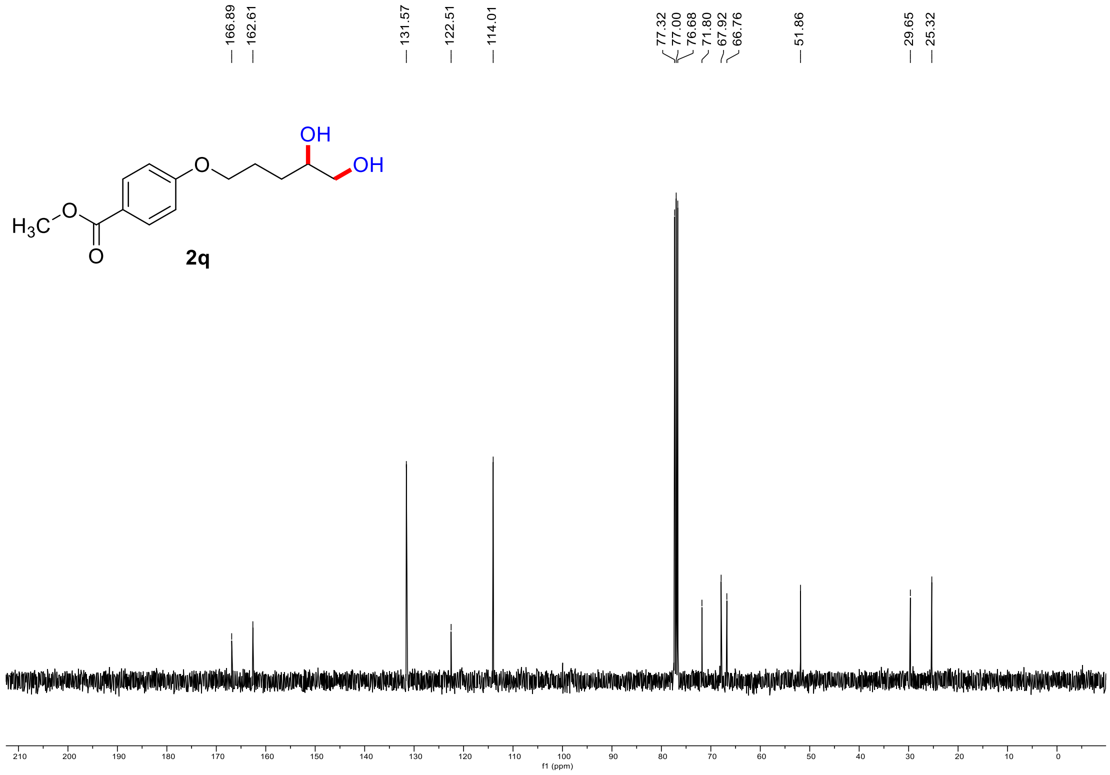

**Supplementary Figure 49**. $^{13}C$ NMR of compound **2q** (100 MHz, Chloroform-*d*)

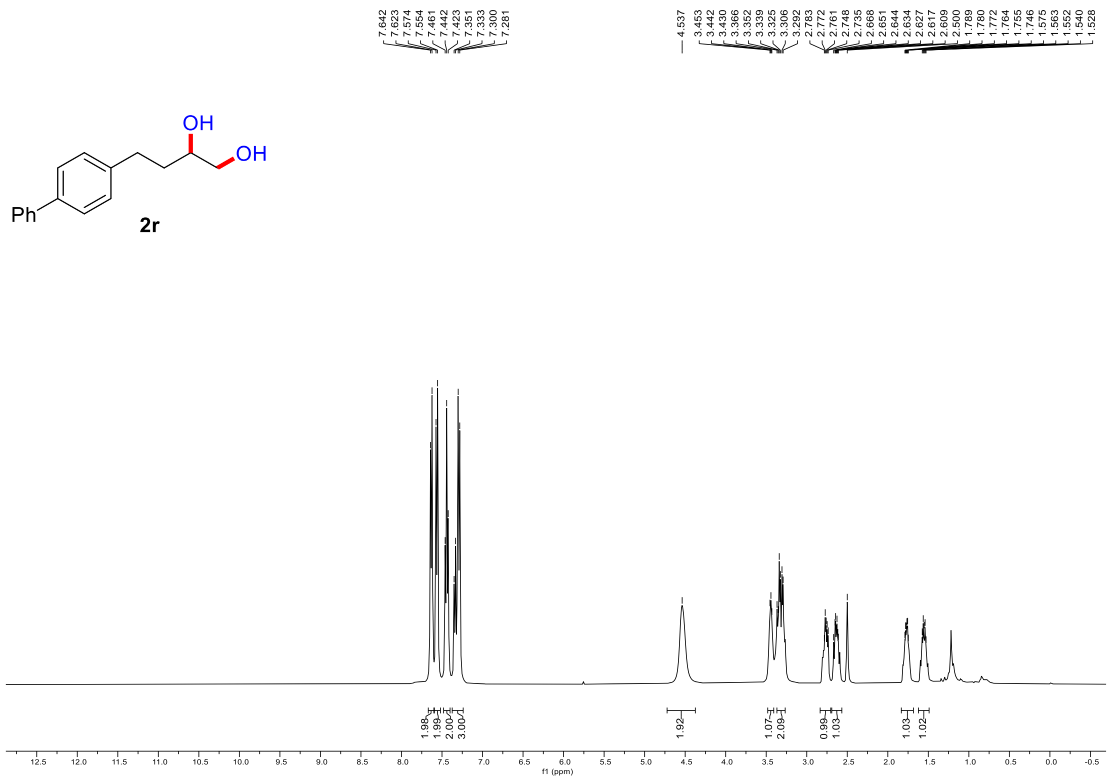

**Supplementary Figure 50**. $^{1}$H NMR of compound **2r** (400 MHz, DMSO-$d_6$)

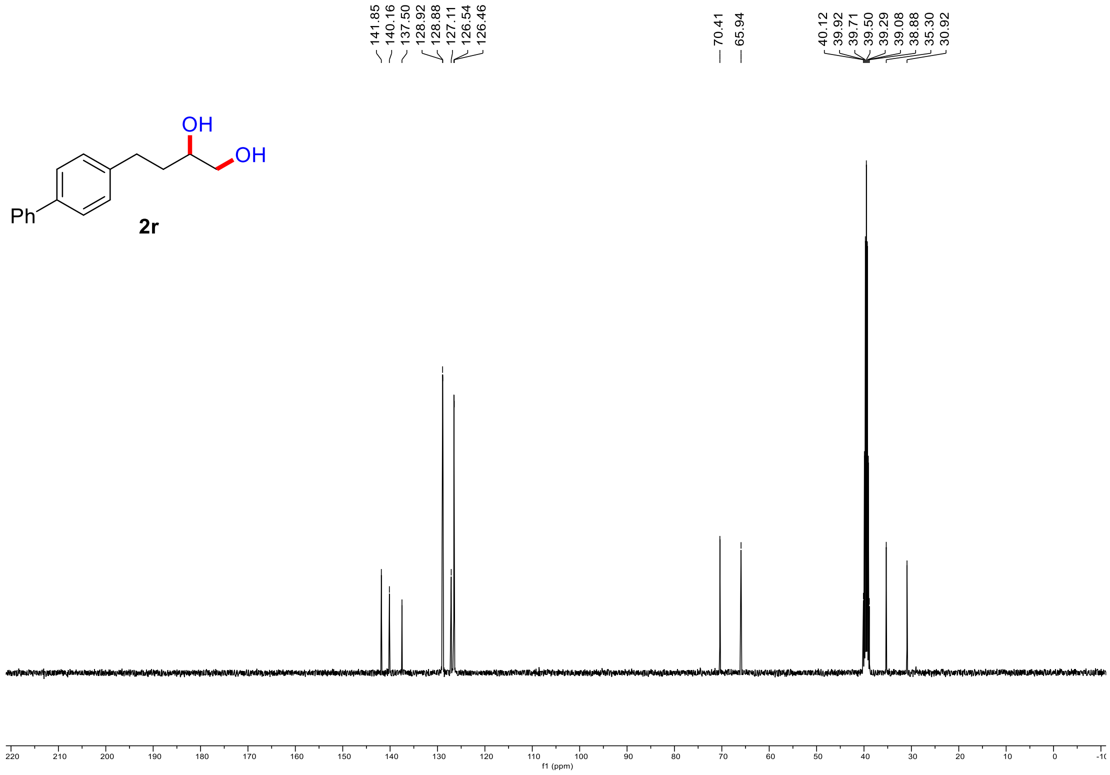

**Supplementary Figure 51**. $^{13}$C NMR of compound **2r** (100 MHz, DMSO-$d_6$)

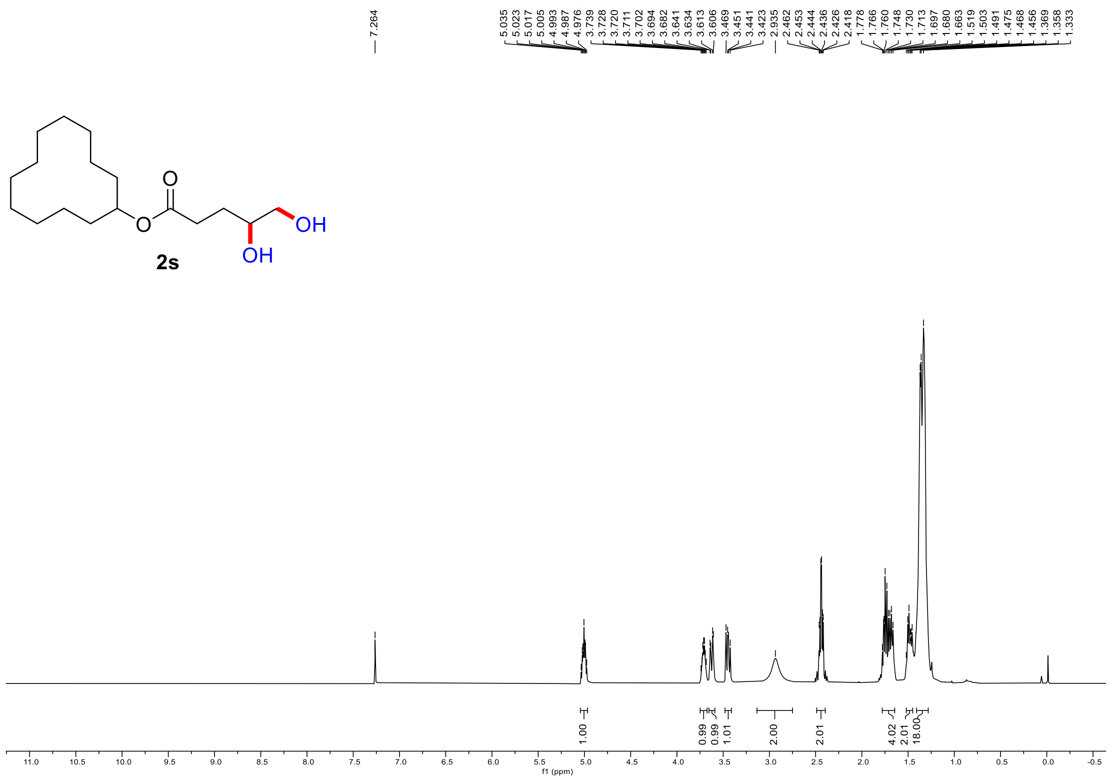


**Supplementary Figure 52**. $^{1}$H NMR of compound **2s** (400 MHz, Chloroform-*d*)

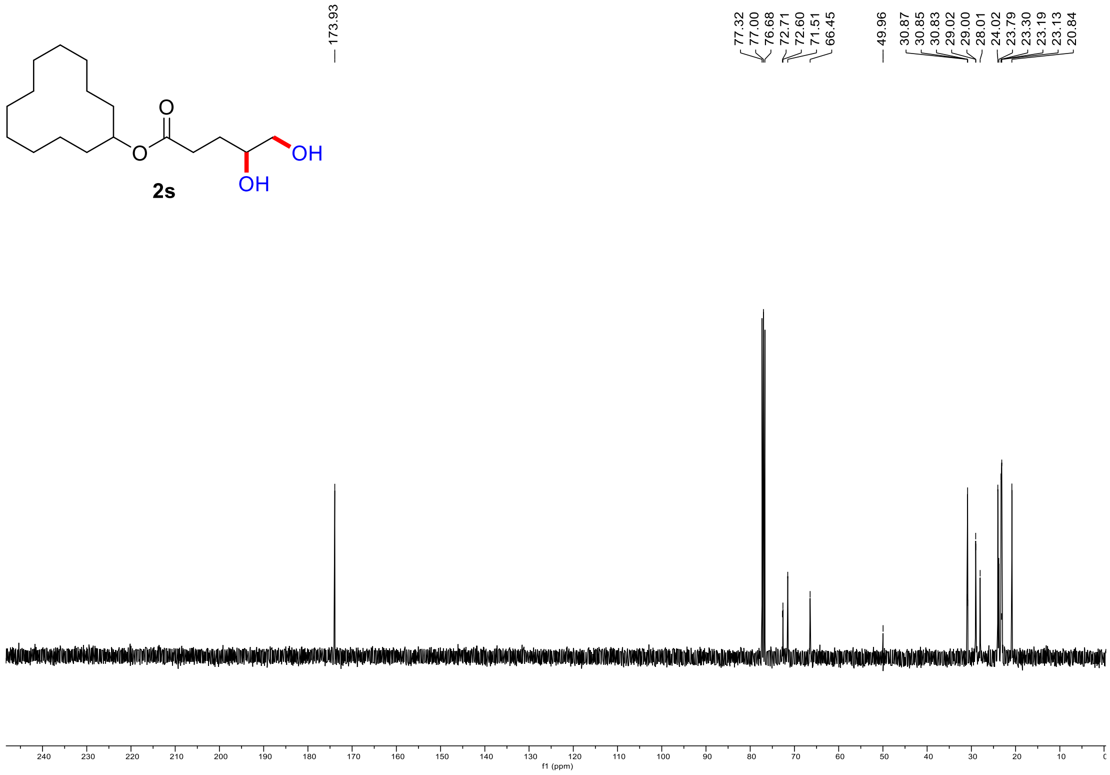


**Supplementary Figure 53**. $^{13}$C NMR of compound **2s** (100 MHz, Chloroform-*d*)

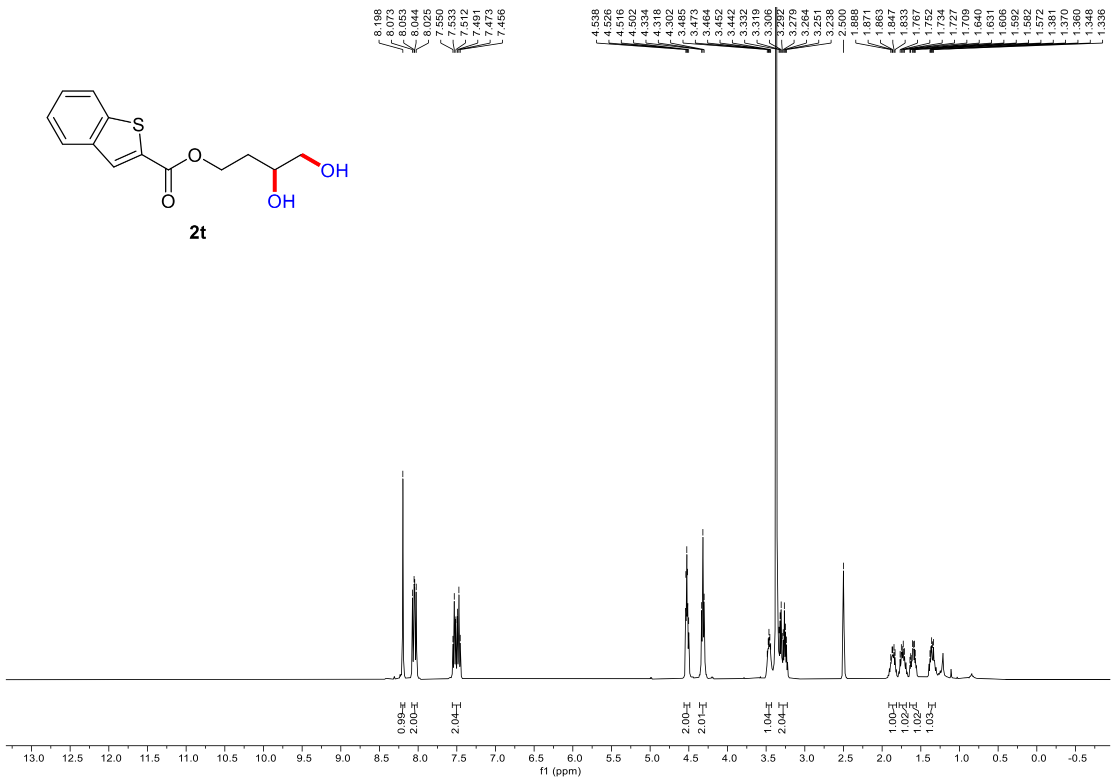

**Supplementary Figure 54**. $^{1}$H NMR of compound **2t** (400 MHz, DMSO-$d_6$)

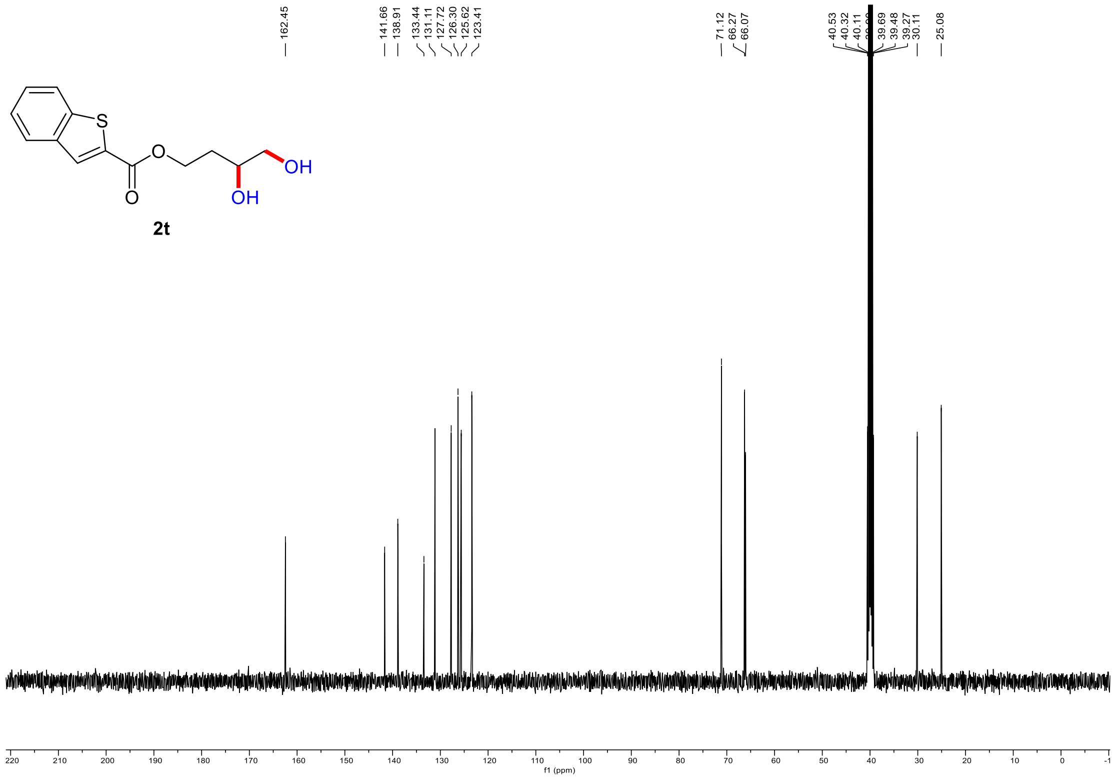

**Supplementary Figure 55**. $^{13}$C NMR of compound **2t** (100 MHz, DMSO-$d_6$)

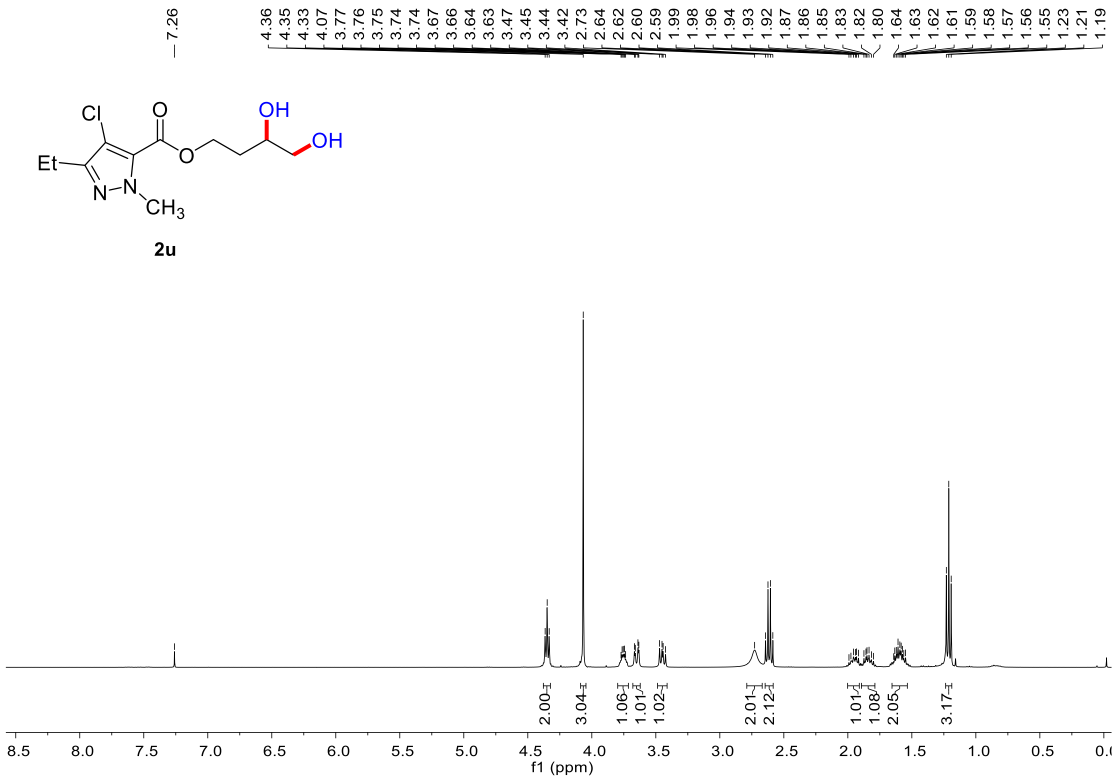


**Supplementary Figure 56**. $^{1}$H NMR of compound **2u** (400 MHz, Chloroform-*d*)

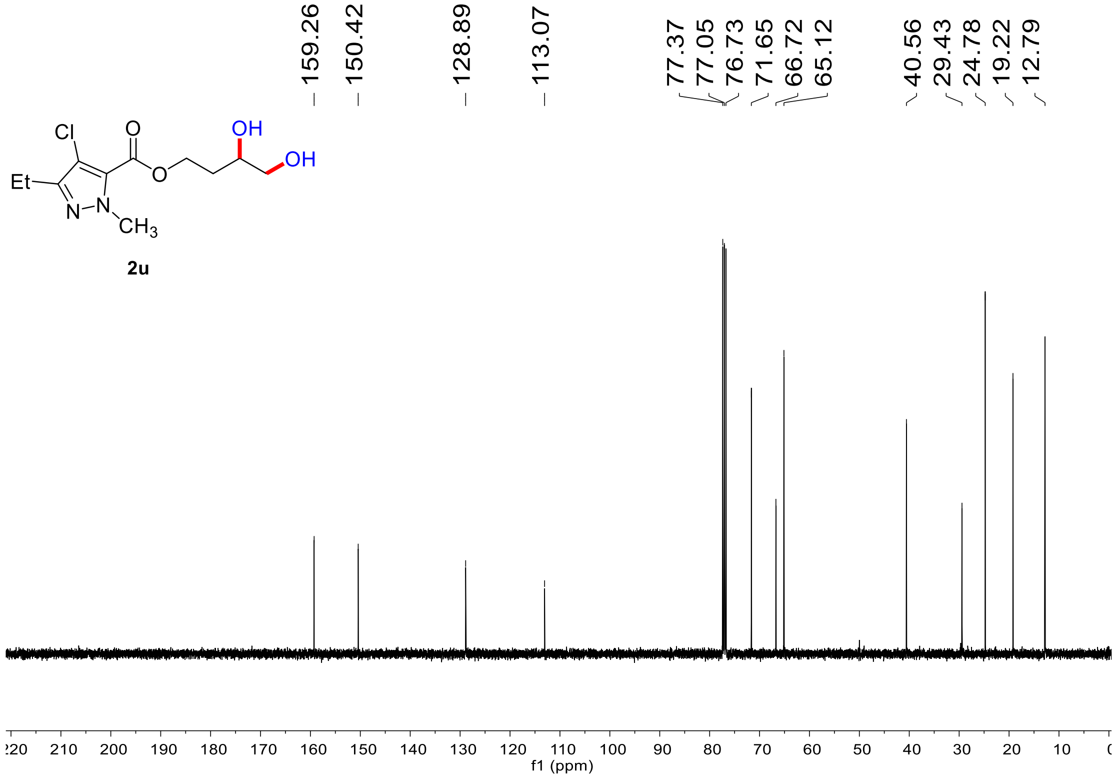


**Supplementary Figure 57**. $^{13}$C NMR of compound **2u** (100 MHz, Chloroform-*d*)

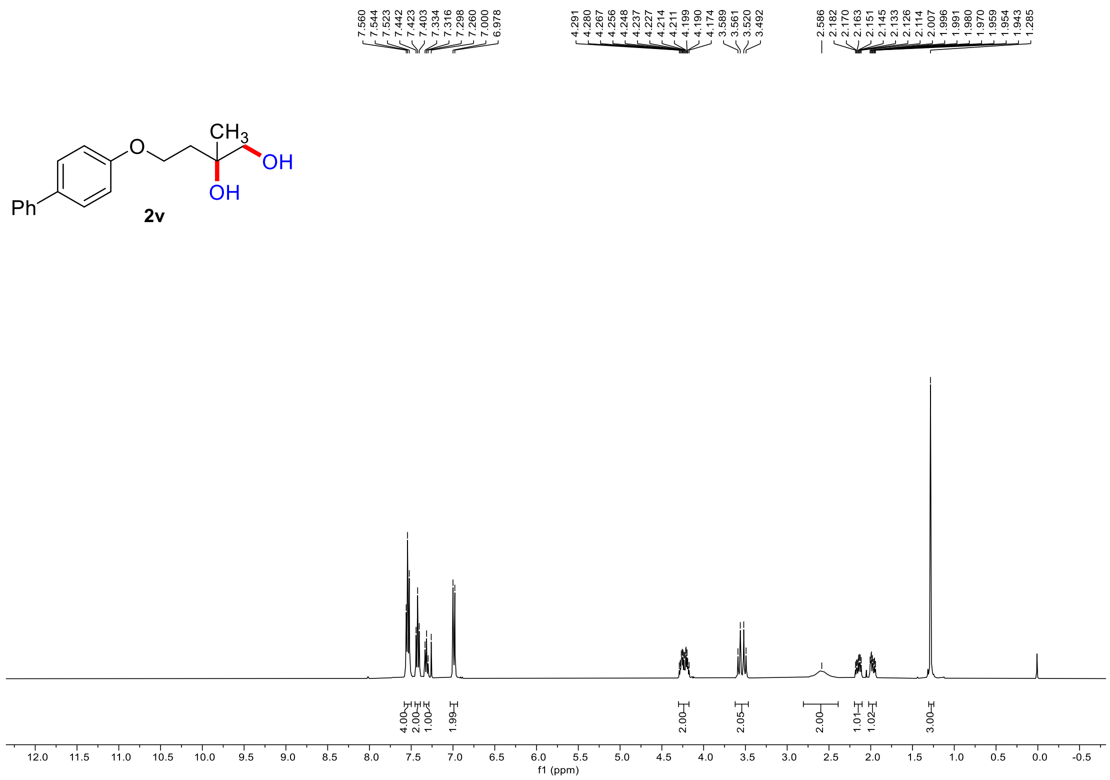

**Supplementary Figure 58**. $^{1}$H NMR of compound **2v** (400 MHz, Chloroform-*d*)

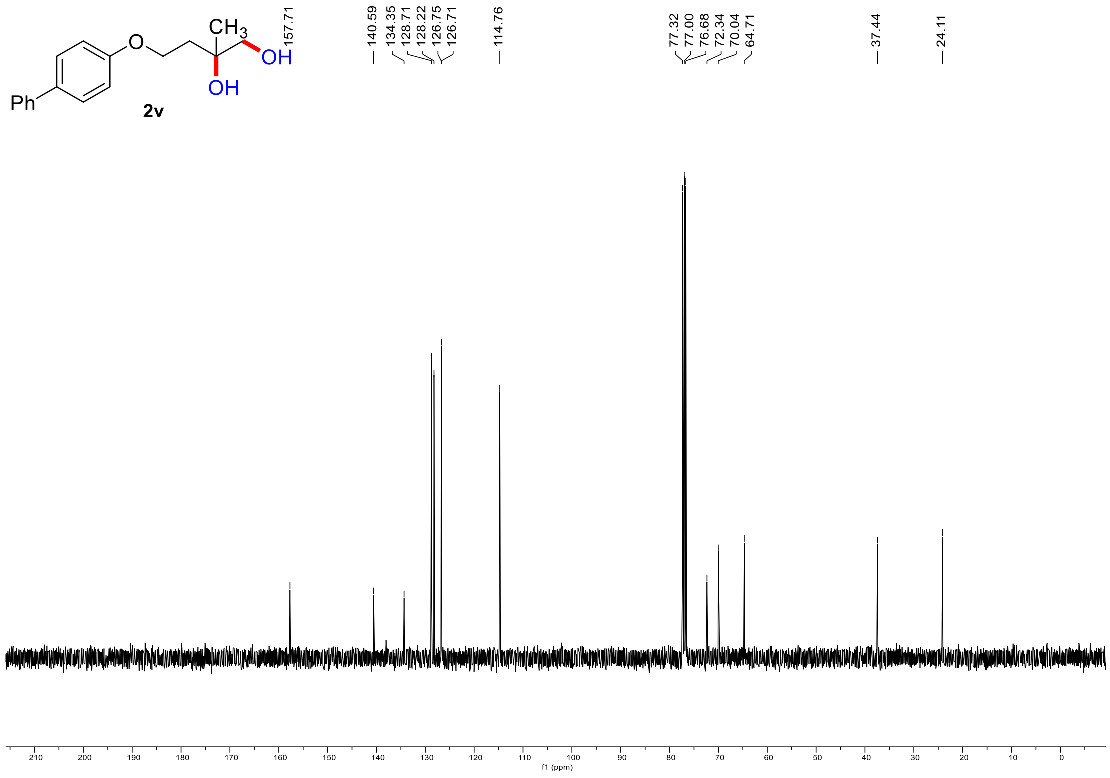

**Supplementary Figure 59**. $^{13}$C NMR of compound **2v** (100 MHz, Chloroform-*d*)

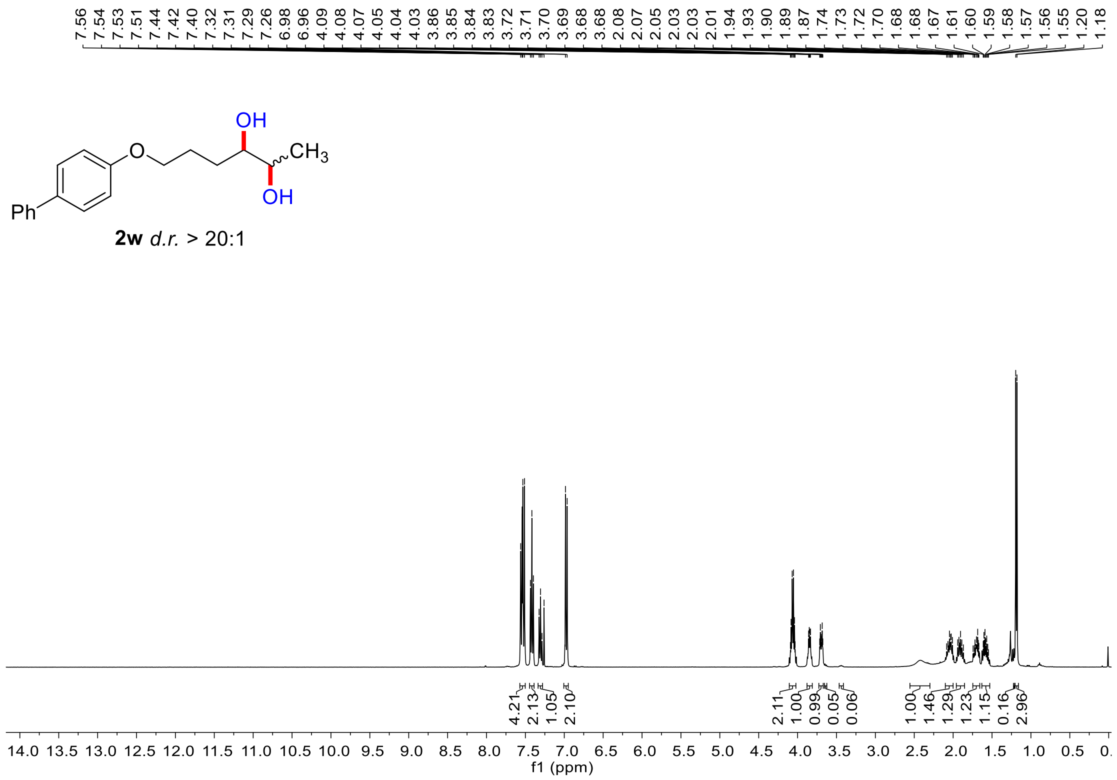


**Supplementary Figure 60**. $^{1}$H NMR of compound **2w** (400 MHz, Chloroform-*d*)

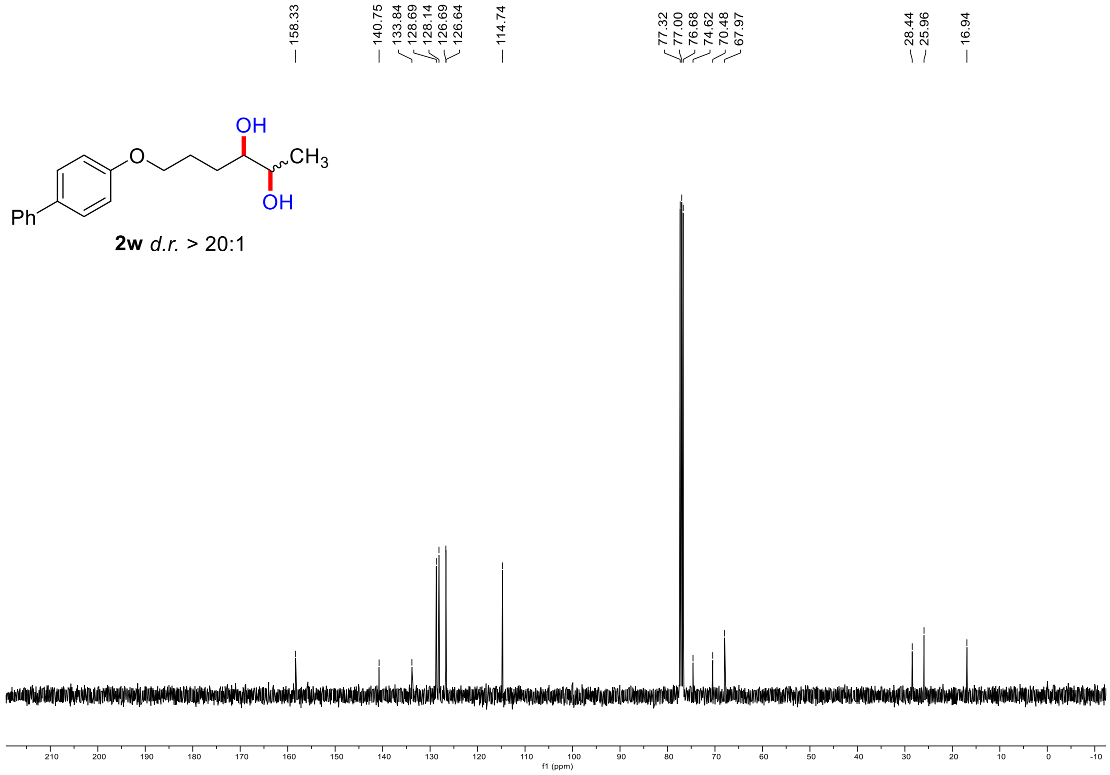


**Supplementary Figure 61**. $^{13}$C NMR of compound **2w** (100 MHz, Chloroform-*d*)

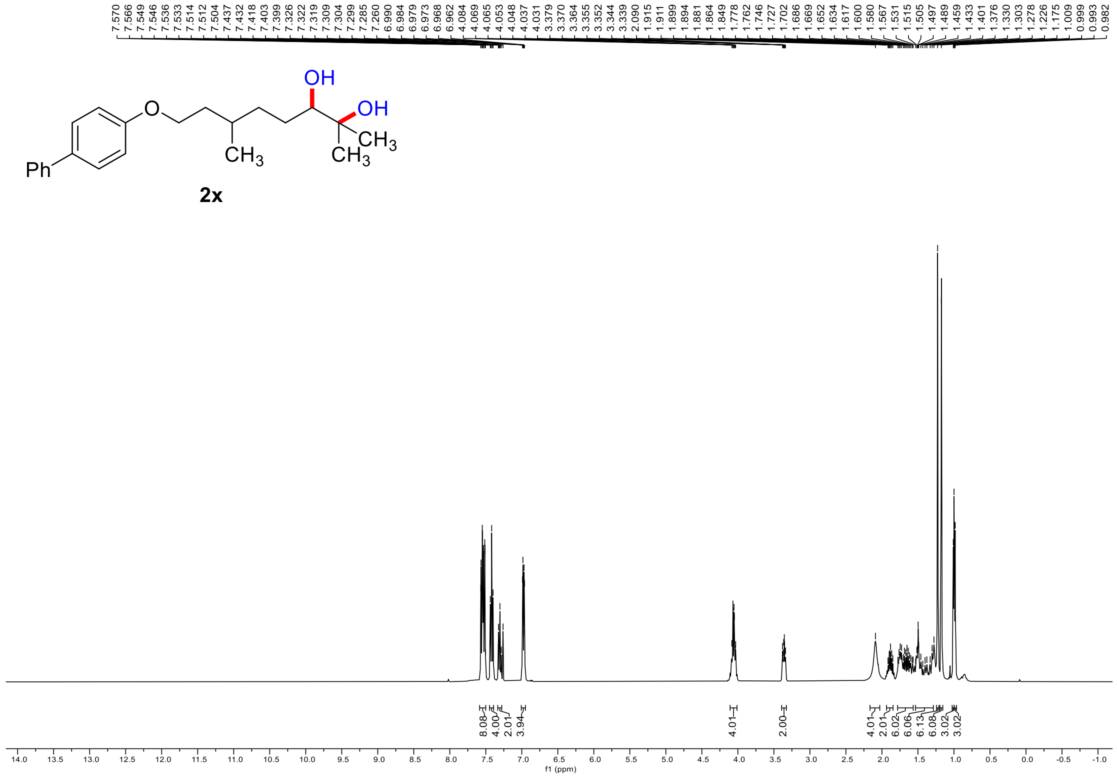


**Supplementary Figure 62**. $^{1}$H NMR of compound **2x** (400 MHz, Chloroform-*d*)

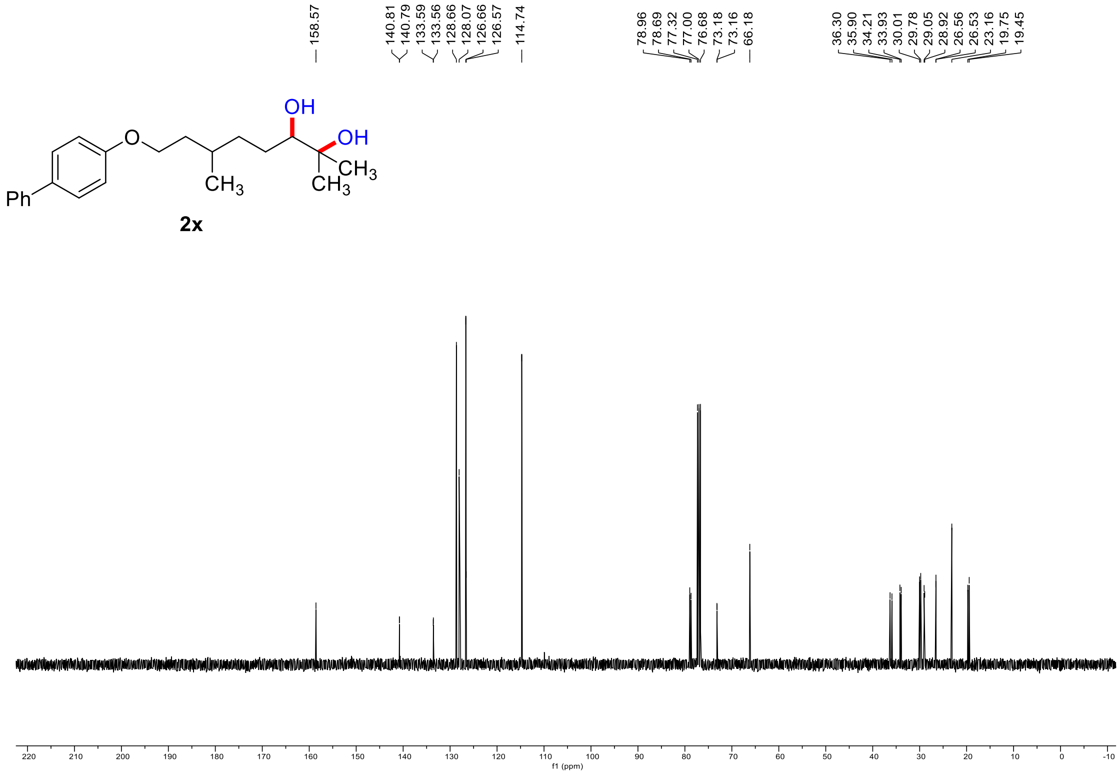


**Supplementary Figure 63**. $^{13}$C NMR of compound **2x** (100 MHz, Chloroform-*d*)

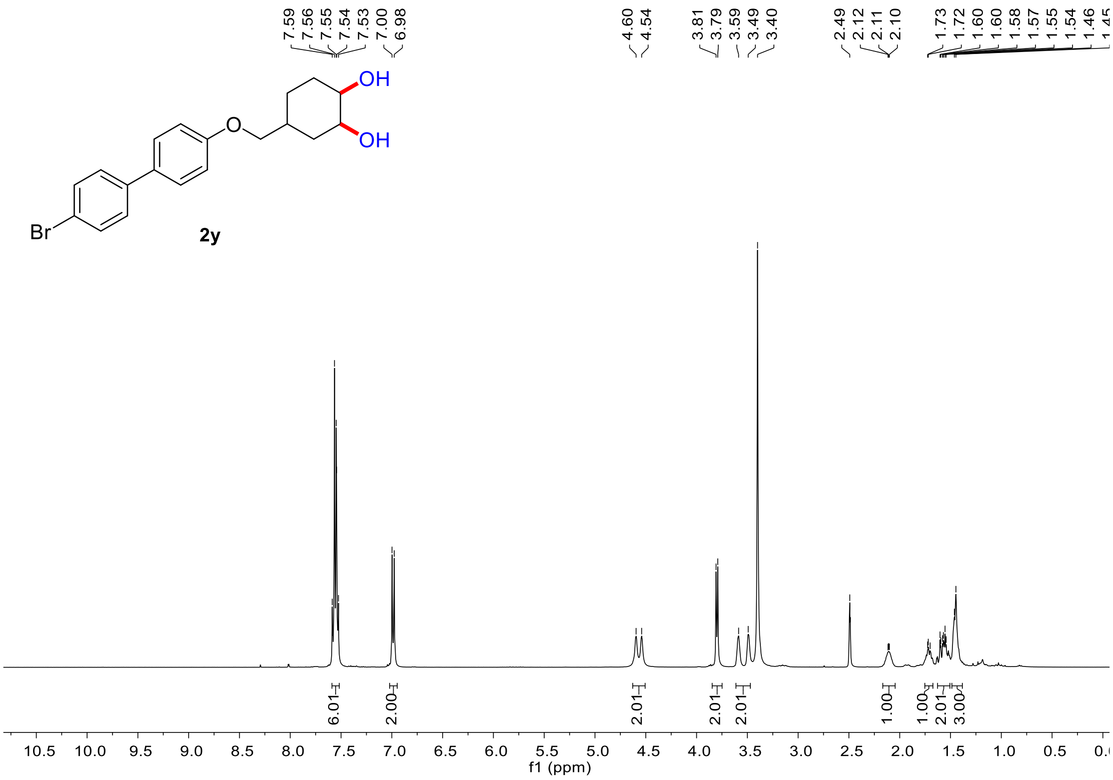

**Supplementary Figure 64**. $^{1}H$ NMR of compound **2y** (400 MHz, DMSO-$d_6$)

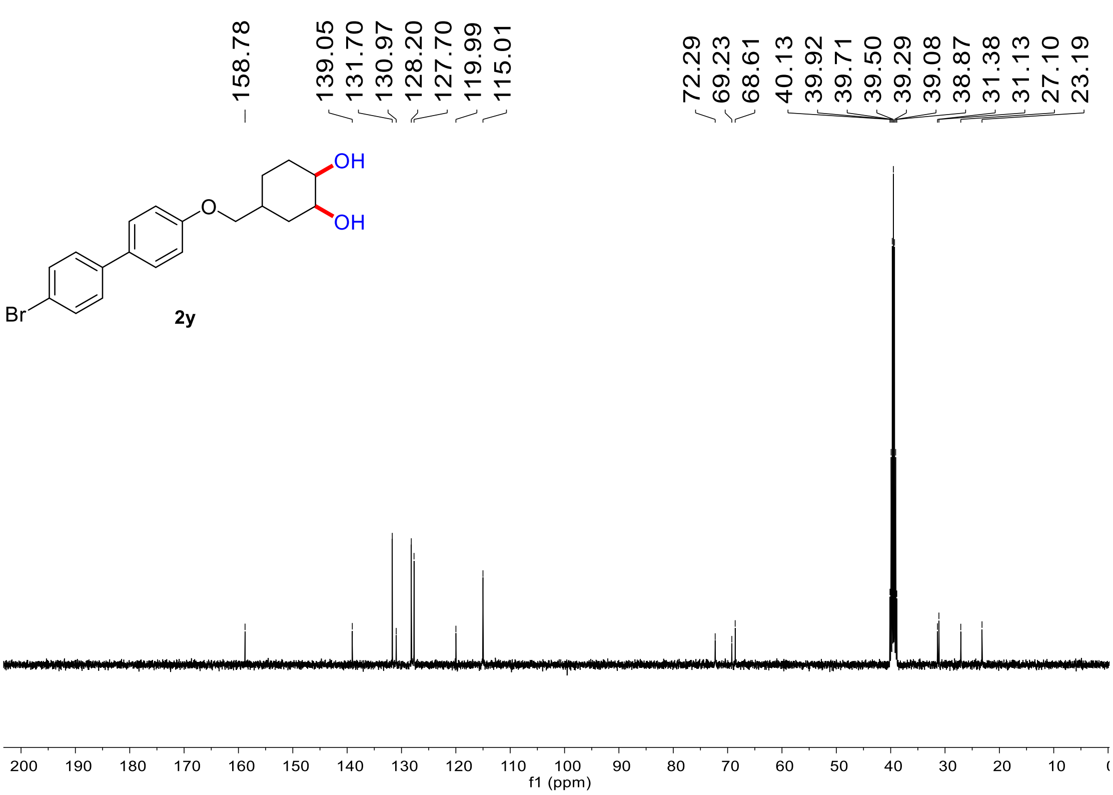

**Supplementary Figure 65**. $^{13}C$ NMR of compound **2y** (400 MHz, DMSO-$d_6$)

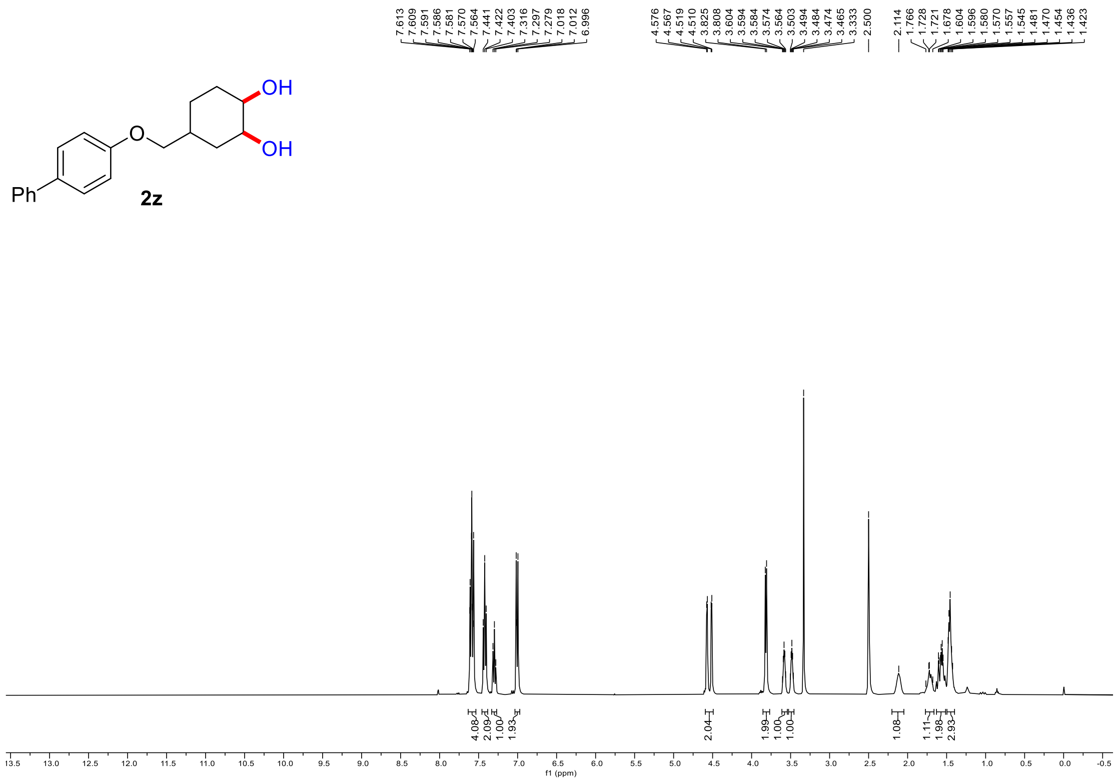


**Supplementary Figure 66**. $^{1}$H NMR of compound **2z** (400 MHz, DMSO-$d_6$)

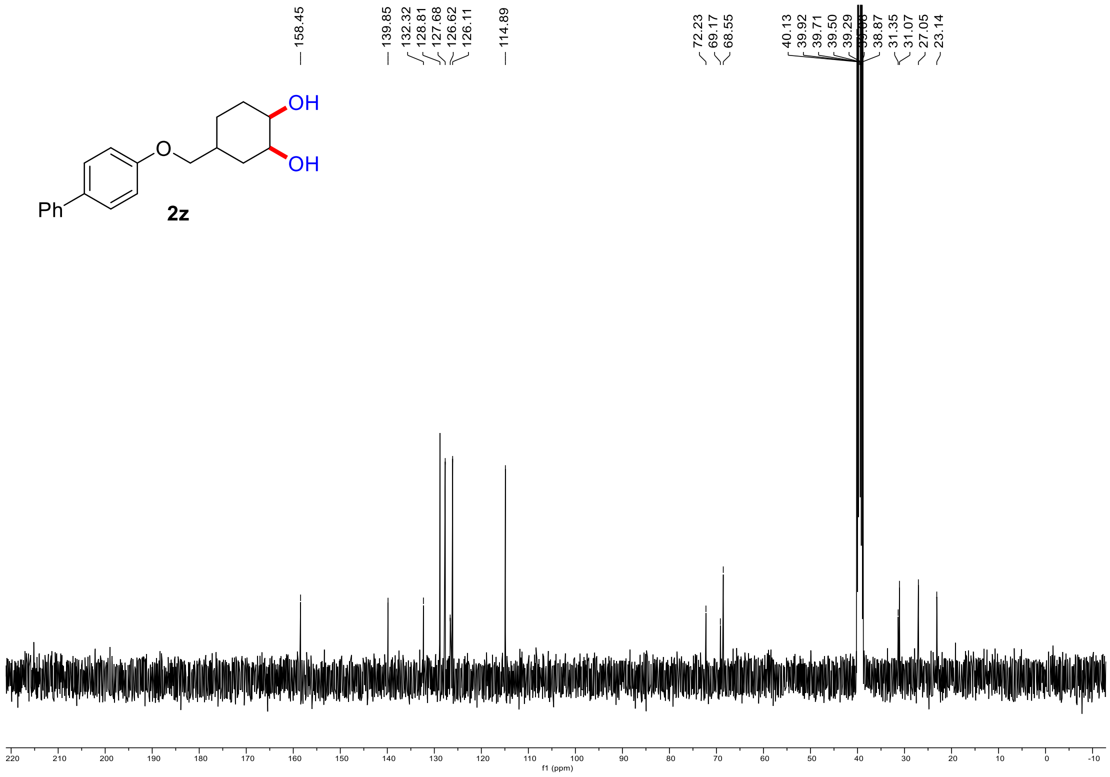


**Supplementary Figure 67**. $^{13}$C NMR of compound **2z** (100 MHz, DMSO-$d_6$)

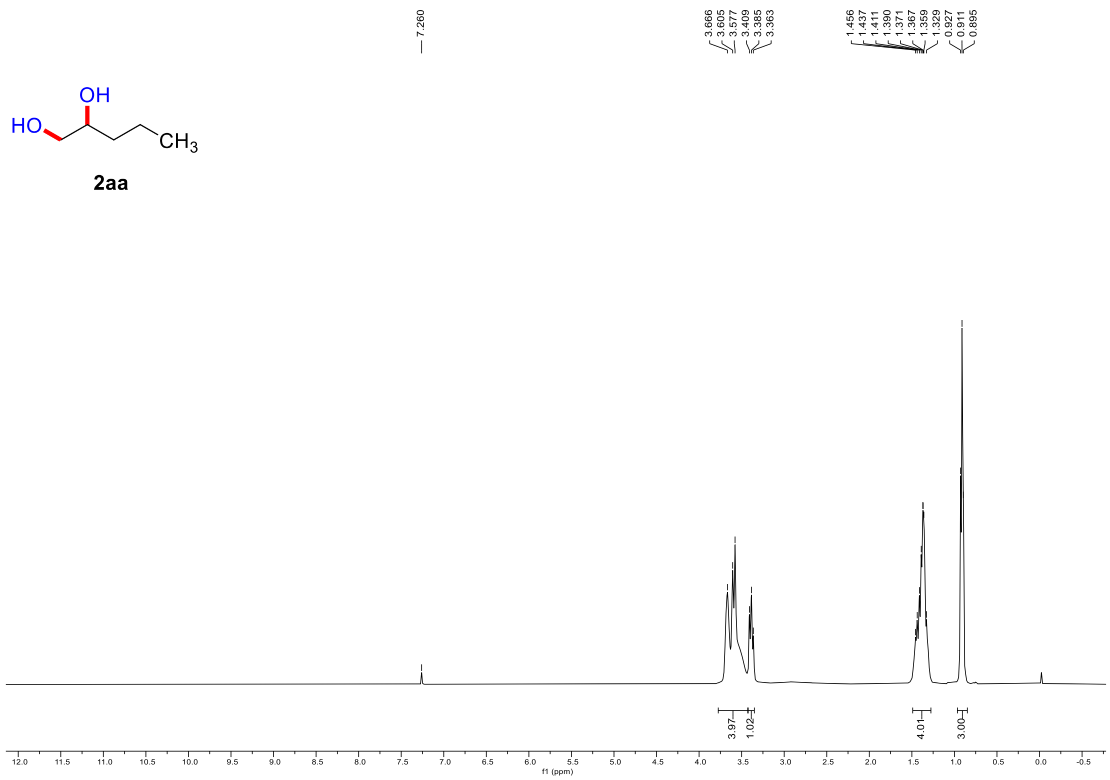


**Supplementary Figure 68**. $^{1}$H NMR of compound **2aa** (400 MHz, Chloroform-*d*)

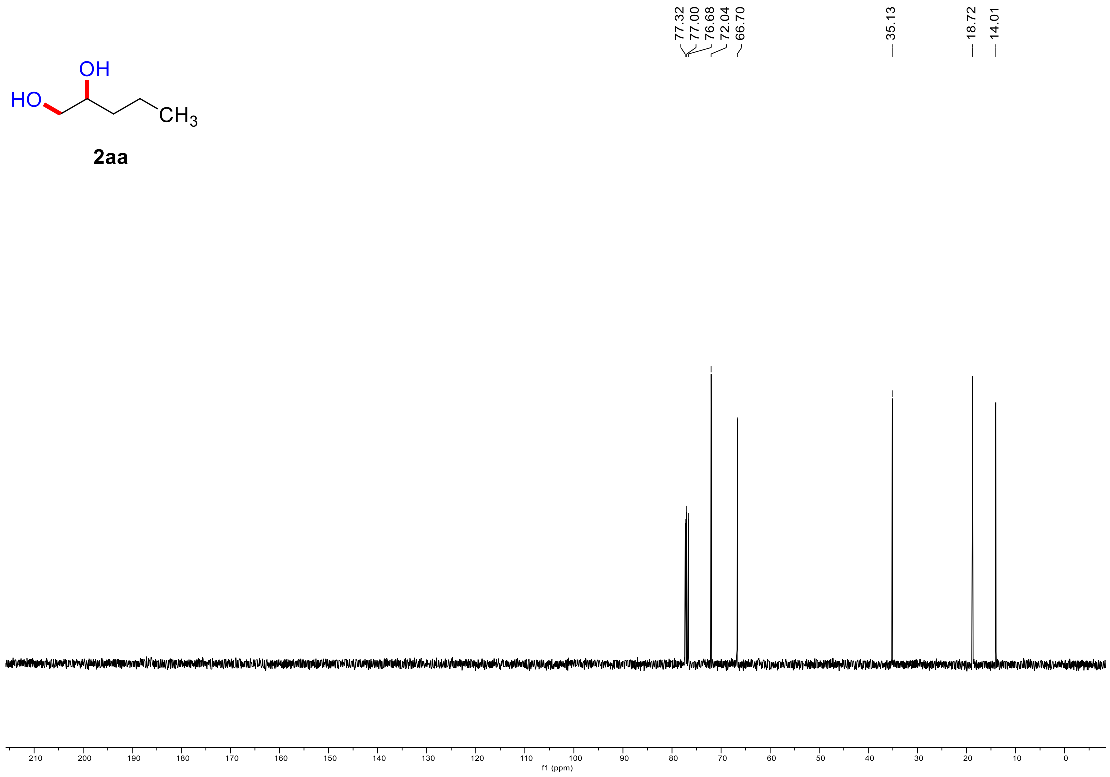


**Supplementary Figure 69**. $^{13}$C NMR of compound **2aa** (100 MHz, Chloroform-*d*)

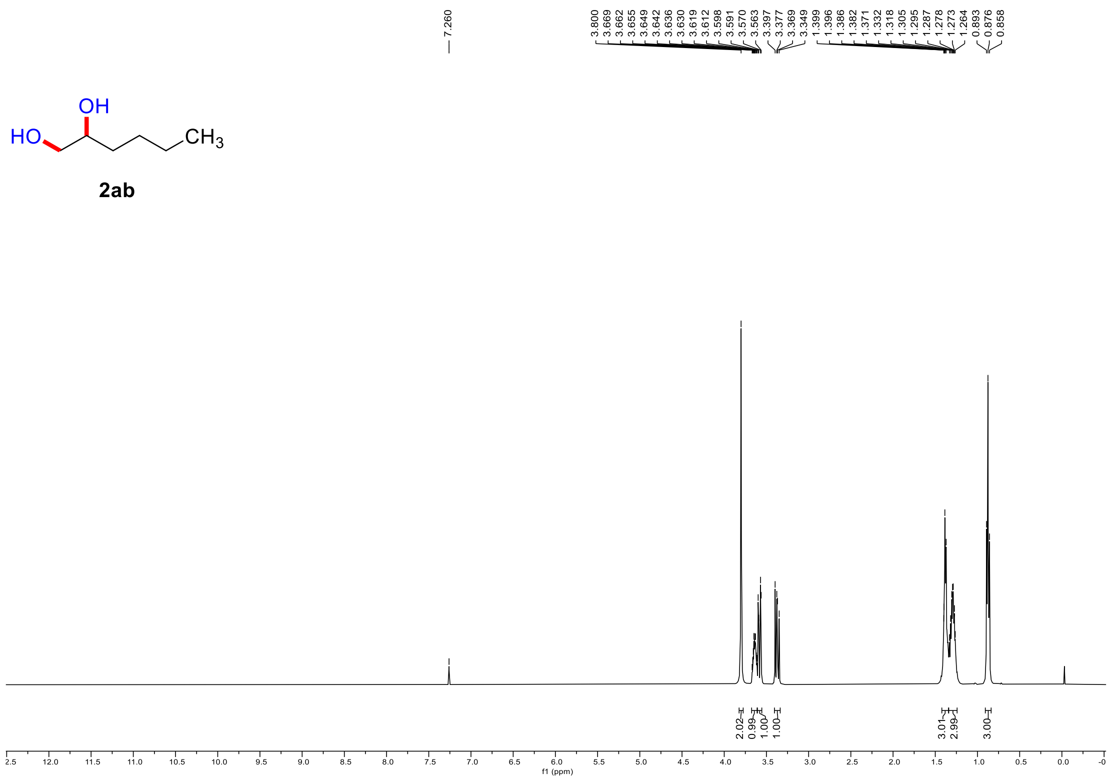

**Supplementary Figure 70**. $^{1}H$ NMR of compound **2ab** (400 MHz, Chloroform-*d*)

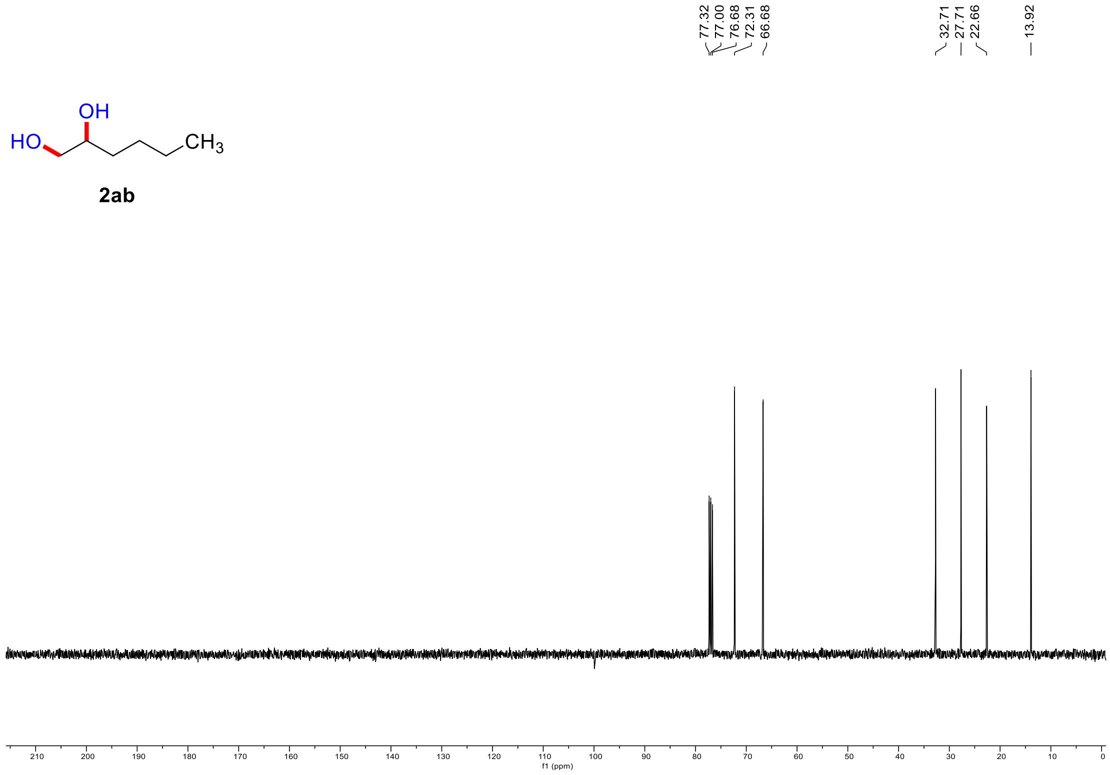

**Supplementary Figure 71**. $^{13}C$ NMR of compound **2ab** (100 MHz, Chloroform-*d*)

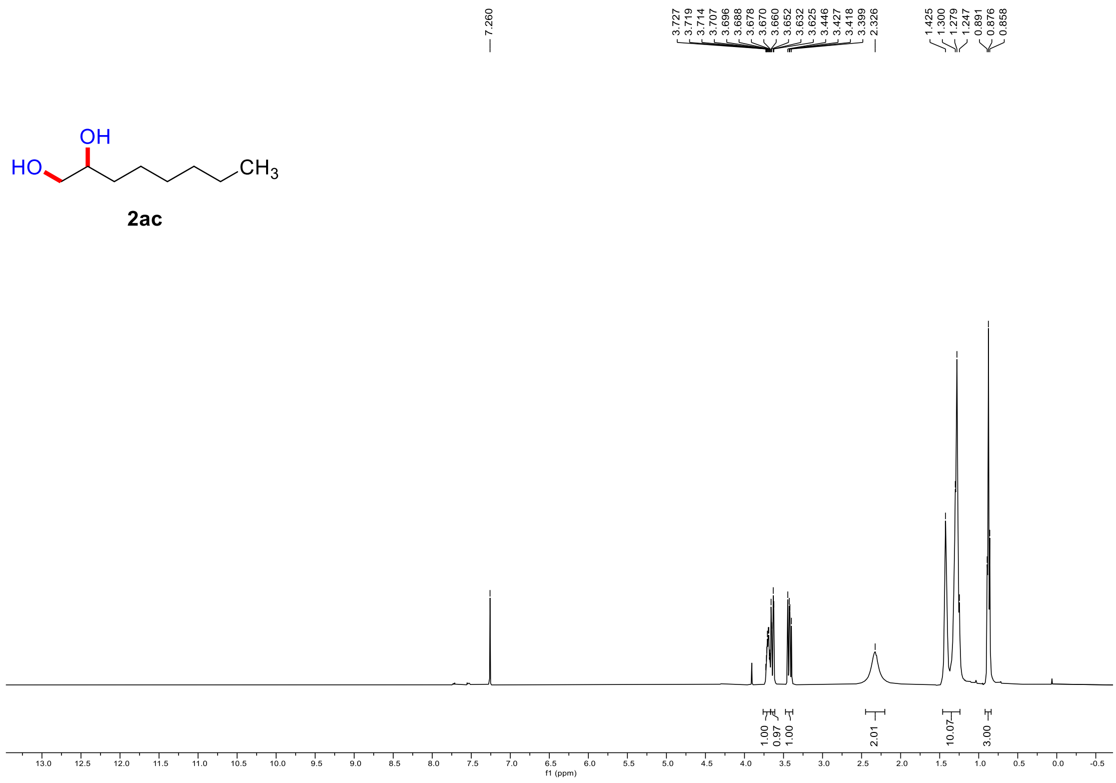


**Supplementary Figure 72**. $^{1}$H NMR of compound **2ac** (400 MHz, Chloroform-*d*)

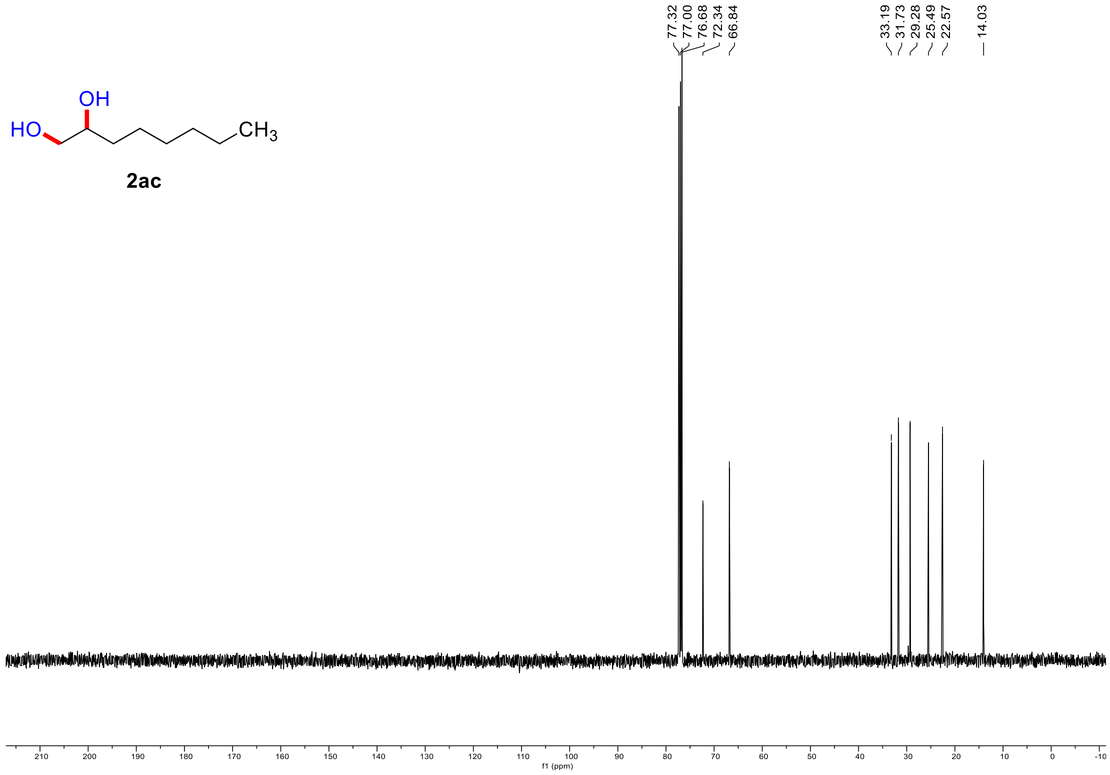


**Supplementary Figure 73**. $^{13}$C NMR of compound **2ac** (100 MHz, Chloroform-*d*)

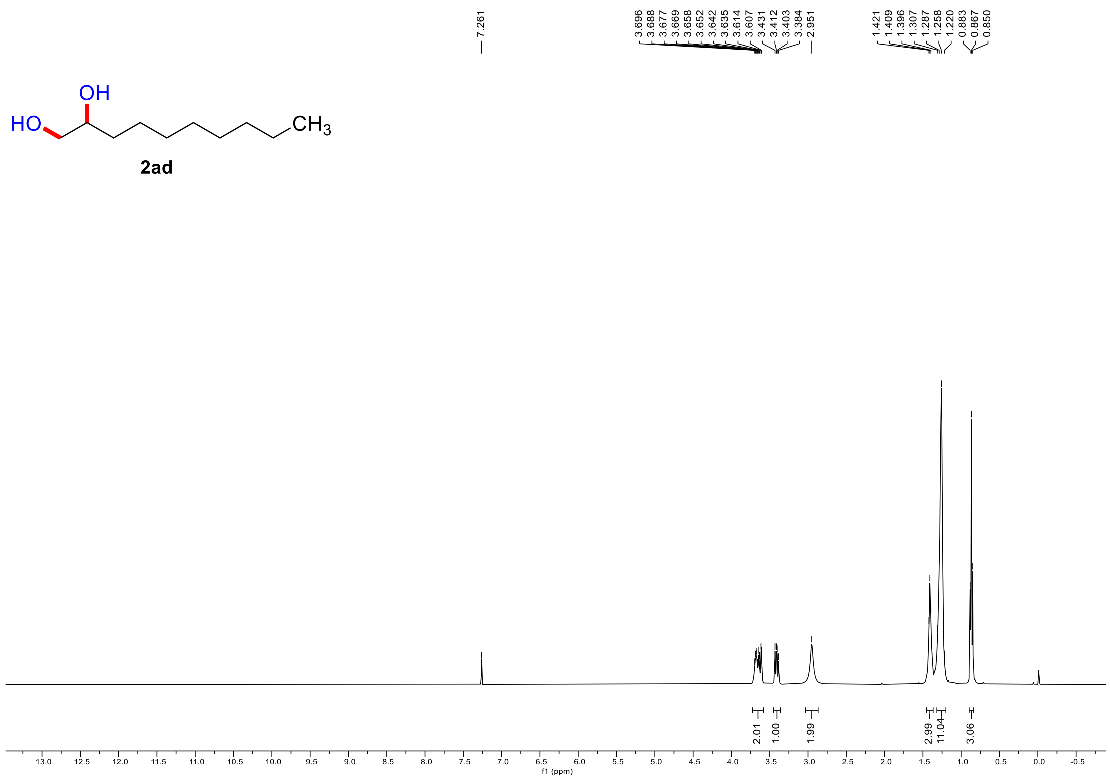


**Supplementary Figure 74**. $^{1}$H NMR of compound **2ad** (400 MHz, Chloroform-*d*)

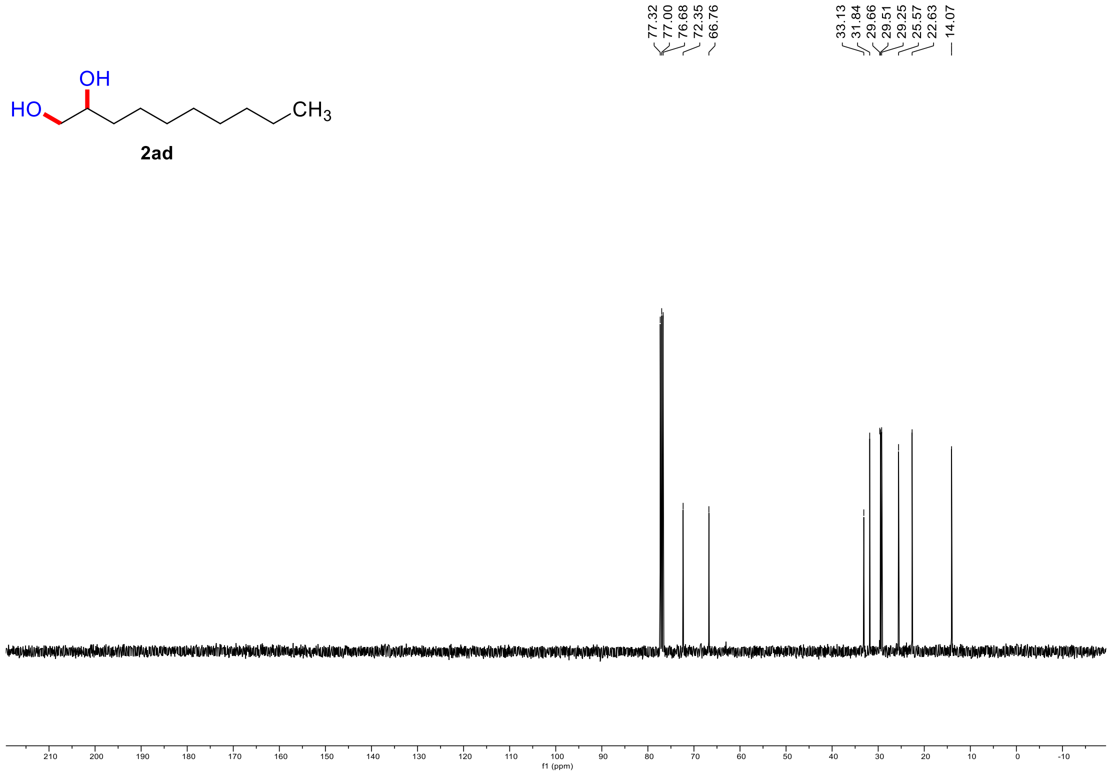


**Supplementary Figure 75**. $^{13}$C NMR of compound **2ad** (100 MHz, Chloroform-*d*)

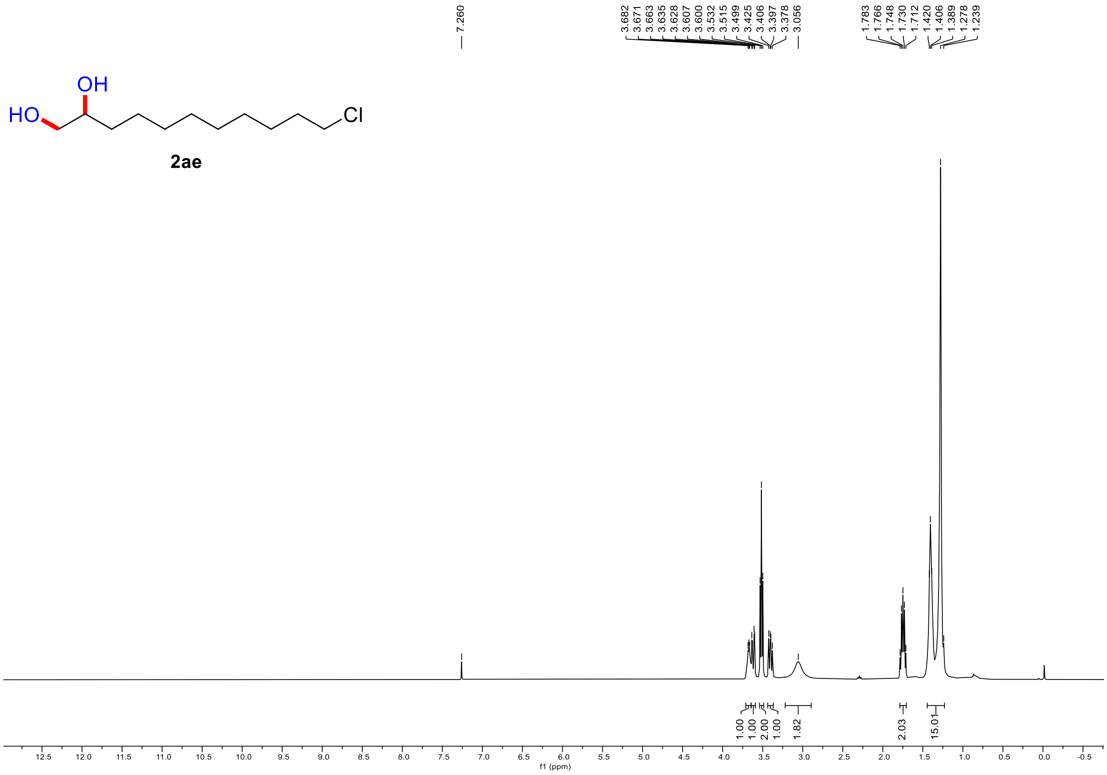


**Supplementary Figure 76**. $^{1}H$ NMR of compound **2ae** (400 MHz, Chloroform-*d*)

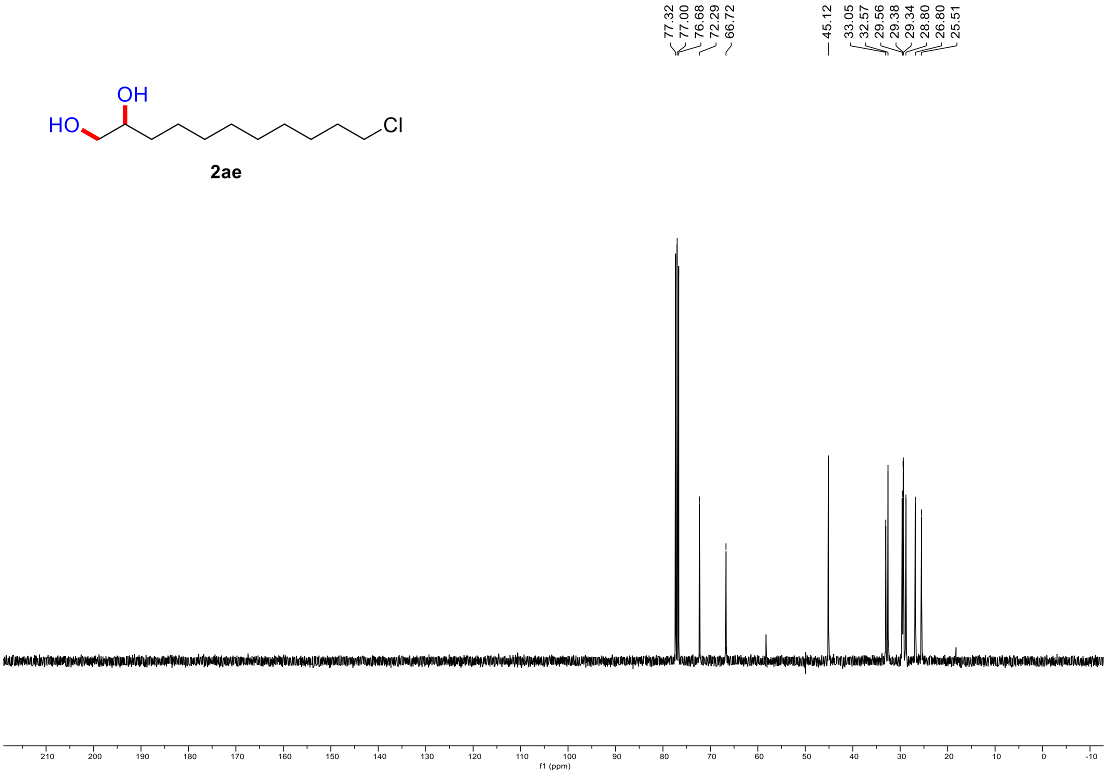


**Supplementary Figure 77**. $^{13}C$ NMR of compound **2ae** (100 MHz, Chloroform-*d*)

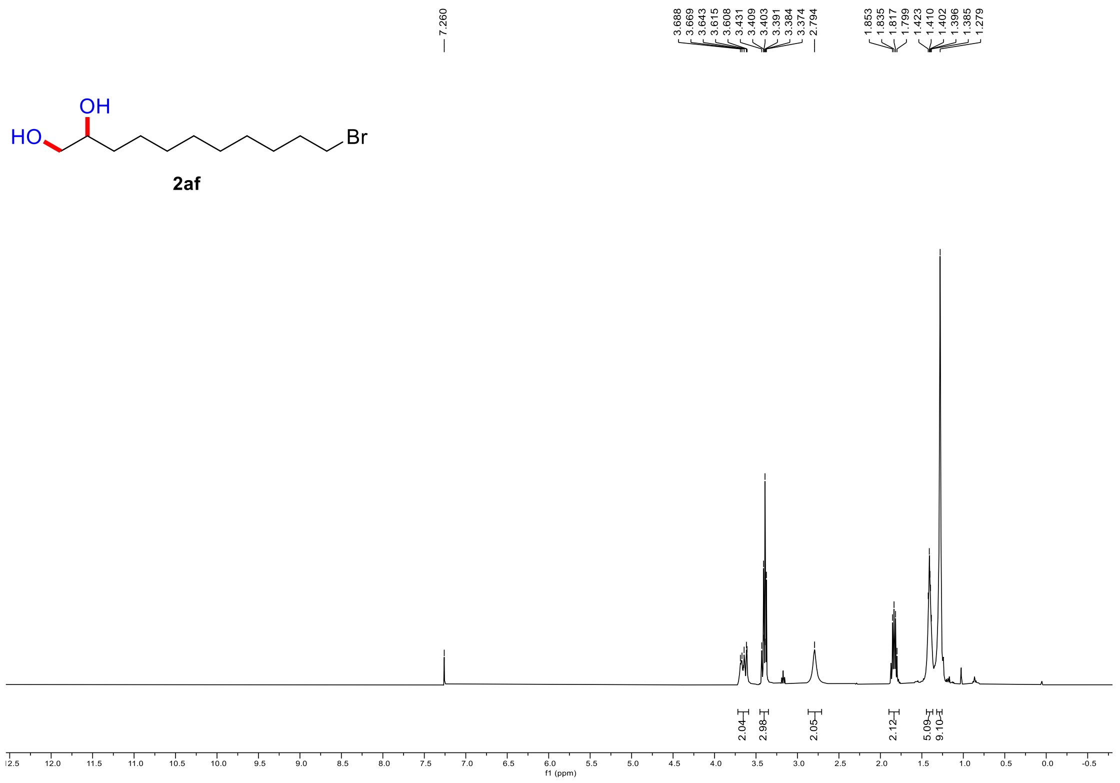


**Supplementary Figure 78**. $^{1}H$ NMR of compound **2af** (400 MHz, Chloroform-*d*)

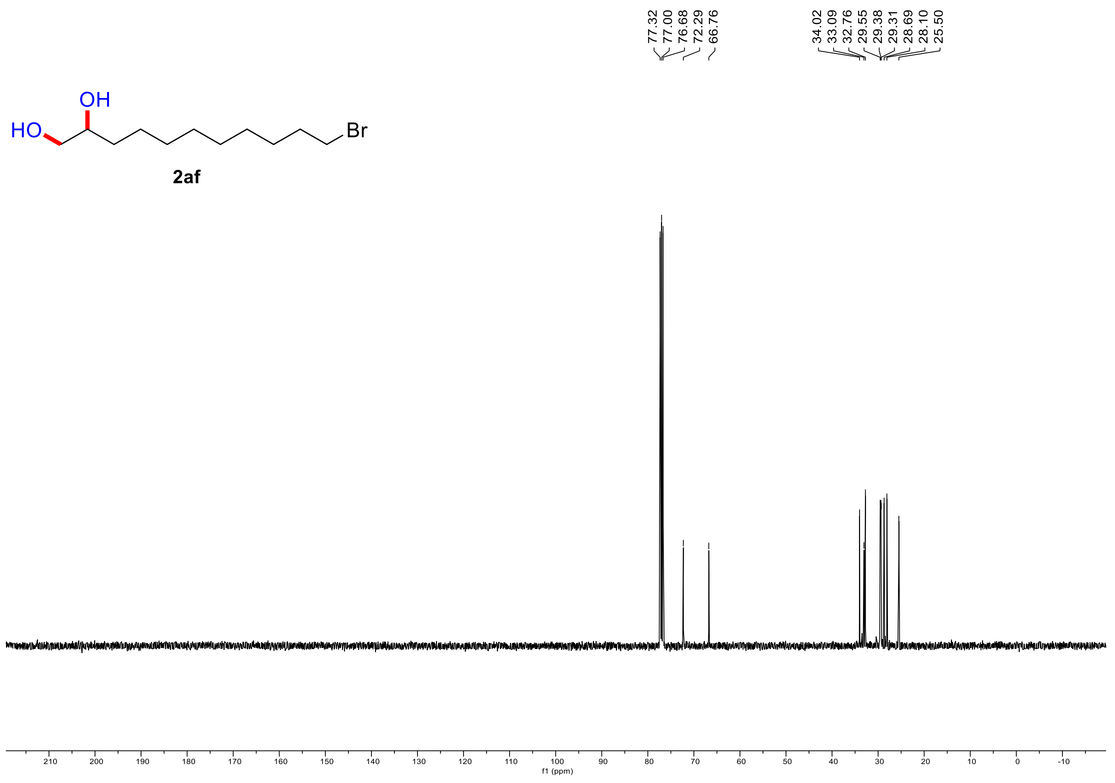


**Supplementary Figure 79**. $^{13}C$ NMR of compound **2af** (100 MHz, Chloroform-*d*)

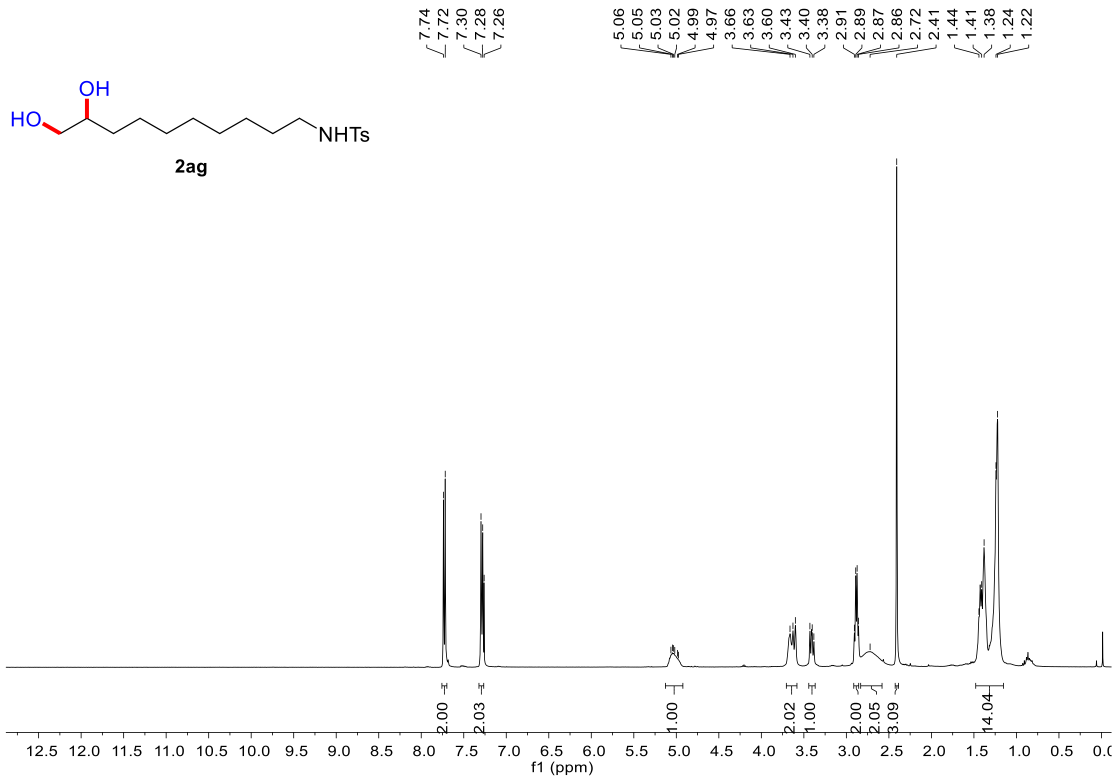

**Supplementary Figure 80**. $^{1}$H NMR of compound **2ag** (400 MHz, Chloroform-*d*)

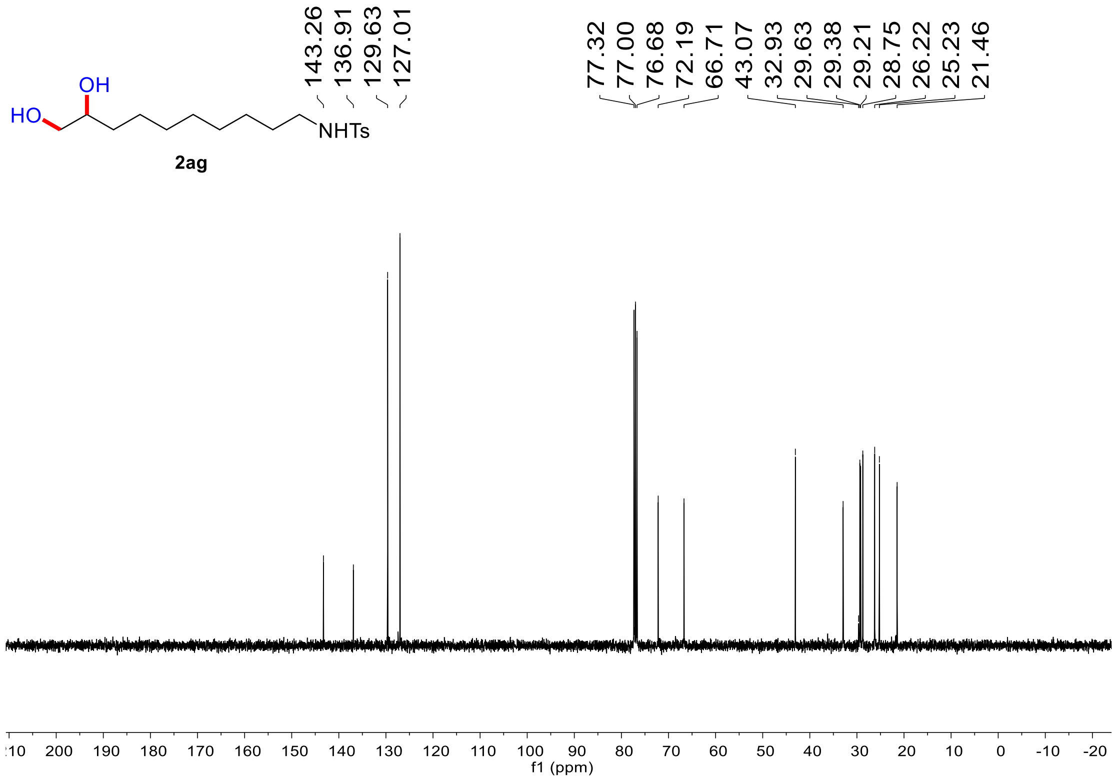

**Supplementary Figure 81**. $^{13}$C NMR of compound **2ag** (100 MHz, Chloroform-*d*)

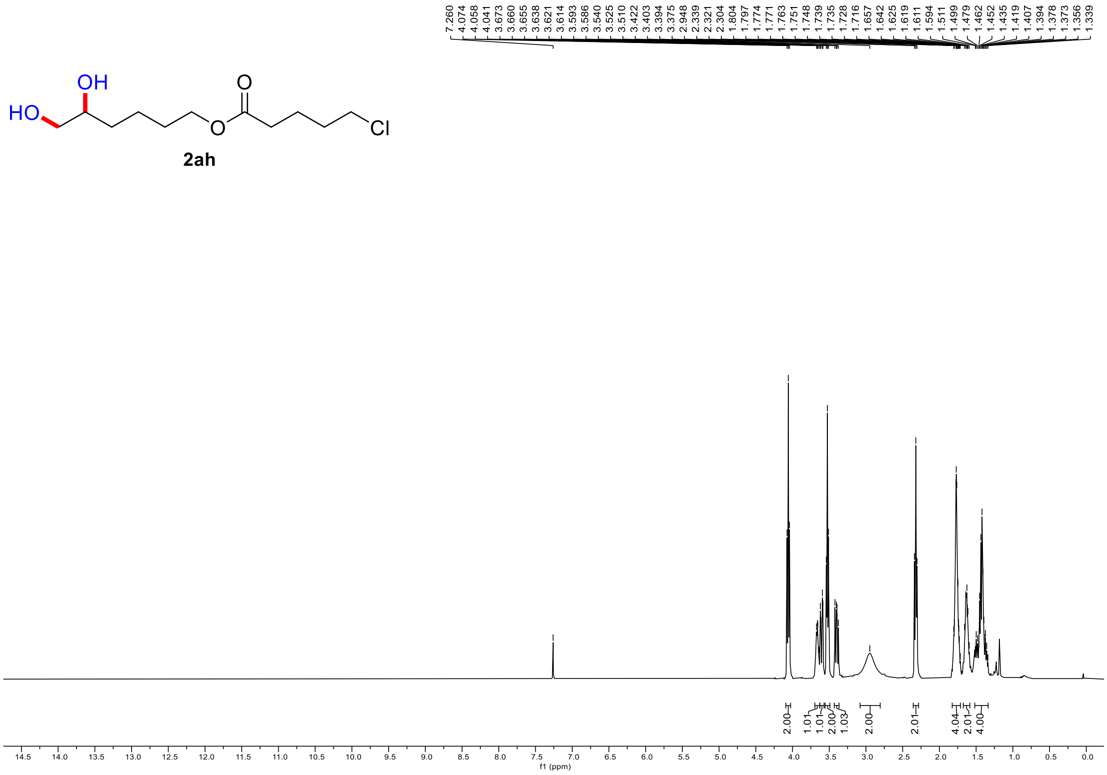


**Supplementary Figure 82**. $^{1}$H NMR of compound **2ah** (400 MHz, Chloroform-*d*)

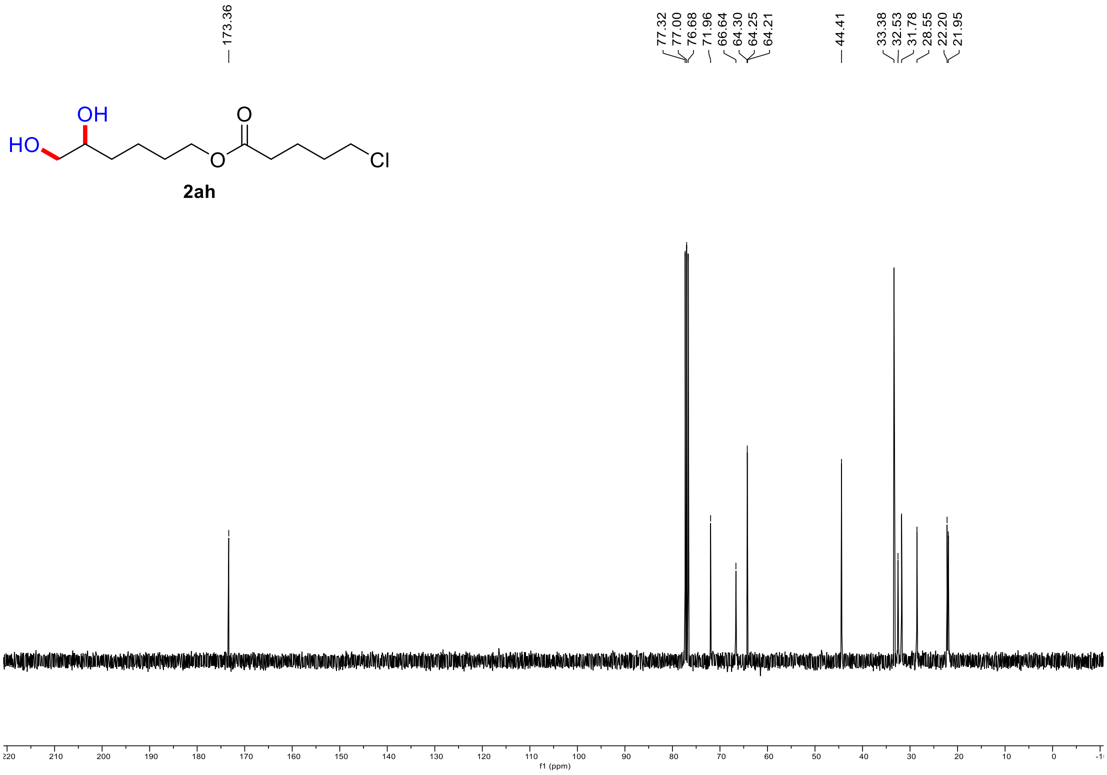


**Supplementary Figure 83**. $^{13}$C NMR of compound **2ah** (100 MHz, Chloroform-*d*)

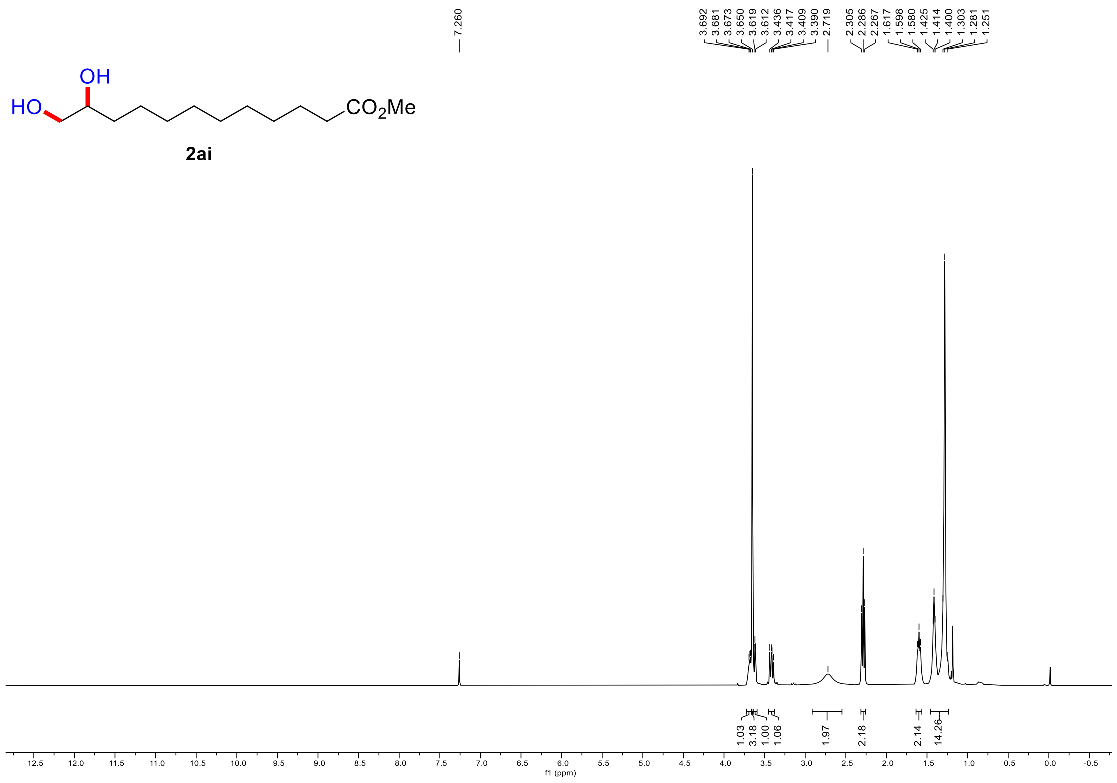


**Supplementary Figure 84**. $^{1}$H NMR of compound **2ai** (400 MHz, Chloroform-*d*)

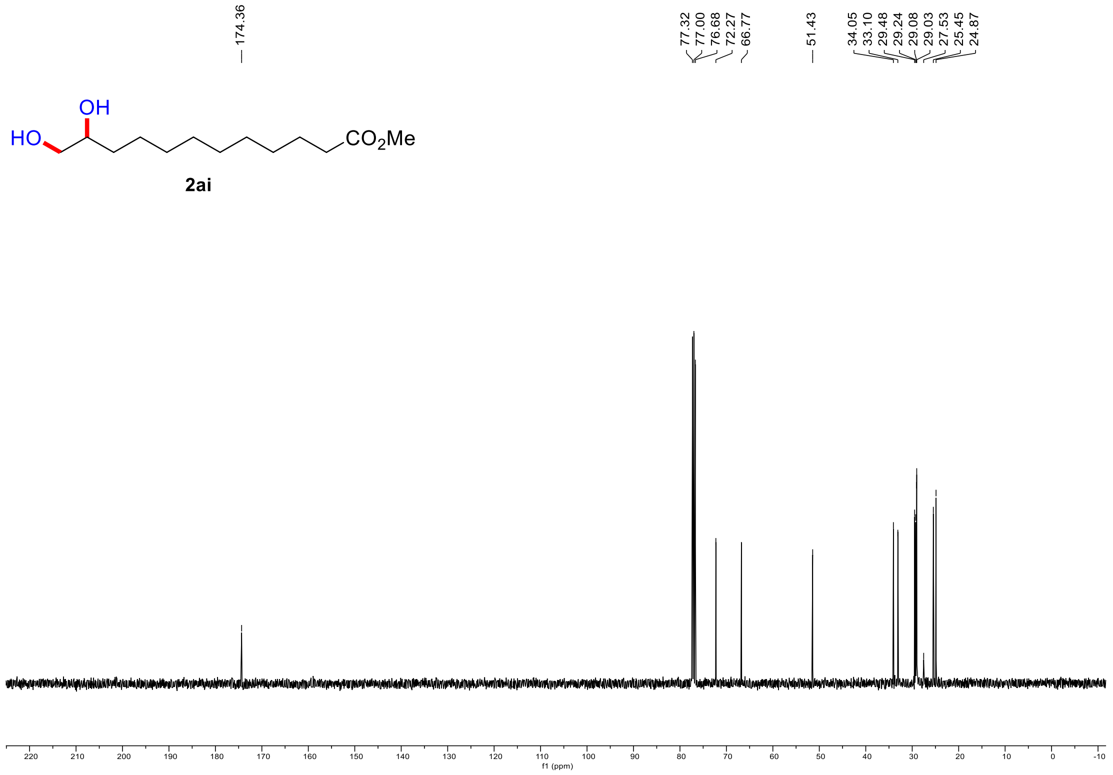


**Supplementary Figure 85**. $^{13}$C NMR of compound **2ai** (100 MHz, Chloroform-*d*)

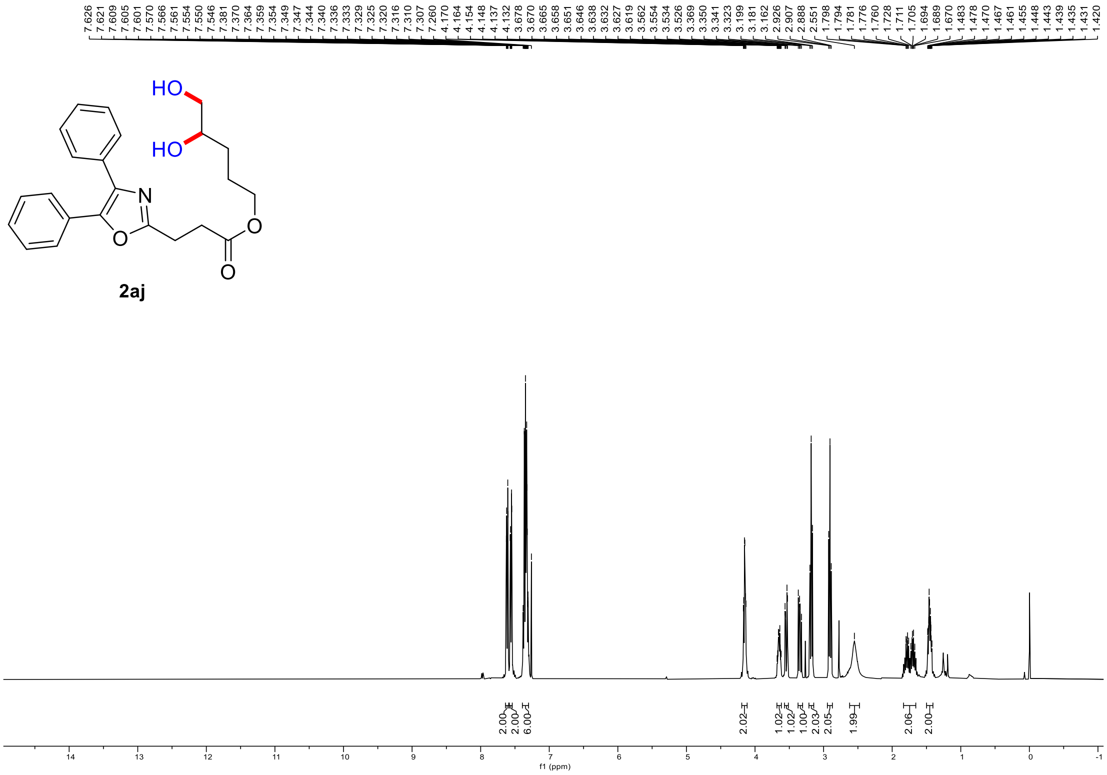


**Supplementary Figure 86**. $^{1}$H NMR of compound **2aj** (400 MHz, Chloroform-*d*)

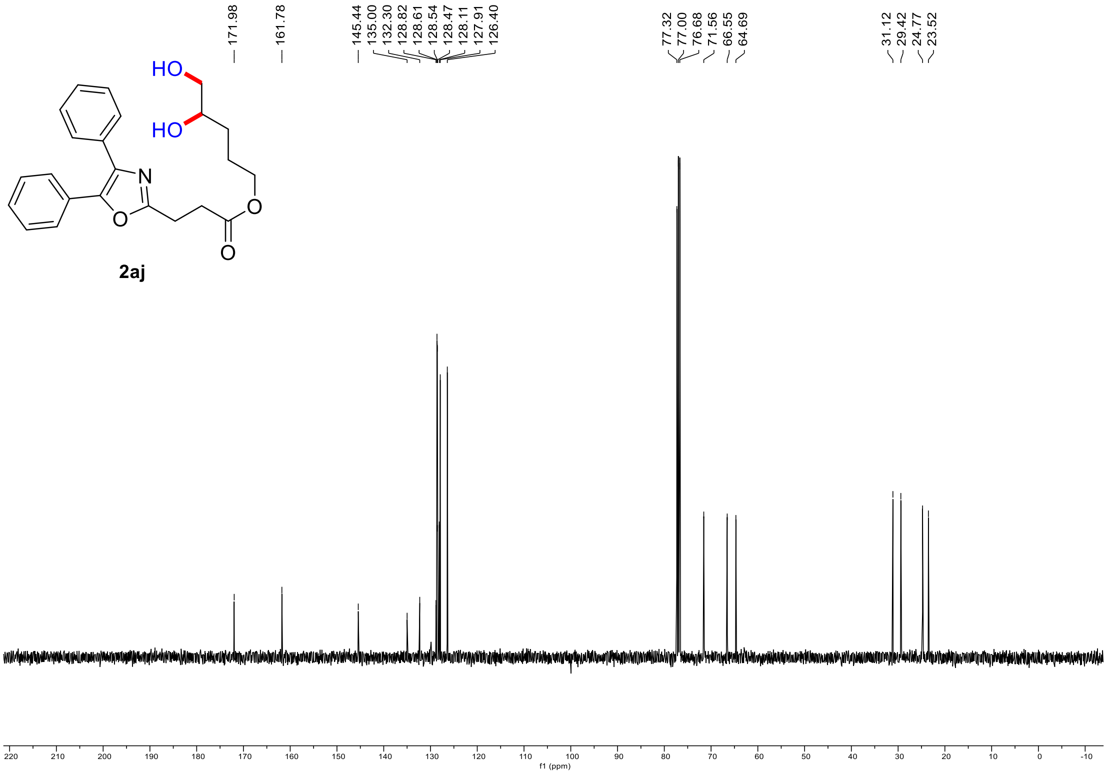


**Supplementary Figure 87**. $^{13}$C NMR of compound **2aj** (100 MHz, Chloroform-*d*)

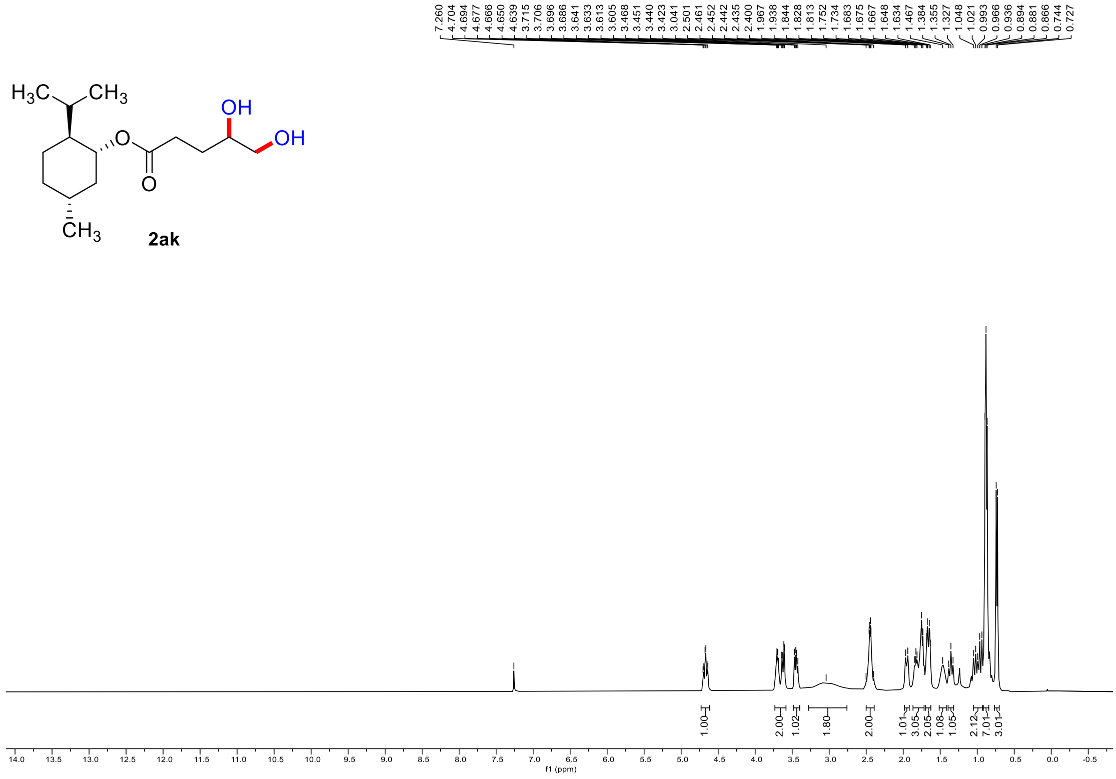


**Supplementary Figure 88**. $^{1}$H NMR of compound **2ak** (400 MHz, Chloroform-*d*)

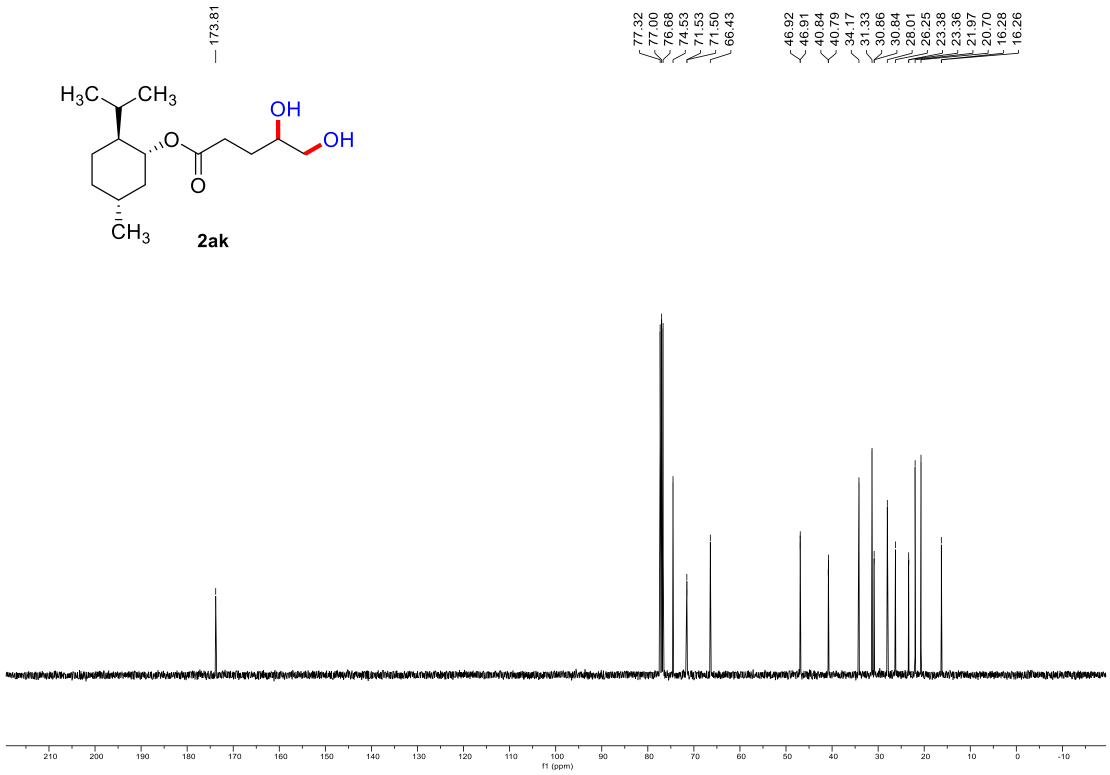


**Supplementary Figure 89**. $^{13}$C NMR of compound **2ak** (100 MHz, Chloroform-*d*)

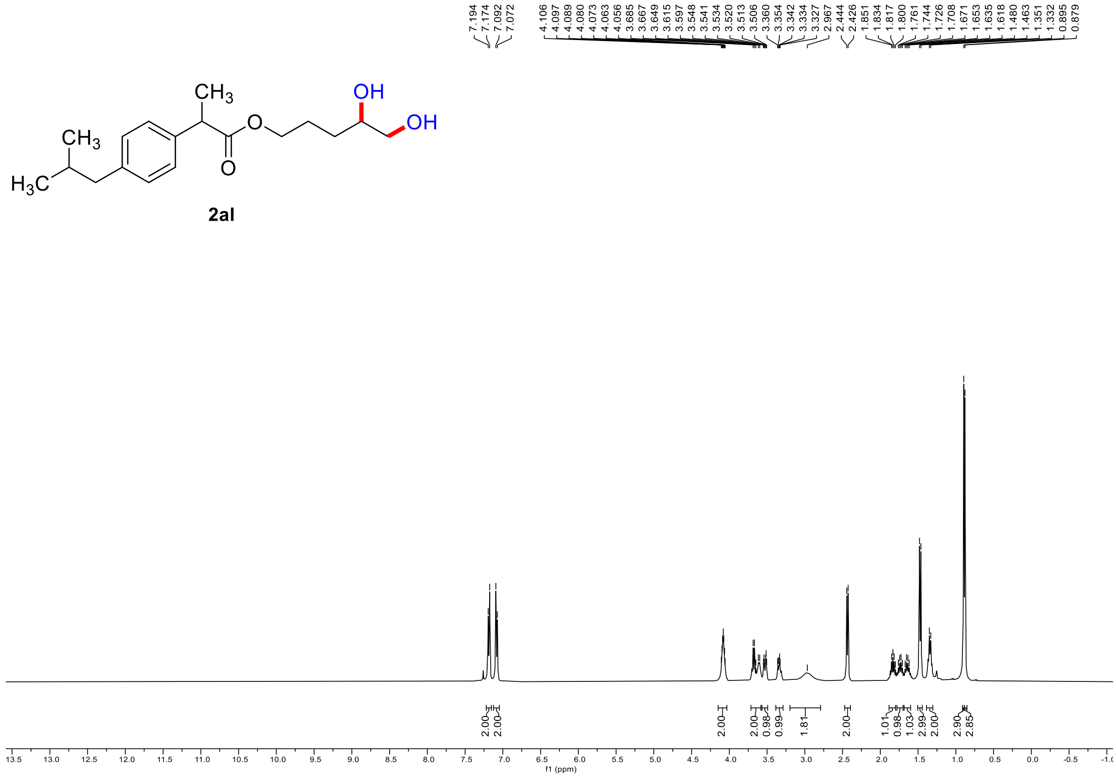


**Supplementary Figure 90**. $^{1}$H NMR of compound **2al** (400 MHz, Chloroform-*d*)

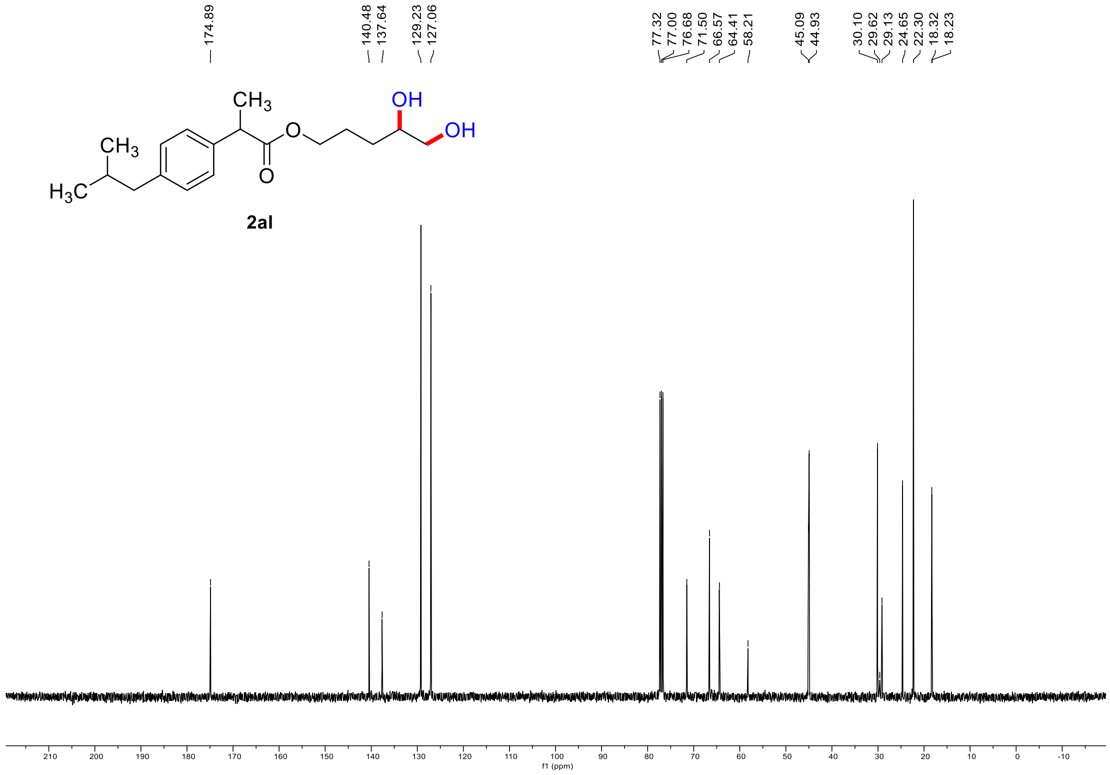


**Supplementary Figure 91**. $^{13}$C NMR of compound **2al** (100 MHz, Chloroform-*d*)

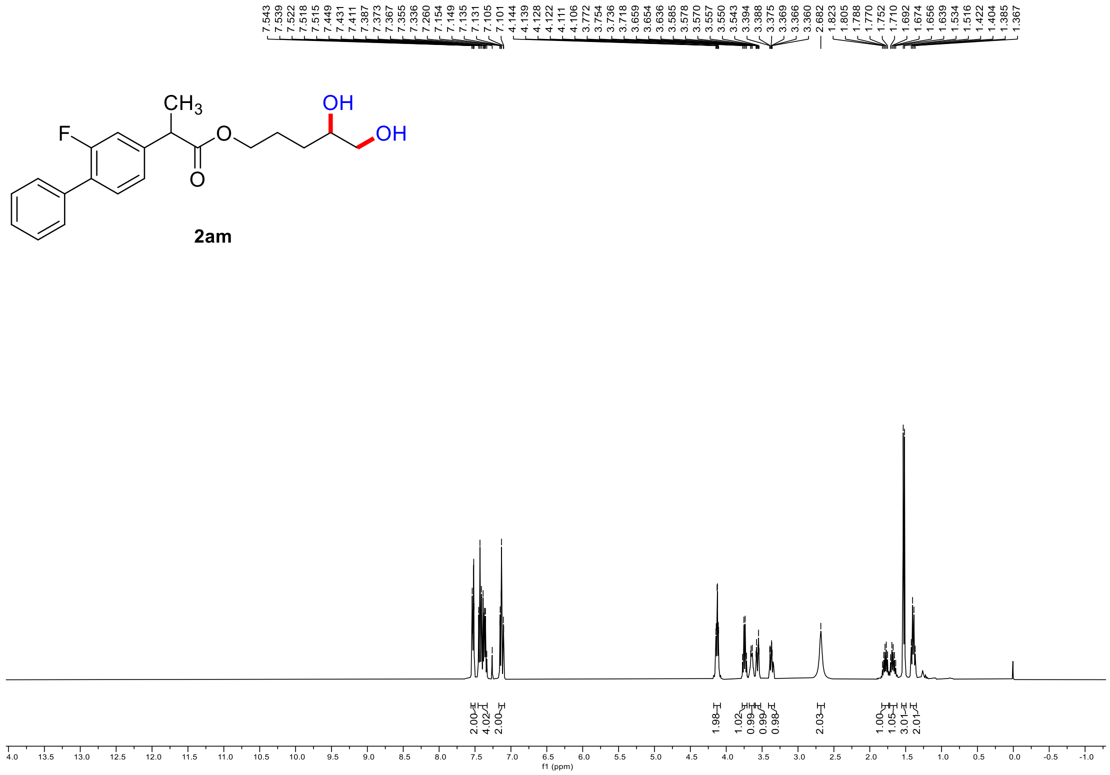


**Supplementary Figure 92**. $^{1}$H NMR of compound **2am** (400 MHz, Chloroform-*d*)

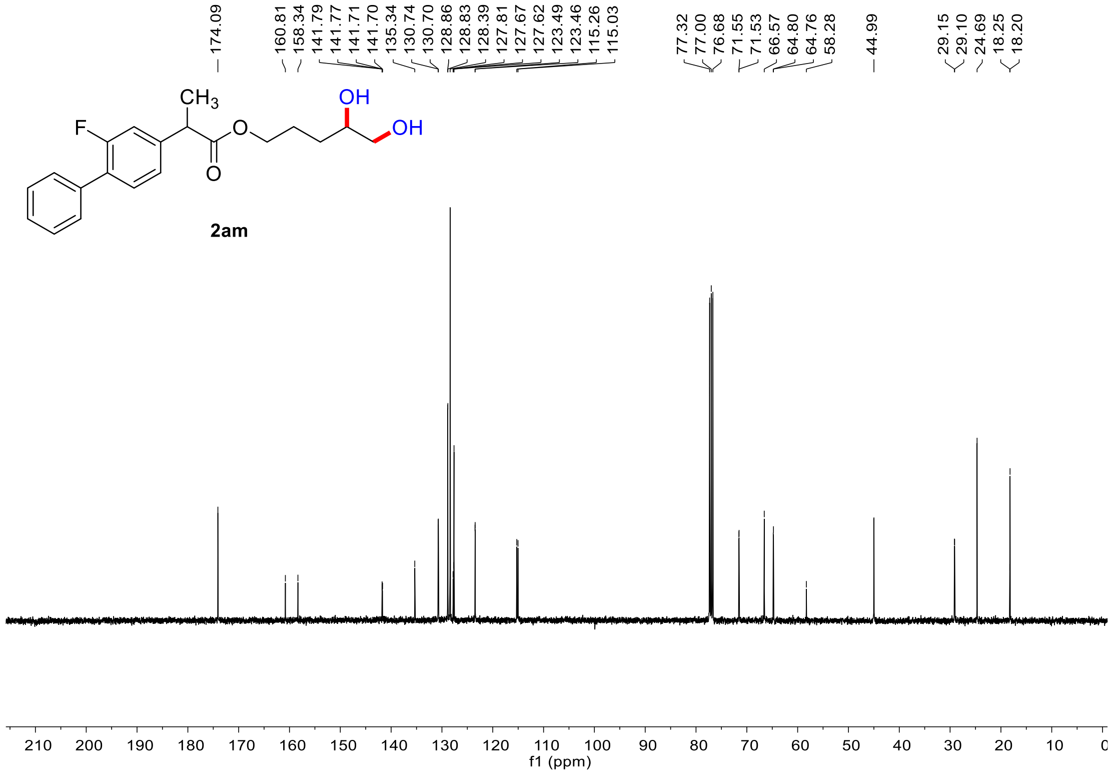


**Supplementary Figure 93**. $^{13}$C NMR of compound **2am** (100 MHz, Chloroform-*d*)

**$^{19}F$ NMR of Compound 2am (375 MHz, $CDCl_3$):**

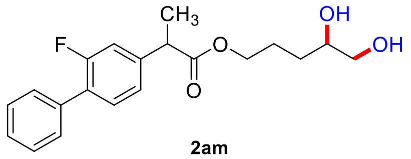

**2am**

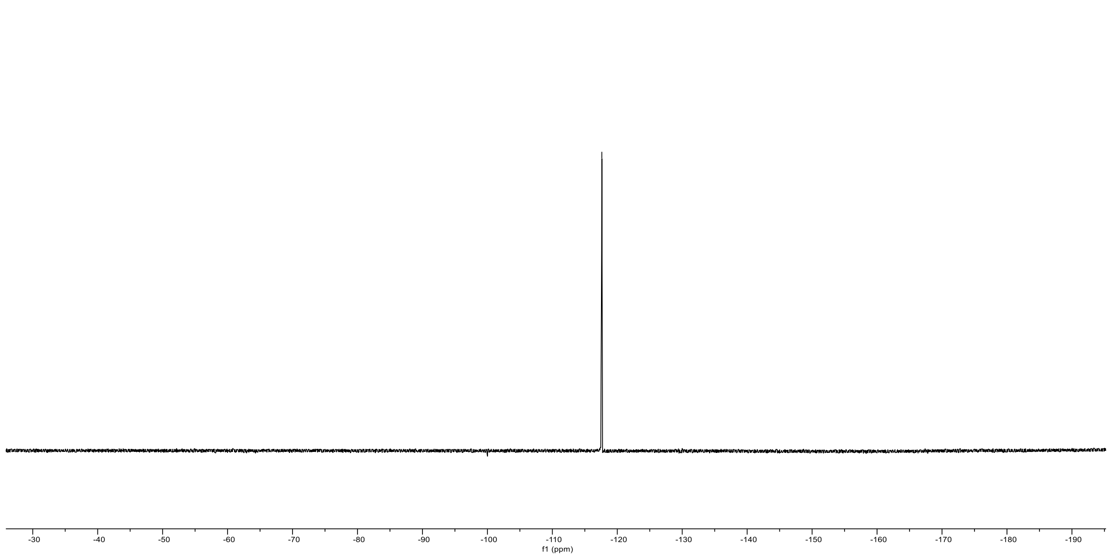


**Supplementary Figure 94**. $^{19}F$ NMR of compound **2am** (375 MHz, Chloroform-*d*)

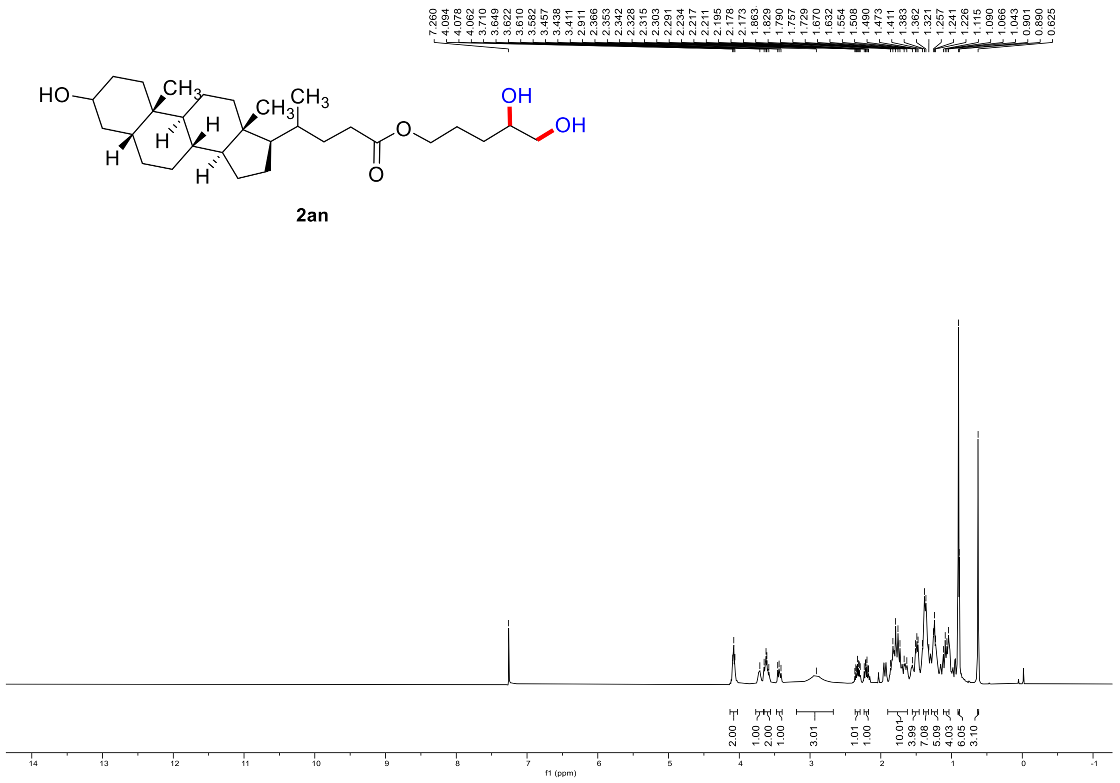


**Supplementary Figure 95**. $^{1}$H NMR of compound **2an** (400 MHz, Chloroform-*d*)

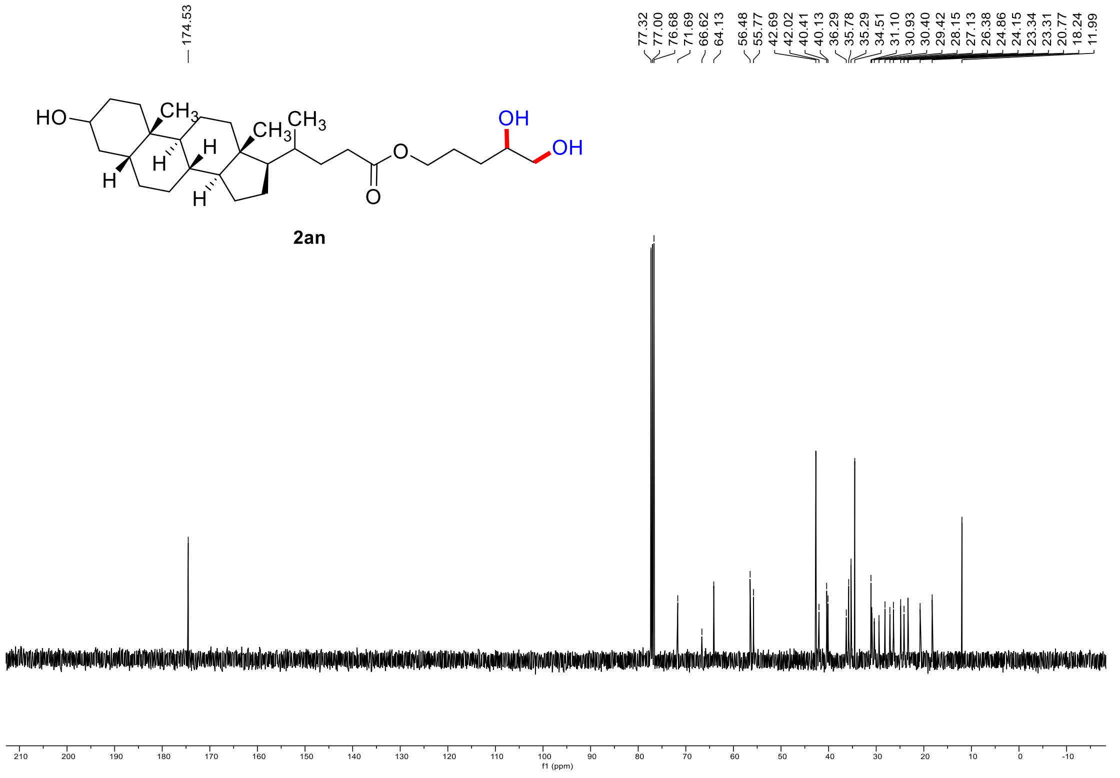


**Supplementary Figure 96**. $^{13}$C NMR of compound **2an** (100 MHz, Chloroform-*d*)

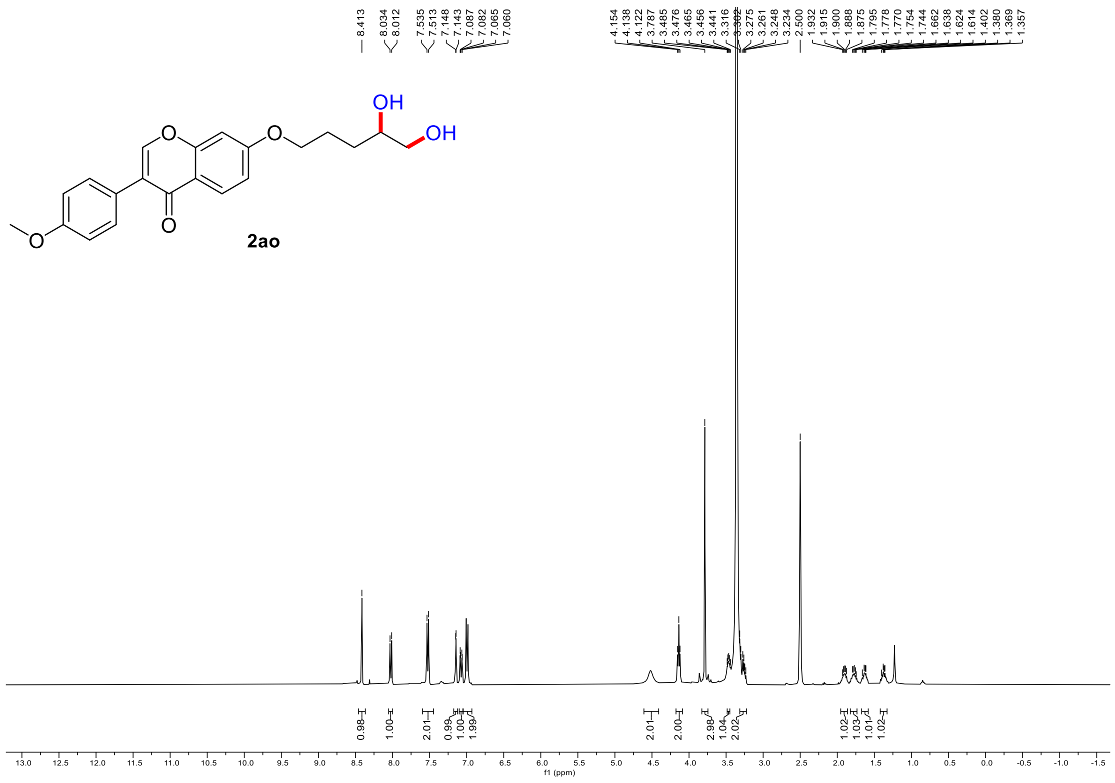

**Supplementary Figure 97**. $^{1}$H NMR of compound **2ao** (400 MHz, Chloroform-*d*)

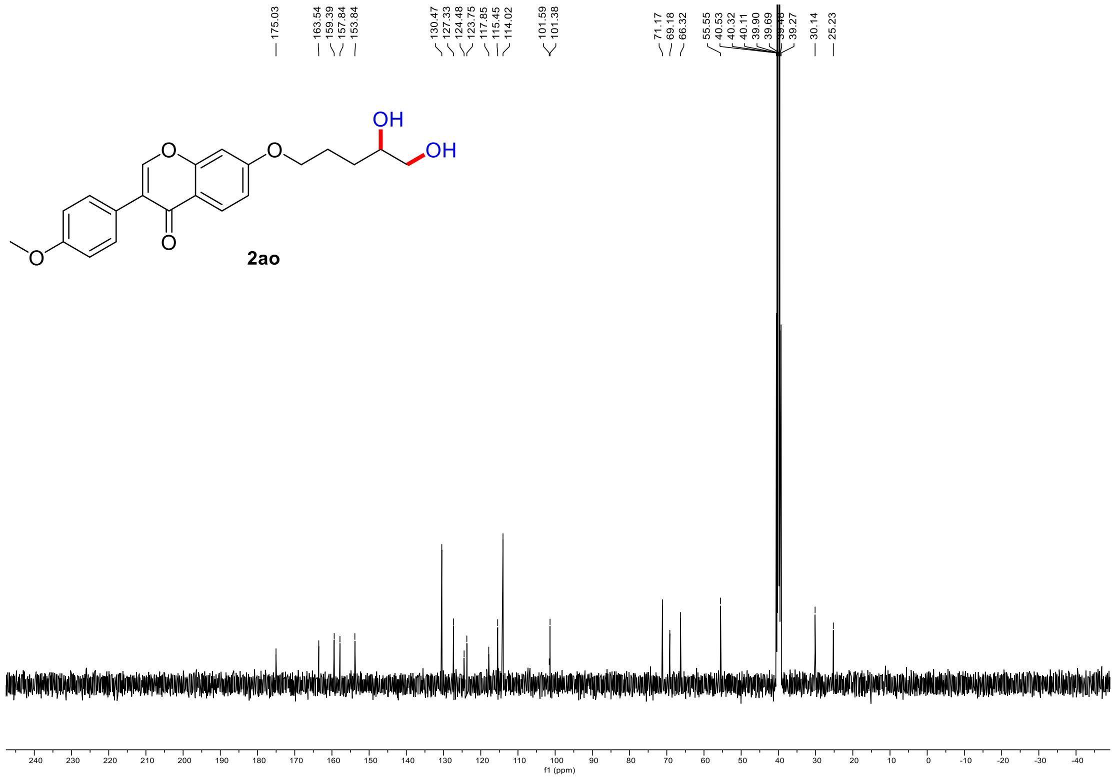

**Supplementary Figure 98**. $^{13}$C NMR of compound **2ao** (100 MHz, Chloroform-*d*)

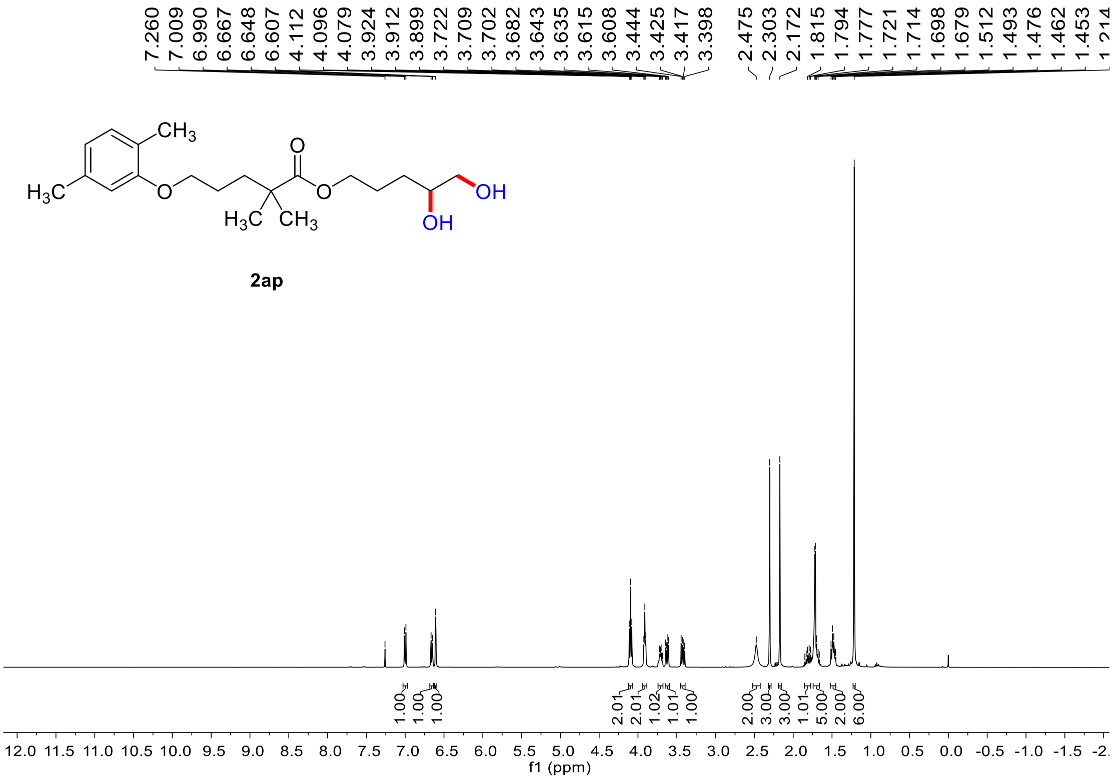

**Supplementary Figure 99**. $^{1}H$ NMR of compound **2ap** (400 MHz, Chloroform-*d*)

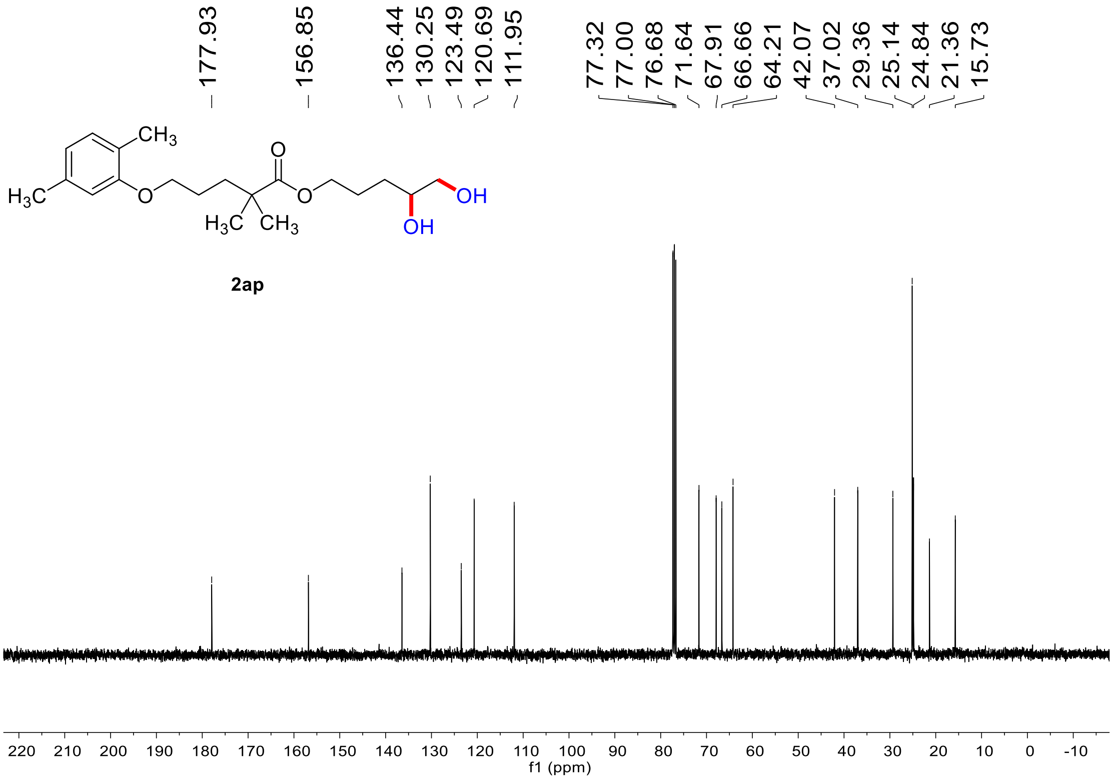

**Supplementary Figure 100**. $^{13}C$ NMR of compound **2ap** (100 MHz, Chloroform-*d*)

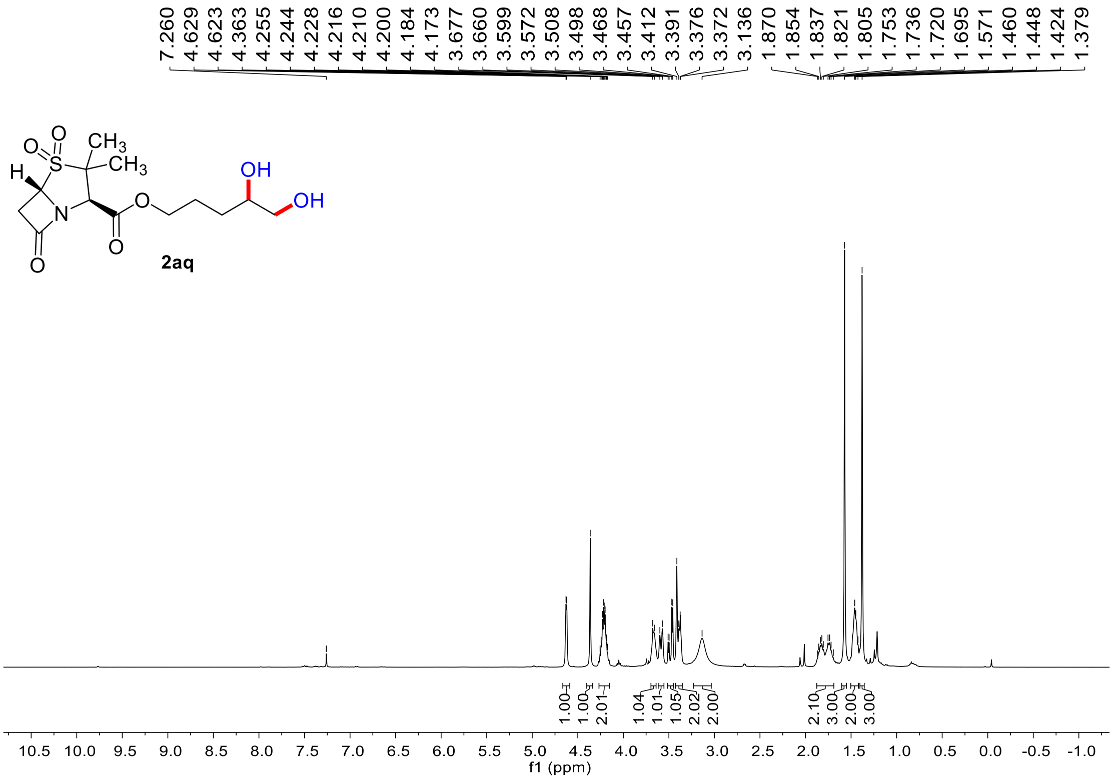


**Supplementary Figure 101**. $^{1}$H NMR of compound **2aq** (400 MHz, Chloroform-*d*)

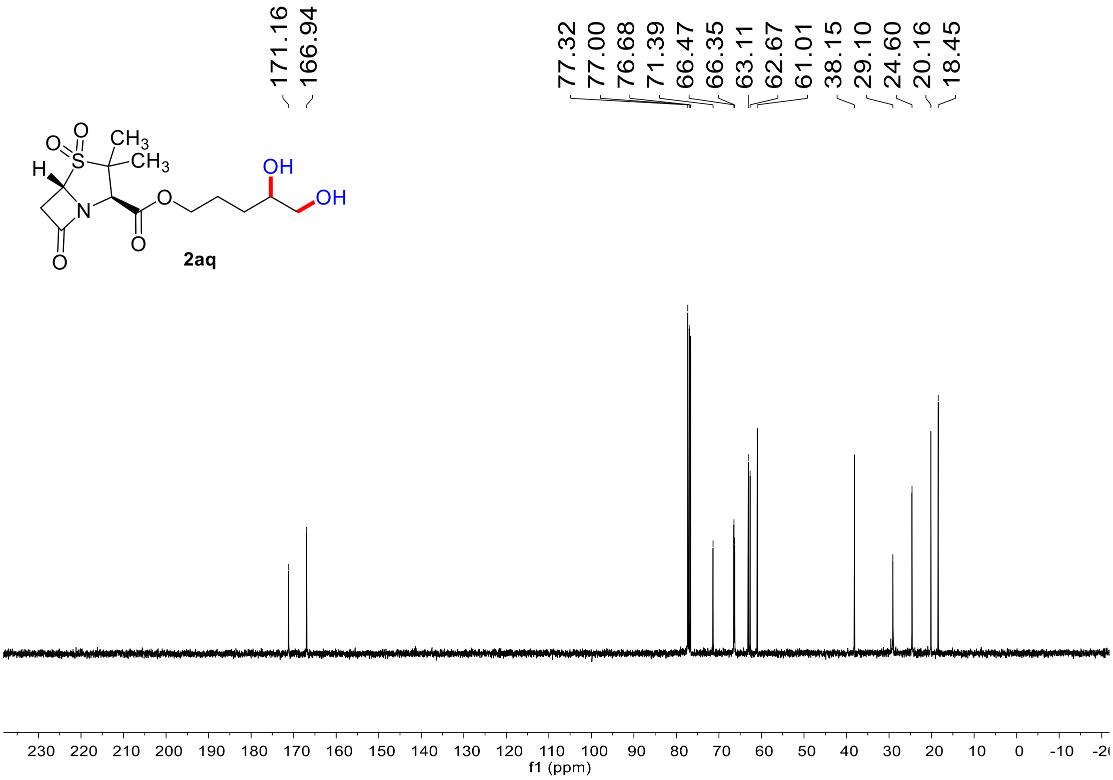


**Supplementary Figure 102**. $^{13}$C NMR of compound **2aq** (100 MHz, Chloroform-*d*)

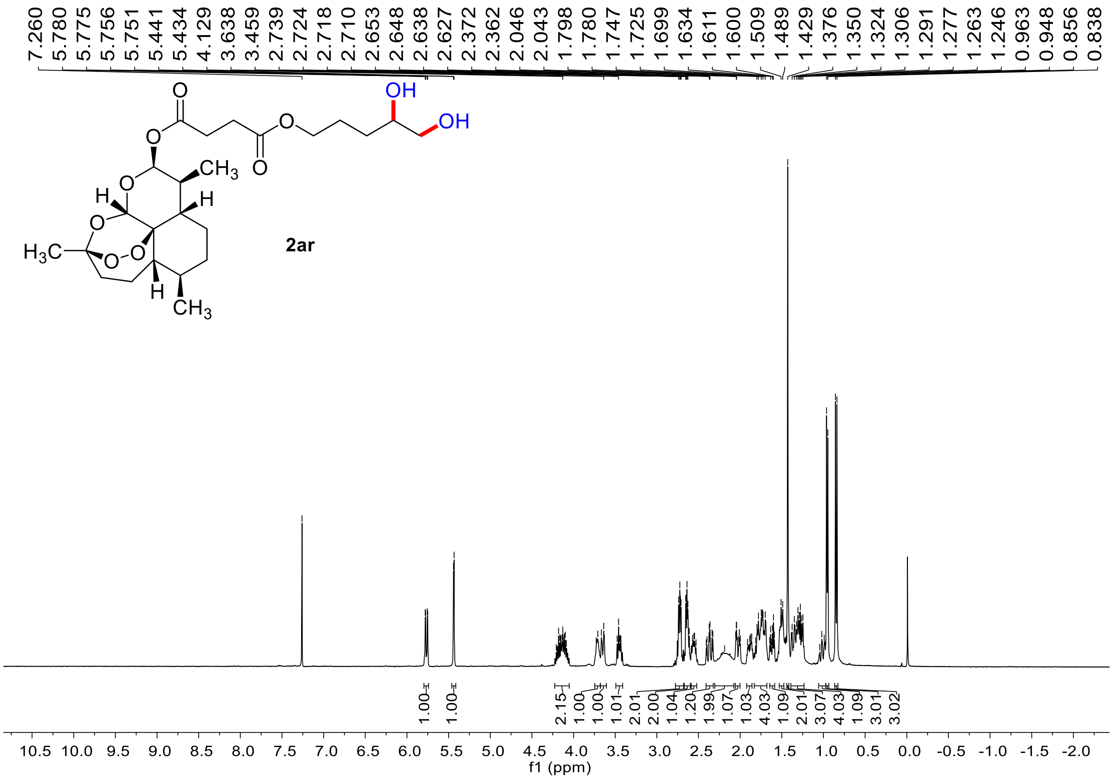


**Supplementary Figure 103**. $^{1}H$ NMR of compound **2ar** (400 MHz, Chloroform-*d*)

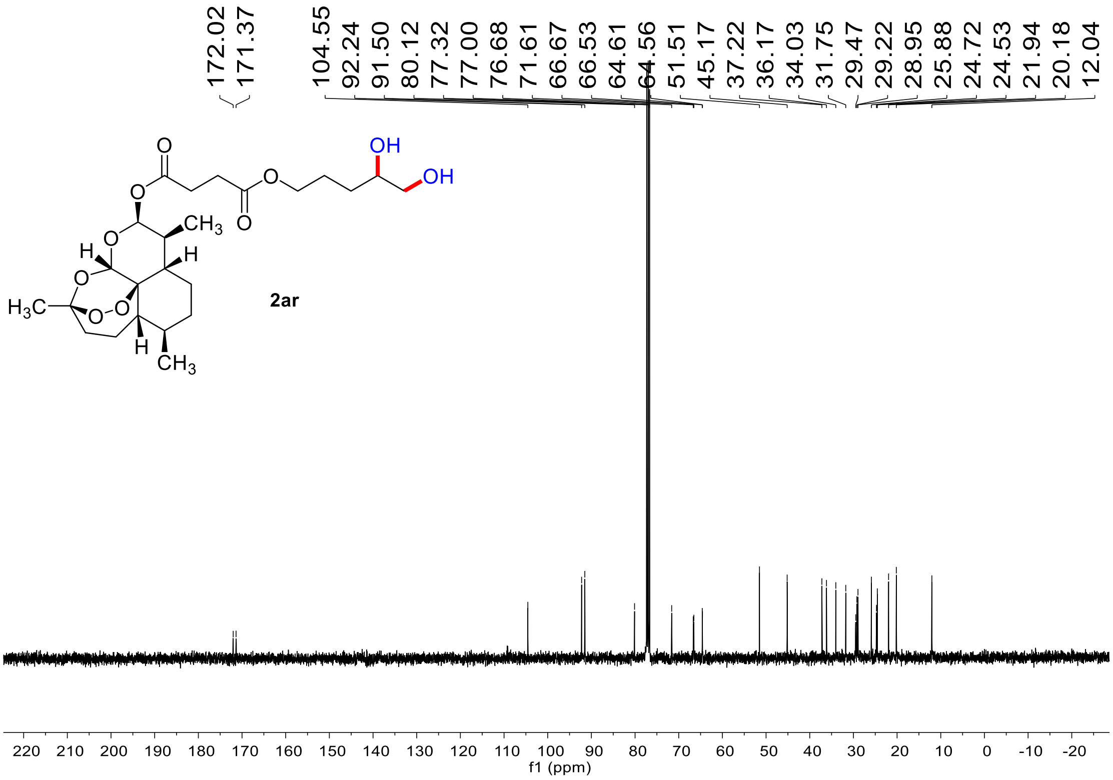


**Supplementary Figure 104**. $^{13}C$ NMR of compound **2ar** (100 MHz, Chloroform-*d*)

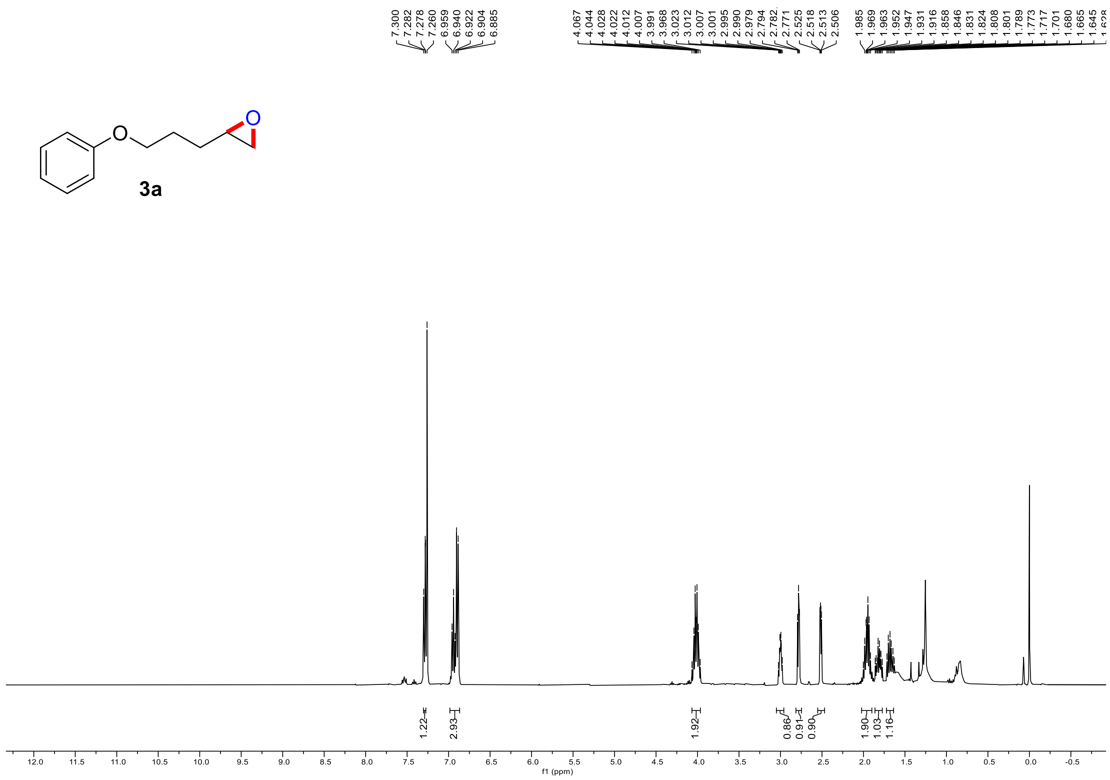

**Supplementary Figure 105**. $^{1}$H NMR of compound **3a** (400 MHz, Chloroform-*d*)

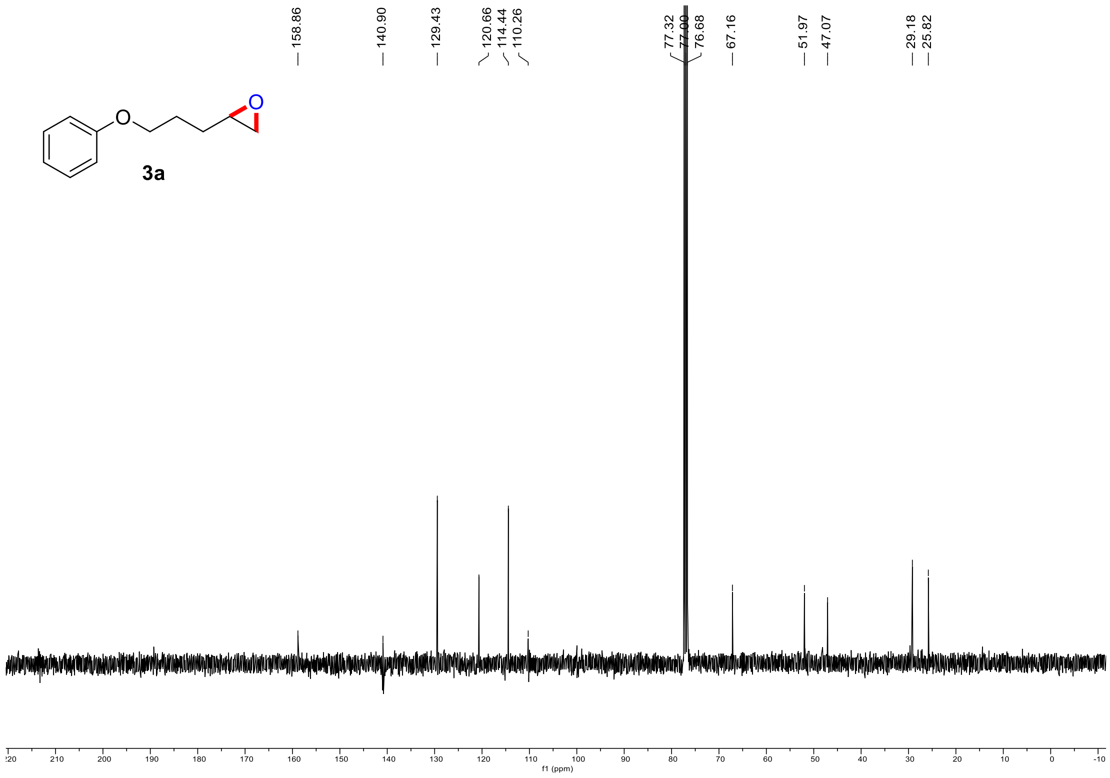

**Supplementary Figure 106**. $^{13}$C NMR of compound **3a** (100 MHz, Chloroform-*d*)

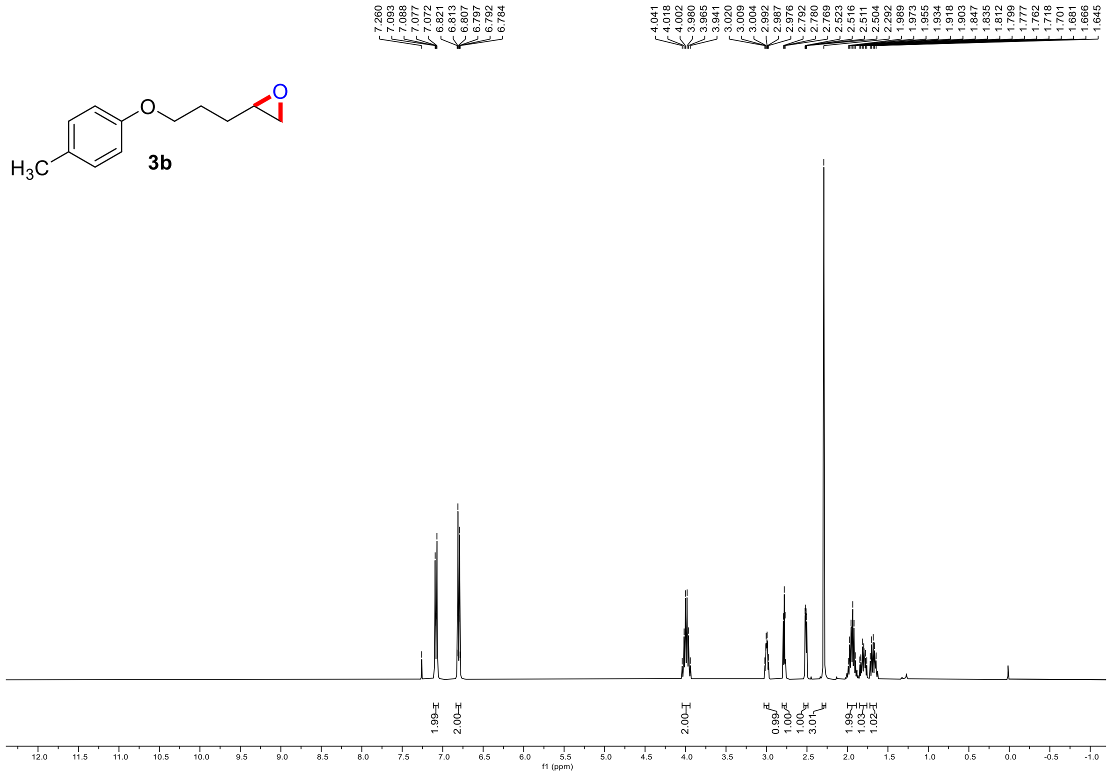


**Supplementary Figure 107**. $^{1}$H NMR of compound **3b** (400 MHz, Chloroform-*d*)

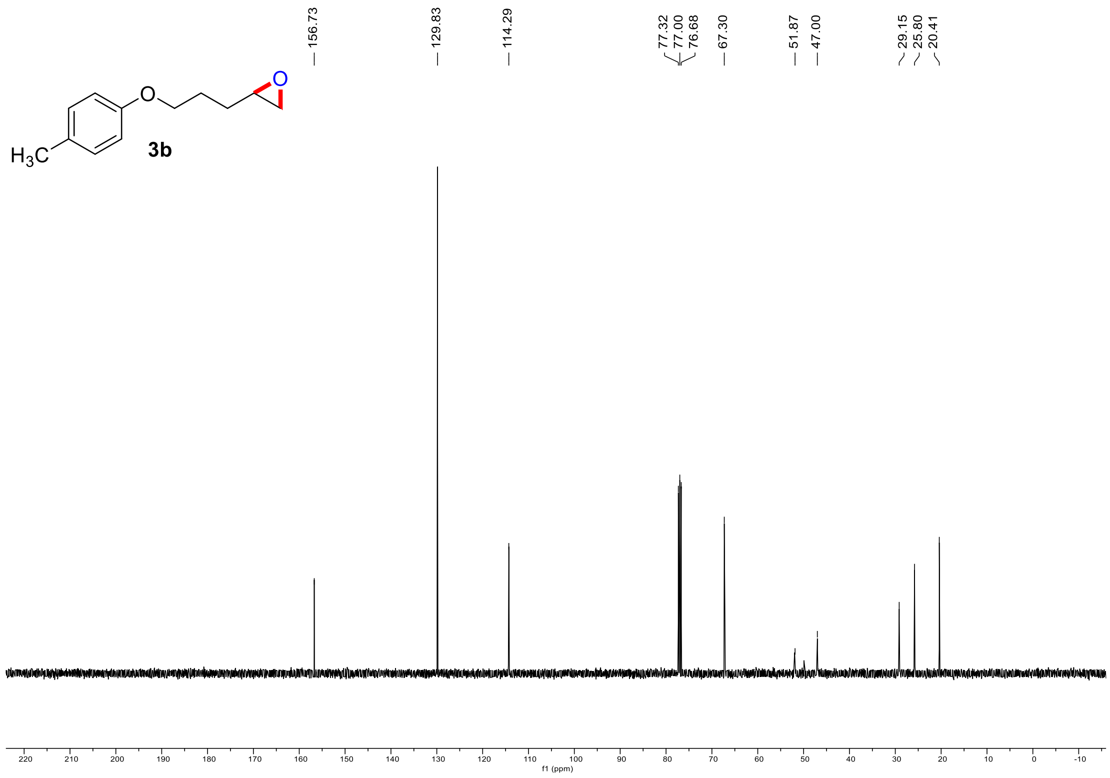


**Supplementary Figure 108**. $^{13}$C NMR of compound **3b** (100 MHz, Chloroform-*d*)

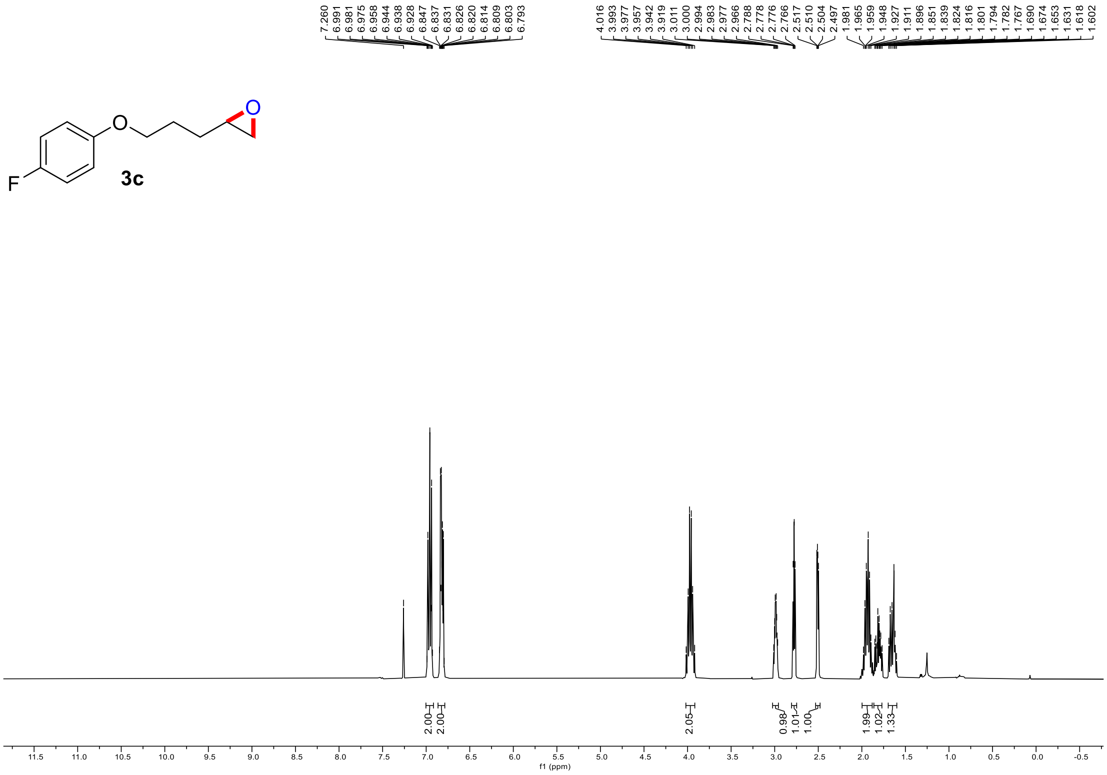


**Supplementary Figure 109**. $^{1}$H NMR of compound **3c** (400 MHz, Chloroform-*d*)

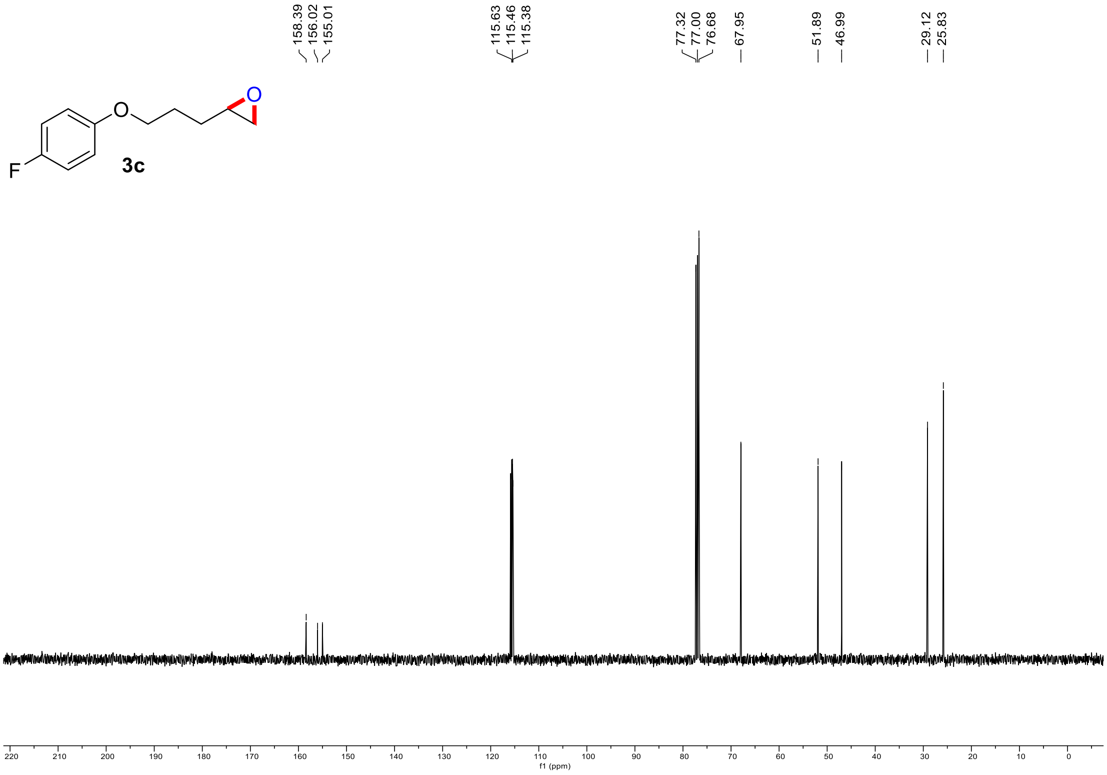


**Supplementary Figure 110**. $^{13}$C NMR of compound **3c** (100 MHz, Chloroform-*d*)

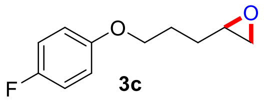


**3c**

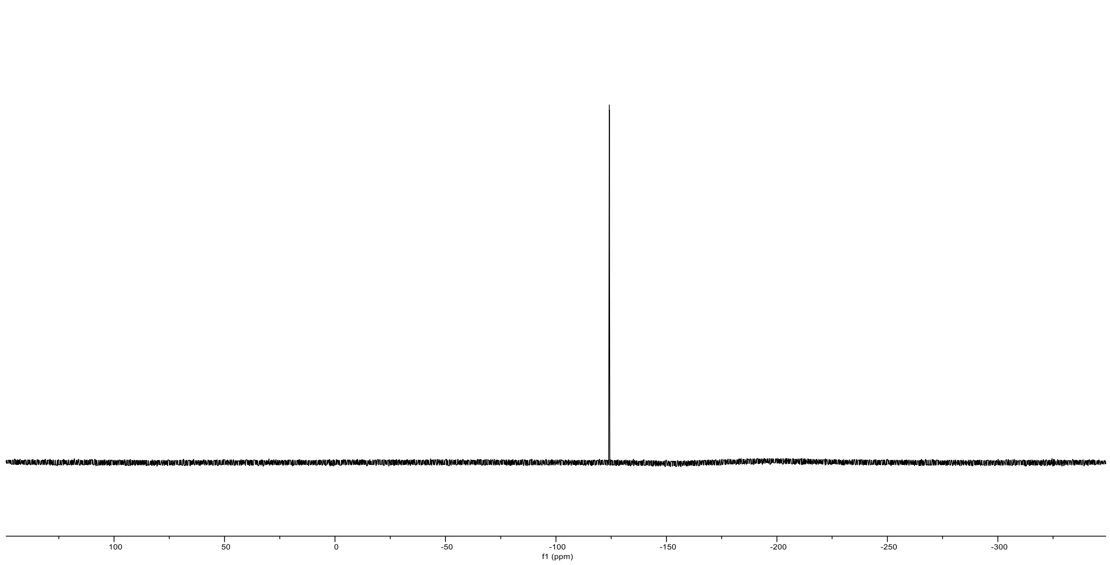


**Supplementary Figure 111**. $^{19}$F NMR of compound **3c** (375 MHz, Chloroform-*d*)

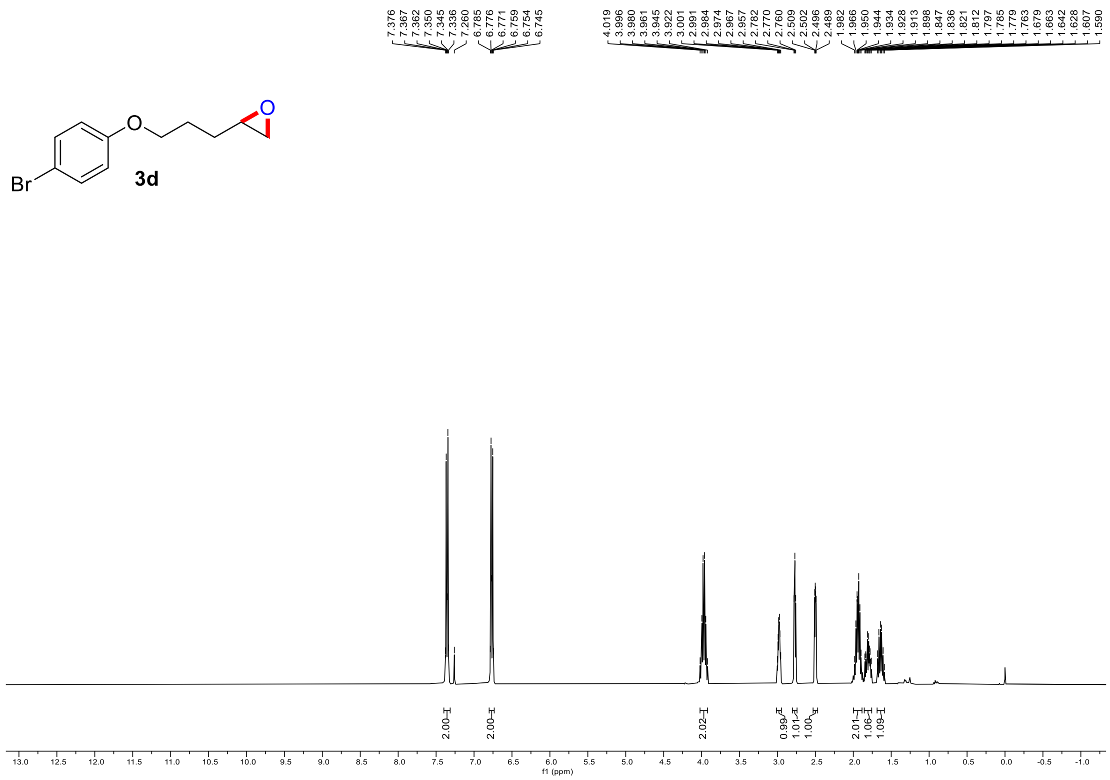

**Supplementary Figure 112**. $^{1}$H NMR of compound **3d** (400 MHz, Chloroform-*d*)

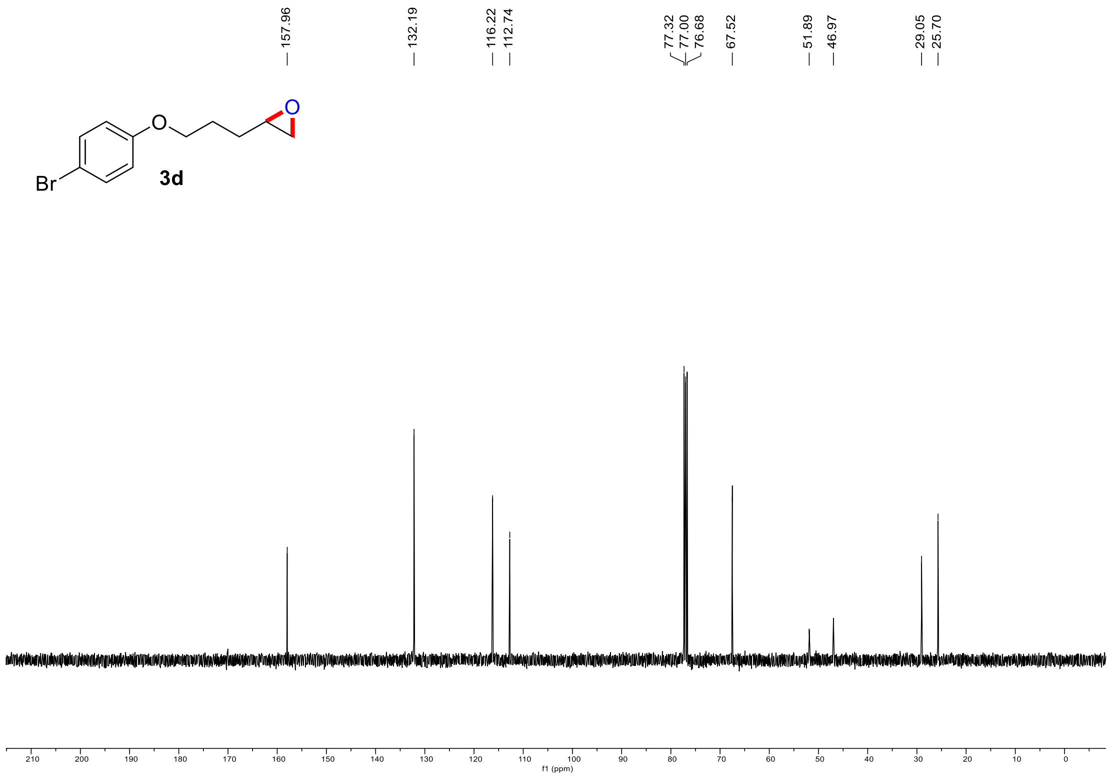

**Supplementary Figure 113**. $^{13}$C NMR of compound **3d** (100 MHz, Chloroform-*d*)

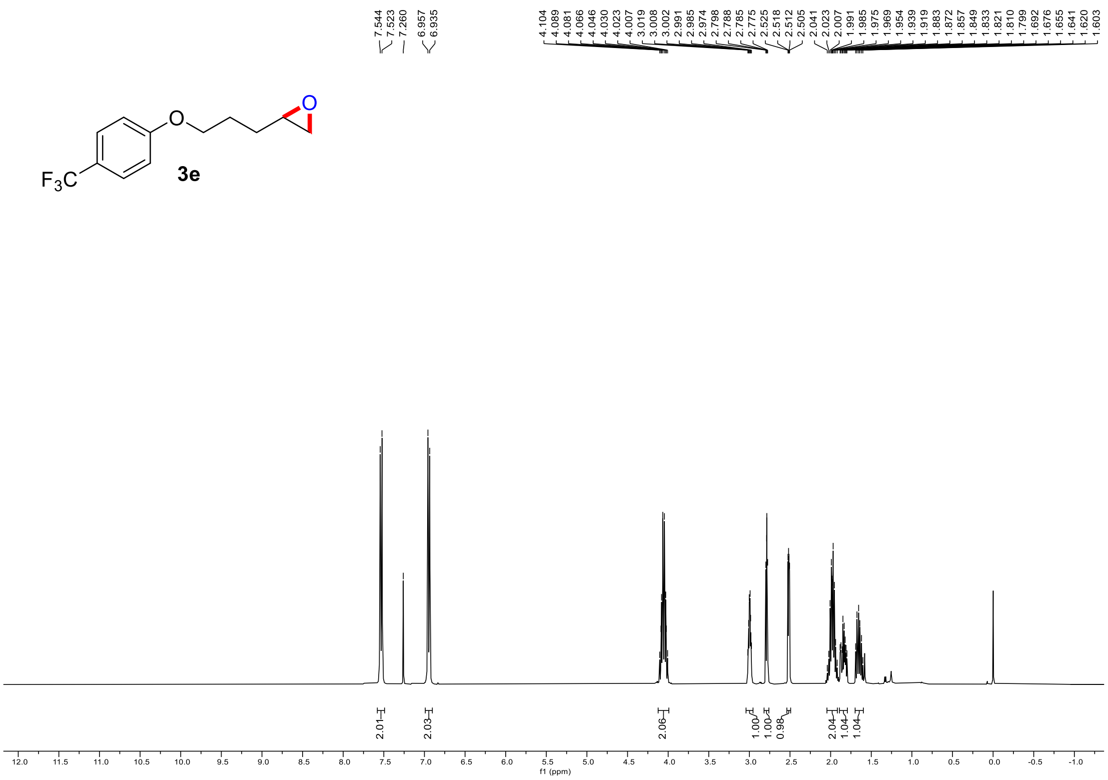

**Supplementary Figure 114**. $^{1}$H NMR of compound **3e** (400 MHz, Chloroform-*d*)

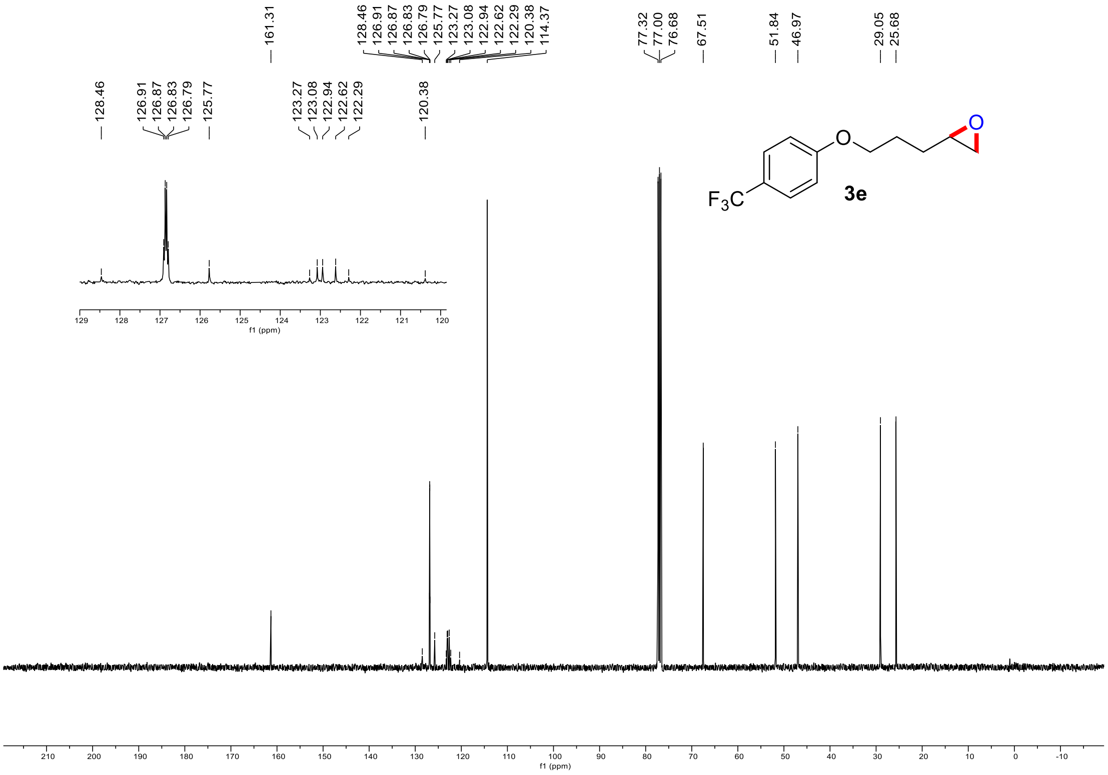

**Supplementary Figure 115**. $^{13}$C NMR of compound **3e** (100 MHz, Chloroform-*d*)

## $^{19}$F NMR of Compound 3e (375 MHz, $CDCl_3$):

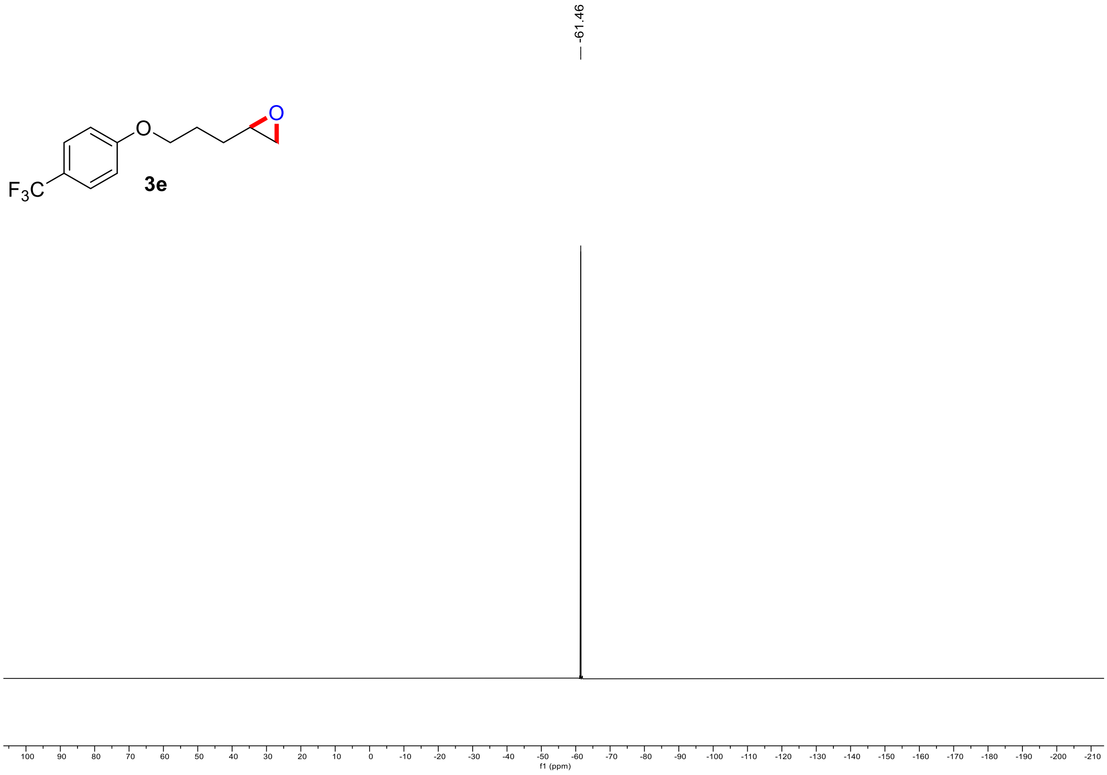


**Supplementary Figure 116**. $^{19}$F NMR of compound **3e** (100 MHz, Chloroform-*d*)

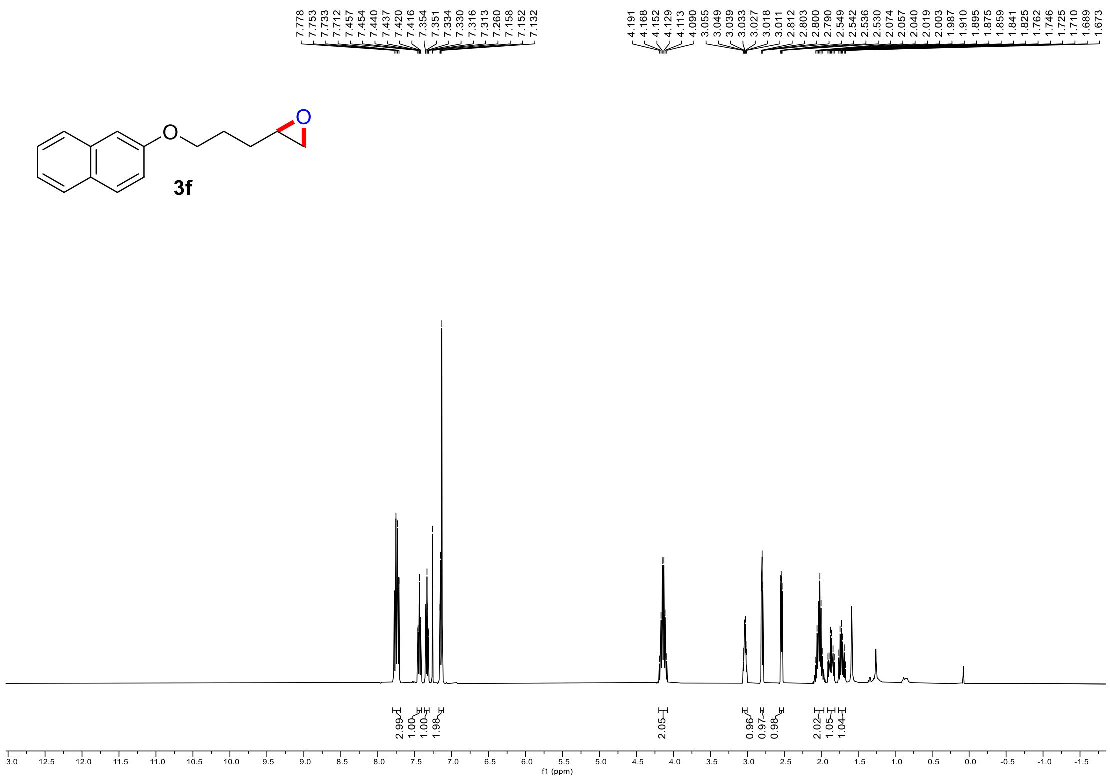

**Supplementary Figure 117**. $^{1}$H NMR of compound **3f** (400 MHz, Chloroform-*d*)

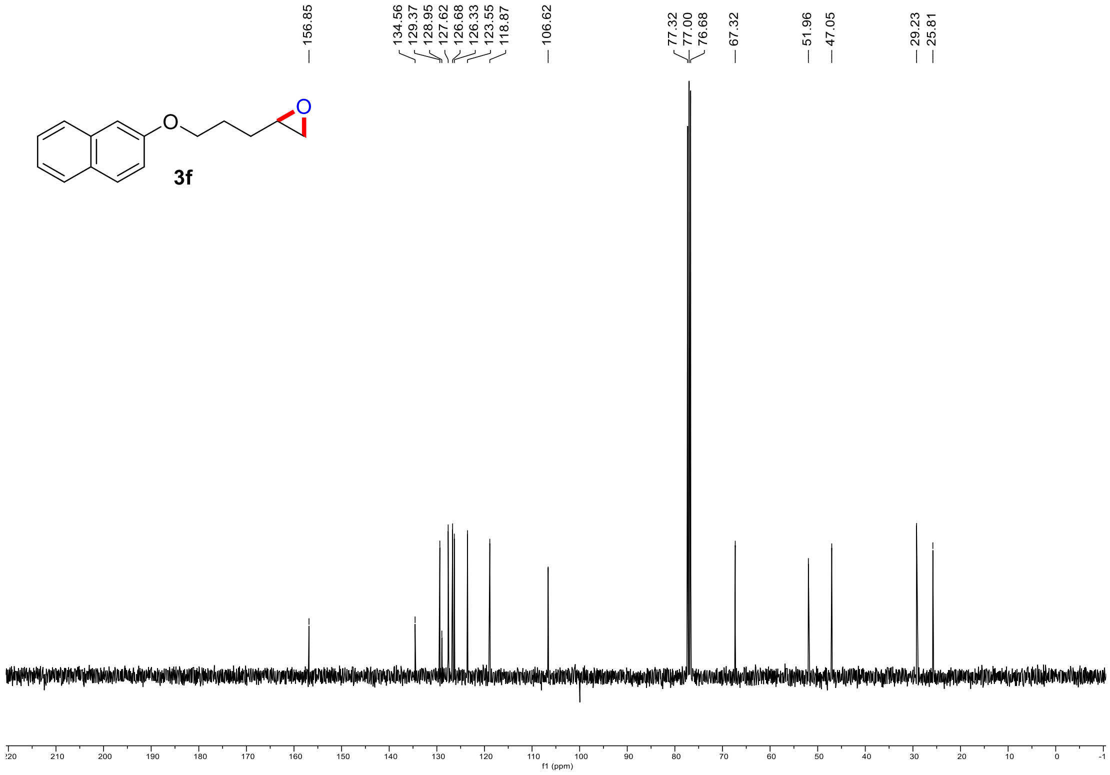

**Supplementary Figure 118**. $^{13}$C NMR of compound **3f** (100 MHz, Chloroform-*d*)

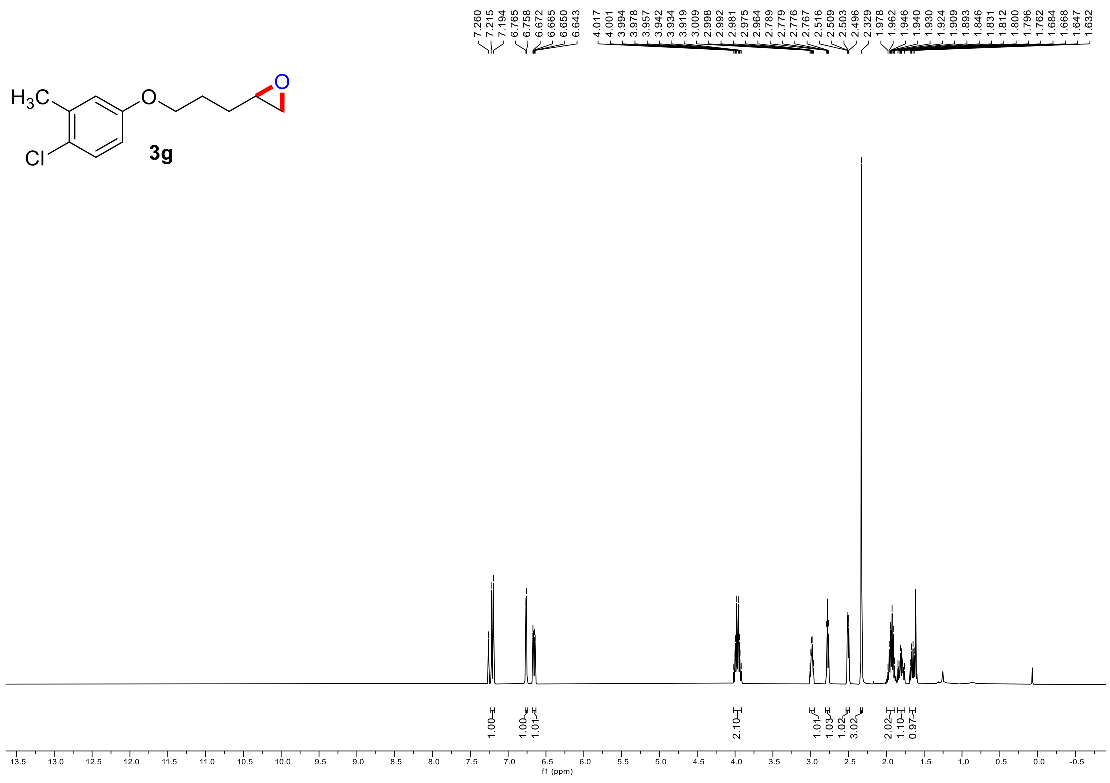

**Supplementary Figure 119**. $^{1}$H NMR of compound **3g** (400 MHz, Chloroform-*d*)

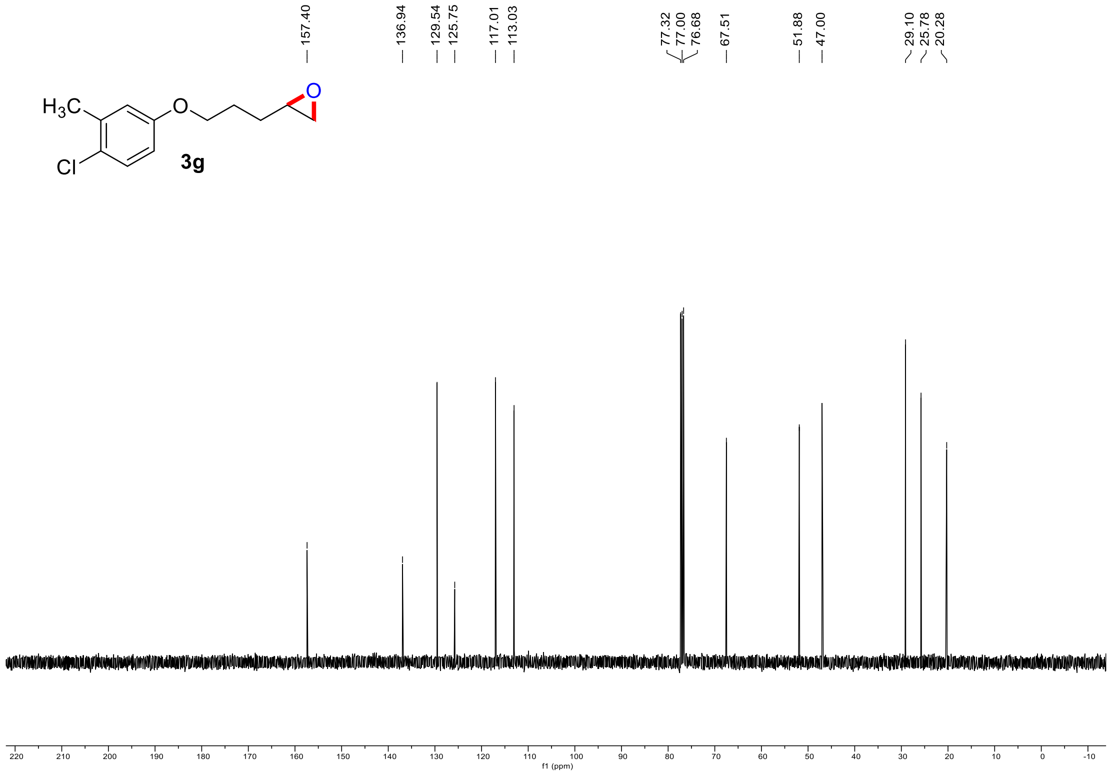

**Supplementary Figure 120**. $^{13}$C NMR of compound **3g** (100 MHz, Chloroform-*d*)

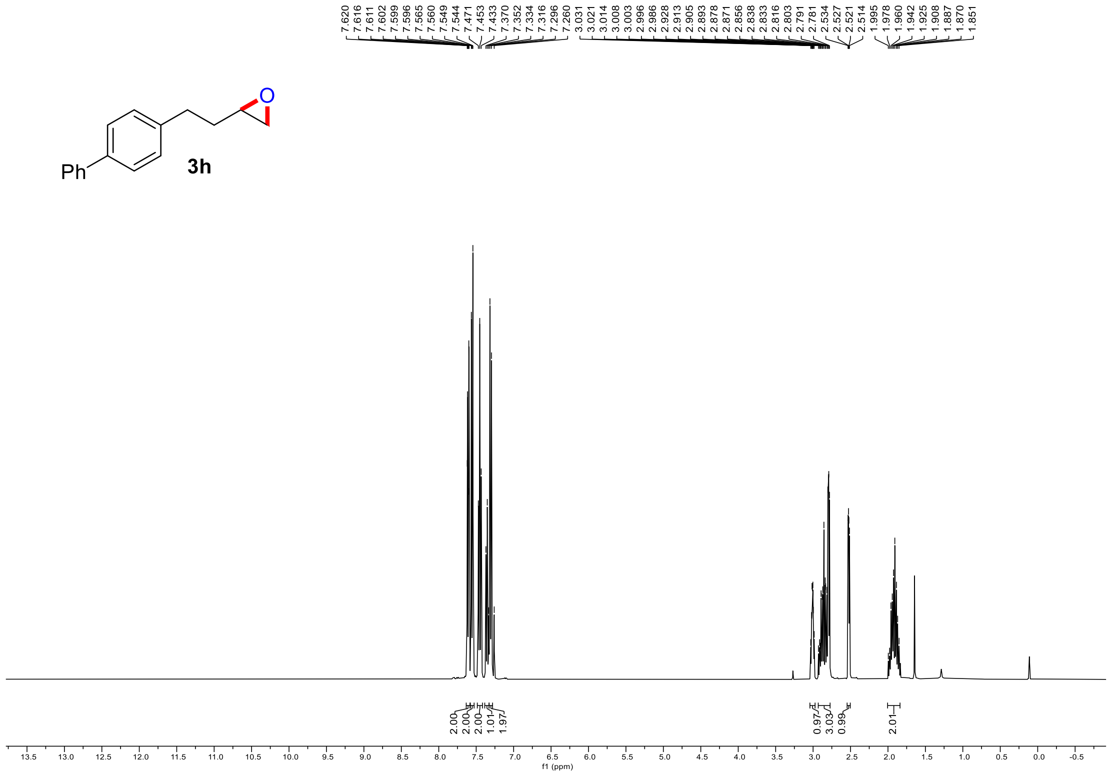


**Supplementary Figure 121**. $^{1}$H NMR of compound **3h** (400 MHz, Chloroform-*d*)

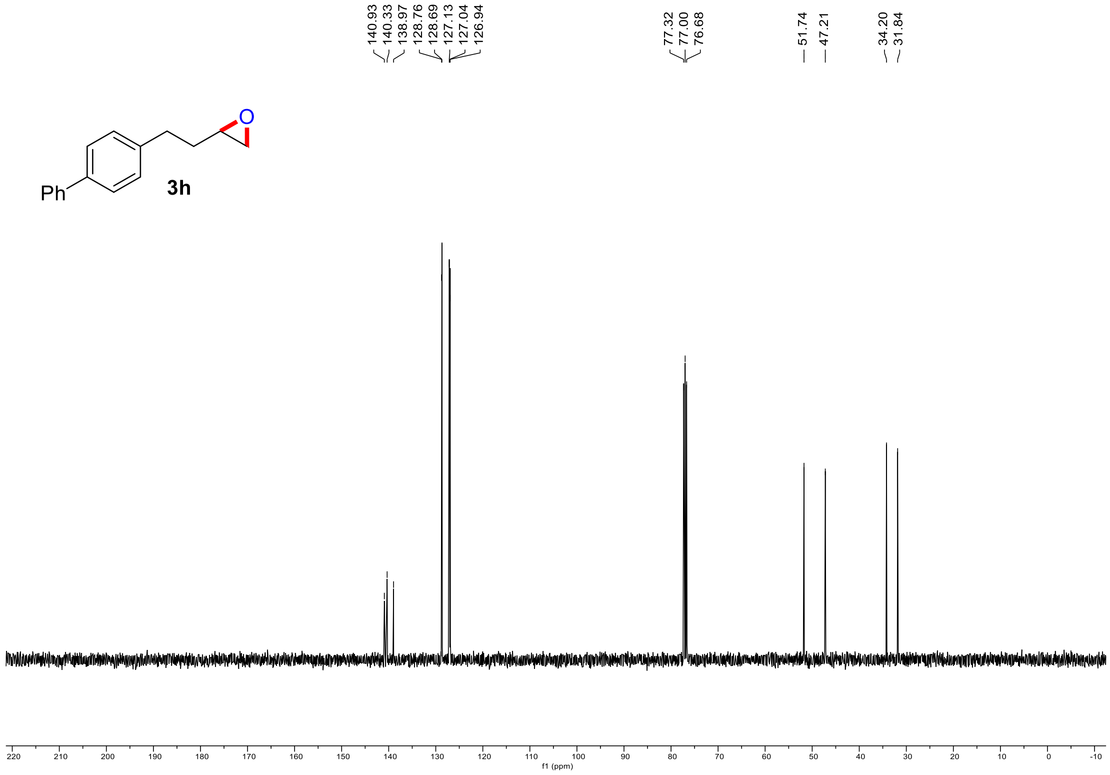


**Supplementary Figure 122**. $^{13}$C NMR of compound **3h** (100 MHz, Chloroform-*d*)

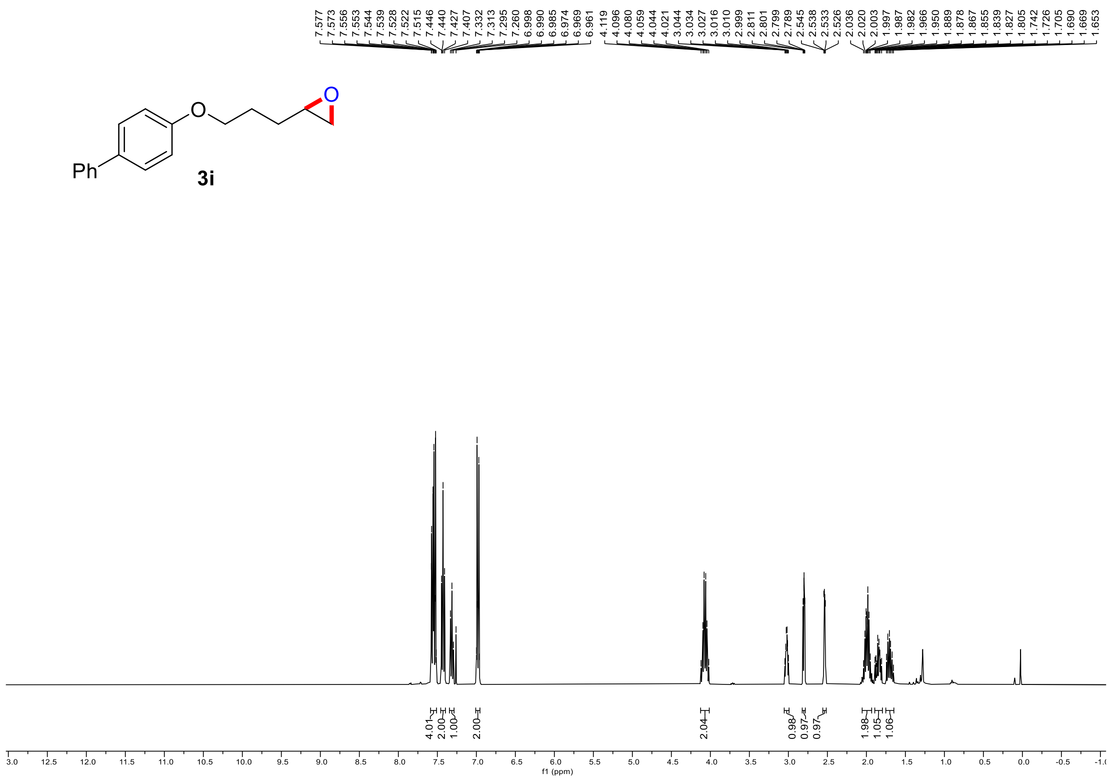


**Supplementary Figure 123**. $^{1}H$ NMR of compound **3i** (400 MHz, Chloroform-*d*)

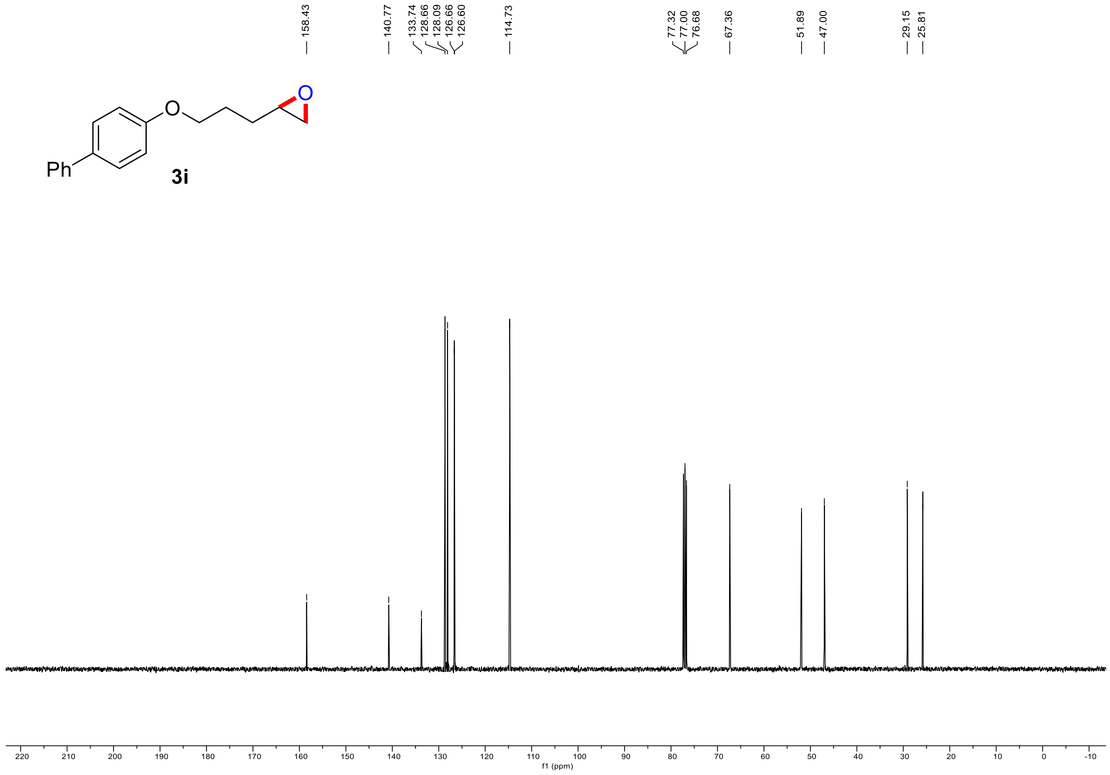


**Supplementary Figure 124**. $^{13}C$ NMR of compound **3i** (100 MHz, Chloroform-*d*)

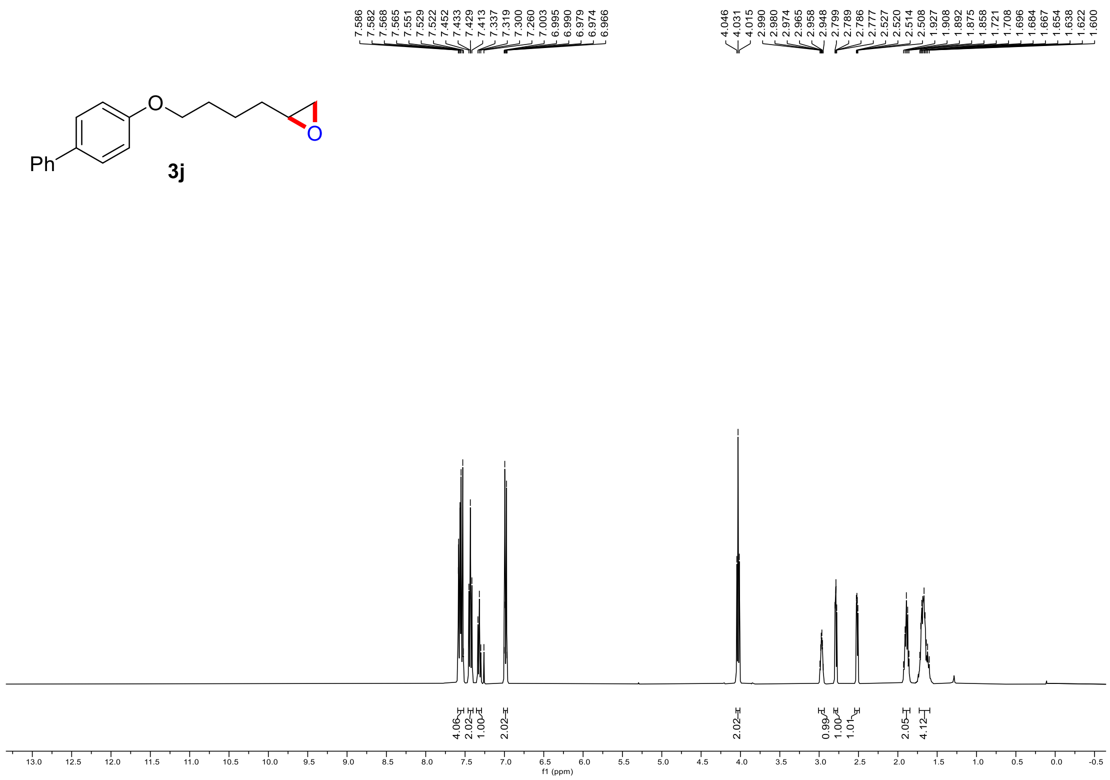

**Supplementary Figure 125**. $^{1}$H NMR of compound **3j** (400 MHz, Chloroform-*d*)

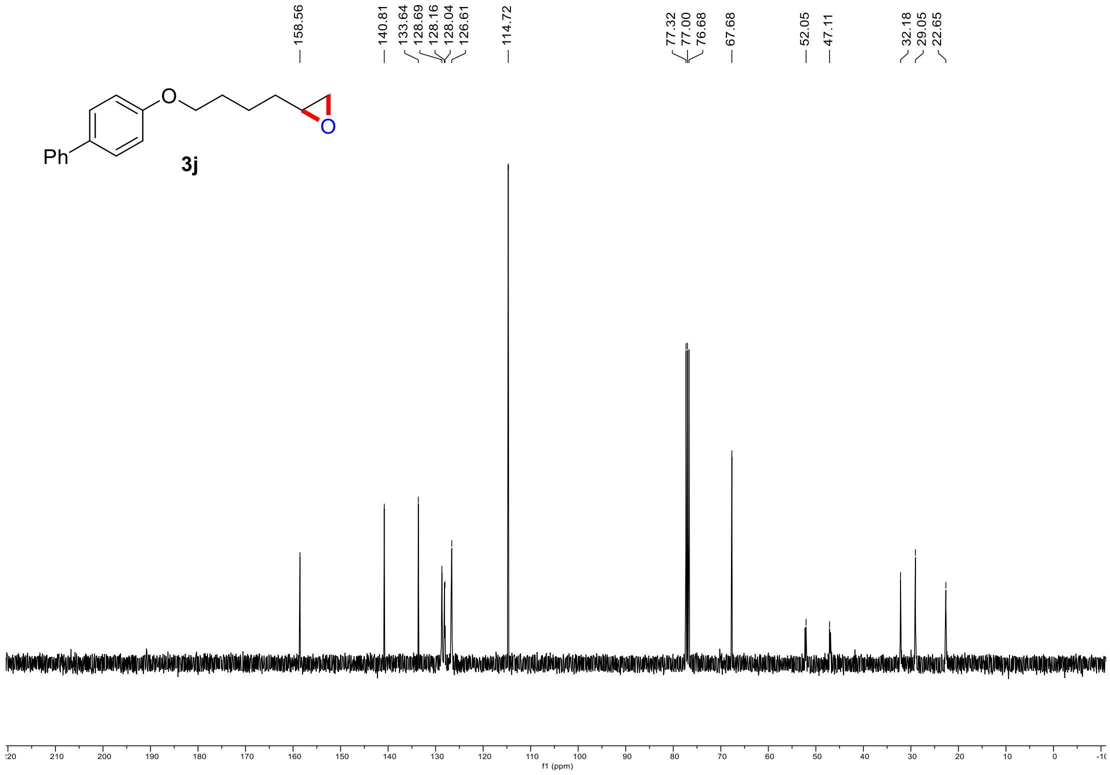

**Supplementary Figure 126**. $^{13}$C NMR of compound **3j** (100 MHz, Chloroform-*d*)

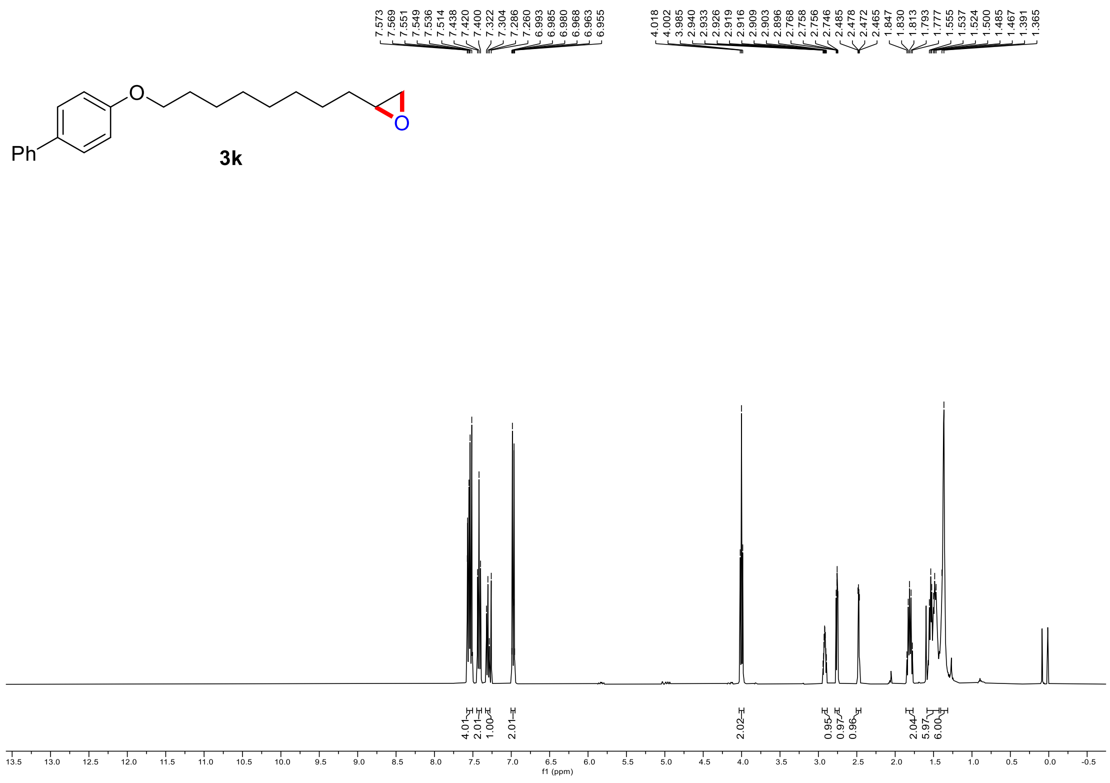

**Supplementary Figure 127**. $^{1}$H NMR of compound **3k** (400 MHz, Chloroform-*d*)

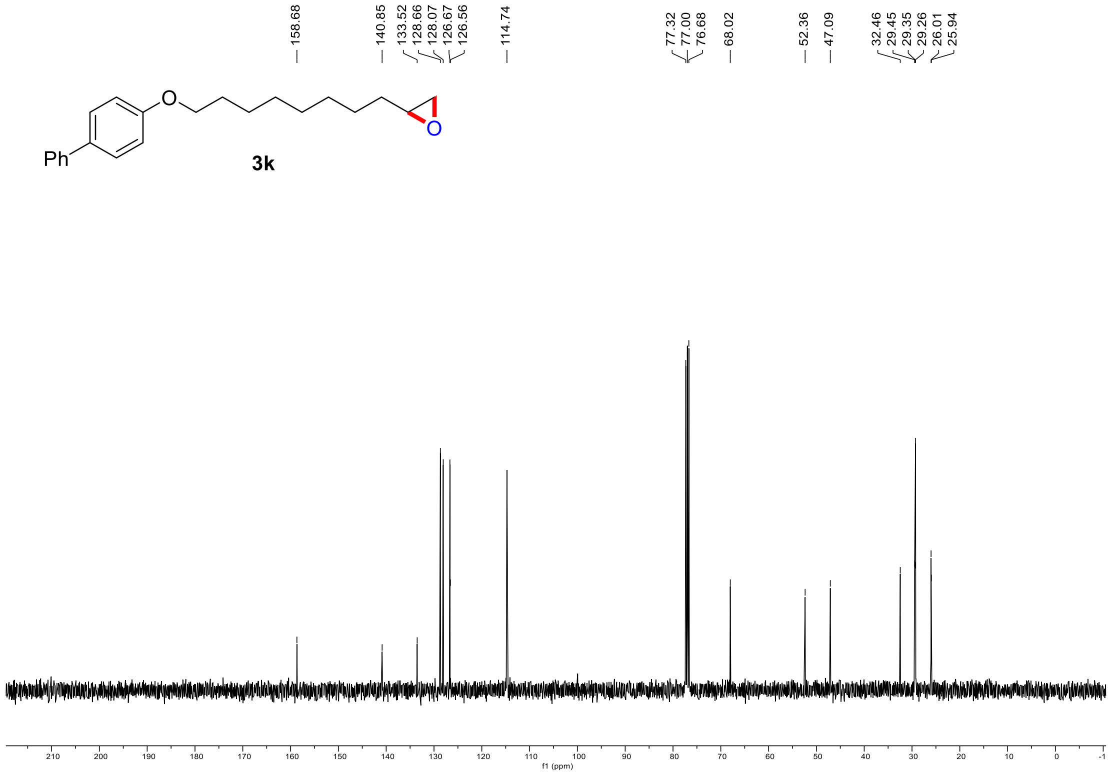

**Supplementary Figure 128**. $^{13}$C NMR of compound **3k** (100 MHz, Chloroform-*d*)

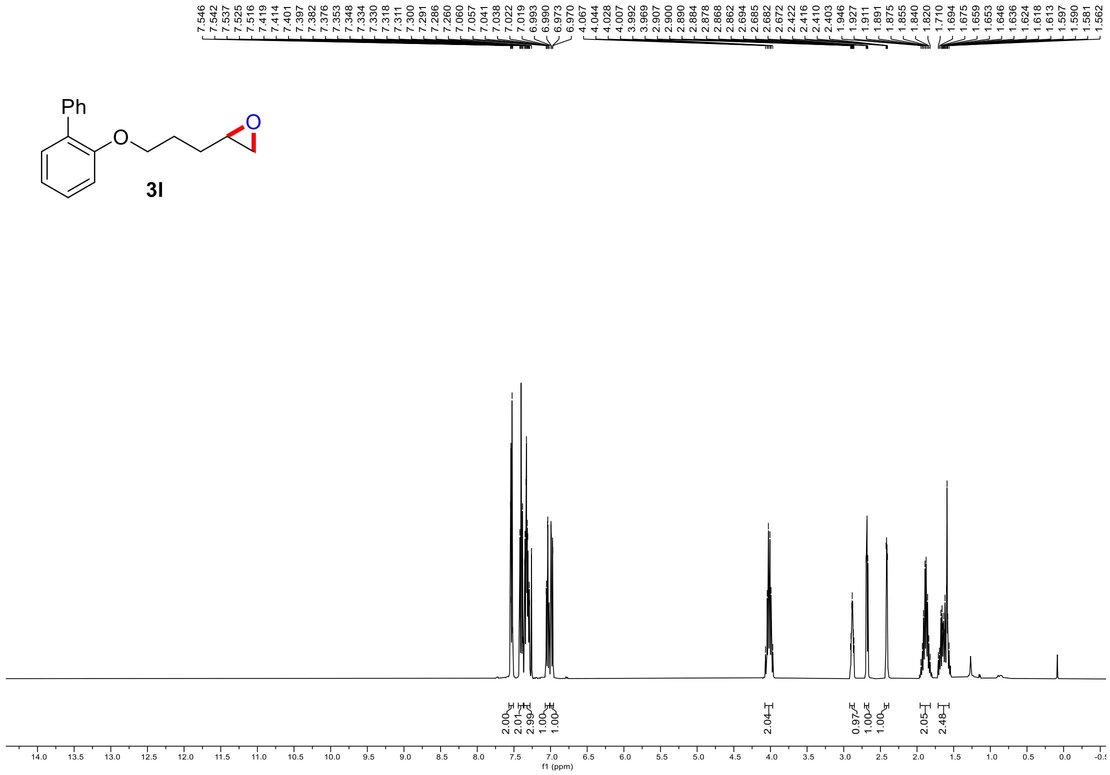


**Supplementary Figure 129**. $^{1}$H NMR of compound **3l** (400 MHz, Chloroform-*d*)

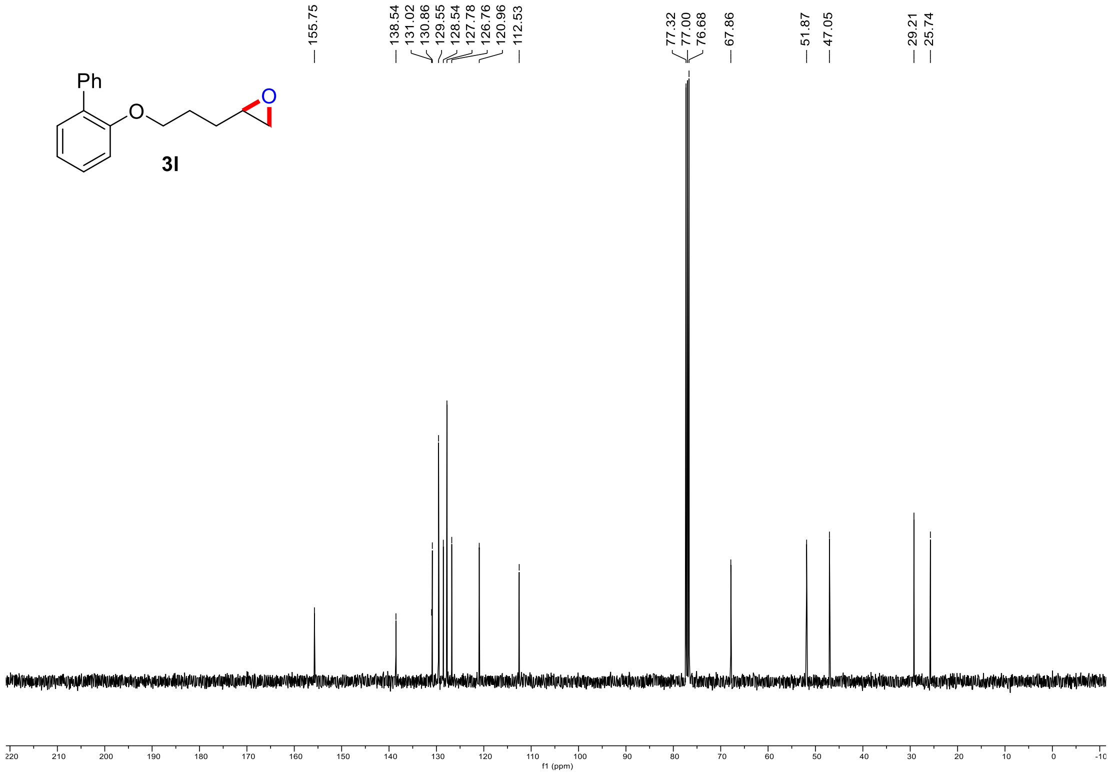


**Supplementary Figure 130**. $^{13}$C NMR of compound **3l** (100 MHz, Chloroform-*d*)

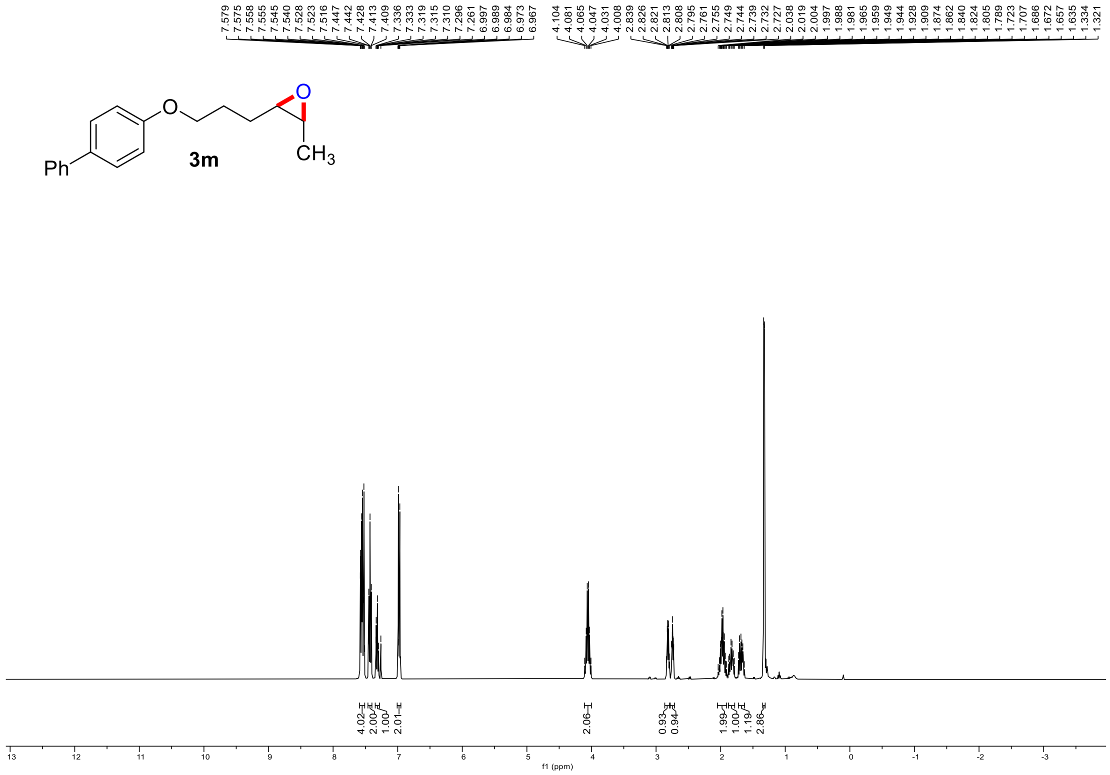


**Supplementary Figure 131**. $^{1}$H NMR of compound **3m** (400 MHz, Chloroform-*d*)

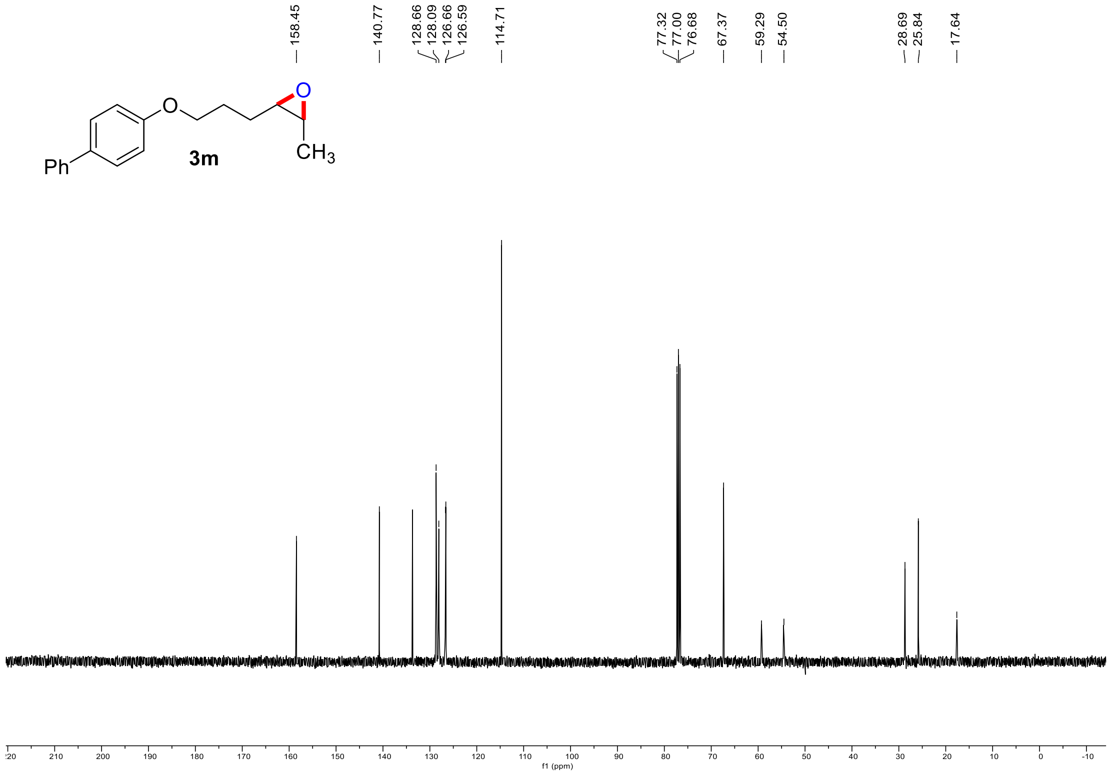


**Supplementary Figure 132**. $^{13}$C NMR of compound **3m** (100 MHz, Chloroform-*d*)

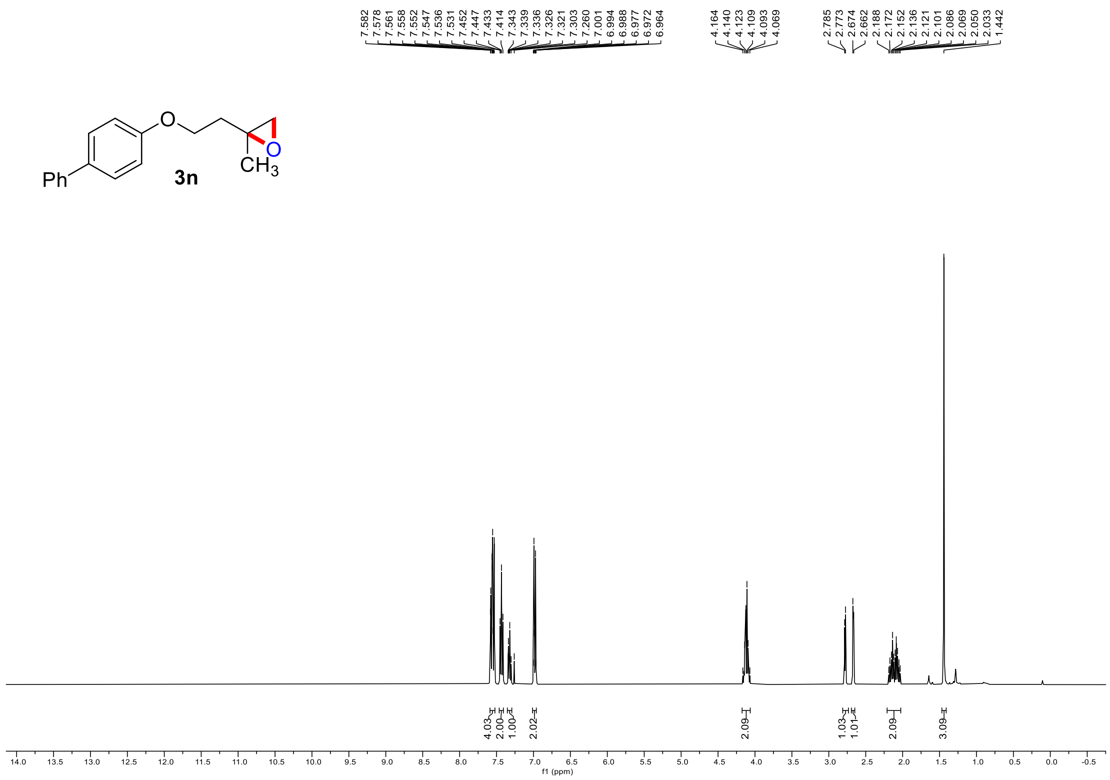


**Supplementary Figure 133**. $^{1}$H NMR of compound **3n** (400 MHz, Chloroform-*d*)

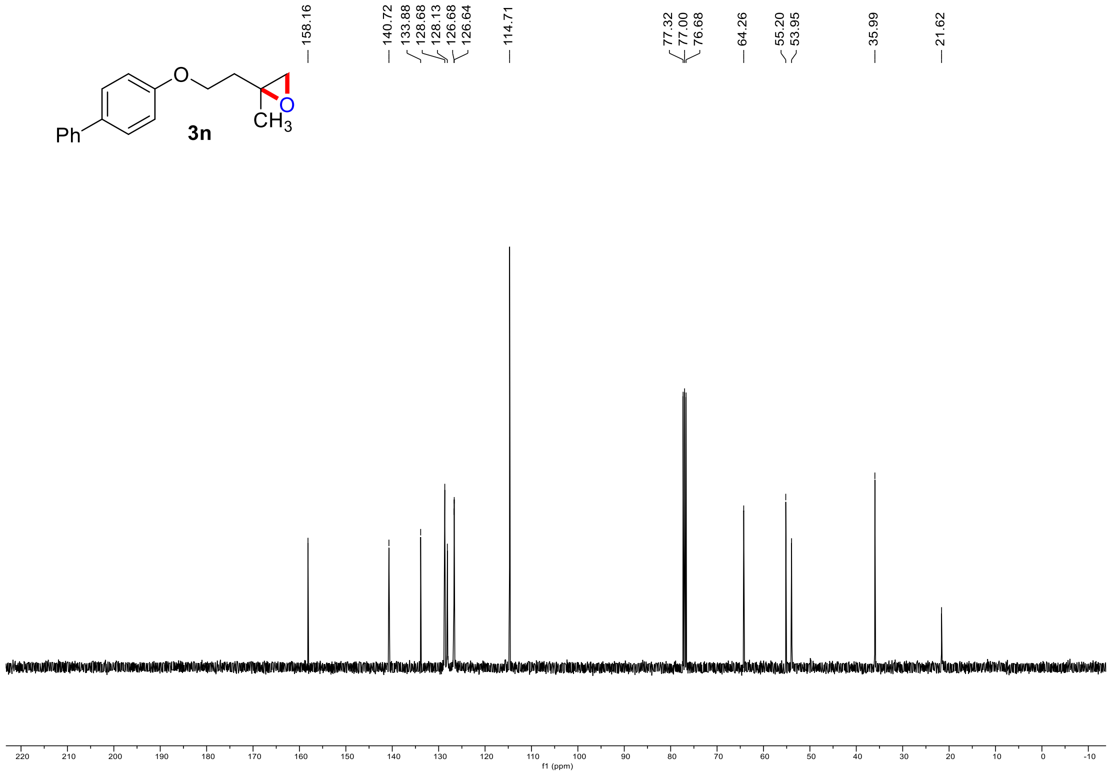


**Supplementary Figure 134**. $^{13}$C NMR of compound **3n** (100 MHz, Chloroform-*d*)

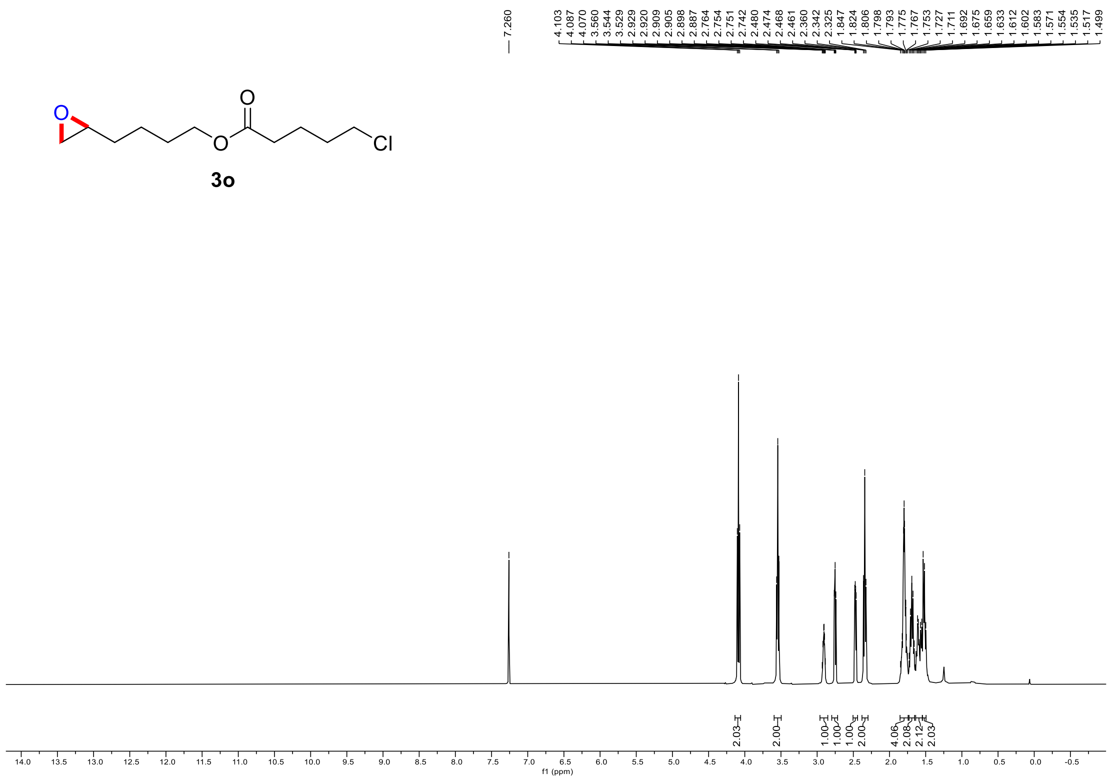


**Supplementary Figure 135**. $^{1}$H NMR of compound **3o** (400 MHz, Chloroform-*d*)

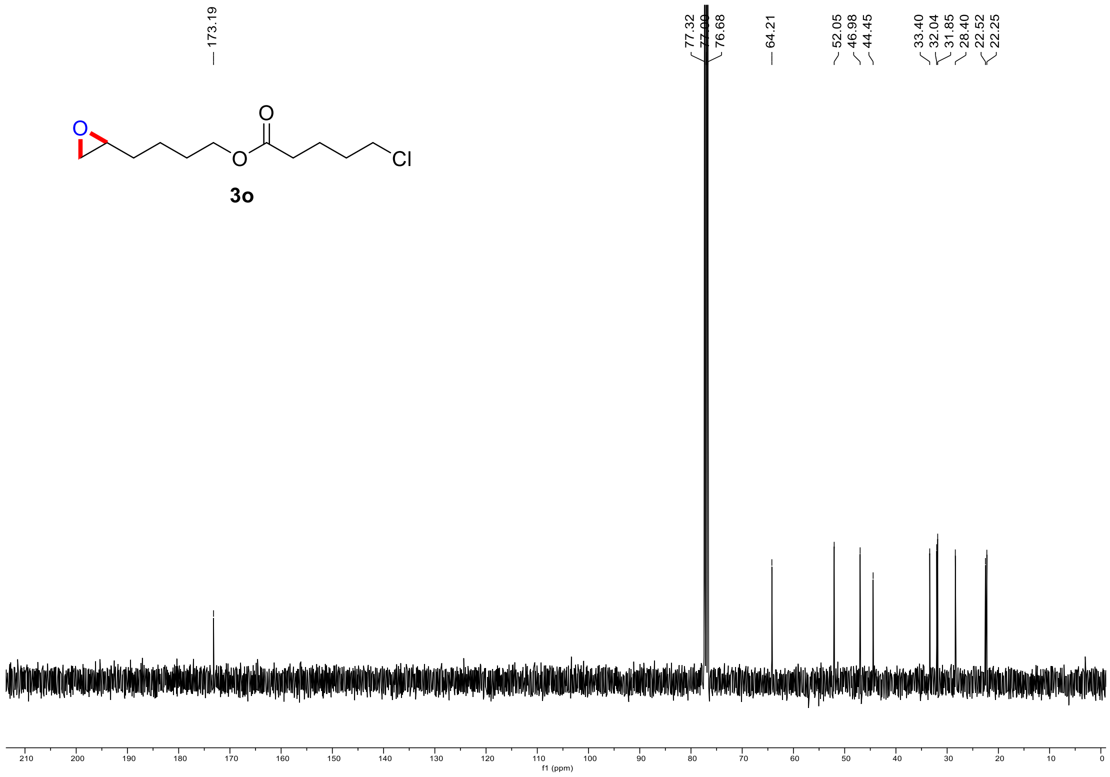


**Supplementary Figure 136**. $^{13}$C NMR of compound **3o** (100 MHz, Chloroform-*d*)

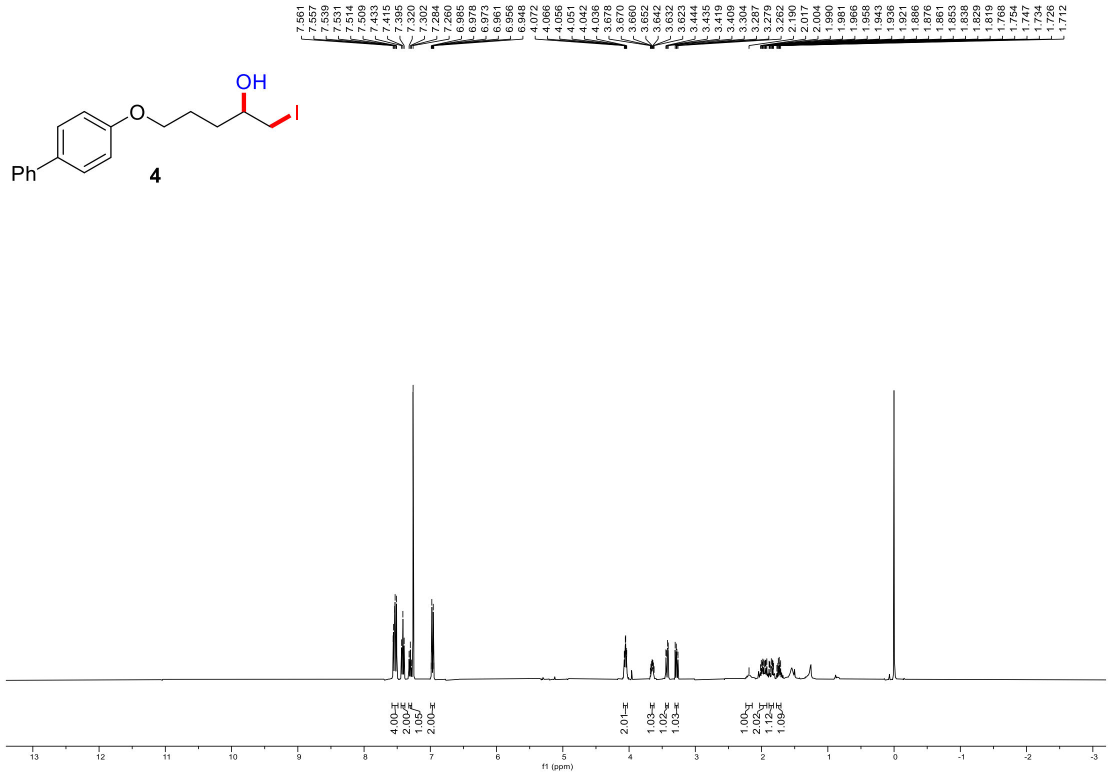

**Supplementary Figure 137**. $^{1}$H NMR of compound **4** (400 MHz, Chloroform-*d*)

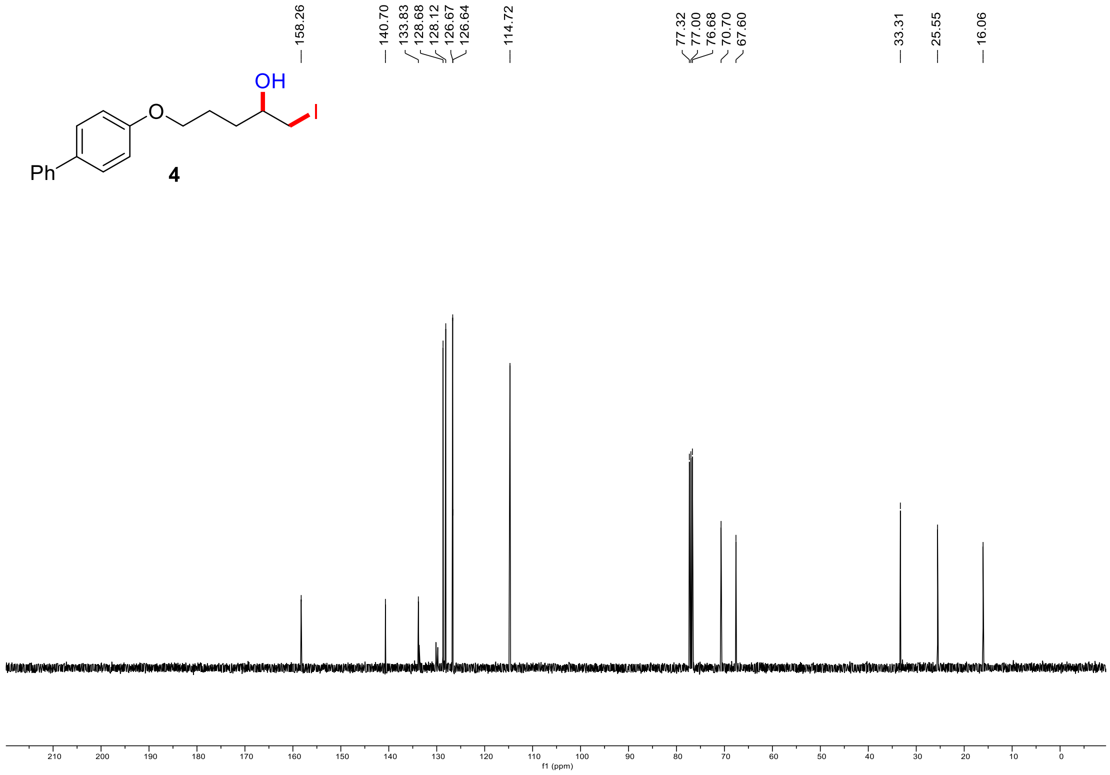

**Supplementary Figure 138**. $^{13}$C NMR of compound **4** (100 MHz, Chloroform-*d*)

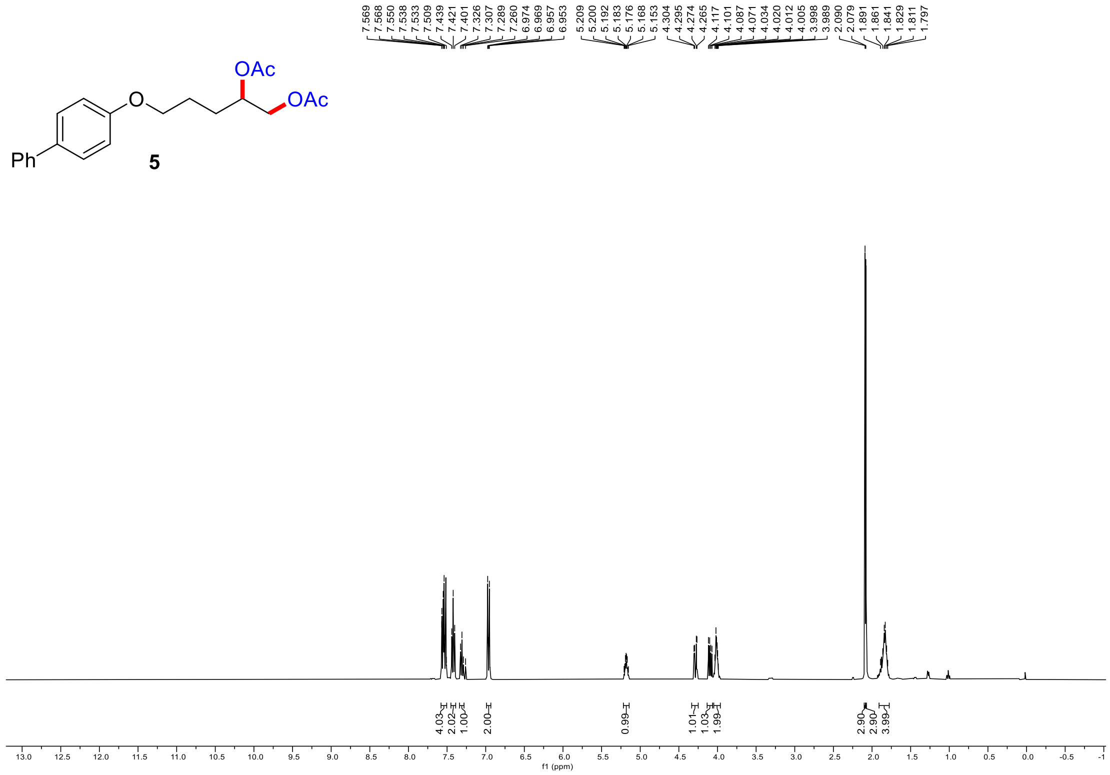

**Supplementary Figure 139**. $^{1}$H NMR of compound **5** (400 MHz, Chloroform-*d*)

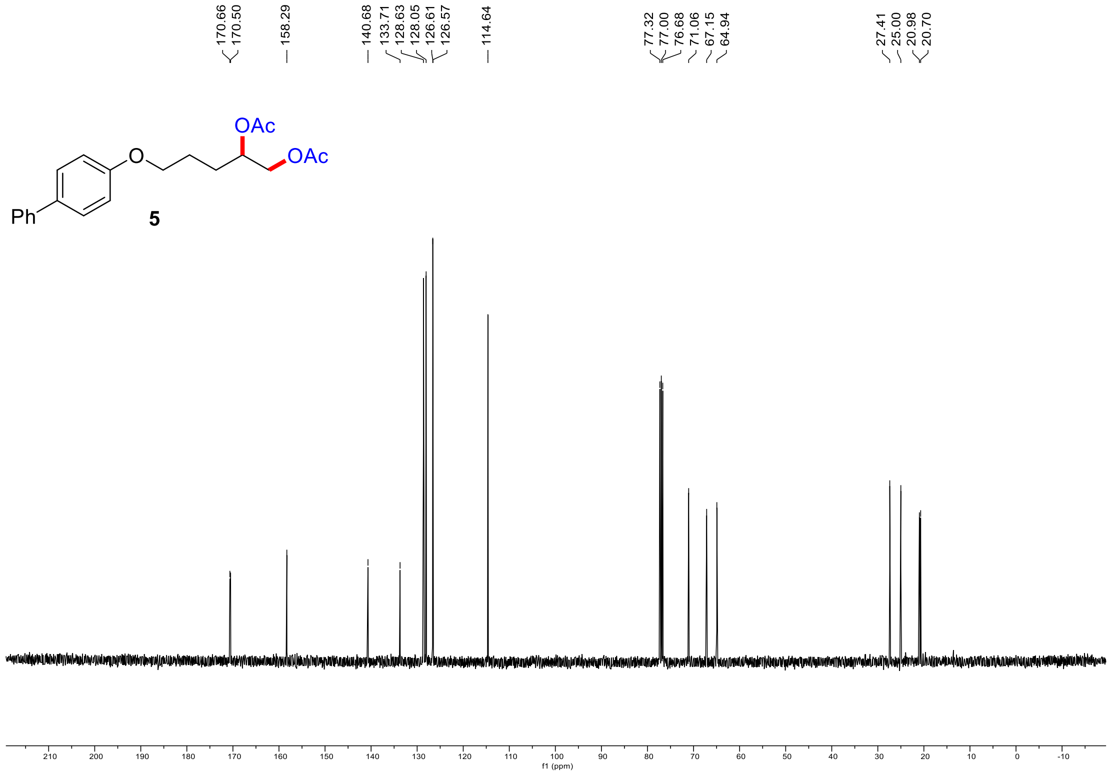

**Supplementary Figure 140**. $^{13}$C NMR of compound **5** (100 MHz, Chloroform-*d*

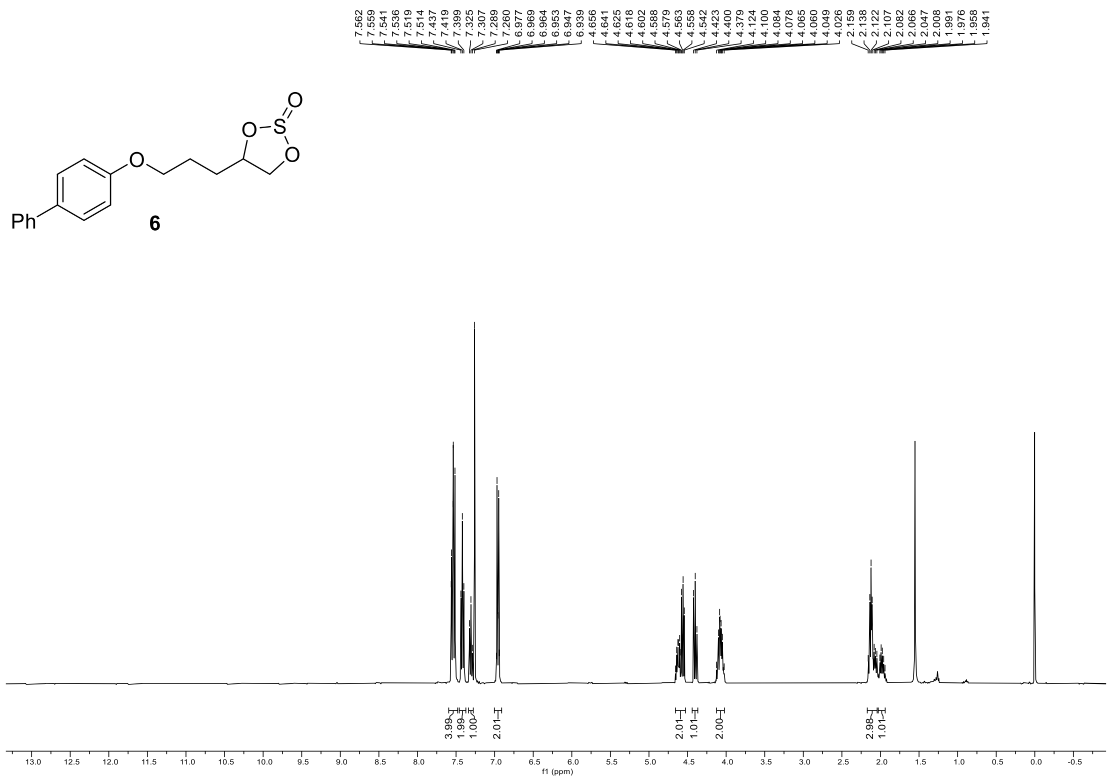


**Supplementary Figure 141**. $^{1}$H NMR of compound **6** (400 MHz, Chloroform-*d*)

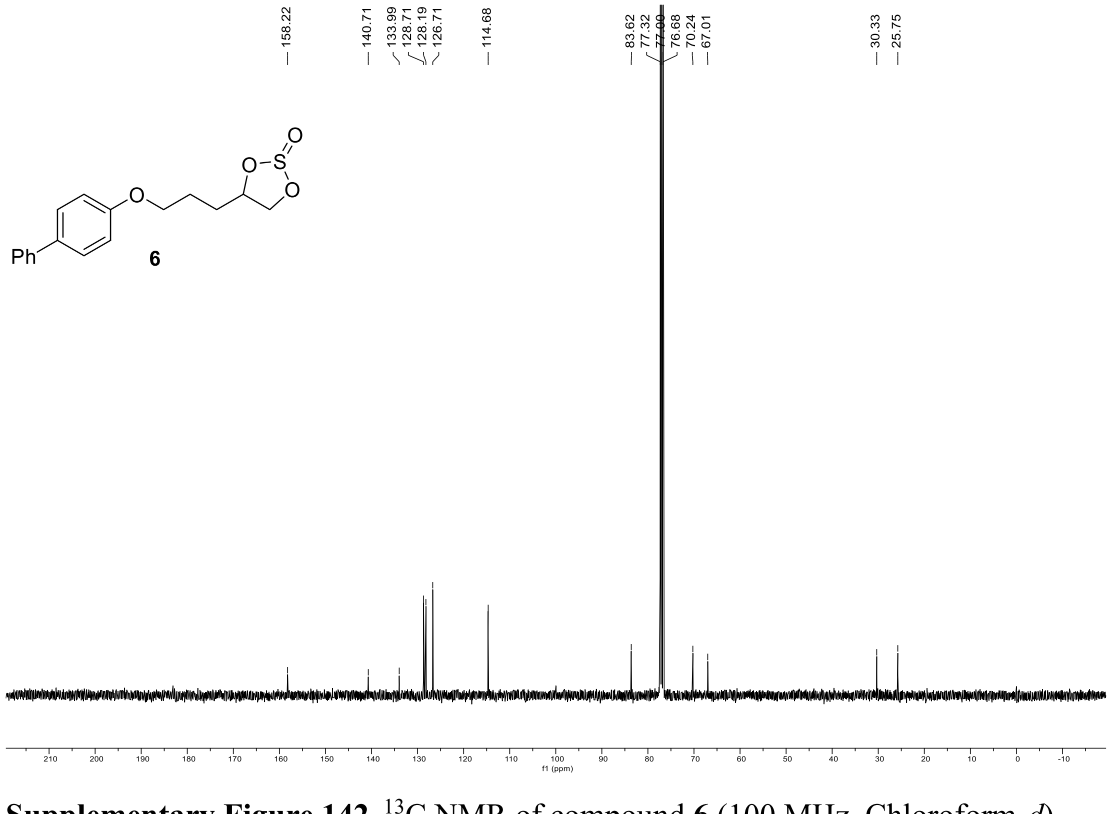


**Supplementary Figure 142**. $^{13}$C NMR of compound **6** (100 MHz, Chloroform-*d*)

**$^1$H NMR of Compound 7 (400 MHz, $CDCl_3$):**

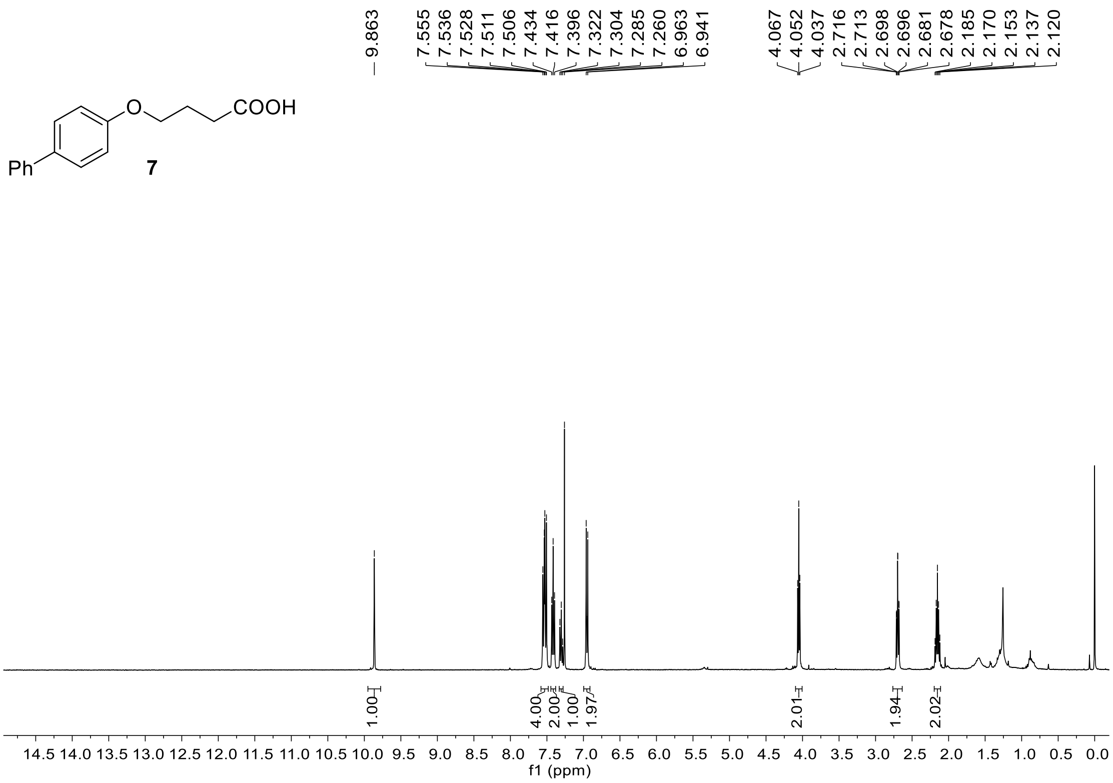


**Supplementary Figure 143**. $^1$H NMR of compound **7** (400 MHz, Chloroform-*d*)

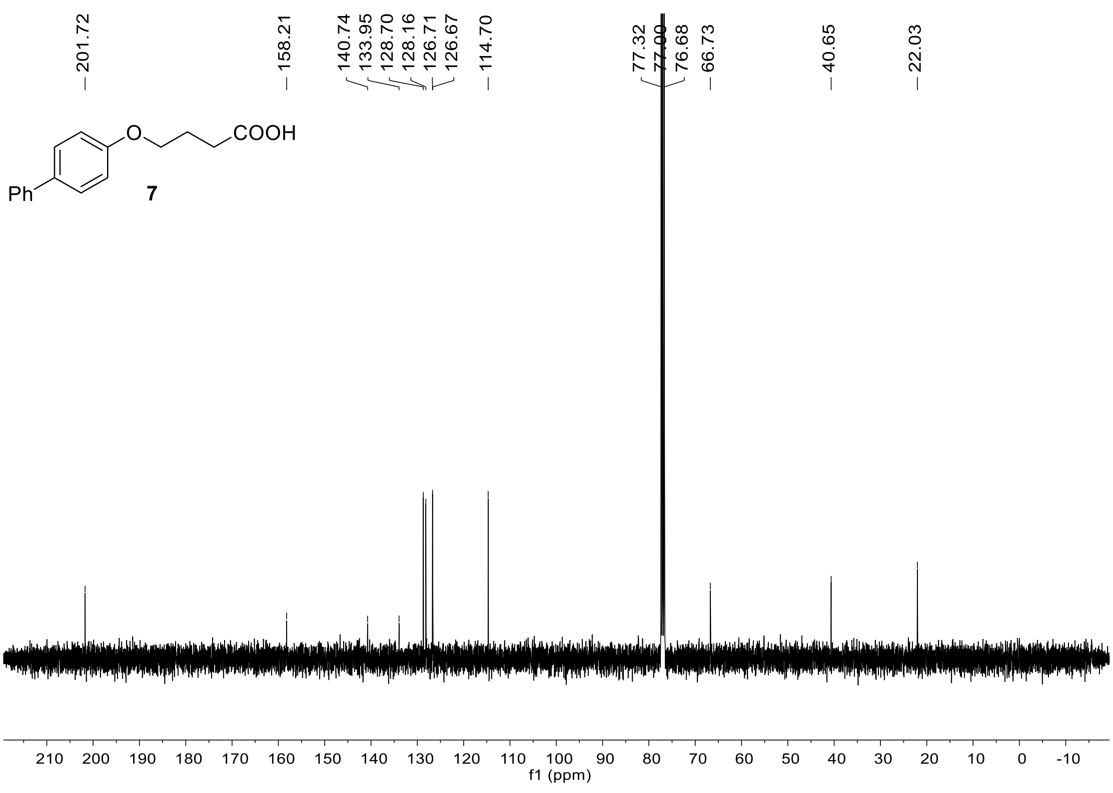


**Supplementary Figure 144**. $^{13}$C NMR of compound **7** (100 MHz, Chloroform-*d*)

## Supplementary References


[1]. R.-Z. Liu, Y.-Y. Zhang, J. Xu, Selective Hydroboration of Equilibrating Allylic Azides. *Chem. Commun*. **2021**, *57*, 8913–8916.

[2]. S. Bordi , J. T. Starr, Hydropyridylation of Olefins by Intramolecular Minisci Reaction. *Org. Lett*. **2017**, *19*, 2290−2293.

[3]. O. E. Okoromoba, Z. Li, N. Robertson, M. S. Mashuta, U. R. Couto, C. F. Tormena, B. Xu, G. B. Hammond, Achieving Regio- and Stereo-control in the Fluorination of Aziridines under Acidic Conditions. *Chem. Commun*. **2016**, *52*, 13353−13356.

[4]. R. Liu, Z.-H. Lu, X.-H. Hu, H.-L. Li, X.-J. Yang, *Org. Lett*. **2015**, *17*, 1489−1492.

[5]. Z.-C. Bao, M. R. Huang, Y. Xu, X.-H. Zhang, Y.-D. Wu, J.-B. Wang, Selective Formal Carbene Insertion into Carbon-Boron Bonds of Diboronates by N-Trisylhydrazones. *Angew. Chem. Int. Ed.* **2023**, *62*, e202216356; *Angew. Chem.* **2023**, *135*, e202216356.

[6]. B. J. Deadman, S. Gian, V. E. Y. Lee, L. A. Adrio, K. Hellgardtb, K. K. Hii, On-demand, in Situ, Generation of Ammonium Caroate (Peroxymonosulfate) for the Dihydroxylation of Alkenes to vicinal diols. *Green Chem*. **2022**, *24*, 5570–5578.

[7]. T. Maegawa, Y. Koutani, K. Otake, H. Fujioka, Methylene Acetal Formation from 1,2- and 1,3-Diols Using an O,S-Acetal, 1,3-Dibromo-5,5-dimethylhydantoin, and BHT. *J. Org. Chem.* **2013**, *78*, 3384−3390.

[8]. G. Xu, G. Yang, Y. Wang, P.-L. Shao, J. N. N. Yau, B. Liu, Y.-B Zhao, Y. Sun, X.-X. Xie, S. Wang, Y. Zhang, L.-X. Xia, Y. Zhao, Stereoconvergent, Redox-Neutral Access to Tetrahydroquinoxalines through Relay Epoxide Opening/Amination of Alcohols*Angew. Chem. Int. Ed.* **2019**, *58*, 14082–14088; *Angew. Chem.* **2019**, *131*, 14082–14088.
